# Development and Optimization of *Kunzea ericoides* Nanoemulgel Using a Quality by Design Approach for Transdermal Anti-Inflammatory Therapy

**DOI:** 10.3390/gels11060400

**Published:** 2025-05-27

**Authors:** Koushik Yetukuri, Marakanam Srinivasan Umashankar

**Affiliations:** Department of Pharmaceutics, SRM Institute of Science and Technology, Kattankulathur, Chengalpattu District, Tamil Nadu 603203, India; yetukurikoushik@gmail.com

**Keywords:** quality by design, nanoemulgel, *Kunzea ericoides*, transdermal delivery, anti-inflammatory, antibacterial, drug release kinetics, central composite design

## Abstract

This study investigates the Quality by Design (QbD)-driven development and optimization of a nanoemulgel incorporating *Kunzea ericoides* oil for transdermal therapy. Nanoemulgels enhance percutaneous drug delivery, sustain release profiles, and improve bioavailability. A central composite design was employed to optimize critical formulation parameters, with ANOVA confirming a statistically significant impact on particle size and drug release kinetics (*p* < 0.05). The optimized formulation exhibited a particle size of 112.38 nm, a polydispersity index of 0.203, and a zeta potential of −29.0 mV, ensuring colloidal stability. In vitro drug release followed the Higuchi model (R^2^ = 0.989, kH = 4.776), indicating diffusion-controlled kinetics, while the Korsmeyer–Peppas model (*n* = 0.88) suggested an anomalous transport mechanism. Antibacterial studies determined minimum inhibitory concentrations of 250 µg/mL for *Staphylococcus aureus* and 500 µg/mL for *Escherichia coli*, indicating greater susceptibility in *S. aureus*. In vivo anti-inflammatory evaluation using a carrageenan-induced paw edema model demonstrated a statistically significant reduction in inflammation (*p* = 0.005 at 60 min), with a near-complete resolution by 240 min. These findings underscore the potential of *Kunzea ericoides* nanoemulgel as a promising transdermal therapeutic, integrating controlled drug release with potent anti-inflammatory and antibacterial properties for dermatological and inflammatory conditions.

## 1. Introduction

The protective function of the skin is often compromised by environmental stressors, intrinsic aging, and pathological conditions, leading to oxidative stress, inflammation, and impaired tissue repair mechanisms [1]. Among these factors, ultraviolet (UV) radiation induces reactive oxygen species (ROS) generation, triggering lipid peroxidation, DNA damage, and mitochondrial dysfunction, all of which contribute to photoaging and inflammatory disorders. Chronic skin conditions such as acne, eczema, and psoriasis further disrupt the epidermal barrier, alter cutaneous microbiota, and exacerbate inflammatory responses, diminishing the skin’s regenerative capacity [2]. Natural bioactive compounds with anti-inflammatory, antimicrobial, and antioxidant properties are increasingly explored for skin repair and protection. *Kunzea ericoides oil* (Kanuka oil) exhibits antibacterial, wound-healing, and free radical-scavenging properties. However, its clinical application is limited by poor solubility, instability, and inadequate skin penetration [3].

To overcome these challenges, nanoemulsions (NEs) offer an advanced drug delivery approach, improving solubility, enhancing skin permeation, and enabling controlled drug release. Nanoemulgel systems are classified based on the type of nanoemulsion used, such as oil-in-water (O/W) or water-in-oil (W/O), and the nature of the gelling agent incorporated. Typically, an O/W nanoemulsion is preferred for topical delivery due to better skin compatibility and enhanced release of hydrophobic drugs. The preparation of nanoemulgel involves two key steps: first, the formation of a stable nanoemulsion using high-energy techniques (such as ultrasonication or high-pressure homogenization) or low-energy methods (such as spontaneous emulsification); second, the incorporation of the nanoemulsion into a hydrogel matrix. Commonly used gelling agents include synthetic polymers like Carbopol 940 and natural polymers such as xanthan gum or HPMC, which provide the desired viscosity, spreadability, and stability. These systems enhance skin adhesion, allow for controlled release, and improve patient compliance. The type and concentration of oil, surfactant/co-surfactant, and gelling agent all influence the physicochemical characteristics and therapeutic efficacy of the final formulation. Incorporating NEs into a gel matrix (nanoemulgel) enhances viscosity, prolongs skin retention, and optimizes therapeutic efficacy for transdermal applications [4].

This study aims to develop and optimize a Kanuka-loaded nanoemulgel using the Quality by Design (QbD) approach, employing Central Composite Design (CCD) to assess the impact of formulation parameters on stability, drug release, and therapeutic performance. The optimized formulation was subjected to physicochemical characterization, in vitro drug release studies, antibacterial assessment, and in vivo anti-inflammatory evaluation using the carrageenan-induced hind paw edema model in Wistar rats. The results demonstrate the potential of Kanuka nanoemulgel as a transdermal therapeutic system with sustained drug release and dual antibacterial and anti-inflammatory activity for dermatological applications.

## 2. Results and Discussion

### 2.1. FT-IR Analysis of Kanuka Oil

**Figure 1:**
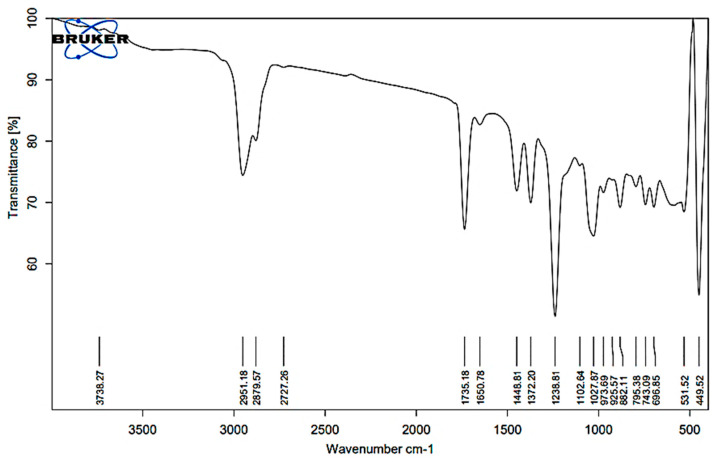
FT-IR spectrum of Kanuka oil, confirming key functional groups: O–H (3738 cm^−1^), C–H (2951, 2917, 2772 cm^−1^), C=O (1735 cm^−1^), C=C (1653 cm^−1^), and C–O (1243–1027 cm^−1^), indicating the presence of monoterpenes, sesquiterpenes, esters, and alcohols.

The IR spectrum of Kanuka oil exhibits characteristic peaks corresponding to various functional groups, confirming its complex chemical composition. A broad absorption band at 3738.27 cm^−1^ corresponds to the free hydroxyl (O–H) stretching mode, indicative of phenols and alcohols (Figure 1). Peaks at 2951.18, 2927.57, and 2727.26 cm^−1^ correspond to C–H stretching vibrations, indicating the presence of aliphatic chains, including alkanes and other saturated hydrocarbons. The intense C=O stretching bands at 1735.18 and 1650.78 cm^−1^ suggest the presence of esters and ketones, which are common constituents of essential oils and may contribute to their fragrance and stability. Further evidence of aliphatic structures is supported by C–H bending peaks at 1448.81 and 1322.20 cm^−1^. Strong absorption bands at 1123.89 and 1102.64 cm^−1^ correspond to C–O stretching, indicating ether or alcohol functionalities.

Additionally, peaks at 1027.69, 787.17, and 882.51 cm^−1^ suggest C=C bending, confirming the presence of aromatic rings, likely representing monoterpenes and sesquiterpenes. The fingerprint region, with peaks at 531.52 and 449.52 cm^−1^, provides unique spectral characteristics specific to Kanuka oil. The functional group assignments corresponding to the observed peaks are summarized in Table 1, which provides the accepted wavenumber ranges for each functional group. The results confirm the presence of bioactive monoterpenes, sesquiterpenes, esters, and alcohols, validating the chemical integrity of Kanuka oil for formulation studies.

### 2.2. GC-MS Analysis of Kanuka Oil

**Figure 2:**
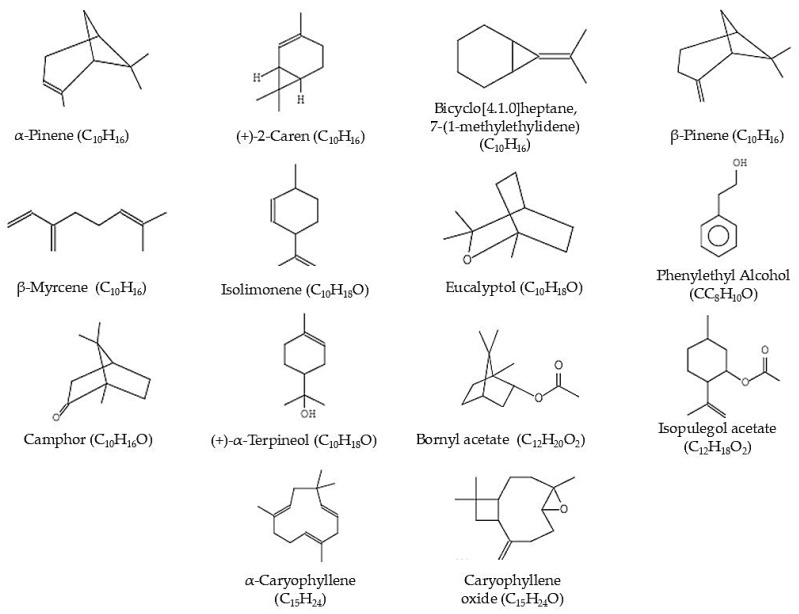
Chemical structures of major constituents identified in Kanuka oil by GC-MS, representing diverse chemical classes such as monoterpenes, sesquiterpenes, esters, alcohols, and ketones that contribute to its therapeutic properties.

The GC-MS analysis of Kanuka oil led to the identification of 119 compounds, among which 14 major constituents were selected based on their relative peak area and therapeutic relevance (Table 2, Figure 2). For instance, α-Pinene (C_10_H_16_, Mol. Wt. 136.24) was observed at RT 4.085 min with a peak area of 0.06%, known for its anti-inflammatory and bronchodilatory effects. (+)-2-Caren (C_10_H_16_, Mol. Wt. 136.24), eluting at 4.361 min and contributing 0.04%, is associated with antimicrobial properties. Bicyclo[4.1.0]heptane, 7-(1-methylethylidene)-(C_10_H_16_, Mol. Wt. 136.24), identified at RT 5.158 min with 9.31% peak area, has potential in anti-inflammatory and bronchodilatory therapies. Similarly, β-Pinene (C_10_H_16_, Mol. Wt. 136.24) appears at RT 5.559 min with 1.63%, and β-Myrcene (C_10_H_16_, Mol. Wt. 134.24) at 5.811 min with 0.27%, both contributing to the anti-inflammatory effects. Isolimonene (C_10_H_16_, Mol. Wt. 136.24) was detected at RT 6.439 min, contributing 9.76%, and is known for its antioxidant and antimicrobial activities. Eucalyptol (C_10_H_18_O, Mol. Wt. 154.25), observed at 6.464 min with 1.32%, has anti-inflammatory properties. Phenylethyl alcohol (C_8_H_10_O, Mol. Wt. 122.16), appearing at 7.834 min with 2.45%, contributes to the oil’s fragrance and antimicrobial activity. Camphor (C_10_H_16_O, Mol. Wt. 152.23), eluting at 8.195 min with 2.32%, is recognized for its anti-inflammatory and analgesic effects. (+)-α-Terpineol (C_10_H_18_O, Mol. Wt. 154.25) at RT 8.956 min (6.32%) is known for its antimicrobial and anti-inflammatory activities. The presence of Bornyl acetate (C_10_H_16_O_2_, Mol. Wt. 168.23) at RT 9.625 min with 0.18%, Isopulegol acetate (C_10_H_18_O_2_, Mol. Wt. 170.25) at RT 9.736 min with 0.45%, and α-Caryophyllene (C_15_H_24_, Mol. Wt. 204.35) at RT 12.445 min with 0.04%, further supports the oil’s anti-inflammatory and analgesic properties. Other significant compounds such as Caryophyllene oxide (C_15_H_24_O, Mol. Wt. 220.35) at RT 13.896 min with 0.21%, contribute to the oil’s therapeutic profile. The diversity and abundance of these constituents suggest that Kanuka oil possesses strong anti-inflammatory, antimicrobial, and antioxidant properties. The complete GC-MS analysis report, including the full list of compounds and chromatogram, is available in Supplementary Material File S1.

### 2.3. Preparation and Optimization of Kanuka Nanoemulsions

Nanoemulsions were optimized according to the predefined criteria levels and specific HLB values, with droplet size as the response parameter. Table 3 provides an overview of the experimental design matrix and the corresponding observed results. It was observed that the particle size of the formulation is significantly influenced by Tween 80 concentration (Factor 1) and homogenization time (Factor 2). From the experimental data, the smallest particle size (100.65 nm) was observed when Tween 80 was at its center level (X_1_ = 0) and homogenization time was at its highest axial level (X_2_ = A). This indicates that increasing homogenization time enhances particle size reduction, likely due to greater shear forces breaking down droplet size more effectively. In contrast, the largest particle size (184.9 nm) was obtained at the lowest levels of both factors (X_1_ = −, X_2_ = −), suggesting that insufficient surfactant leads to poor emulsification, while shorter homogenization time results in inadequate particle size reduction.

Additionally, excessive Tween 80 did not significantly reduce particle size further, implying that an optimal concentration is necessary to stabilize the formulation without excessive micelle formation. Thus, to achieve a minimum particle size, a combination of moderate Tween 80 concentration and higher homogenization time is recommended. After data fitting, JMP software generated polynomial model equations for the primary factors and their interactions. the quadratic (second-order polynomial) model equation for particle size (PS) can be written in the standard form:

PS = 122.68 − 10.43X_1_ − 21.67X_2_ − 0.31X_1_X_2_ + 10.64X_1_^2^ + 4.03X_2_^2^ (1)

where X_1_ represents the Tween 80 concentration (2–6%), and X_2_ denotes the homogenization time (10–30 min) in coded values. The model suggests that both factors significantly influence particle size, with higher Tween 80 and increased homogenization time reducing droplet size. However, a quadratic effect for Tween 80 indicates an optimal concentration beyond which further addition may not enhance emulsification. The ANOVA analysis (Table 4) demonstrates that the model is statistically valid (*p* = 0.0092), indicating that Tween 80 concentration and homogenization time have a strong influence on particle size reduction. The model explains 84.62% of the variation in particle size (R^2^ = 0.8462), and the adjusted R^2^ value of 0.7942 accounts for predictor adjustments, confirming a good fit. Among the individual factors, Tween 80 (*p* = 0.0426) and homogenization time (*p* = 0.0013) significantly reduce particle size. The quadratic term for Tween 80 (*p* = 0.0509) suggests a non-linear effect, while the homogenization time squared term was not significant (*p* = 0.4027), indicating a more linear influence.

**Figure 3:**
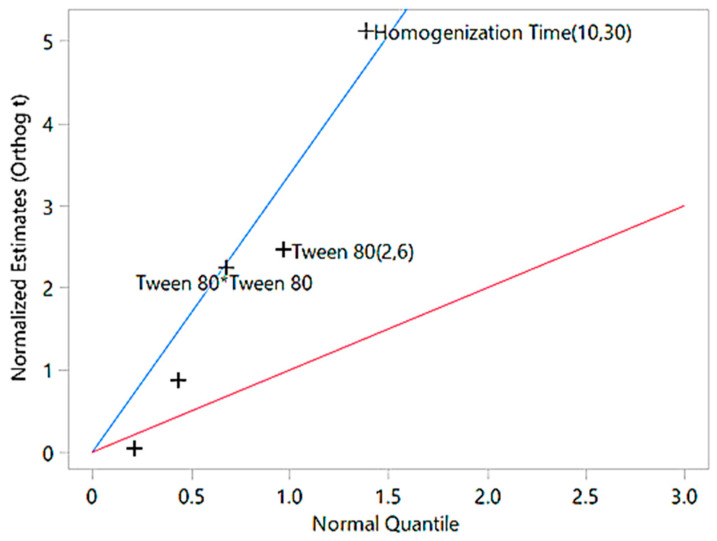
Half-normal plot of factor effects on particle size. The blue line represents Lenth’s pseudo-standard error (PSE) slope, used to assess factor significance, while the red line serves as a reference slope of 1. Factors further from the red line and closer to the blue line are more significant in influencing particle size.

The interaction effect between Tween 80 and homogenization time was also insignificant (*p* = 0.96), suggesting they work independently. Overall, the model provides a reliable predictive framework for optimizing particle size in nanoemulsions. The significance of the factors identified through ANOVA is further illustrated by the half-normal plot, which visually represents the impact of individual factors and their interactions on particle size (Figure 3).

The half-normal plot illustrates the impact of various factors on particle size. Factors like homogenization time (10,30) and Tween 80 (2,6), which are far above the blue line, are the most significant contributors to particle size reduction. The quadratic term of Tween 80 lies closer to the blue line, indicating a minor non-linear effect, while factors near or below the red line are statistically insignificant and have minimal influence. This emphasizes that the most impactful factors deviate significantly above the blue line, confirming their critical role in particle size optimization. The zeta potential values, as presented in Table 3, provide critical insight into the stability of the formulations. Zeta potential ranges from −16.9 mV to −31.5 mV, with higher absolute values indicating greater electrostatic repulsion and improved colloidal stability.

The highest absolute zeta potential (−31.5 mV) was observed at high levels of homogenization time (A). This may be attributed to enhanced adsorption and orientation of the surfactant (Tween 80) molecules at the oil–water interface due to higher shear forces during homogenization. The increased interfacial surface area created by smaller droplets allows for more uniform and dense surfactant coverage, leading to greater surface charge density and thus a more negative zeta potential, which in turn contributes to improved particle stability. Conversely, the least negative value of −16.9 mV was observed at low levels of both Tween 80 (-) and homogenization time (-), indicating reduced stability under these conditions. Intermediate values of zeta potential are noted when Tween 80 or homogenization time is varied individually, highlighting their individual contributions to colloidal stability. Overall, the results suggest that optimizing both factors can significantly enhance particle stability, as indicated by more negative zeta potential values.

**Figure 4:**
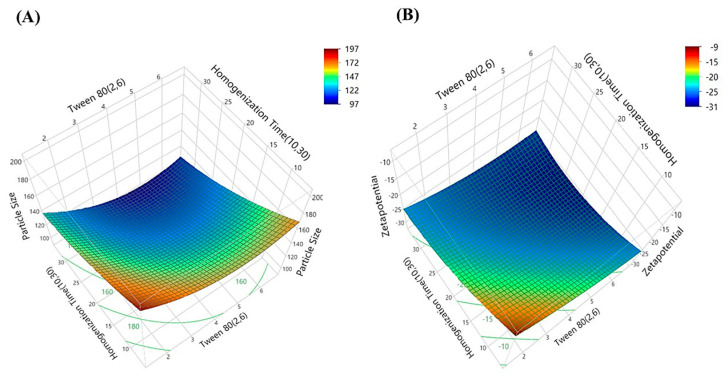
3D surface plots illustrating the interaction effects of Tween 80 concentration and homogenization time on (**A**) particle size and (**B**) zeta potential. These response surface plots highlight the trends in particle size reduction and charge stabilization, aiding in the identification of optimal formulation conditions.

The 3D surface plots for particle size and zeta potential (Figure 4) depict the interactive effects of Tween 80 concentration and homogenization time on these critical response parameters. In the particle size surface plot, a clear color gradient is observed—transitioning from red (indicating larger particles) to blue (indicating smaller particles). The blue zones highlight the region of minimum particle size, suggesting optimal formulation conditions for size reduction.

**Figure 5:**
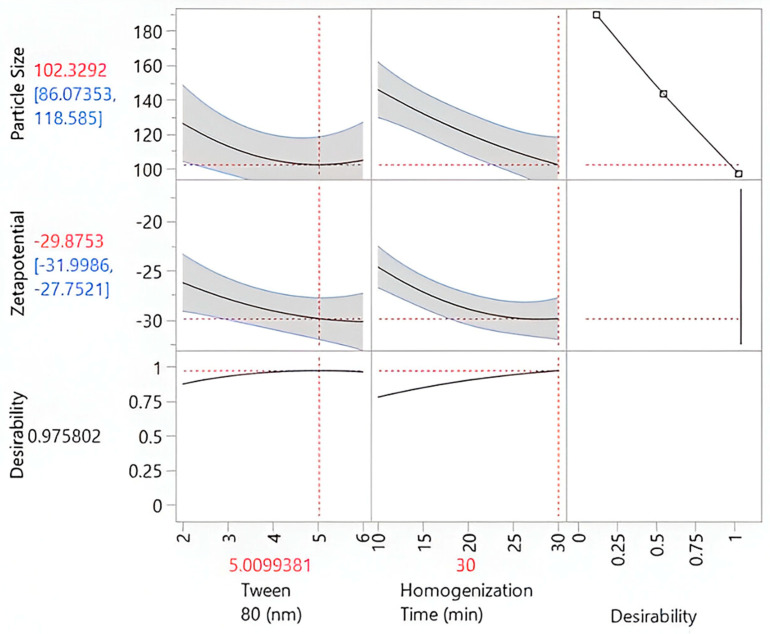
Prediction profiler illustrating the influence of Tween 80 concentration and homogenization time on particle size, zeta potential, and overall desirability. The plots display model-based predictions (black lines) with confidence intervals (blue lines) and indicate optimal parameter settings (red dashed lines) for achieving the desired formulation characteristics.

Similarly, the zeta potential plot shows a gradient from red (higher, less negative values) to blue (lower, more negative values), where more negative zeta potential (blue zones) signifies improved stability. The data points, represented as black markers, align closely with the surface in both plots, validating the predictive accuracy of the model. The particle size plot highlights conditions that minimize size, while the zeta potential plot emphasizes structural stability. Together, these plots underscore the significance of balancing Tween 80 and homogenization time to achieve optimal particle size and stability, with the alignment of experimental data reinforcing the reliability of the model. The optimal experimental conditions were identified using the prediction profiler (Figure 5), indicating a Tween 80 concentration of 5.01 and a homogenization time of 30 min. At these settings, the predicted particle size was 102.33 nm, with a confidence interval ranging from 86.07 nm to 118.58 nm, ensuring the reliability of the prediction. The zeta potential was predicted to be −29.88 mV, with a confidence interval of −31.99 mV to −27.75 mV, reflecting the stability of the colloidal system. The desirability score was 0.9758, highlighting the efficacy of the optimization approach in achieving the desired balance between particle size and zeta potential. These outputs confirm the robustness of the model and its ability to predict optimal conditions effectively.

### 2.4. Characterization Study of Kanuka-Loaded Nanoemulsions (K-NE)

To achieve a formulation with optimal characteristics, freshly prepared K-NEs were evaluated using various stability parameters. Over 60 days under different thermal conditions, the formulations exhibited no signs of physical instability, such as visible creaming, phase separation, or significant changes in color and transparency. Furthermore, the outcomes from heating–cooling and freeze–thaw cycles confirmed adequate thermodynamic stability, demonstrating the robustness of the Kanuka nanoemulsions under various conditions. Among the tested formulations, the one optimized through prediction profiles using CCD was selected for further studies, as it exhibited the best balance of stability and other desired characteristics.

### 2.5. Physicochemical Properties and Morphological Evaluation

**Figure 6:**
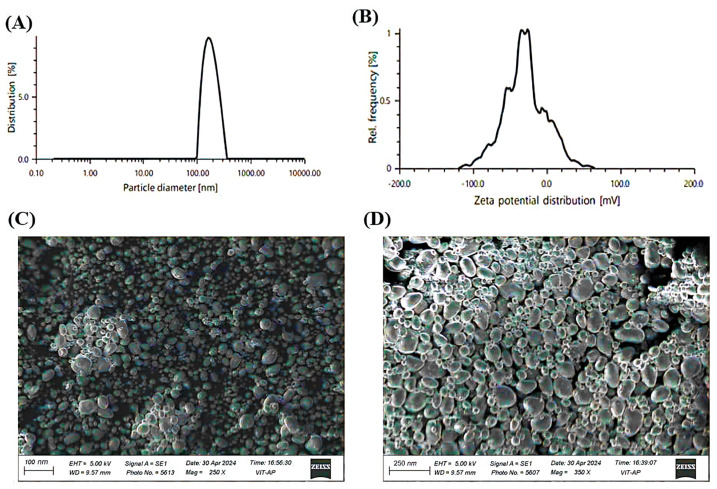
(**A**) Particle size distribution of the nanoemulsion, indicating uniformity and size range. (**B**) Zeta potential distribution, reflecting colloidal stability. (**C**) Scanning electron microscopy (SEM) image of the nanoemulsion at 250× magnification, showing surface morphology. (**D**) SEM image at 350× magnification, providing a detailed view of the structural characteristics.

The particle size analysis revealed an average diameter of 112.38 nm with a PDI of 0.203, indicating a consistent and well-distributed particle size with uniform dispersion. Zeta potential measurements recorded a value of −29.0 mV, suggesting good colloidal stability by preventing particle aggregation. Thermal stability assessments, including heating–cooling and freeze–thaw cycles, demonstrated stable thermodynamic behavior with no phase separation, ensuring formulation robustness. SEM analysis showed predominantly spherical morphology with a smooth surface texture, indicating uniform droplet formation. The absence of crystalline structures in the micrographs confirmed the amorphous nature of the formulation, which is crucial for enhancing solubility and bioavailability [5]. Furthermore, no visible aggregation or structural irregularities were observed, highlighting the excellent dispersion quality and physical stability of the nanoemulsion (Figure 6).

### 2.6. Entrapment Efficiency of Kanuka Nanoemulsion

The entrapment efficiency was assessed by UV-Vis spectrophotometry. For this, a calibration curve for Kanuka oil was constructed within the concentration range of 1 to 15 μg/mL, demonstrating excellent linearity with a high correlation coefficient (R^2^ = 0.998). The LOD and LOQ were determined to be 0.45 µg/mL and 1.42 µg/mL, respectively, indicating the sensitivity and precision of the method. The Kanuka content in the optimized nanoemulsion formulation was measured at 98.12 ± 0.42% on the day of formulation preparation. Following 60 days of storage, the Kanuka content decreased to 92.87 ± 1.36%, indicating a retention rate of 94.65%, suggesting good stability over time. A well-balanced emulsifier system minimizes droplet fusion, stabilizing the nanoemulsion and resulting in a higher entrapment efficiency (EE%). The optimized K-NEs formulation exhibited an EE% of 96.78 ± 0.55%, confirming effective encapsulation and long-term stability.

### 2.7. Development and Characterization of Kanuka Nanoemulgel (K-NG)

The optimized Kanuka Nanoemulsion (K-NE) was incorporated into varying concentrations of polyacrylic acid hydrogel to develop a stable nanoemulgel (K-NG) formulation. The hydrogel base was prepared by dispersing polyacrylic acid powder (commercially known as Carbopol 940, as specified in the Section 4.1) into distilled water with continuous stirring, followed by neutralization with triethanolamine until a clear gel was formed. Among the formulations tested, the hydrogel containing 1% *w*/*w* of polyacrylic acid (i.e., 1 g polymer in 100 g total gel) exhibited optimal physicochemical characteristics, including clarity, uniformity, consistency, and suitable adhesion strength, as evaluated using the thumb test over 30 days. Accelerated thermal stability testing confirmed that the K-NG remained physically stable, showing no signs of phase separation, aggregation, or syneresis. Formulations with higher polymer concentrations displayed enhanced viscosity, mucoadhesiveness, and stability during centrifugation but exhibited slightly reduced spreadability and ease of application [6]. The pH of the optimized K-NG was 5.74 ± 0.09 and remained stable with minimal variation throughout the 30-day observation period, supporting its suitability for topical use without risk of skin irritation. Overall, the K-NG exhibited desirable uniformity, physical appearance, spreadability, and thermodynamic stability as determined through visual inspection, consistency evaluation, glass plate method, and thermal cycling studies, making it a promising candidate for topical applications.

### 2.8. In Vitro Drug Release and Diffusion Kinetics

**Figure 7:**
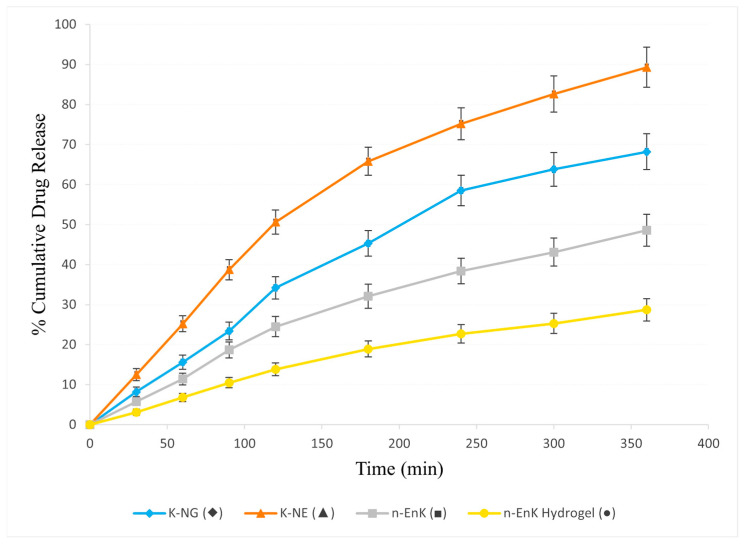
Cumulative drug release (%) of different formulations over time, presented as mean ± SD (n = 3). The release profiles of K-NG (♦), K-NE (▲), n-EnK (■), and n-EnK Hydrogel (●) illustrate differences in drug release kinetics among formulations. Error bars indicate standard deviation, highlighting variability in release behavior.

The in vitro drug release profile of K-NG was evaluated and compared with K-NEs, n-EnKan (non-encapsulated Kanuka oil) and n-EnKan hydrogel. The cumulative drug release data over 360 min (Figure 7) indicated that K-NEs exhibited the highest drug release (89.3%), followed by K-NG (68.2%), n-EnKan (48.6%), and n-EnKan hydrogel (28.7%). The drug release kinetics of Kanuka Nanoemulgel were analyzed using the Higuchi model, which describes drug diffusion from a matrix system [7]. A linear regression of cumulative drug release (Q) vs. sqrt t showed a high correlation (R^2^ = 0.989), with a Higuchi rate constant (kH) of 4.776. This indicates that the release mechanism is predominantly diffusion-controlled. Additionally, the Korsmeyer–Peppas model was applied, yielding an exponent (n = 0.88), suggesting an anomalous (non-Fickian) transport mechanism, where both diffusion and polymer relaxation (swelling/erosion) contribute to drug release [8]. Among the formulations, K-NG provided a sustained release profile, demonstrating its potential for prolonged therapeutic effects compared to K-NEs. The controlled release behavior is attributed to the gel matrix, which modulates the diffusion of the nanoemulsion, leading to a more extended release period [9].

### 2.9. Evaluation of Antibacterial Activity

The antimicrobial activity of K-NG was evaluated against *Staphylococcus aureus* and Escherichia coli using the broth dilution method. The minimum inhibitory concentration (MIC) values demonstrated that *S. aureus* exhibited greater susceptibility (MIC = 250 µg/mL) compared to *E. coli* (MIC = 500 µg/mL). This indicates a pronounced antibacterial effect, with a stronger inhibition against Gram-positive bacteria. While the MIC values of K-NG were higher compared to conventional antibiotics, Kanuka oil is well known for its favorable safety profile and has demonstrated antimicrobial efficacy even at relatively high doses without toxic effects [10]. Given its topical application, the formulation remains within safe and effective concentration limits, making it a promising candidate for reducing bacterial bio-burden on the skin [11]. This suggests potential benefits in managing dermatological conditions such as acne, burns, and wounds susceptible to bacterial contamination. Additionally, the combined antibacterial and anti-inflammatory properties of K-NG could aid in wound healing, further supporting its therapeutic potential in infection control and skin care.

### 2.10. Evaluation of Dermal Response in the Acute Irritation Study

The acute dermal irritation potential of the K-NG was evaluated based on OECD guideline 404 using three groups of male Wistar rats (n = 3 per group). The irritation response was assessed at 1, 24, 48, and 72 h post-application. The irritation scores, expressed as median ± interquartile range (IQR), are summarized in Table 5. At 1 h, the Kanuka nanoemulgel group exhibited mild redness (median score: 1.0 ± 0.5), which resolved completely within 24 h, with no further irritation observed at later time points. In contrast, the positive control (formalin) caused persistent erythema (median score: 2.0 ± 0.5) along with mild edema, which remained evident up to 72 h, though with a gradual reduction in severity. The negative control (blank gel) did not induce any dermal reactions throughout the study. Statistical analysis using the Kruskal–Wallis test confirmed a significant difference (*p* < 0.05) between groups at all time points, indicating that the Kanuka nanoemulgel is significantly less irritating than Formalin and comparable to the negative control. The results confirm the non-irritant nature of the Kanuka nanoemulgel, making it a promising candidate for dermatological applications.

### 2.11. In-Vivo Anti-Inflammatory Activity

**Figure 8:**
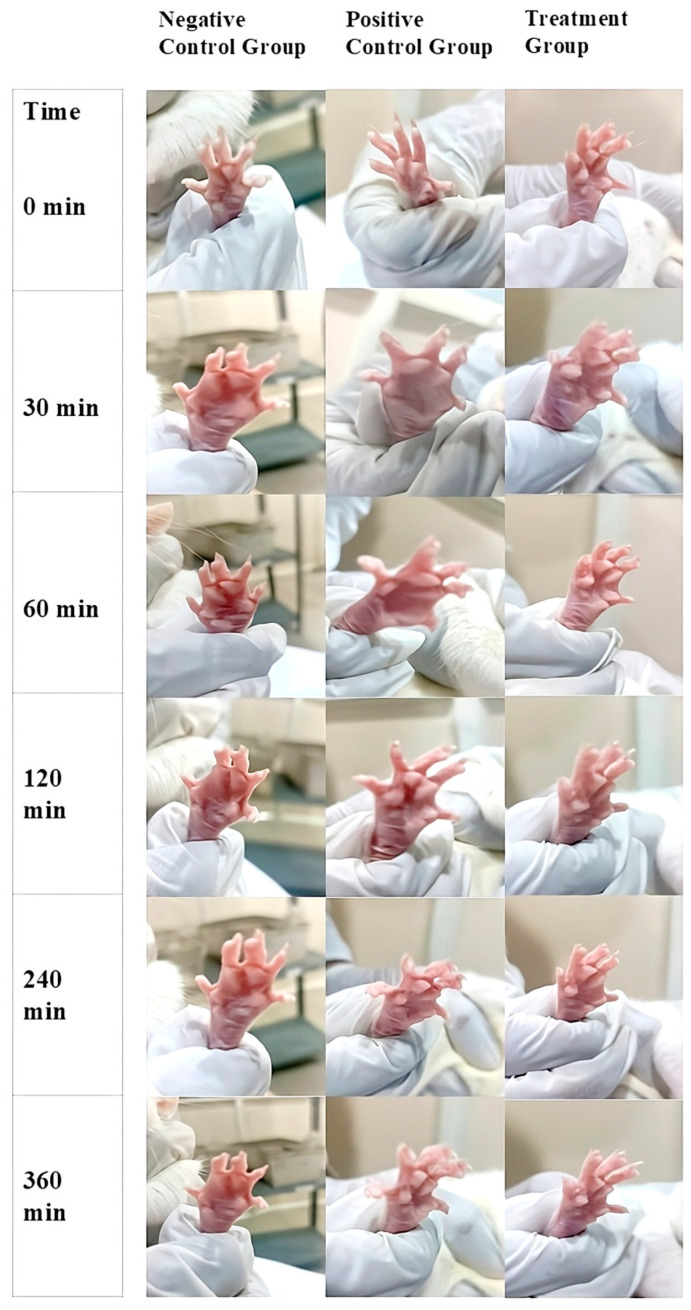
In vivo anti-inflammatory evaluation of K-NG using the carrageenan-induced paw edema model. Images show paw swelling in the negative control (blank nanoemulgel), positive control (Dicloran^®^ gel 2.5%), and treatment (K-NG) groups over time (0–360 min), highlighting K-NG’s edema-reducing effect.

Inflammation is a crucial defense mechanism of the immune system, protecting the host from various invading pathogens [12]. However, it often leads to distressing conditions such as redness, pain, and edema, significantly impacting the quality of life. Chronic inflammation is a primary reason for frequent physician visits, and while oral anti-inflammatory drugs are effective, their long-term use is associated with adverse effects [13]. To overcome these limitations, a topically applied transdermal formulation with enhanced patient compliance is desirable [14]. The in vivo anti-inflammatory activity of K-NG was evaluated using the carrageenan-induced hind paw edema method. The topical application of K-NG was compared with a negative control (blank nanoemulgel) and a positive control (Dicloran^®^ gel 2.5%). The progression of paw edema was recorded at 0, 30, 60, 120, 240, and 360 min (Figure 8), and the reduction in inflammation was quantified at regular intervals. Paw volume was measured in milliliters (mL), and inhibition of edema (%) was calculated. Statistical analysis was performed using one-way ANOVA followed by the Tukey-Kramer post hoc test, with significance set at *p* < 0.05. The results demonstrated a significant reduction in paw edema (*p* < 0.05) in the K-NG-treated group compared to the control (Table 6). At 30 min, the positive control (Dicloran^®^ gel) reduced paw edema volume to 0.30 ± 0.004 mL, while K-NG significantly decreased it further to 0.22 ± 0.003 mL. The inhibition of edema was 23.1% in the standard treatment group and 43.6% in the K-NG-treated group.

A statistically significant reduction was observed in the positive control compared to the control (*p* = 0.038). At 60 min, the positive control group showed 26.3% inhibition of edema, while K-NG demonstrated a highly significant reduction of 57.9% (*p* = 0.005). At 120 min, the K-NG-treated group exhibited a greater reduction in paw edema (0.08 ± 0.001 mL, 78.4% inhibition) compared to the standard (0.21 ± 0.002 mL, 43.2% inhibition), though the difference was not statistically significant (*p* = 0.089). By 240 min, the paw edema volume in the K-NG-treated group was 0.02 ± 0.001 mL, achieving 94.4% inhibition, while the positive control group had a volume of 0.07 ± 0.001 mL with 80.5% inhibition, demonstrating the potent anti-inflammatory effect of K-NG. By 360 min, both K-NG and Dicloran^®^ gel completely resolved inflammation (100% inhibition of edema). The results confirm the efficacy of K-NG in reducing inflammation, with comparable or superior effects to the standard treatment, suggesting its potential as an effective transdermal anti-inflammatory therapy.

## 3. Conclusions

This study successfully developed and optimized a Kunzea ericoides oil-loaded nanoemulgel (K-NG) using a Quality by Design (QdB) approach. The optimized formulation demonstrated desirable physicochemical characteristics, including a globule size of 98.3 nm, a PDI of 0.227, and a zeta potential of –28.6 mV, indicating good colloidal stability. In vitro drug release studies showed a sustained release of 68.2% over 360 min, following Higuchi kinetics (R^2^ = 0.989) and an anomalous diffusion mechanism (n = 0.88). The nanoemulgel exhibited significant anti-inflammatory activity in vivo, with 94.4% edema inhibition at 240 min and complete resolution at 360 min. Acute dermal irritation studies confirmed the formulation’s dermal safety, with no visible signs of irritation after 24 h. Antibacterial evaluation through MIC determination demonstrated effective activity against *Staphylococcus aureus* and *Escherichia coli*. Overall, the findings suggest that K-NG holds strong potential as a safe and effective transdermal therapy for managing inflammatory and dermatological conditions. Nonetheless, further studies are required with larger sample sizes and extended toxicity and pharmacokinetic evaluations to validate its long-term safety and commercial applicability.

## 4. Materials and Methods

### 4.1. Materials

Kanuka oil was acquired from Avi Naturals (Delhi, India). Surfactants, along with polyacrylic acid xerogel (Carbopol 940), Benzyl alcohol, and NaOH, were obtained through Loba Chemie Private Limited, Mumbai, India. *Staphylococcus aureus* Strain (737—MTCC), *Escherichia coli* (1035—MTCC) were acquired from the Microbial Type Culture Collection and gene bank situated in Chandigarh.

### 4.2. Screening of Surfactants and FT-IR Analysis of Kanuka Oil

A comprehensive screening process was conducted to assess the suitability of various surfactants for oil-in-water (O/W) nanoemulsions. The surfactants evaluated included Polysorbates (Tween 80, Tween 20), sorbitan esters (Span 80, Span 20), and polyethylene glycol (PEG 400) as excipients. Formulations were prepared with surfactant concentrations ranging from 1% to 20% *w*/*v*, maintaining the oil phase at 5% *w*/*v* for consistency. The screening process involved solubility studies, phase behavior analysis, and emulsification efficiency testing. Key parameters, including visual appearance, emulsion stability, and droplet size, were systematically analyzed to determine the optimal surfactant [15]. Tween 80 demonstrated superior emulsification capacity, producing stable, homogenous nanoemulsions with Kanuka oil, resulting in an optimized formulation with improved stability and reduced droplet size. Thus, Tween 80 was selected as the surfactant of choice for further formulation development.

FT-IR analysis of Kanuka oil was performed using an attenuated total reflectance (ATR) accessory (Lab India-Bruker). A small aliquot of the oil was applied directly onto the ATR crystal to ensure optimal contact. The spectrum was recorded over the wavenumber range of 4000–400 cm^−1^ with a resolution of 4 cm^−1^, averaging 32 scans to enhance signal accuracy. Background correction was conducted prior to sample analysis, and the acquired spectral data were examined for characteristic functional group assignments.

### 4.3. Gas Chromatography–Mass Spectrometry (GC–MS) Analysis

The chemical composition of Kanuka oil was analyzed using Gas Chromatography–Mass Spectrometry (GC–MS) with a Shimadzu GCMS-QP2010 instrument. A 1.00 µL sample of Kanuka oil was injected in split mode. The analysis was performed using a standard GC-MS method optimized for fatty acid and volatile compound profiling. Separation was carried out on a fused-silica capillary column with helium as the carrier gas. The column temperature was programmed to enable efficient volatilization and separation of essential oil constituents [16]. Mass spectral data were acquired in scan mode, and compound identification was achieved by matching the mass spectra against entries in the NIST107 library (National Institute of Standards and Technology). This procedure provided a comprehensive chemical fingerprint of Kanuka oil, supporting the identification of its potential therapeutic constituents.

### 4.4. Preparation and Optimization of Kanuka Nanoemulsion

Based on preliminary studies and preformulation analysis, the optimal concentration of Kanuka oil was determined to be 5% *w*/*v*. The nanoemulsion was prepared using the oil-in-water (O/W) emulsification technique. The aqueous phase, consisting of distilled water (89–93% *w*/*v*) and Tween 80 (2–6% *w*/*v*), was gradually added dropwise into the oil phase under continuous stirring using a Remi stirrer at 40 °C for 1 h to obtain a homogenous mixture [17]. The resulting pre-emulsion was then subjected to high-speed homogenization at 1400 rpm for 10–30 min to achieve a stable nanoemulsion.

To optimize the Kanuka oil nanoemulsion, a 2-factor, 3-level Central Composite Design (CCD) was constructed using JMP Statistical Software (version 17.0.0, JMP Statistical Discovery LLC, USA; accessed under a valid institutional student subscription license for academic use). Surfactant concentration (X_1_) and homogenization time (X2) were selected as independent variables, with levels ranging from 2% to 6% and 10 to 30 min, respectively. The mean droplet size (Y1) was minimized, while the zeta potential (Y2) was maintained within a specified range. These responses were analyzed to determine the optimal formulation conditions. A total of 13 experiments were conducted, including one replicate and five center points to ensure model reliability. The obtained results were analyzed using ANOVA at a 95% confidence interval (CI), with significance set at *p* ≤ 0.05. The formulation exhibiting the optimal response in terms of droplet size and zeta potential was selected for further characterization and evaluation.

### 4.5. Evaluation of Entrapment Efficiency

The Entrapment Efficiency (EE) of Kanuka-NEs (K-NEs) was assessed following previously established methodologies [18]. The calibration curve of the Kanuka extract was initially plotted at its λmax of 450 nm, with linearity confirmed utilizing a regression equation. Additionally, the Limit of Detection (LOD) and Limit of Quantification (LOQ) were evaluated [19]. In the experiment, 100 µL of K-NEs was diluted at a 1:100 ratio using an ethanol–water mixture in equal proportions (1:1 *v*/*v*). The Kanuka concentration (µg/mL) in each sample was determined using UV-visible spectroscopy. To eliminate interference from formulation components, a Kanuka-free formulation served as a blank. Each sample test was carried out in triplicate. The entrapment efficiency (EE%) was calculated using the following equation:

$$EE\%=\left(\frac{Wt}{Wi}\right)\times 100$$

where *Wt* is the amount of Kanuka oil entrapped in the nanoemulsion and *Wi* is the total amount of Kanuka oil initially used in the formulation. The entrapped amount (*Wt*) was determined by subtracting the free (unentrapped) Kanuka oil present in the supernatant from the total amount added.

### 4.6. Physicochemical Characterization and Stability Assessment

The prepared K-NEs were analyzed for zeta potential, particle size, and polydispersity index (PDI), utilizing the Malvern Zetasizer Nano ZS 90 (Mumbai, India), through photon correlation spectroscopy. Measurements were conducted at 25 °C in disposable polystyrene cells for size and PDI, while omega cuvettes were used for zeta potential analysis [20]. Stability assessments included thermal stress evaluations through heating–cooling and freeze–thaw cycles. In the heating–cooling cycle, the samples were subjected to alternating temperatures of 40 °C and 48 °C for 48-h intervals over three cycles, followed by examination for phase separation. The freeze–thaw cycle involved repeated freezing at −20 °C and thawing at 25 °C for three cycles, with subsequent centrifugation to detect instability [21]. Morphological characterization was conducted using Scanning Electron Microscopy (SEM), where samples were analyzed at various magnifications under an optimal accelerating voltage of 5 kV [22]. Automated image analysis techniques were utilized to evaluate the shape and surface characteristics of the dispersed phase. These evaluations provide critical insights into the physicochemical stability and structural integrity of K-NEs.

### 4.7. Preparation of Kanuka Loaded Nanoemulgel

Kanuka Nanoemulgel (K-NG) was prepared using polyacrylic acid xerogel 940 concentrations ranging from 0.5% to 2% *w*/*w*. The precise quantity of polyacrylic acid xerogel was blended using the ideal K-NEs under constant stirring. Benzyl alcohol was incorporated as a preservative, and the pH was adjusted using 1 M NaOH [23]. The formulation underwent physicochemical characterization, Parameters such as transparency, color stability, bubble formation, consistency, content leakage, and uniformity were assessed. Adhesion strength was determined using the Thumb Test, confirming the lack of visible droplets and verifying formulation integrity [24]. Spreadability was assessed using the glass plate method, where a Brookfield viscometer (ULA S00 spindle, 4 rpm, torque level 10) was used to determine viscosity. Additionally, the pH stability of the K-NG was analyzed to ensure long-term stability and skin compatibility.

### 4.8. In Vitro Drug Release Study and Kinetics

The in vitro release profile of the K-NG formulation was evaluated using a modified Franz diffusion cell method [25]. A cellophane membrane (12 kDa molecular weight cut-off) was pre-soaked in a 1:1 ethanol–water mixture overnight to ensure complete saturation. Subsequently, 1 g of K-NG was loaded into the donor compartment of the diffusion cell. Sampling was performed at fixed time intervals, and fresh medium was replenished in the acceptor compartment after each collection to maintain volume and diffusion conditions. The experiment was conducted under controlled conditions, with constant stirring at 200 rpm and temperature maintained at 32 °C [26]. After each sampling, the K-NG formulation was appropriately diluted, and the Kanuka oil concentration was determined using UV-visible spectroscopy at 450 nm, based on the established calibration curve. A Kanuka-free formulation was used as a blank to account for any potential interference from excipients.

In addition to the developed K-NG, the release profiles of its corresponding nanoemulsion (K-NEs), non-encapsulated Kanuka oil (n-EnKan) (Kanuka oil dissolved in ethanol–water), and n-EnKan hydrogel formulation (1% *w*/*w* Carbopol 940 incorporating non-encapsulated Kanuka oil) were also evaluated to provide a thorough comparison of the release behavior. The results were represented as the mean ± SD from three independent experiments. To analyze the diffusion characteristics and release kinetics of the optimized K-NG, various kinetic models, including zero-order, first-order, Hixson-Crowell, Higuchi, and Korsmeyer–Peppas, were applied [27]. The best-fit model was selected based on the highest R^2^ value, representing the most accurate depiction of the release profile.

### 4.9. Antimicrobial Activity of K-NG

The antibacterial efficacy of the K-NG formulation was evaluated against Staphylococcus aureus (737—MTCC) and Escherichia coli (1035—MTCC) using the broth microdilution method following Clinical and Laboratory Standards Institute (CLSI) guidelines. Two-fold serial dilutions of the formulation were prepared in Mueller–Hinton Broth (MHB) in a 96-well microplate, ranging from 500 µg/mL to 1.95 µg/mL. Fresh bacterial cultures were grown on a Nutrient Agar medium, and the inoculum was standardized to 0.5 McFarland turbidity (~1.5 × 10^8^ CFU/mL) using sterile saline [28]. The bacterial suspension was further diluted to achieve a final inoculum density of 5 × 10^5^ CFU/mL per well, and each well received 100 µL of bacterial suspension. The plates were incubated at 37 °C for 24 h under controlled conditions [29]. Bacterial growth was assessed by measuring optical density (OD600) using a microplate reader, with MIC defined as the lowest concentration showing no visible turbidity and OD ≤ 0.1. To ensure experimental reliability, appropriate controls were included: a sterility control containing only MHB without bacterial inoculum, a growth control with bacterial inoculum but no K-NG formulation, and an antibiotic control using Ciprofloxacin (2 µg/mL) for comparative efficacy. The experiment was conducted in triplicate, and MIC values were reported as mean ± standard deviation (SD) to ensure reproducibility.

### 4.10. In Vivo Study

This study was conducted on adult male Wistar rats weighing 250 ± 10 g. The animals were maintained under standard conditions, including a room temperature of 25 ± 2 °C, a 12-h light/12-h dark cycle, and free access to a standard laboratory chow diet [30]. All experimental procedures were conducted in strict compliance with the Institutional Animal Ethics Committee (IAEC) (Reg. No: 1048/PO/Re/S/07/CPCSEA). The study protocol, approved under certificate number 07/IAEC/CLPT/2023-24, adhered fully to the CPCSEA requirements.

### 4.11. Acute Dermal Irritation Study

The acute dermal irritation potential of the K-NG formulation was evaluated using healthy male Wistar rats in accordance with OECD guideline 404 [31]. A dose of 0.5 g of the K-NG was applied to a 6 cm^2^ shaved dorsal skin area under a semi-occlusive dressing for 4 h. The positive control (formalin, 0.8% solution) and negative control (blank formulation gel) were similarly tested. Following exposure, the sites were rinsed with lukewarm water, and dermal reactions were assessed at 1, 24, 48, and 72 h for erythema, edema, and other signs of irritation.

### 4.12. Evaluation of In Vivo Anti-Inflammatory Activity

The in vivo anti-inflammatory activity of K-NG was evaluated using the carrageenan-induced hind paw edema model in male Wistar rats (n = 6 per group) weighing 200–300 g. The animals were housed under controlled conditions (25 ± 2 °C, 12-h light/dark cycle) with free access to food and water [32]. The study was conducted following ethical approval from the IAEC. The animals were randomly divided into three groups: Group 1 (negative control) received blank nanoemulgel, Group 2 (positive control) was treated with Dicloran^®^ gel (2.5%), and Group 3 (treatment) received K-NG (5% *w*/*w* Kanuka oil nanoemulgel). Inflammation was induced by subplantar injection of 0.1 mL of 1% *w*/*v* carrageenan suspension into the right hind paw of all groups. Immediately after induction, 0.5 g of the respective formulations was topically applied to the affected area and gently massaged for uniform absorption. The application was performed once, and paw edema volume was measured at baseline (0 min) and at 30, 60, 120, 240, and 360 min using a digital vernier caliper by an observer blinded to the treatment groups. The percentage change in paw edema volume was calculated using the standard formula:

$$\%\;Change\;in\;Hind\;Paw\;Volume=\left(\frac{Mean\;C_{n}-Mean\;C_{i}}{Mean\;C_{i}}\right)\times 100$$

where Mean C_n_ represents the mean paw volume at a given time point, and Mean C_i_ represents the initial paw volume before inflammation induction. The inhibition of edema was further analyzed in comparison to the control group [33].

### 4.13. Statistical Analysis and Interpretation

Statistical analysis was performed using the Student’s *t*-test for pairwise comparisons and one-way ANOVA followed by the Tukey–Kramer post hoc test for multiple group comparisons. Results were expressed as mean ± standard deviation (SD) for in vitro studies and mean ± standard error of the mean (SEM) for in vivo experiments. A *p*-value of less than 0.05 was considered statistically significant to ensure the reliability and reproducibility of the findings.

## Figures and Tables

**Table 1:** FTIR absorption peaks of Kanuka oil and their functional group assignments.

Wavenumber (cm^−1^)	Functional Group	Accepted Range (cm^−1^)	Probable Compound Type
3738.27	O–H stretch	3200–3700	Phenols, Alcohols
2951.18, 2927.57, 2727.26	C–H stretch	2800–3000	Aliphatic chains (including alkanes)
1735.18, 1650.78	C=O stretch	1650–1750	Esters, Ketones
1448.81, 1322.20	C–H bending	1300–1500	Aliphatic hydrocarbons
1123.89, 1102.64	C–O stretch	1000–1300	Ethers, Alcohols
1027.69, 787.17, 882.51	C=C bending	650–1000	Terpenes, Phenolics
531.52, 449.52	Fingerprint region	<600	Specific spectral features

**Table 2:** GC-MS identification and % peak area contributions of major components in Kanuka oil.

Peak	RT (min)	Compound Name	Molecular Formula	Molecular Weight (g/mol)	Peak Area (%)
5	4.085	α-Pinene	C_10_H_16_	136.24	0.06%
6	4.361	(+)-2-Caren	C_10_H_16_	136.24	0.04%
12	5.158	Bicyclo[4.1.0]heptane, 7-(1-methylethylidene)	C_10_H_16_	136.24	9.31%
14	5.559	β-Pinene	C_10_H_16_	136.24	1.63%
16	5.811	β-Myrcene	C_10_H_16_	134.24	0.27%
22	6.439	Isolimonene	C_10_H_16_	136.24	9.76%
23	6.464	Eucalyptol	C_10_H_18_O	154.25	1.32%
32	7.834	Phenylethyl Alcohol	C_8_H_10_O	122.16	2.45%
36	8.195	Camphor	C_10_H_16_O	152.23	2.32%
41	8.956	(+)-α-Terpineol	C_10_H_18_O	154.25	6.32%
47	9.625	Bornyl acetate	C_12_H_20_O_2_	196.29	0.18%
49	9.736	Isopulegol acetate	C_12_H_18_O_2_	194.27	0.45%
69	12.445	α-Caryophyllene	C_15_H_24_	204.36	0.04%
82	13.896	Caryophyllene oxide	C_15_H_24_O	220.35	0.21%

**Table 3:** Experimental design matrix with actual and coded levels of variables, and observed particle size and zeta potential.

Formulations	X_1_ (Coded Level)	X_2_ (Coded Level)	Tween 80 (%) X_1_	Homogenization Time (min) X_2_	Particle Size (nm)	Zetapotential
1	0	0	4.0	20.0	136.67	−28.5
2	+	+	6.0	30.0	101.76	−29.9
3	−	−	2.0	10.0	184.90	−16.9
4	0	0	4.0	20.0	119.78	−29.1
5	a	0	1.2	20.0	145.87	−19.4
6	0	0	4.0	20.0	113.23	−24.7
7	−	+	2.0	30.0	132.50	−27.4
8	0	0	4.0	20.0	125.38	−27.6
9	0	A	4.0	34.1	100.65	−28.3
10	0	a	4.0	05.9	148.23	−19.5
11	0	0	4.0	20.0	118.34	−26.8
12	A	0	6.8	20.0	129.45	−31.5
13	+	−	6.0	10.0	155.40	−25.6

**Table 4:** ANOVA summary for model significance and statistical analysis.

Factor	df	Total Sum of Squares	Average Square	F-Ratio	Probability Value	Significance
Model	5	5462.94	1092.59	7.68	0.0092	Significant
Error	7	995.58	142.23	-	-	-
Total	12	6458.53	-	-	-	-
Intercept	-	-	122.68	23	<0.0001	Highly significant
Tween 80	1	-	−10.43	−2.47	0.0426	Significant
Homogenization Time	1	-	−21.67	−5.14	0.0013	Highly significant
Tween 80 × Homogenization Time	1	-	−0.31	−0.05	0.96	Not significant
Tween 80 × Tween 80	1	-	10.64	2.35	0.0509	Marginally significant
Homogenization Time × Time	1	-	4.03	0.89	0.4027	Not significant
R^2^	-	0.8462	-	-	-	Indicates strong model fit
Adjusted R^2^	-	0.7942	-	-	-	Adjusted for predictors

**Table 5:** Acute dermal irritation scores of different groups.

Time Point	Kanuka Nanoemulgel ^1^	Positive Control (Formalin) ^1^	Negative Control (Blank Gel) ^1^	*p*-Value ^2^
1 h	1.0 ± 0.5	2.0 ± 0.5	0.0 ± 0.0	0.0319
24 h	0.0 ± 0.0	2.0 ± 0.0	0.0 ± 0.0	0.0183
48 h	0.0 ± 0.0	2.0 ± 0.5	0.0 ± 0.0	0.0211
72 h	0.0 ± 0.0	1.0 ± 0.0	0.0 ± 0.0	0.0183

^1^ n = 3 (median ± IQR). ^2^ *p* < 0.05, significant difference between groups (Kruskal–Wallis test).

**Table 6:** In vivo anti-inflammatory assessment: edema inhibition and statistical comparisons.

Time (min)	Negative Control Group (mL) ^1^	Positive Control Group (mL) ^1^	Treatment Group (mL) ^1^	Inhibition of Edema (%)	Group Comparisons	Mean Difference (mL)	*p*-Value ^4^
0	0.00 ± 0.00	0.00 ± 0.00	0.00 ± 0.00	-	-	-	-
30	0.39 ± 0.005	0.30 ± 0.004	0.22 ± 0.003	23.1 **^2^**/ 43.6 **^3^**	Control vs. Standard	−0.9	0.038
60	0.38 ± 0.004	0.28 ± 0.003	0.16 ± 0.002	26.3 **^2^**/ 57.9 **^3^**	Control vs. Treatment	−2.2	0.005
120	0.37 ± 0.003	0.21 ± 0.002	0.08 ± 0.001	43.2 **^2^**/ 78.4 **^3^**	Standard vs. Treatment	−1.3	0.089
240	0.36 ± 0.002	0.07 ± 0.001	0.02 ± 0.001	80.5 **^2^**/ 94.4 **^3^**	-	-	-
360	0.35 ± 0.001	0.00 ± 0.00	0.00 ± 0.00	100 **^2,3^**	-	-	-

**^1^** Mean ± SEM (n = 6). **^2^** Inhibition percentage relative to the positive control group (Standard, S). **^3^** Inhibition percentage relative to treatment group (T). **^4^** Statistical significance set at *p* < 0.05.

## Data Availability

The data generated in this study can be requested from the corresponding author.

## Supplementary Materials

The following supporting information can be downloaded at: https://www.mdpi.com/article/10.3390/gels11060400/s1, File S1: Complete GC–MS Report of Kanuka Oil.

## Abbreviations

The following abbreviations are used in this manuscript:

QbD	Quality by Design
NE	Nanoemulsion
NEs	Nanoemulsions
K-NE	Kanuka Nanoemulsion
K-NEs	Kanuka Nanoemulsions
n-EnKan	non-encapsulated Kanuka oil
K-NG	Kanuka Nanoemulgel
CCD	Central Composite Design
ANOVA	Analysis of Variance
PDI	Polydispersity Index
CI	Confidence Interval
EE	Entrapment Efficiency
LOD	Limit of Detection
LOQ	Limit of Quantification
SEM	Scanning Electron Microscopy
UV	Ultraviolet
O/W	Oil-in-Water
MHB	Mueller–Hinton Broth
MIC	Minimum Inhibitory Concentration
CLSI	Clinical and Laboratory Standards Institute
CFU	Colony-Forming Unit
IAEC	Institutional Animal Ethics Committee
