# Supplementary material for: Development and Optimization of Kunzea ericoides Nanoemulgel Using a Quality by Design Approach for Transdermal Anti-Inflammatory Therapy
Source: Gels. 2025 May 27;11(6):400. doi: 10.3390/gels11060400 (PMC12192126; doi:10.3390/gels11060400)

CENTRAL ANALYTICAL FACILITY

Sample Information

Analyzed : 4/15/2025 11:45:18 AM

Sample Name : Kanuka Oil

Injection Volume : 1.00

Data File : E:\2025\April\15-GCMS\Kanuka Oil.qgd

Method File : C:\GCMSsolution\Data\Project1\GC_MS Fatty acid.qgm

Instrument Model :GCMSQP2010, SHIMADZU

Chromatogram Kanuka Oil

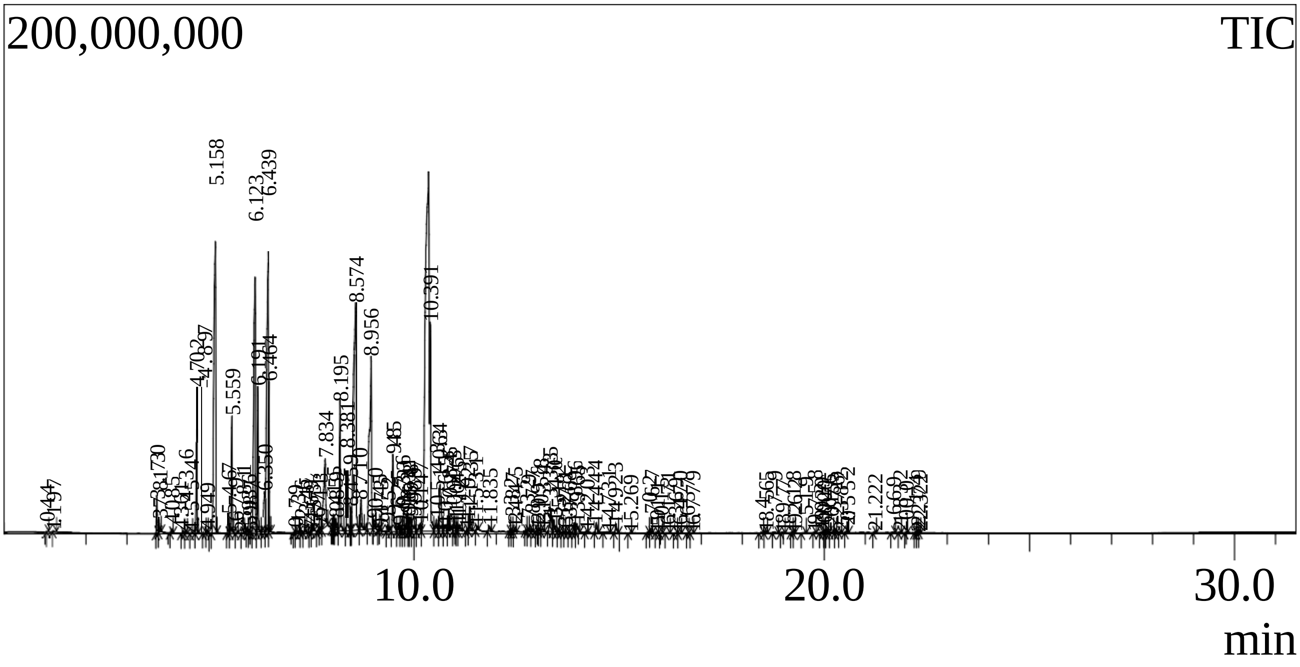

Peak Report TIC

Peak	R.Time	Area	Area%	Height	A/H	Base m/z	Name
1	1.044	213916	0.01	187123	1.14	44.10	Carbon dioxide
2	1.197	958598	0.03	538883	1.78	43.15	Acetone
3	3.730	21230182	0.57	14305265	1.48	41.15	3-Hexen-1-ol
4	3.781	7550290	0.20	4710840	1.60	41.15	3-Hexen-1-ol
5	4.085	2080926	0.06	1686117	1.23	93.20	.alpha.-Pinene
6	4.361	1349421	0.04	915864	1.47	93.20	(+)-2-Caren
7	4.436	1095320	0.03	838064	1.31	93.20	Bicyclo[2.2.1]hept-2-ene, 2,7,7-trimethyl-
8	4.554	230963	0.01	147052	1.57	55.15	4-Nonene, 5-methyl-
9	4.702	84603428	2.25	54991550	1.54	93.25	Cyclopentene, 4-ethenyl-1,5,5-trimethyl-
10	4.897	94166522	2.51	59579705	1.58	93.25	Bicyclo[3.1.1]hept-2-ene, 2,6,6-trimethyl-, (.+/-.)-
11	4.949	1137022	0.03	948868	1.20	79.20	Bicyclo[3.3.0]oct-2-en-7-one, 6-methyl-

Pea	R.Time	Area	Area%	Height	A/H	Base m/z	Name
12	5.158	349377129	9.31	109906841	3.18	121.25	Bicyclo[4.1.0]heptane, 7-(1-methylethylidene)-
13	5.467	11018995	0.29	7583077	1.45	119.25	1,3,5-Cycloheptatriene, 3,7,7-trimethyl-
14	5.559	61119871	1.63	44127713	1.39	93.20	.beta.-Pinene
15	5.679	508715	0.01	283588	1.79	95.20	3-Menthene
16	5.811	10094952	0.27	6222951	1.62	41.15	.beta.-Myrcene
17	5.936	1818031	0.05	1430316	1.27	93.20	Cyclohexene, 4-methyl-3-(1-methylethylidene)-
18	5.987	275832	0.01	224437	1.23	91.20	Dispiro[2.0.2.5]undecane, 8-methylene-
19	6.123	251971902	6.72	96551589	2.61	93.15	3-Octen-5-yne, 2,7-dimethyl-, (Z)-
20	6.191	83660451	2.23	55221269	1.52	43.15	7-Oxabicyclo[2.2.1]heptane, 1-methyl-4-(1-methylethyl)-
21	6.350	38992274	1.04	15245224	2.56	119.25	Benzene, 1-methyl-2-(1-methylethyl)-
22	6.439	366354103	9.76	105840969	3.46	67.15	Isolimonene
23	6.464	49715204	1.32	56815702	0.88	43.10	Eucalyptol
24	7.079	223320	0.01	158689	1.41	79.15	6-Propenylbicyclo[3.1.0]hexan-2-one
25	7.136	1002618	0.03	360332	2.78	43.10	Propanedioic acid, diethyl ester
26	7.246	946619	0.03	479415	1.97	132.30	Benzene, 1-methyl-4-(1-methylethenyl)-
27	7.347	1872808	0.05	934590	2.00	117.20	Benzene, 1-methyl-4-(1-methylethenyl)-
28	7.469	939669	0.03	704182	1.33	108.25	Bicyclo[2.2.1]heptane-2,5-diol, 1,7,7-trimethyl-, (2-endo,5-exo)-
29	7.534	4755648	0.13	2250902	2.11	119.25	(+)-3-Carene, 2-(acetylmethyl)-
30	7.671	1771979	0.05	1137575	1.56	139.30	2H-Pyran, tetrahydro-4-methyl-2-(2-methyl-1-propenyl)-
31	7.713	1690162	0.05	1084714	1.56	81.20	Bicyclo[2.2.1]heptan-2-ol, 1,3,3-trimethyl-
32	7.834	92032890	2.45	25785278	3.57	91.20	Phenylethyl Alcohol
33	8.015	6918735	0.18	4298693	1.61	67.15	Cyclohexanone, 2-(2-methylpropylidene)-
34	8.045	6524093	0.17	4094525	1.59	43.15	2,4,6-Trimethyl-3-cyclohexen-1-carboxaldehyde
35	8.089	8954295	0.24	4273652	2.10	67.15	1-Decalone (cis-trans)
36	8.195	87124164	2.32	49507183	1.76	95.20	Camphor
37	8.381	98740249	2.63	30887200	3.20	95.20	Bicyclo[2.2.1]heptan-2-ol, 1,7,7-trimethyl-, formate, endo-
38	8.459	16352144	0.44	8814730	1.86	87.15	Ethyl acetoacetate ethylene acetal
39	8.574	364749811	9.72	85940164	4.24	108.15	Benzylcarbamate
40	8.710	26754316	0.71	11550611	2.32	91.15	Bicyclo[3.1.0]hexan-2-ol, 2-methyl-5-(1-methylethyl)-, (1.alpha.,2.beta.,
41	8.956	237198486	6.32	65175101	3.64	59.15	(+)-.alpha.-Terpineol (p-menth-1-en-8-ol)
42	9.030	7585106	0.20	3493275	2.17	121.25	Bicyclo[3.1.0]hexane, 6-isopropylidene-1-methyl-
43	9.145	524778	0.01	383948	1.37	119.25	(E)-3-Caren-2-ol
44	9.173	324810	0.01	299713	1.08	43.10	2-Oxabicyclo[2.2.2]octan-6-ol, 1,3,3-trimethyl-
45	9.352	5144618	0.14	2454786	2.10	91.15	2-Cyclohexen-1-ol, 2-methyl-5-(1-methylethenyl)-, cis-
46	9.485	58662087	1.56	29273323	2.00	41.15	6-Octen-1-ol, 3,7-dimethyl-
47	9.625	6602529	0.18	2790007	2.37	43.15	Bornyl acetate
48	9.683	4234332	0.11	2022807	2.09	82.15	2-Cyclohexen-1-one, 2-methyl-5-(1-methylethenyl)-
49	9.736	16810407	0.45	9483185	1.77	80.15	Isopulegol acetate
50	9.831	18077864	0.48	7312608	2.47	108.25	3-Cyclohexene-1-methanol, 6-methyl-
51	9.864	8560526	0.23	5259434	1.63	41.15	2,6-Octadien-1-ol, 3,7-dimethyl-, (Z)-
52	9.898	12292709	0.33	5246780	2.34	91.15	Propanoic acid, phenylmethyl ester
53	9.989	22872921	0.61	5996238	3.81	95.20	Isopulegol acetate
54	10.147	9083970	0.24	2936827	3.09	41.15	6-Octen-1-ol, 3,7-dimethyl-
55	10.391	953685779	25.42	78979997	12.08	57.15	4-tert-Butylcyclohexyl acetate
56	10.543	14500243	0.39	4450569	3.26	43.10	Bicyclo[3.1.1]hept-3-en-2-ol, 4,6,6-trimethyl-, (1.alpha.,2.alpha.,5.alpha
57	10.634	47077705	1.25	17679549	2.66	57.15	4-tert-Butylcyclohexyl acetate
58	10.765	8320174	0.22	2229432	3.73	43.10	1,6-Octadien-3-ol, 3,7-dimethyl-, 2-aminobenzoate
59	10.845	13039431	0.35	8269710	1.58	43.10	Isopulegol acetate
60	10.973	11830181	0.32	5764537	2.05	93.20	3,5-Heptadienal, 2-ethylidene-6-methyl-
61	11.046	6087114	0.16	3320725	1.83	91.20	2-Cyclohexen-1-ol, 2-methyl-5-(1-methylethenyl)-
62	11.125	5726985	0.15	2004650	2.86	41.10	cis-2,6-Dimethyl-2,6-octadiene
63	11.292	1147276	0.03	737363	1.56	91.15	D-Verbenone
64	11.357	14655620	0.39	9009411	1.63	91.20	2-Cyclohexen-1-ol, 2-methyl-5-(1-methylethenyl)-
65	11.531	1960603	0.05	1337017	1.47	93.20	Butanoic acid, 3,7-dimethyl-2,6-octadienyl ester, (E)-
66	11.835	4086013	0.11	1890472	2.16	43.10	1H-Benzocycloheptene, 2,4a,5,6,7,8,9,9a-octahydro-3,5,5-trimethyl-9-m
67	12.332	463036	0.01	339004	1.37	119.25	Dihydrocurcumene
68	12.387	541611	0.01	395032	1.37	91.15	1R,3Z,9s-4,11,11-Trimethyl-8-methylenebicyclo[7.2.0]undec-3-ene

69	12.445	1495672	0.04	1006433	1.49	93.20	.alpha.-Caryophyllene
70	12.732	742208	0.02	494952	1.50	161.30	Isoledene
71	12.779	981397	0.03	616020	1.59	135.25	.alpha. Isomethyl ionone
72	12.877	1866380	0.05	1192414	1.57	41.10	Eudesma-4(14),11-diene
73	12.998	1784018	0.05	960792	1.86	93.20	Naphthalene, 1,2,3,4,4a,5,6,8a-octahydro-4a,8-dimethyl-2-(1-methyleth
74	13.058	1431488	0.04	967111	1.48	105.20	Naphthalene, 1,2,4a,5,6,8a-hexahydro-4,7-dimethyl-1-(1-methylethyl)-
75	13.134	2452089	0.07	829208	2.96	43.10	5-Isopropenyl-1,2-dimethylcyclohex-2-enol
76	13.335	23545563	0.63	8680078	2.71	66.15	Indan-1,3-diol monopropionate
77	13.406	10489087	0.28	4530933	2.31	159.30	Naphthalene, 1,2,3,4-tetrahydro-1,6-dimethyl-4-(1-methylethyl)-, (1S-ci
78	13.512	529878	0.01	308009	1.72	57.10	Indan-1,3-diol monopropionate
79	13.628	96623	0.00	59615	1.62	105.20	Naphthalene, 1,2,4a,5,6,8a-hexahydro-4,7-dimethyl-1-(1-methylethyl)-,[
80	13.671	152788	0.00	113351	1.35	57.15	Tricyclo[3.3.1.1(3,7)]decane-2,4-diol
81	13.769	270590	0.01	182335	1.48	82.20	1H-3a,7-Methanoazulene, octahydro-3,6,8,8-tetramethyl-, [3R-(3.alpha.
82	13.896	7797294	0.21	3582779	2.18	41.15	Caryophyllene oxide

Pea	R.Time	Area	Area%	Height	A/H	Base m/z	Name
83	13.965	10165825	0.27	3399536	2.99	43.10	Rose acetate
84	14.203	1449427	0.04	646982	2.24	111.25	1-Phenanthrenemethanol, 1,2,3,4,4a,5,6,9,10,10a-decahydro-1,4a-dimet
85	14.444	8955124	0.24	3804388	2.35	41.15	Caryophyllene oxide
86	14.731	1917885	0.05	321118	5.97	149.20	5-Bromoadamantan-2-one
87	14.923	8182653	0.22	3311044	2.47	41.15	Caryophyllene oxide
88	15.269	265768	0.01	132842	2.00	81.15	cis-Z-.alpha.-Bisabolene epoxide
89	15.702	662374	0.02	326491	2.03	83.15	Methyl dihydrojasmonate
90	15.767	339785	0.01	199108	1.71	157.25	Cadala-1(10),3,8-triene
91	15.911	446149	0.01	216039	2.07	157.25	Cadala-1(10),3,8-triene
92	16.015	237827	0.01	116693	2.04	183.25	Azulene, 1,4-dimethyl-7-(1-methylethyl)-
93	16.171	592125	0.02	143612	4.12	55.10	Cyclopentaneacetaldehyde, 2-formyl-3-methyl-.alpha.-methylene-
94	16.369	257717	0.01	124862	2.06	55.15	Bicyclo[3.1.1]heptan-3-ol, 6,6-dimethyl-2-methylene-
95	16.470	245965	0.01	107479	2.29	41.15	1H-Indole-2-carboxylic acid, 6-hydroxy-, ethyl ester
96	16.657	79975	0.00	46817	1.71	43.10	Ergostane-3,5,6,12,25-pentol, 25-acetate, (3.beta.,5.alpha.,6.beta.,12.bet
97	16.779	232304	0.01	134597	1.73	43.10	1H-Cycloprop[e]azulen-4-ol, decahydro-1,1,4,7-tetramethyl-, [1ar-(1a.al
98	18.465	278850	0.01	138269	2.02	243.30	Cyclopenta[g]-2-benzopyran, 1,3,4,6,7,8-hexahydro-4,6,6,7,8,8-hexame
99	18.565	1092736	0.03	519800	2.10	133.25	1,3-Cyclopentadiene, 5-(1,3-dimethylbutylidene)-
100	18.779	1274888	0.03	434047	2.94	105.20	Phenylethylacetal
101	18.977	772930	0.02	339479	2.28	135.25	1-(2,4-Dimethylphenyl)ethanol
102	19.218	345698	0.01	217224	1.59	135.25	Benzenemethanol, 4-(1-methylethyl)-
103	19.262	137355	0.00	95194	1.44	133.25	Spiro[androst-5-ene-17,1'-cyclobutan]-2'-one, 3-hydroxy-, (3.beta.,17.be
104	19.519	606979	0.02	170590	3.56	41.15	Bicyclo[3.1.1]hept-2-ene, 2,2'-(1,2-ethanediyl)bis[6,6-dimethyl-
105	19.758	379034	0.01	193802	1.96	105.20	Bicyclo[4.1.0]hept-3-ene, 7,7-dimethyl-3-vinyl-
106	19.929	438918	0.01	151759	2.89	91.15	2-Cyclohexen-1-ol, 2-methyl-5-(1-methylethenyl)-
107	20.000	128357	0.00	89804	1.43	105.15	Bicyclo[4.1.0]hept-3-ene, 7,7-dimethyl-3-vinyl-
108	20.076	364177	0.01	255967	1.42	241.35	.alpha.-Cedrene, 2-bromo-
109	20.155	725562	0.02	387797	1.87	135.20	Acetic acid, 3-cyclohex-1-enyl-1-methylprop-2-ynyl ester
110	20.276	191575	0.01	120694	1.59	283.35	2(1H)-Phenanthrenone, 4a,9,10,10a-tetrahydro-6-hydroxy-1,1,4a-trimet
111	20.382	742775	0.02	423775	1.75	135.25	Acetic acid, 3-cyclohex-1-enyl-1-methylprop-2-ynyl ester
112	20.552	2145941	0.06	1287980	1.67	135.25	2-Isopropylidene-3-methylhexa-3,5-dienal
113	21.222	846732	0.02	454734	1.86	141.20	3-Heptyne-2,5-diol, 6-methyl-5-(1-methylethyl)-
114	21.669	496336	0.01	172966	2.87	105.15	7-Methyl-1,2,3,5,8,8a-hexahydronaphthalene
115	21.832	501654	0.01	262909	1.91	267.35	Ether, bis(p-tert-butylphenyl)
116	22.000	224250	0.01	158398	1.42	43.10	Pregnan-20-one, 3,11-dihydroxy-, (3.beta.,5.beta.,11.alpha.)-
117	22.214	170459	0.00	106426	1.60	223.30	4,4'-Diisopropylbiphenyl
118	22.279	165476	0.00	115272	1.44	43.10	Anthiaergosta-5,7,9-trien-3-one
119	22.322	566813	0.02	350151	1.62	283.35	2(1H)-Phenanthrenone, 4a,9,10,10a-tetrahydro-6-hydroxy-1,1,4a-trimet
		3752237954	100.00	1192103617			

Library

<< Target >>
Line#:1 R.Time:1.045(Scan#:210) MassPeaks:6
RawMode:Averaged 1.040-1.050(209-211) BasePeak:44.10(154277)
BG Mode:Calc. from Peak

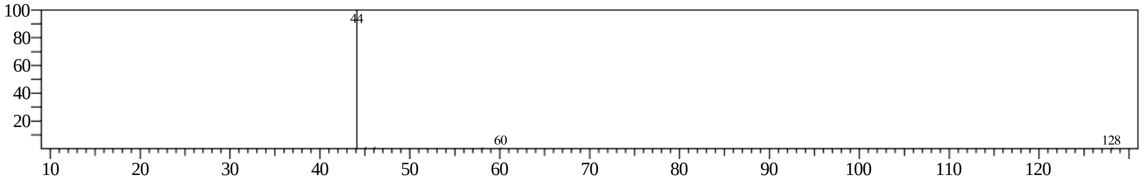

Hit#:1 Entry:36 Library:NIST107.LIB
SI:99 Formula:CO2 CAS:124-38-9 MolWeight:44 RetIndex:0
CompName:Carbon dioxide $$ Carbon oxide (CO2) $$ Carbonic acid, gas $$ Carbonic anhydride $$ Dry ice $$ CO2 $$ Anhydride carbonique $$ Carbonic

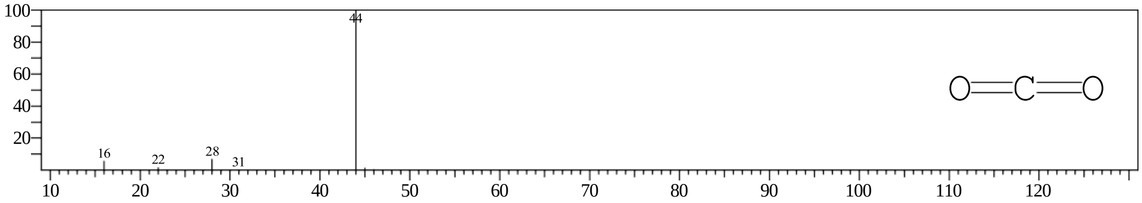

Hit#:2 Entry:41 Library:NIST107.LIB
SI:99 Formula:N2O CAS:10024-97-2 MolWeight:44 RetIndex:0
CompName:Nitrous Oxide $$ Nitrogen oxide (N2O) $$ Dinitrogen monoxide $$ Dinitrogen oxide $$ Laughing gas $$ N2O $$ Factitious air $$ Hyponitrou

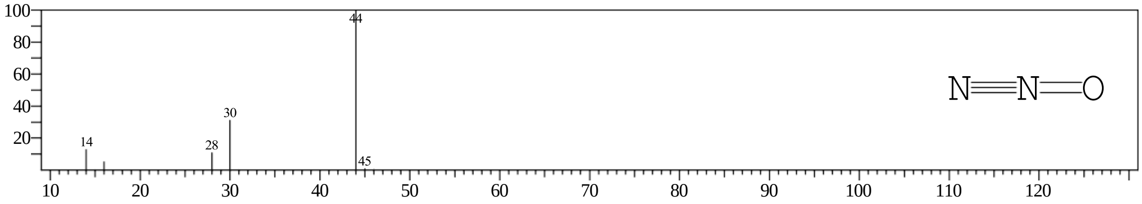

Hit#:3 Entry:440 Library:NIST107.LIB
SI:99 Formula:CH6N2O2 CAS:1111-78-0 MolWeight:78 RetIndex:0
CompName:Carbamic acid, monoammonium salt $$ Ammonium carbamate $$ Carbamic acid, ammonium salt $$ Ammonium carbaminate $$

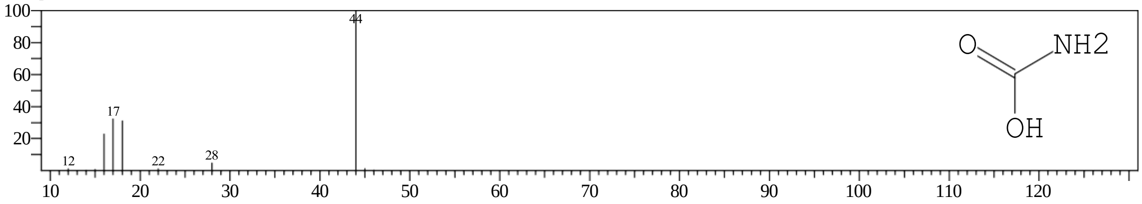

Hit#:4 Entry:37 Library:NIST107.LIB
SI:98 Formula:C2HF CAS:2713-09-9 MolWeight:44 RetIndex:0
CompName:Ethyne, fluoro- $$ Acetylene, fluoro- $$ Fluoroacetylene $$ Monofluoroacetylene $$ HC.$.CF $$ Fluoroethyne $$ C2HF $$

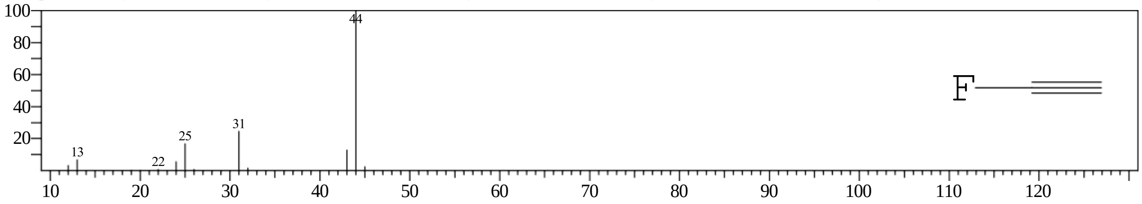

Hit#:5 Entry:39 Library:NIST107.LIB
SI:98 Formula:C2H4O CAS:75-21-8 MolWeight:44 RetIndex:0
CompName:Ethylene oxide $$ Oxirane $$ Dihydrooxirene $$ Dimethylene oxide $$ Epoxyethane $$ Ethene oxide $$ ETO $$ Oxacyclopropane $$ Oxane

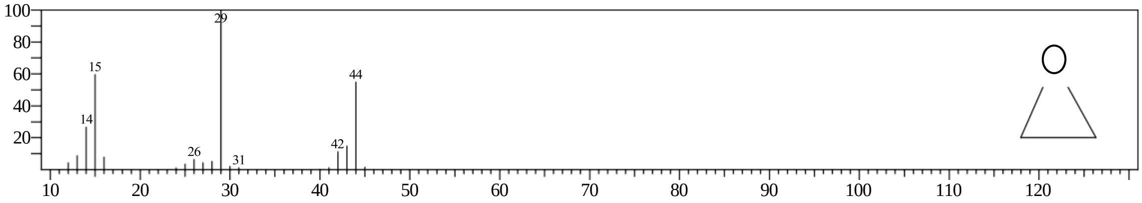

<< Target >>
Line#:2 R.Time:1.195(Scan#:240) MassPeaks:15
RawMode:Averaged 1.190-1.200(239-241) BasePeak:43.15(291849)
BG Mode:Calc. from Peak

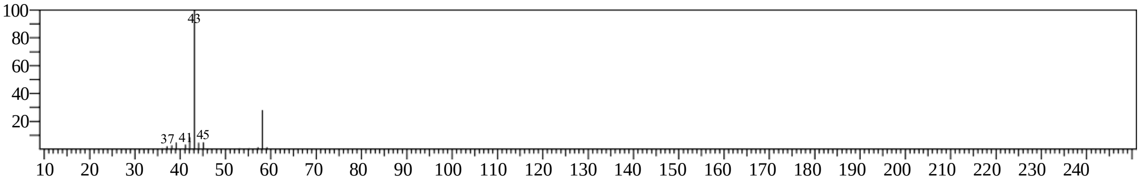

Hit#:1 Entry:103 Library:NIST107.LIB
SI:95 Formula:C3H6O CAS:67-64-1 MolWeight:58 RetIndex:0
CompName:Acetone $$ 2-Propanone $$ .beta.-Ketopropane $$ Dimethyl ketone $$ Dimethylformaldehyde $$ Methyl ketone $$ Propanone $$ Pyroacetic e

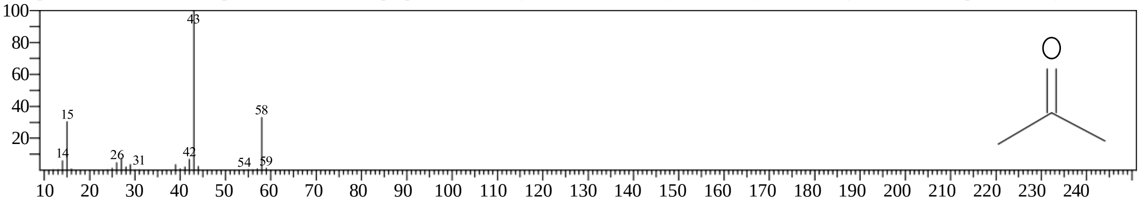

Hit#:2 Entry:709 Library:NIST107.LIB
SI:94 Formula:C4H7NO CAS:75-86-5 MolWeight:85 RetIndex:0
CompName:Propanenitrile, 2-hydroxy-2-methyl- $$ Lactonitrile, 2-methyl- $$ .alpha.-Hydroxyisobutyronitrile $$ Acetone cyanohydrin $$ 2-Cyano-2-propa

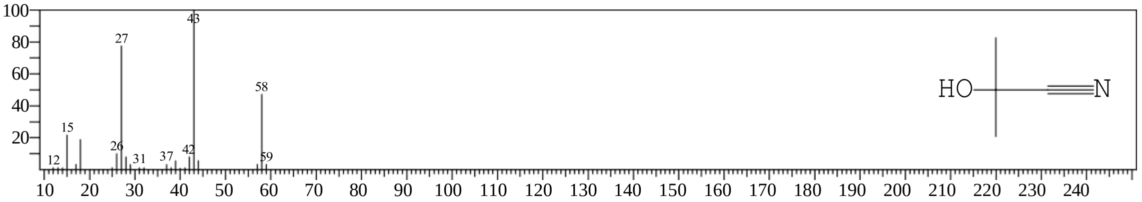

Hit#:3 Entry:73329 Library:NIST107.LIB
SI:90 Formula:C16H18O6 CAS:0-00-0 MolWeight:306 RetIndex:0
CompName:2-Benzofurancarboxylic acid, 7-methoxy-, (3,4,4-trimethyl-1,2-dioxetan-3-yl)methyl ester $$

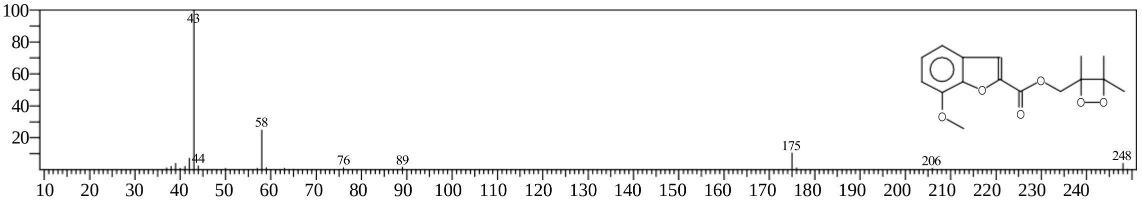

Hit#:4 Entry:2028 Library:NIST107.LIB
SI:90 Formula:C4H6O3 CAS:108-24-7 MolWeight:102 RetIndex:0
CompName:Acetic acid, anhydride $$ Acetic anhydride $$ Acetic oxide $$ Acetyl anhydride $$ Acetyl ether $$ Acetyl oxide $$ Ethanoic anhydride $$ Hyd

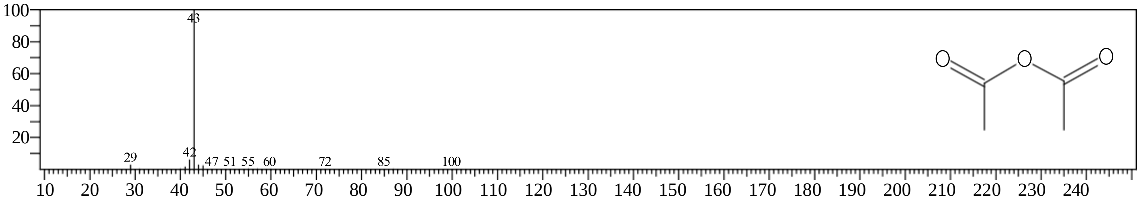

Hit#:5 Entry:1778 Library:NIST107.LIB
SI:90 Formula:C5H8O2 CAS:108-22-5 MolWeight:100 RetIndex:0
CompName:1-Propen-2-ol, acetate $$ Isopropenyl acetate $$ Propen-2-yl acetate $$ 2-Acetoxypropene $$ 2-Acetoxypropylene $$ Acetic acid 1-methylethe

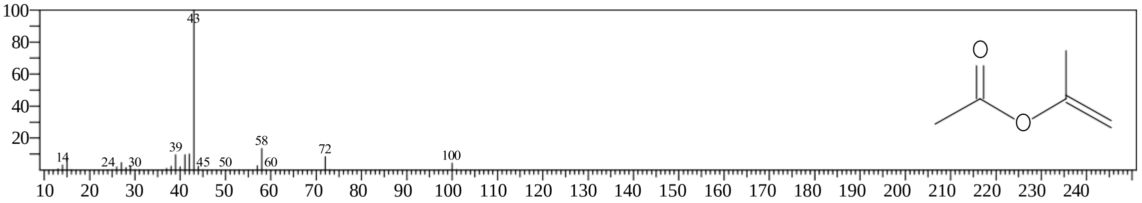

<< Target >>
Line#:3 R.Time:3.730(Scan#:747) MassPeaks:54
RawMode:Averaged 3.725-3.735(746-748) BasePeak:41.15(3212318)
BG Mode:Calc. from Peak

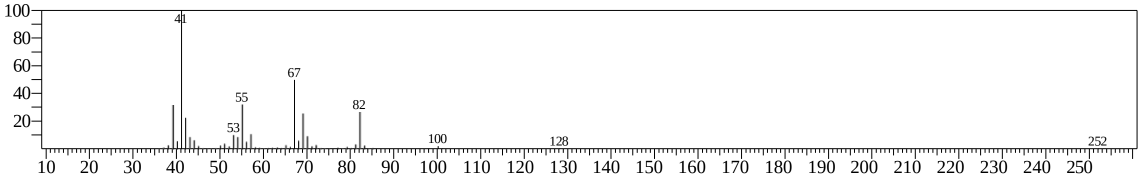

Hit#:1 Entry:1857 Library:NIST107.LIB
SI:96 Formula:C6H12O CAS:544-12-7 MolWeight:100 RetIndex:0
CompName:3-Hexen-1-ol $$ 3-Hexen-1-ol (c,t) $$

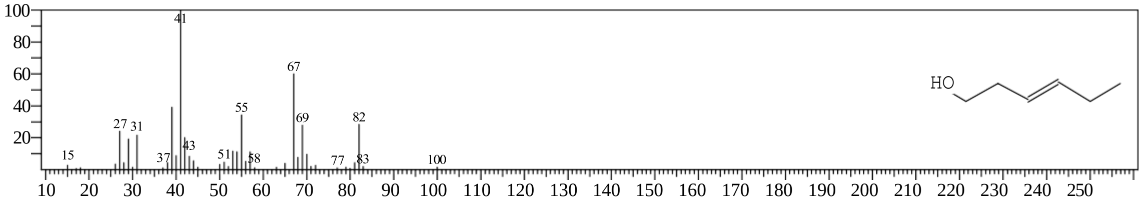

Hit#:2 Entry:1891 Library:NIST107.LIB
SI:96 Formula:C6H12O CAS:928-97-2 MolWeight:100 RetIndex:0
CompName:3-Hexen-1-ol, (E)- $$ trans-3-Hexen-1-ol $$ trans-3-Hexenol $$ E-3-Hexenol $$ (E)-Hex-3-en-1-ol $$

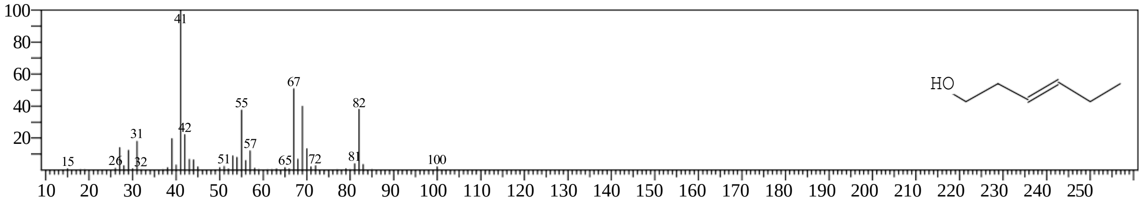

Hit#:3 Entry:1910 Library:NIST107.LIB
SI:91 Formula:C6H12O CAS:5673-98-3 MolWeight:100 RetIndex:0
CompName:4-Penten-1-ol, 2-methyl- $$

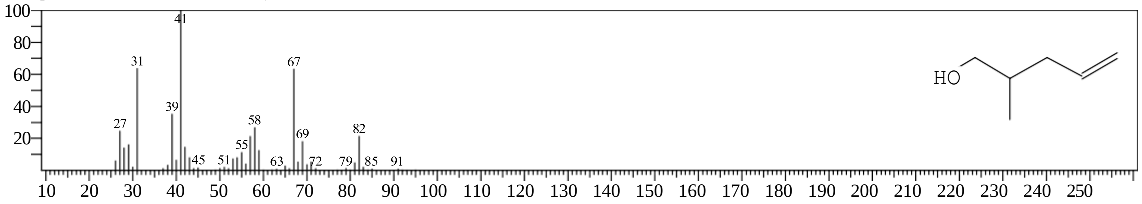

Hit#:4 Entry:1884 Library:NIST107.LIB
SI:90 Formula:C6H12O CAS:928-96-1 MolWeight:100 RetIndex:0
CompName:3-Hexen-1-ol, (Z)- $$ (Z)-Hex-3-en-1-ol $$ cis-3-Hexen-1-ol $$ cis-3-Hexene-1-ol $$ cis-3-Hexenol $$ Blatteralkohol (German) $$ Leaf alcoh

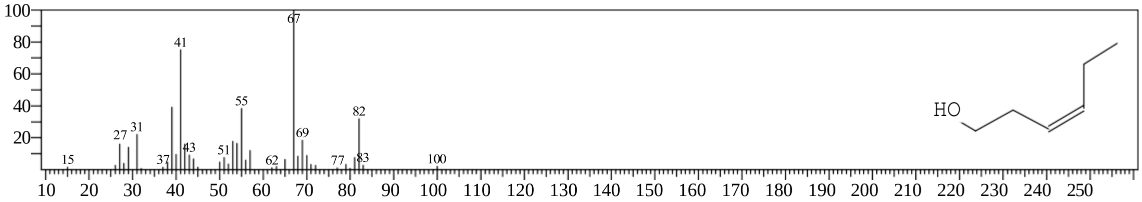

Hit#:5 Entry:1873 Library:NIST107.LIB
SI:89 Formula:C6H12O CAS:6126-50-7 MolWeight:100 RetIndex:0
CompName:4-Hexen-1-ol $$

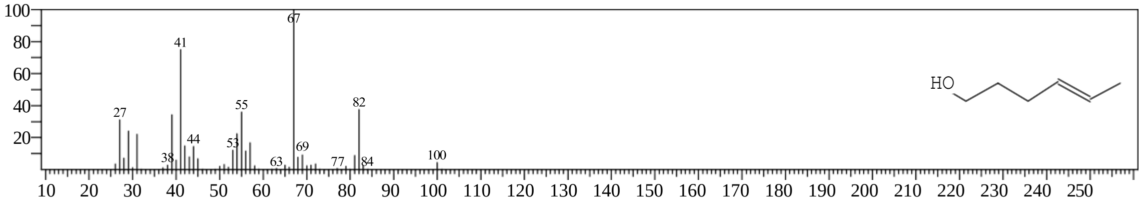

<< Target >>
Line#:4 R.Time:3.780(Scan#:757) MassPeaks:46
RawMode:Averaged 3.775-3.785(756-758) BasePeak:41.15(725759)
BG Mode:Calc. from Peak

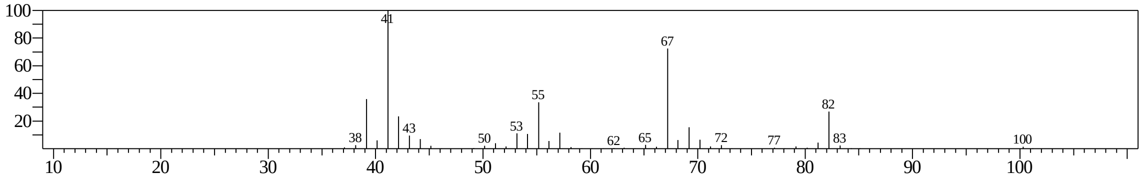

Hit#:1 Entry:1857 Library:NIST107.LIB
SI:96 Formula:C6H12O CAS:544-12-7 MolWeight:100 RetIndex:0
CompName:3-Hexen-1-ol $$ 3-Hexen-1-ol (c,t) $$

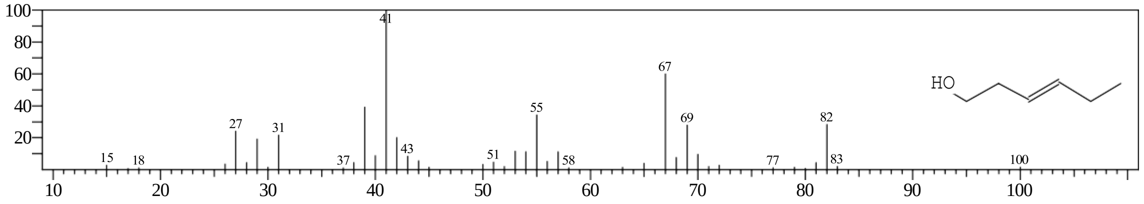

Hit#:2 Entry:1891 Library:NIST107.LIB
SI:93 Formula:C6H12O CAS:928-97-2 MolWeight:100 RetIndex:0
CompName:3-Hexen-1-ol, (E)- $$ trans-3-Hexen-1-ol $$ trans-3-Hexenol $$ E-3-Hexenol $$ (E)-Hex-3-en-1-ol $$

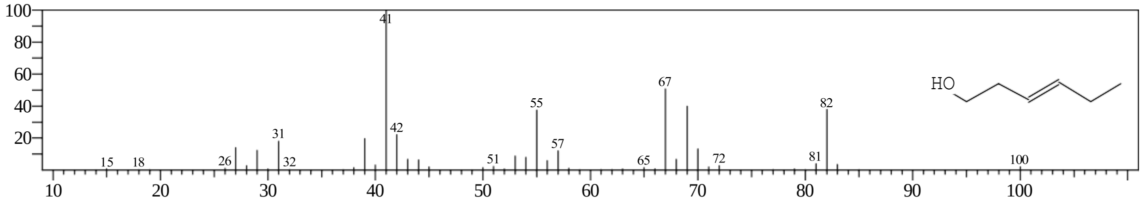

Hit#:3 Entry:1884 Library:NIST107.LIB
SI:93 Formula:C6H12O CAS:928-96-1 MolWeight:100 RetIndex:0
CompName:3-Hexen-1-ol, (Z)- $$ (Z)-Hex-3-en-1-ol $$ cis-3-Hexen-1-ol $$ cis-3-Hexene-1-ol $$ cis-3-Hexenol $$ Blatteralkohol (German) $$ Leaf alcoh

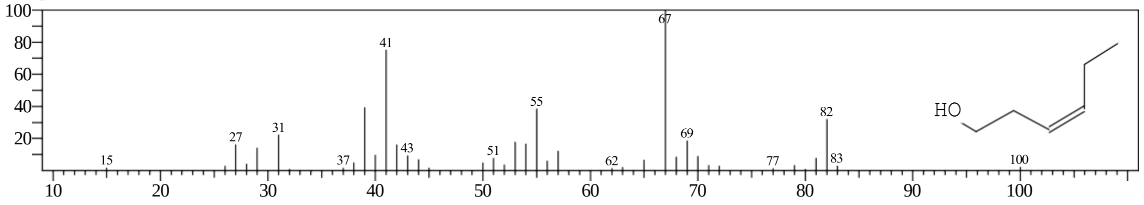

Hit#:4 Entry:1873 Library:NIST107.LIB
SI:92 Formula:C6H12O CAS:6126-50-7 MolWeight:100 RetIndex:0
CompName:4-Hexen-1-ol $$

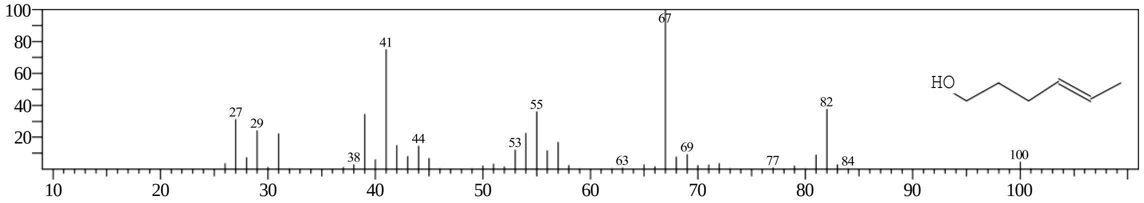

Hit#:5 Entry:1910 Library:NIST107.LIB
SI:92 Formula:C6H12O CAS:5673-98-3 MolWeight:100 RetIndex:0
CompName:4-Penten-1-ol, 2-methyl- $$

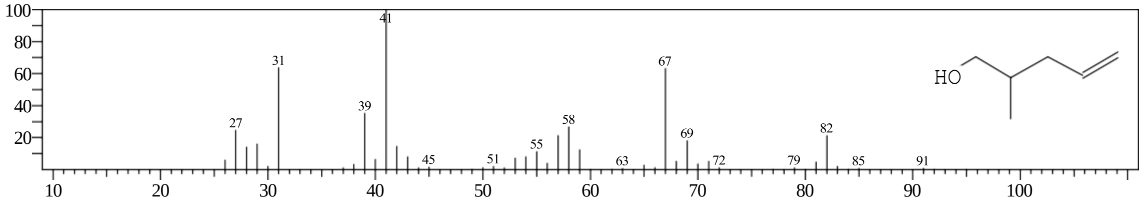

<< Target >>
Line#:5 R.Time:4.085(Scan#:818) MassPeaks:59
RawMode:Averaged 4.080-4.090(817-819) BasePeak:93.20(361433)
BG Mode:Calc. from Peak

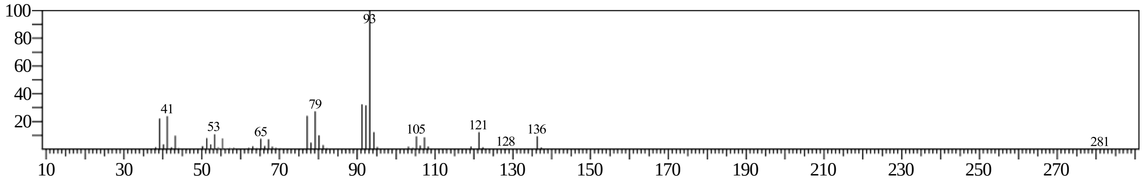

Hit#:1 Entry:8767 Library:NIST107.LIB
SI:97 Formula:C10H16 CAS:80-56-8 MolWeight:136 RetIndex:0
CompName:.alpha.-Pinene $$ Bicyclo[3.1.1]hept-2-ene, 2,6,6-trimethyl- $$ 2-Pinene $$ Pinene $$ 2,6,6-Trimethylbicyclo[3.1.1]hept-2-ene $$ Pinene isom

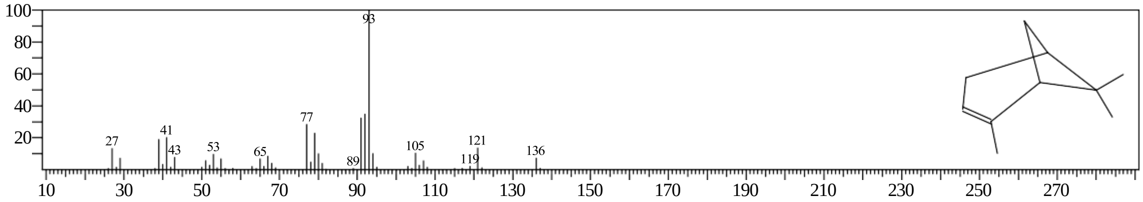

Hit#:2 Entry:8751 Library:NIST107.LIB
SI:96 Formula:C10H16 CAS:508-32-7 MolWeight:136 RetIndex:0
CompName:Tricyclo[2.2.1.02,6]heptane, 1,7,7-trimethyl- $$ Tricyclene $$ 1,7,7-Trimethyltricyclo[2.2.1.0(sup2,6)]heptane $$ 1,7,7-Trimethyltricyclo[2.2.1

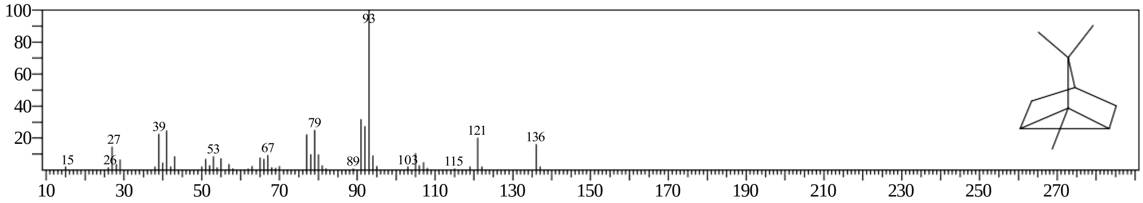

Hit#:3 Entry:19014 Library:NIST107.LIB
SI:95 Formula:C10H16N2 CAS:87143-58-6 MolWeight:164 RetIndex:0
CompName:3,5-Methanocyclopentapyrazole, 3,3a,4,5,6,6a-hexahydro-3a,4,4-trimethyl- $$

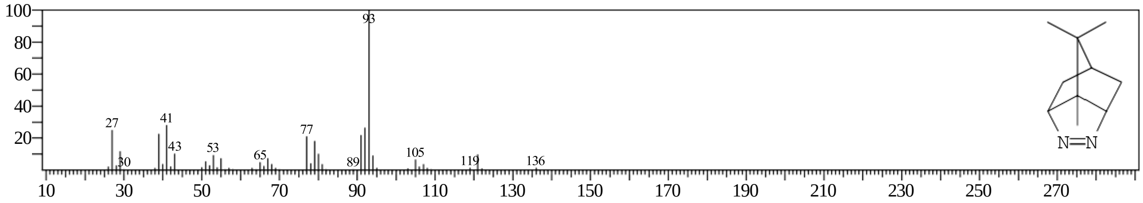

Hit#:4 Entry:8678 Library:NIST107.LIB
SI:95 Formula:C10H16 CAS:488-97-1 MolWeight:136 RetIndex:0
CompName:Tricyclo[2.2.1.02,6]heptane, 1,3,3-trimethyl- $$ Cyclofenchene $$ Cyclofeuchene $$

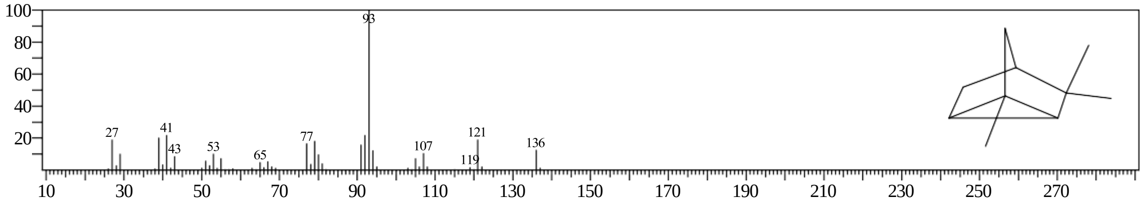

Hit#:5 Entry:8701 Library:NIST107.LIB
SI:95 Formula:C10H16 CAS:7785-26-4 MolWeight:136 RetIndex:0
CompName:(1S)-2,6,6-Trimethylbicyclo[3.1.1]hept-2-ene $$ 1S-.alpha.-Pinene $$ (-)-.alpha.-Pinene $$ L-.alpha.-Pinene $$ Bicyclo[3.1.1]hept-2-ene, 2,6,6

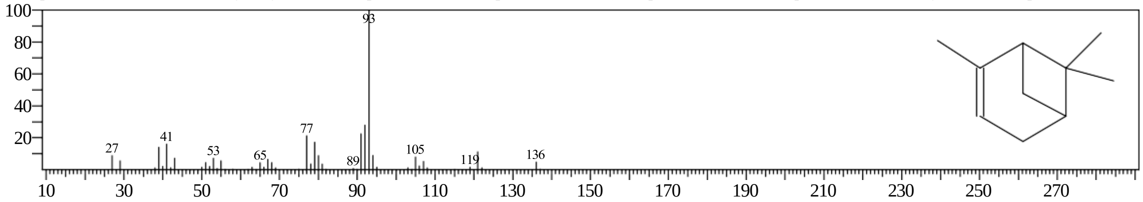

<< Target >>
Line#:6 R.Time:4.360(Scan#:873) MassPeaks:52
RawMode:Averaged 4.355-4.365(872-874) BasePeak:93.20(145190)
BG Mode:Calc. from Peak

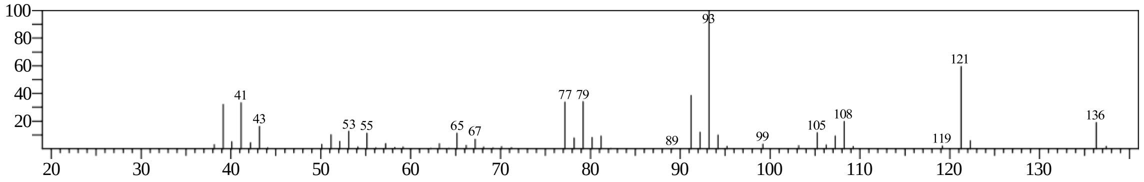

Hit#:1 Entry:8642 Library:NIST107.LIB
SI:93 Formula:C10H16 CAS:0-00-0 MolWeight:136 RetIndex:0
CompName:(+)-2-Caren $$

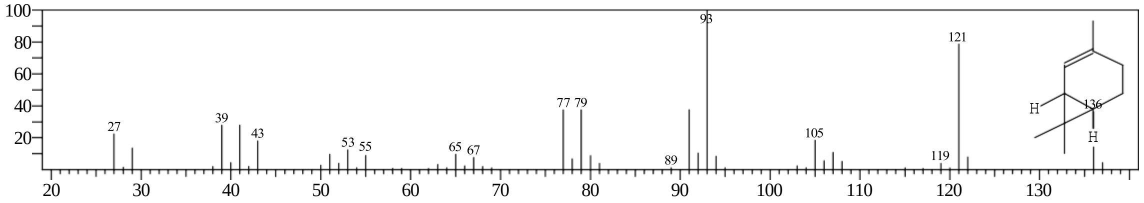

Hit#:2 Entry:8718 Library:NIST107.LIB
SI:93 Formula:C10H16 CAS:514-14-7 MolWeight:136 RetIndex:0
CompName:Bicyclo[2.2.1]hept-2-ene, 2,7,7-trimethyl- $$

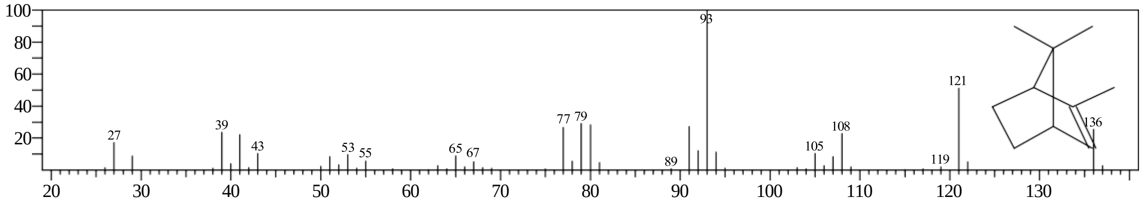

Hit#:3 Entry:8776 Library:NIST107.LIB
SI:93 Formula:C10H16 CAS:1727-69-1 MolWeight:136 RetIndex:0
CompName:Cyclopentene, 4-ethenyl-1,5,5-trimethyl- $$

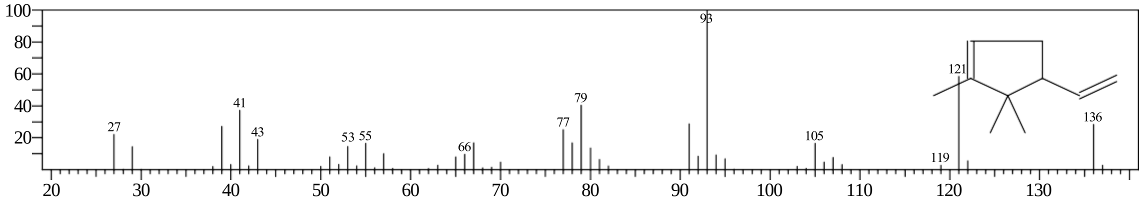

Hit#:4 Entry:8778 Library:NIST107.LIB
SI:92 Formula:C10H16 CAS:99805-90-0 MolWeight:136 RetIndex:0
CompName:Cyclohexene, 4-methyl-3-(1-methylethylidene)- $$

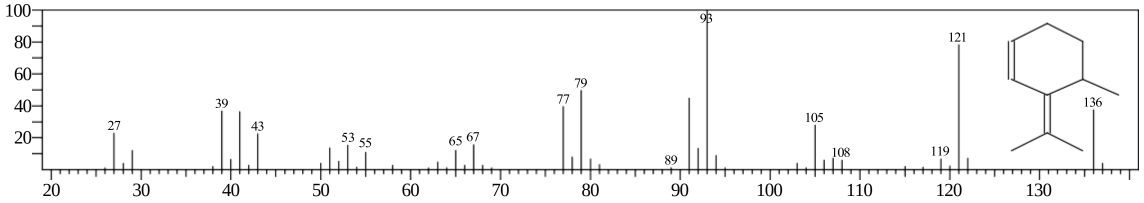

Hit#:5 Entry:8702 Library:NIST107.LIB
SI:92 Formula:C10H16 CAS:79-92-5 MolWeight:136 RetIndex:0
CompName:Camphene $$ Bicyclo[2.2.1]heptane, 2,2-dimethyl-3-methylene- $$ 2,2-Dimethyl-3-methylenebicyclo[2.2.1]heptane $$ 2,2-Dimethyl-3-methyl

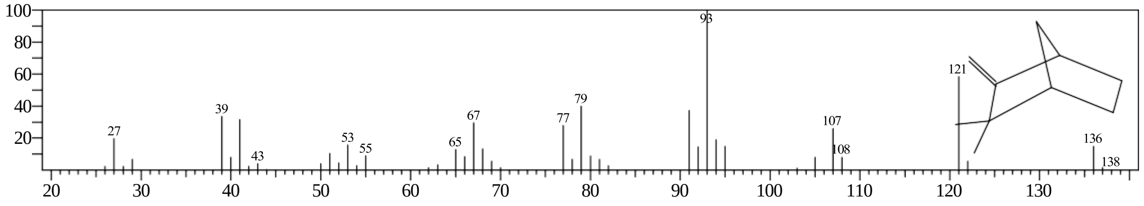

<< Target >>
Line#:7 R.Time:4.435(Scan#:888) MassPeaks:50
RawMode:Averaged 4.430-4.440(887-889) BasePeak:93.20(131611)
BG Mode:Calc. from Peak

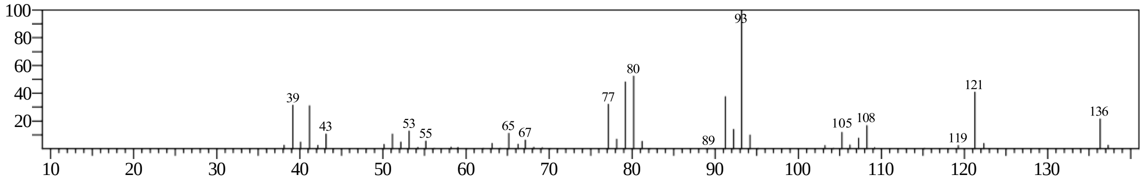

Hit#:1 Entry:8718 Library:NIST107.LIB
SI:94 Formula:C10H16 CAS:514-14-7 MolWeight:136 RetIndex:0
CompName:Bicyclo[2.2.1]hept-2-ene, 2,7,7-trimethyl- $$

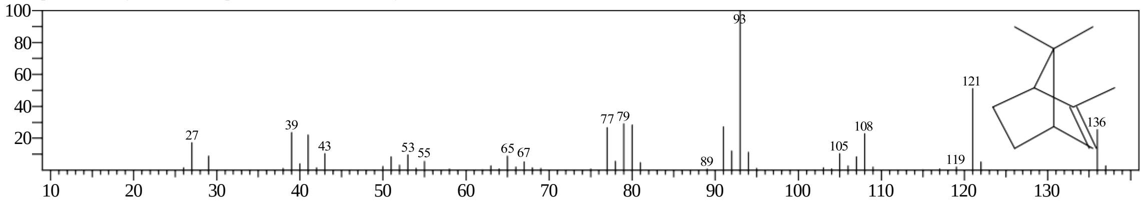

Hit#:2 Entry:8652 Library:NIST107.LIB
SI:92 Formula:C10H16 CAS:0-00-0 MolWeight:136 RetIndex:0
CompName:Cyclopentene, 3-isopropenyl-5,5-dimethyl- $$

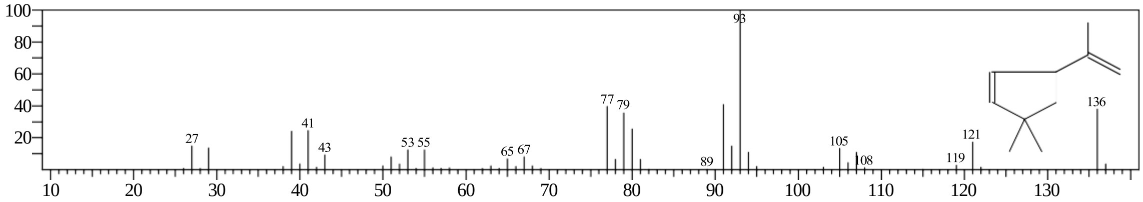

Hit#:3 Entry:8642 Library:NIST107.LIB
SI:92 Formula:C10H16 CAS:0-00-0 MolWeight:136 RetIndex:0
CompName:(+)-2-Caren $$

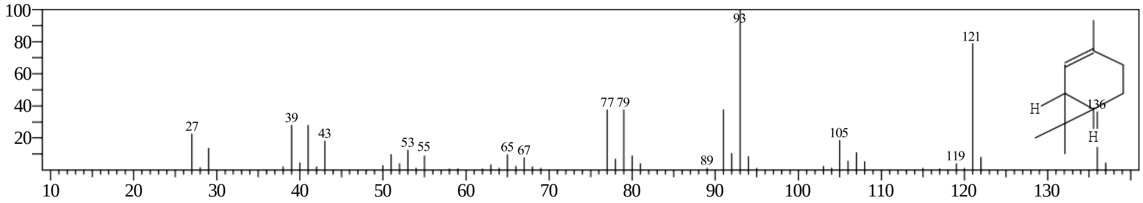

Hit#:4 Entry:8695 Library:NIST107.LIB
SI:91 Formula:C10H16 CAS:471-84-1 MolWeight:136 RetIndex:0
CompName:Bicyclo[2.2.1]heptane, 7,7-dimethyl-2-methylene- $$ Norbornane, 7,7-dimethyl-2-methylene- $$ .alpha.-Fenchene $$ Fenchene $$ 7,7-Dimethy

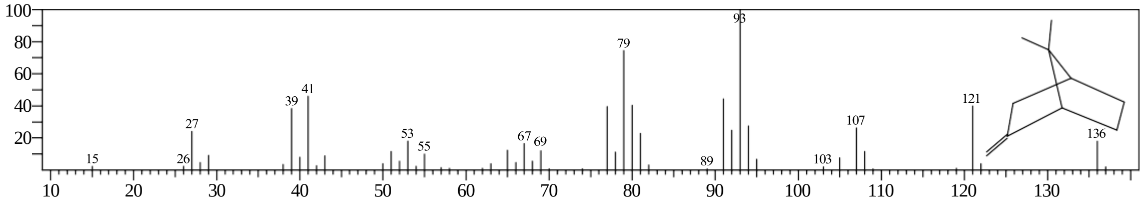

Hit#:5 Entry:8708 Library:NIST107.LIB
SI:91 Formula:C10H16 CAS:68998-21-0 MolWeight:136 RetIndex:0
CompName:Cyclopropane, 1,1-dimethyl-2-(3-methyl-1,3-butadienyl)- $$

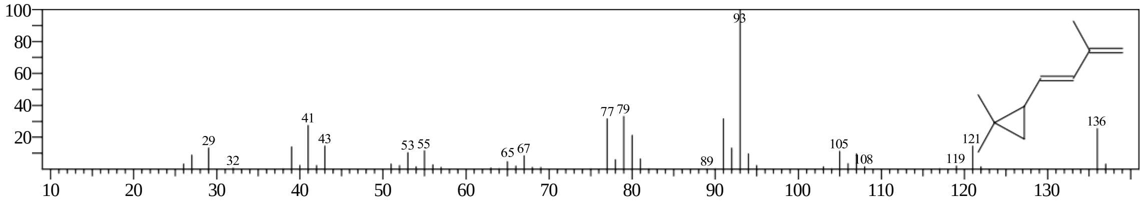

<< Target >>
Line#:8 R.Time:4.555(Scan#:912) MassPeaks:32
RawMode:Averaged 4.550-4.560(911-913) BasePeak:55.15(33441)
BG Mode:Calc. from Peak

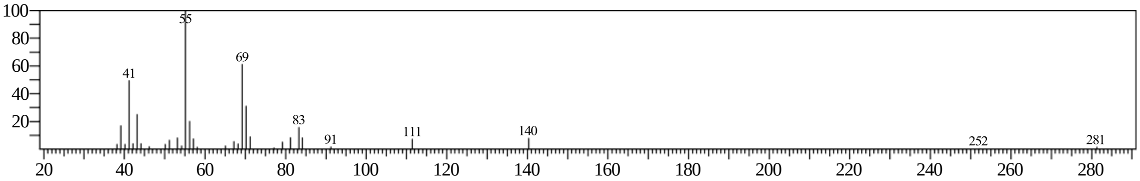

Hit#:1 Entry:10138 Library:NIST107.LIB
SI:91 Formula:C10H20 CAS:15918-07-7 MolWeight:140 RetIndex:0
CompName:4-Nonene, 5-methyl- $$ 5-Methyl-4-nonene $$

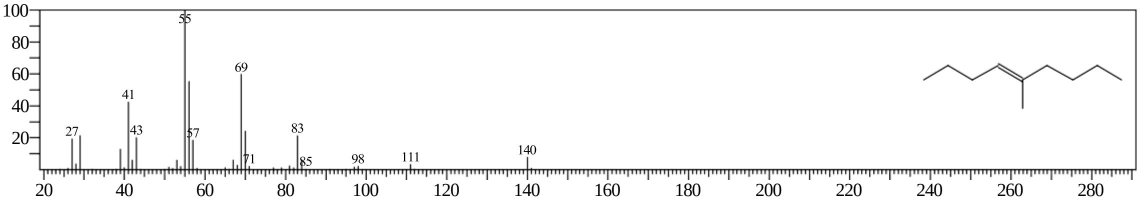

Hit#:2 Entry:10096 Library:NIST107.LIB
SI:90 Formula:C10H20 CAS:63830-69-3 MolWeight:140 RetIndex:0
CompName:4-Nonene, 3-methyl-, (Z)- $$

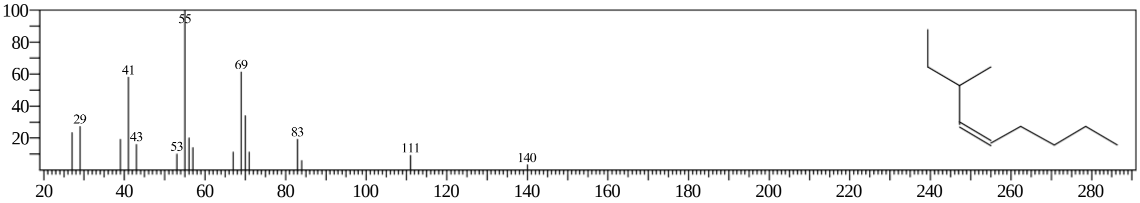

Hit#:3 Entry:10128 Library:NIST107.LIB
SI:90 Formula:C10H20 CAS:69405-42-1 MolWeight:140 RetIndex:0
CompName:3-Nonene, 3-methyl-, (E)- $$

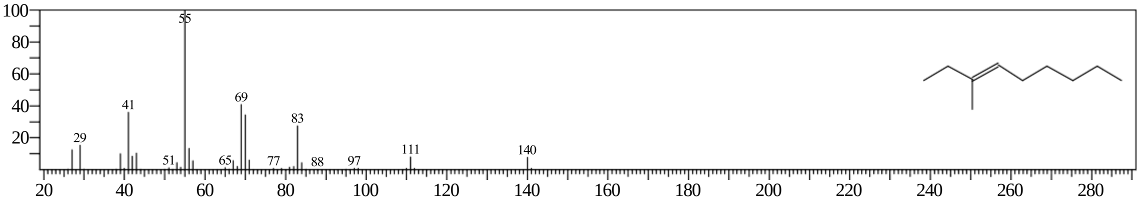

Hit#:4 Entry:10104 Library:NIST107.LIB
SI:89 Formula:C10H20 CAS:53966-52-2 MolWeight:140 RetIndex:0
CompName:2-Octene, 4-ethyl- $$

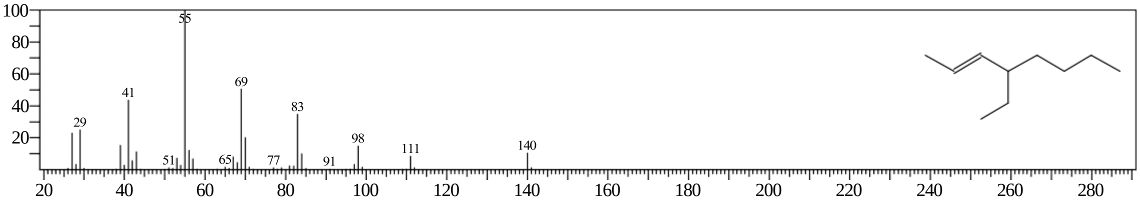

Hit#:5 Entry:10100 Library:NIST107.LIB
SI:89 Formula:C10H20 CAS:53966-51-1 MolWeight:140 RetIndex:0
CompName:3-Octene, 4-ethyl- $$

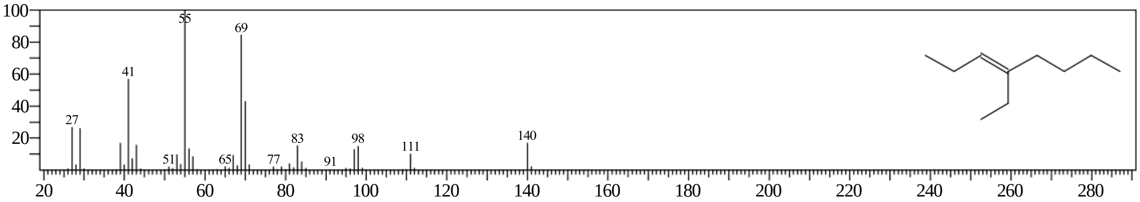

<< Target >>
Line#:9 R.Time:4.700(Scan#:941) MassPeaks:96
RawMode:Averaged 4.695-4.705(940-942) BasePeak:93.25(8346819)
BG Mode:Calc. from Peak

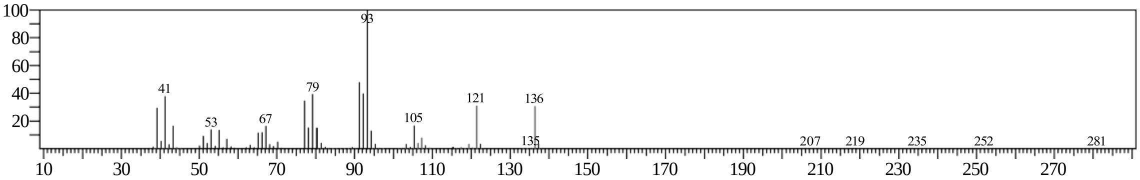

Hit#:1 Entry:8776 Library:NIST107.LIB
SI:94 Formula:C10H16 CAS:1727-69-1 MolWeight:136 RetIndex:0
CompName:Cyclopentene, 4-ethenyl-1,5,5-trimethyl- $$

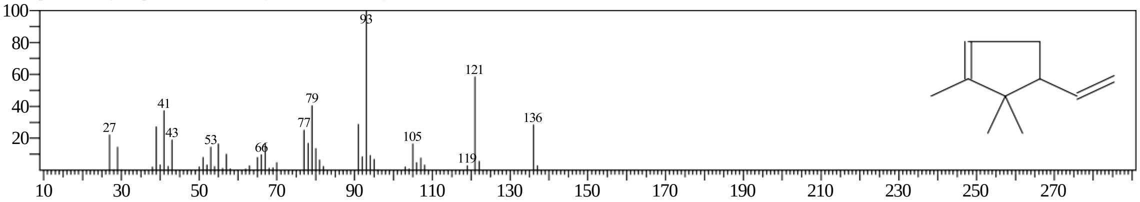

Hit#:2 Entry:8652 Library:NIST107.LIB
SI:92 Formula:C10H16 CAS:0-00-0 MolWeight:136 RetIndex:0
CompName:Cyclopentene, 3-isopropenyl-5,5-dimethyl- $$

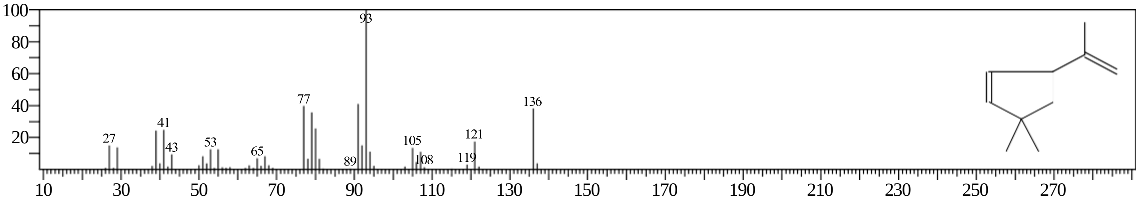

Hit#:3 Entry:8709 Library:NIST107.LIB
SI:92 Formula:C10H16 CAS:2437-95-8 MolWeight:136 RetIndex:0
CompName:Bicyclo[3.1.1]hept-2-ene, 2,6,6-trimethyl-, (.+/-.)- $$

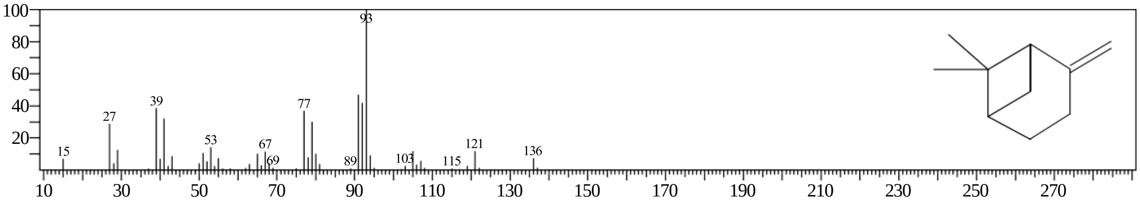

Hit#:4 Entry:8704 Library:NIST107.LIB
SI:91 Formula:C10H16 CAS:3338-55-4 MolWeight:136 RetIndex:0
CompName:1,3,6-Octatriene, 3,7-dimethyl-, (Z)- $$ .beta.-cis-Ocimene $$ cis-.beta.-Ocimene $$ cis-3,7-Dimethyl-1,3,6-Octatriene $$ Ocimene, cis-.beta.- $

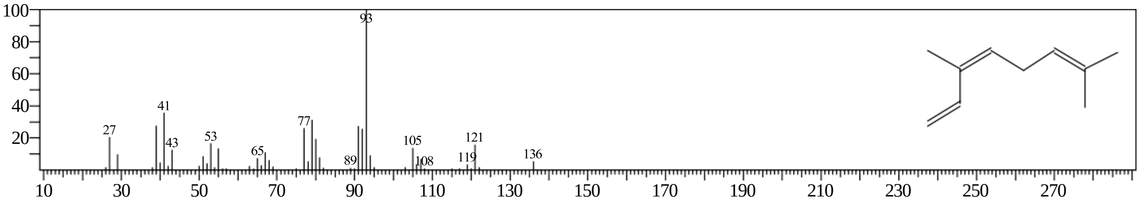

Hit#:5 Entry:8778 Library:NIST107.LIB
SI:91 Formula:C10H16 CAS:99805-90-0 MolWeight:136 RetIndex:0
CompName:Cyclohexene, 4-methyl-3-(1-methylethylidene)- $$

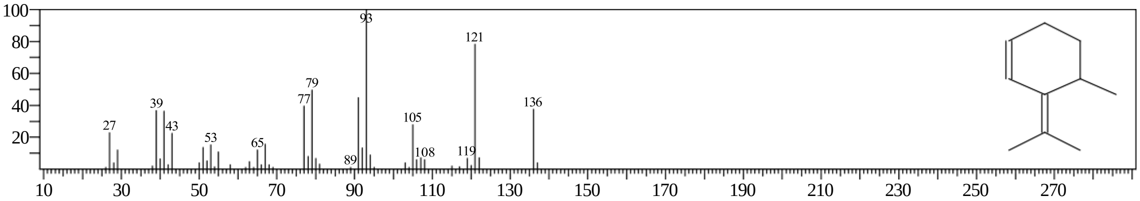

<< Target >>
Line#:10 R.Time:4.895(Scan#:980) MassPeaks:100
RawMode:Averaged 4.890-4.900(979-981) BasePeak:93.25(8363053)
BG Mode:Calc. from Peak

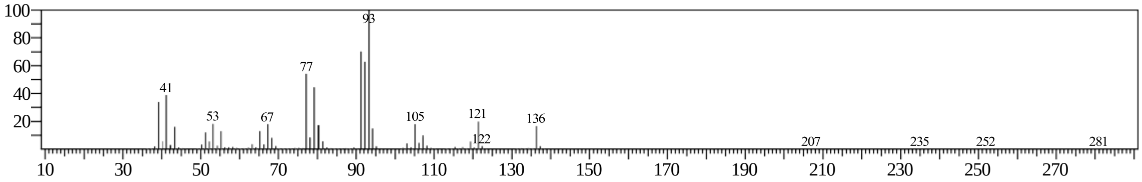

Hit#:1 Entry:8709 Library:NIST107.LIB
SI:92 Formula:C10H16 CAS:2437-95-8 MolWeight:136 RetIndex:0
CompName:Bicyclo[3.1.1]hept-2-ene, 2,6,6-trimethyl-, (.+/-.)- $$

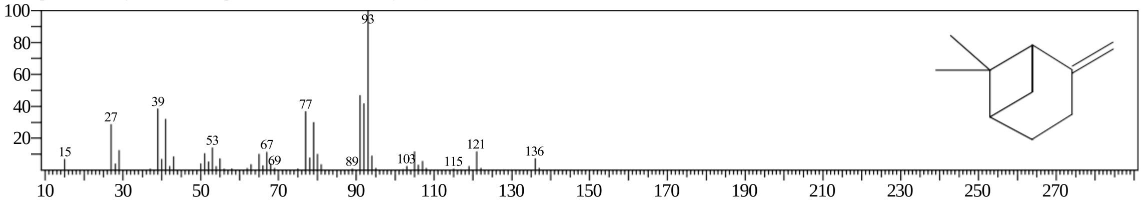

Hit#:2 Entry:8704 Library:NIST107.LIB
SI:91 Formula:C10H16 CAS:3338-55-4 MolWeight:136 RetIndex:0
CompName:1,3,6-Octatriene, 3,7-dimethyl-, (Z)- $$ .beta.-cis-Ocimene $$ cis-.beta.-Ocimene $$ cis-3,7-Dimethyl-1,3,6-Octatriene $$ Ocimene, cis-.beta.- $

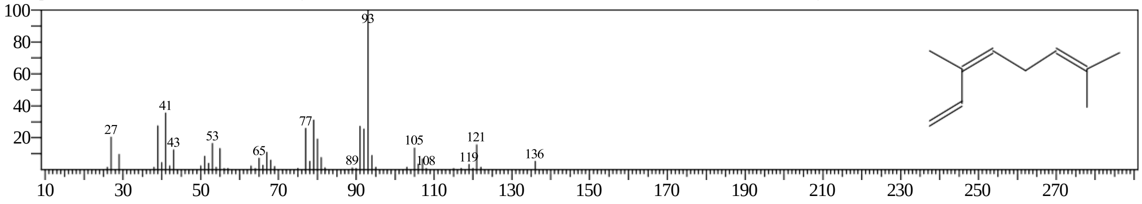

Hit#:3 Entry:8796 Library:NIST107.LIB
SI:90 Formula:C10H16 CAS:29548-02-5 MolWeight:136 RetIndex:0
CompName:1,3,6-Heptatriene, 2,5,5-trimethyl- $$

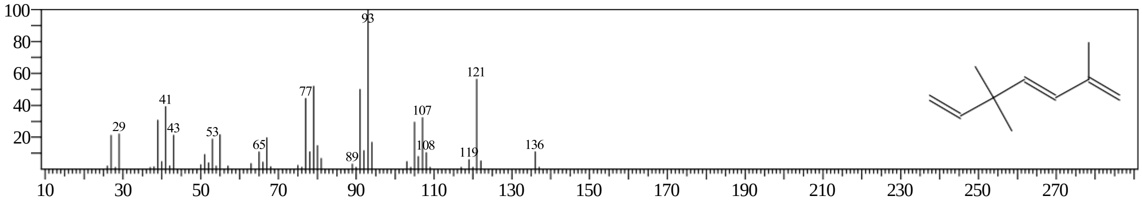

Hit#:4 Entry:8778 Library:NIST107.LIB
SI:90 Formula:C10H16 CAS:99805-90-0 MolWeight:136 RetIndex:0
CompName:Cyclohexene, 4-methyl-3-(1-methylethylidene)- $$

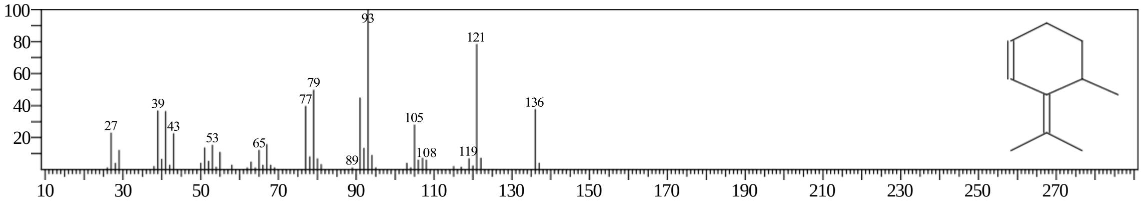

Hit#:5 Entry:8652 Library:NIST107.LIB
SI:90 Formula:C10H16 CAS:0-00-0 MolWeight:136 RetIndex:0
CompName:Cyclopentene, 3-isopropenyl-5,5-dimethyl- $$

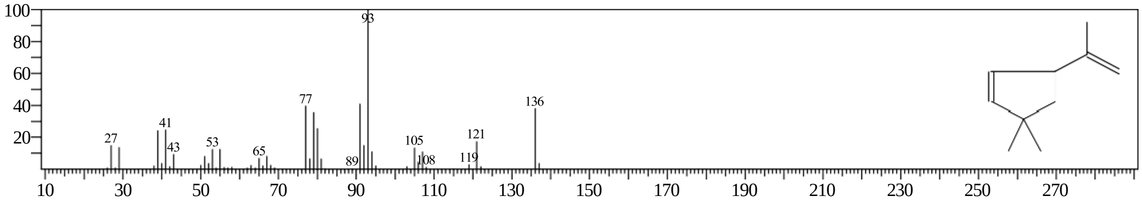

<< Target >>
Line#:11 R.Time:4.950(Scan#:991) MassPeaks:50
RawMode:Averaged 4.945-4.955(990-992) BasePeak:79.20(153748)
BG Mode:Calc. from Peak

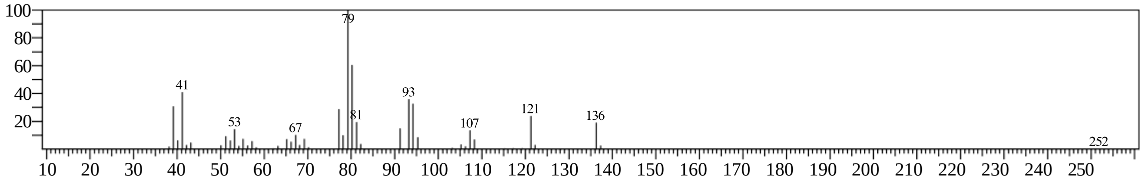

Hit#:1 Entry:8530 Library:NIST107.LIB
SI:91 Formula:C9H12O CAS:0-00-0 MolWeight:136 RetIndex:0
CompName:Bicyclo[3.3.0]oct-2-en-7-one, 6-methyl- $$

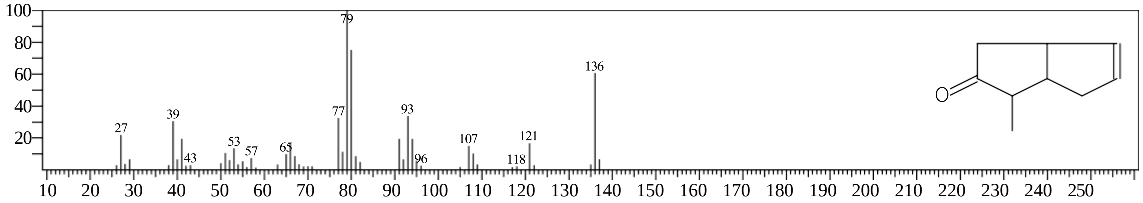

Hit#:2 Entry:8683 Library:NIST107.LIB
SI:88 Formula:C10H16 CAS:70058-00-3 MolWeight:136 RetIndex:0
CompName:2-Methyl-1-nonene-3-yne $$ 2-Methyl-1-nonen-3-yne $$ 1-Nonen-3-yne, 2-methyl- $$

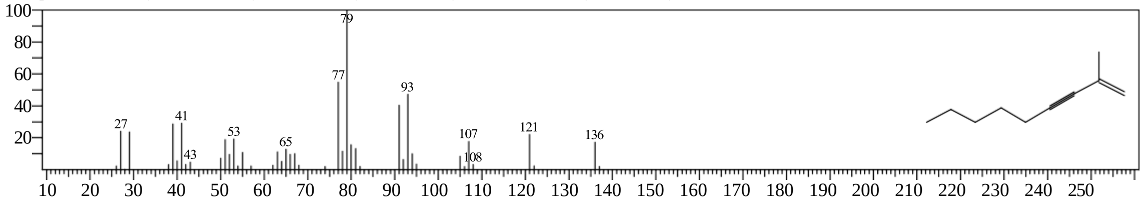

Hit#:3 Entry:8763 Library:NIST107.LIB
SI:87 Formula:C10H16 CAS:586-67-4 MolWeight:136 RetIndex:0
CompName:Cyclohexene, 4-methyl-1-(1-methylethenyl)- $$ p-Mentha-3,8-diene $$ 3,8-p-Menthadiene $$

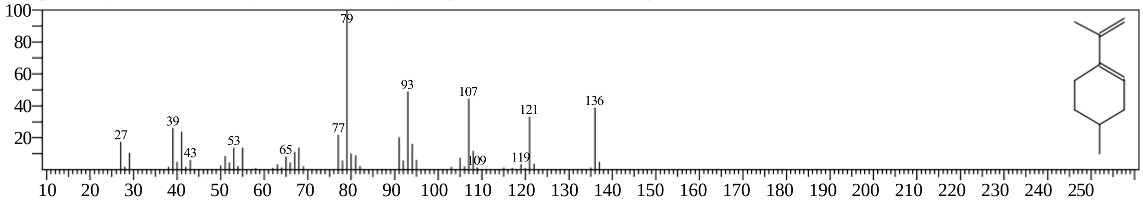

Hit#:4 Entry:8695 Library:NIST107.LIB
SI:87 Formula:C10H16 CAS:471-84-1 MolWeight:136 RetIndex:0
CompName:Bicyclo[2.2.1]heptane, 7,7-dimethyl-2-methylene- $$ Norbornane, 7,7-dimethyl-2-methylene- $$ .alpha.-Fenchene $$ Fenchene $$ 7,7-Dimethy

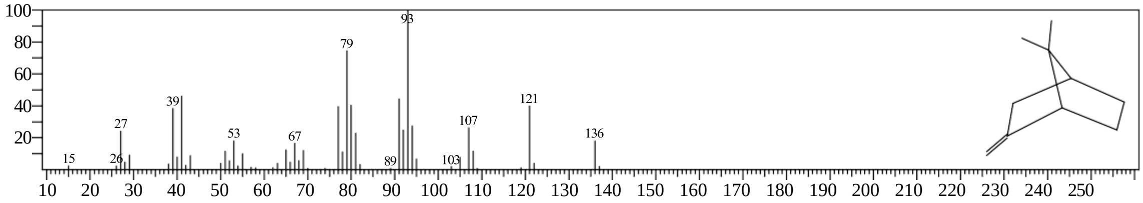

Hit#:5 Entry:8792 Library:NIST107.LIB
SI:87 Formula:C10H16 CAS:5794-03-6 MolWeight:136 RetIndex:0
CompName:Bicyclo[2.2.1]heptane, 2,2-dimethyl-3-methylene-, (1R)- $$ Camphene, (1R,4S)-(+)- $$ (+)-Camphene $$ d-Camphene $$

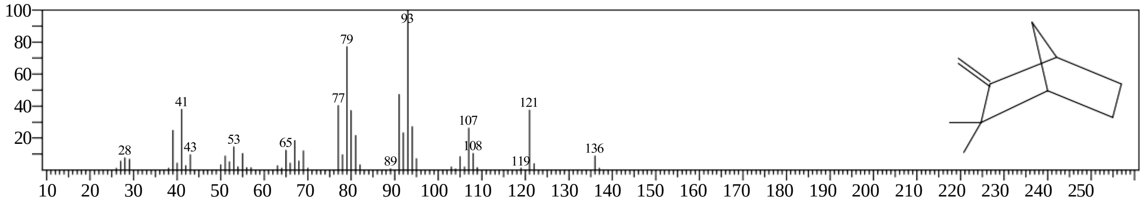

<< Target >>
Line#:12 R.Time:5.160(Scan#:1033) MassPeaks:102
RawMode:Averaged 5.155-5.165(1032-1034) BasePeak:121.25(8359674)
BG Mode:Calc. from Peak

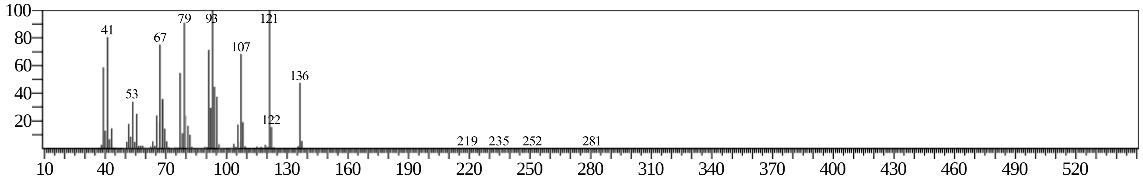

Hit#:1 Entry:8755 Library:NIST107.LIB
SI:91 Formula:C10H16 CAS:53282-47-6 MolWeight:136 RetIndex:0
CompName:Bicyclo[4.1.0]heptane, 7-(1-methylethylidene)- $$

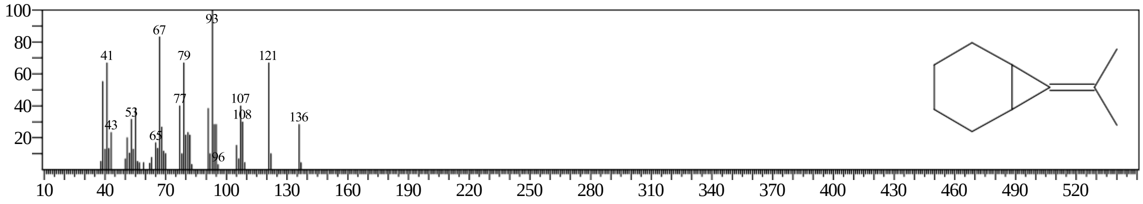

Hit#:2 Entry:8732 Library:NIST107.LIB
SI:90 Formula:C10H16 CAS:13837-95-1 MolWeight:136 RetIndex:0
CompName:Cyclohexane, 1-methylene-3-(1-methylethenyl)-, (R)- $$ m-Mentha-1(7),8-diene, (R)-(-)- $$

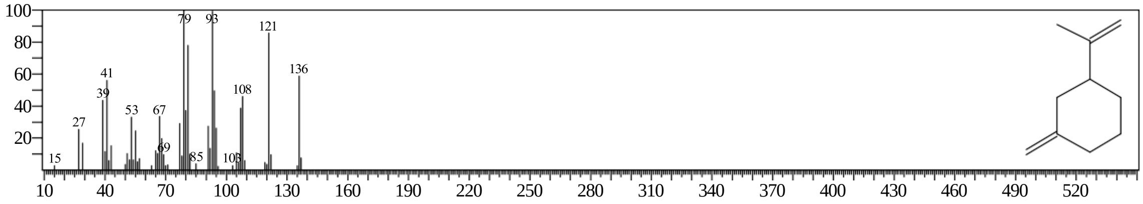

Hit#:3 Entry:8681 Library:NIST107.LIB
SI:89 Formula:C10H16 CAS:5113-87-1 MolWeight:136 RetIndex:0
CompName:Cyclohexene, 3-methyl-6-(1-methylethenyl)-, (3R-trans)- $$ p-Mentha-2,8-diene, (1R,4R)-(+)- $$ (+)-(1R,4R)-trans-Isolimonene $$ (+)-trans-I

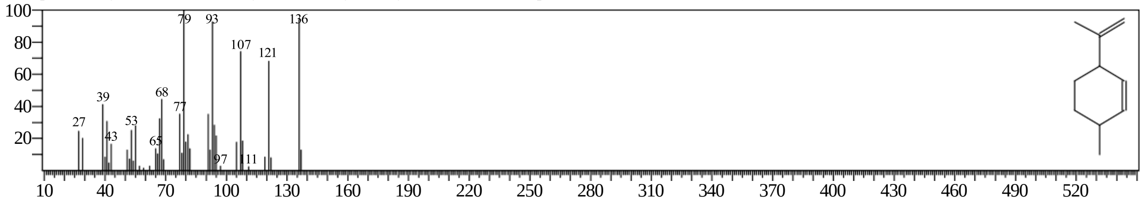

Hit#:4 Entry:8740 Library:NIST107.LIB
SI:89 Formula:C10H16 CAS:42123-66-0 MolWeight:136 RetIndex:0
CompName:1,3,6-Heptatriene, 2,5,6-trimethyl- $$

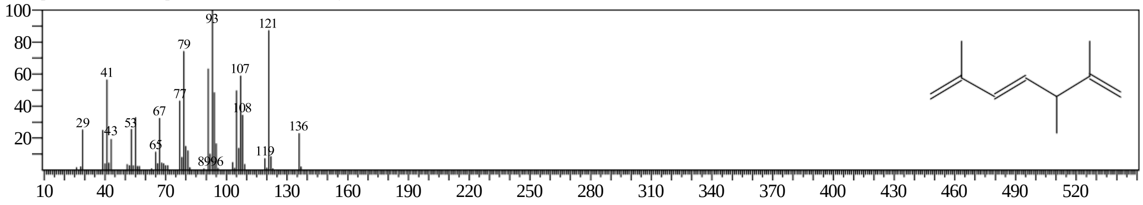

Hit#:5 Entry:104320 Library:NIST107.LIB
SI:88 Formula:C20H32Cl2Rh2 CAS:74842-27-6 MolWeight:548 RetIndex:0
CompName:Rhodium, bis[1,2-bis(.eta.2-ethenyl)cyclohexane]di-.mu.-chlorodi-, stereoisomer $$

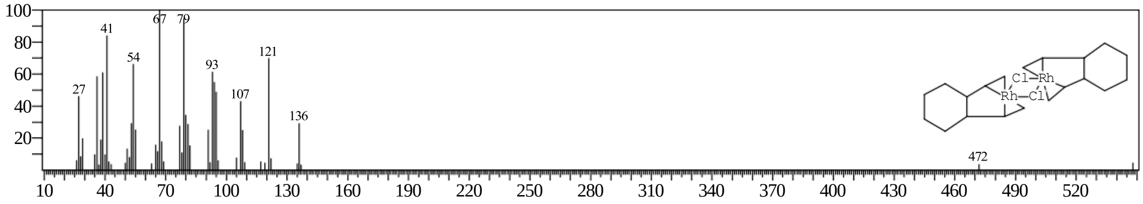

<< Target >>
Line#:13 R.Time:5.465(Scan#:1094) MassPeaks:75
RawMode:Averaged 5.460-5.470(1093-1095) BasePeak:119.25(1804774)
BG Mode:Calc. from Peak

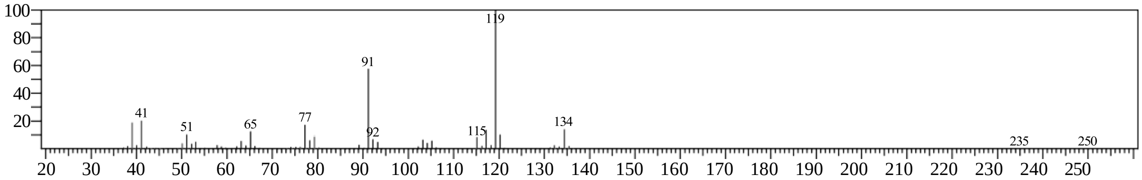

Hit#:1 Entry:8115 Library:NIST107.LIB
SI:93 Formula:C10H14 CAS:3479-89-8 MolWeight:134 RetIndex:0
CompName:1,3,5-Cycloheptatriene, 3,7,7-trimethyl- $$

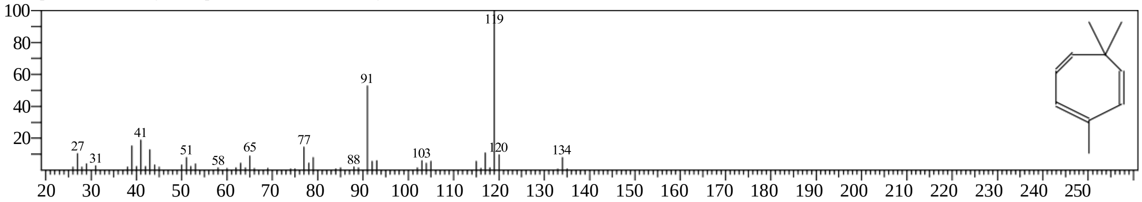

Hit#:2 Entry:8101 Library:NIST107.LIB
SI:91 Formula:C10H14 CAS:98-06-6 MolWeight:134 RetIndex:0
CompName:Benzene, tert-butyl- $$ Benzene, (1,1-dimethylethyl)- $$ tert-Butylbenzene $$ Dimethylethylbenzene $$ Phenyltrimethylmethane $$ Pseudobut

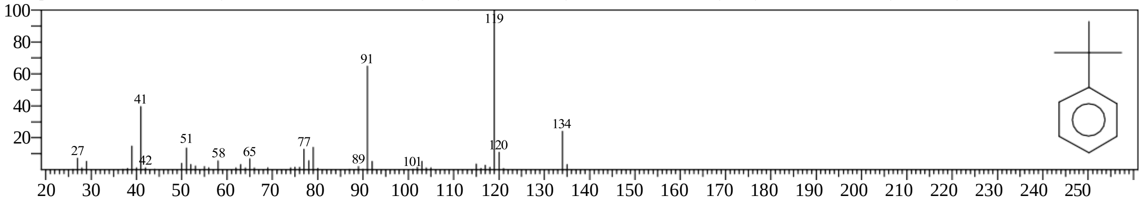

Hit#:3 Entry:8099 Library:NIST107.LIB
SI:90 Formula:C10H14 CAS:62338-57-2 MolWeight:134 RetIndex:0
CompName:1,4-Cyclohexadiene, 3-ethenyl-1,2-dimethyl- $$

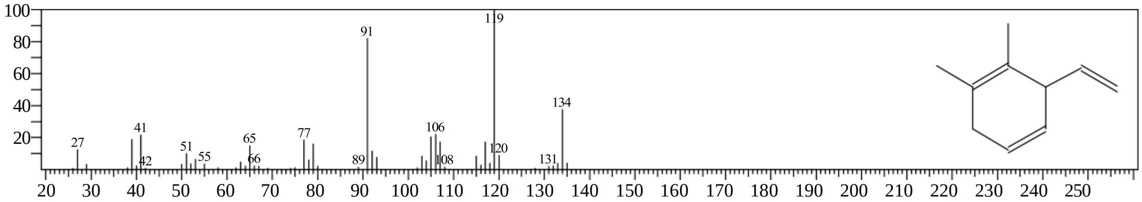

Hit#:4 Entry:14151 Library:NIST107.LIB
SI:89 Formula:C10H16O CAS:0-00-0 MolWeight:152 RetIndex:0
CompName:(E)-3-Caren-2-ol $$

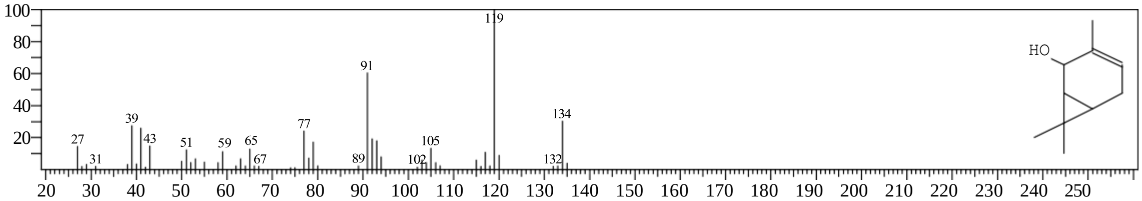

Hit#:5 Entry:14236 Library:NIST107.LIB
SI:89 Formula:C10H16O CAS:20053-58-1 MolWeight:152 RetIndex:0
CompName:2,3-Epoxycarane, (E)- $$

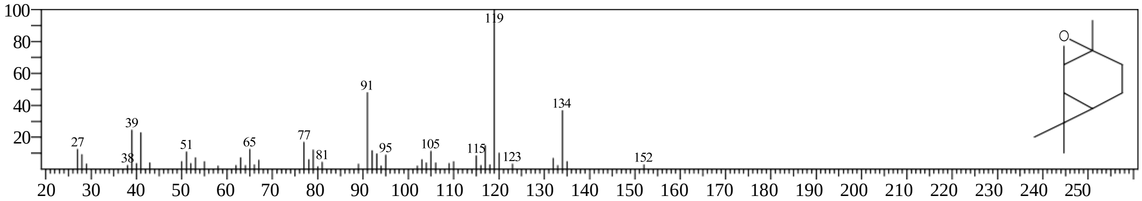

<< Target >>
Line#:14 R.Time:5.560(Scan#:1113) MassPeaks:89
RawMode:Averaged 5.555-5.565(1112-1114) BasePeak:93.20(7619021)
BG Mode:Calc. from Peak

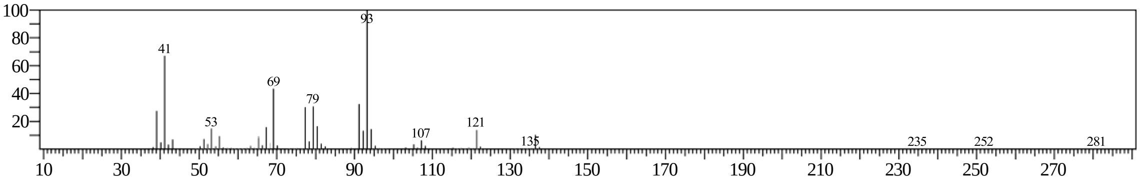

Hit#:1 Entry:8752 Library:NIST107.LIB
SI:97 Formula:C10H16 CAS:127-91-3 MolWeight:136 RetIndex:0
CompName:.beta.-Pinene $$ Bicyclo[3.1.1]heptane, 6,6-dimethyl-2-methylene- $$ 2(10)-Pinene $$ Nopinen $$ Nopinene $$ Pseudopinen $$ Pseudopinene

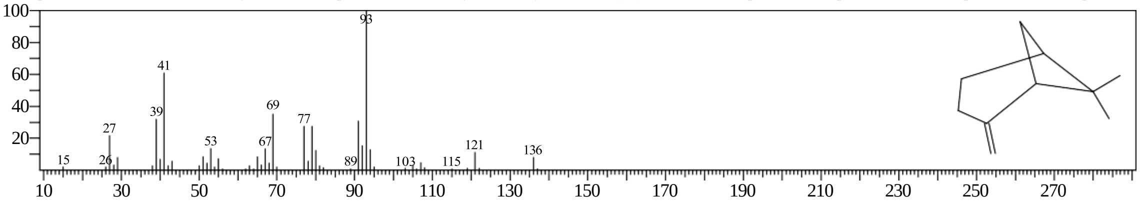

Hit#:2 Entry:8670 Library:NIST107.LIB
SI:95 Formula:C10H16 CAS:18172-67-3 MolWeight:136 RetIndex:0
CompName:Bicyclo[3.1.1]heptane, 6,6-dimethyl-2-methylene-, (1S)- $$ 2(10)-Pinene, (1S,5S)-(-)- $$ (-)-.beta.-Pinene $$ (-)-2(10)-Pinene $$ L-.beta.-pinen

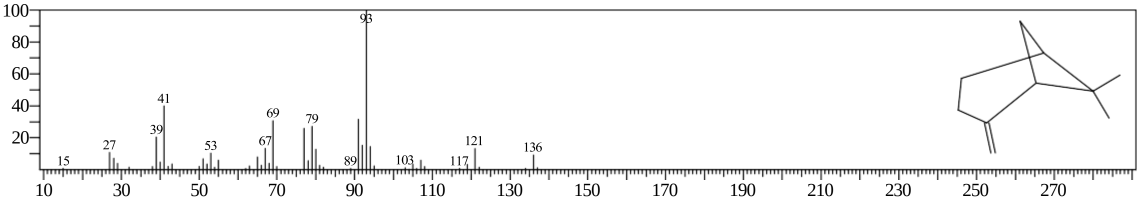

Hit#:3 Entry:8686 Library:NIST107.LIB
SI:92 Formula:C10H16 CAS:3387-41-5 MolWeight:136 RetIndex:0
CompName:Bicyclo[3.1.0]hexane, 4-methylene-1-(1-methylethyl)- $$ 4(10)-Thujene $$ Sabinen $$ Sabinene $$ (+)-Sabinene $$ THUJENE, 4(10)- $$

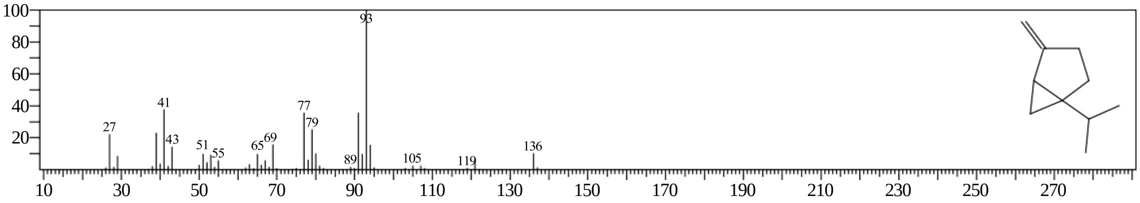

Hit#:4 Entry:8704 Library:NIST107.LIB
SI:91 Formula:C10H16 CAS:3338-55-4 MolWeight:136 RetIndex:0
CompName:1,3,6-Octatriene, 3,7-dimethyl-, (Z)- $$ .beta.-cis-Ocimene $$ cis-.beta.-Ocimene $$ cis-3,7-Dimethyl-1,3,6-Octatriene $$ Ocimene, cis-.beta.- $

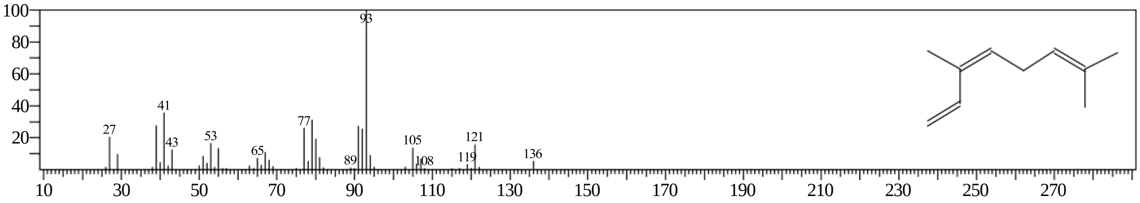

Hit#:5 Entry:8692 Library:NIST107.LIB
SI:91 Formula:C10H16 CAS:3779-61-1 MolWeight:136 RetIndex:0
CompName:1,3,6-Octatriene, 3,7-dimethyl-, (E)- $$ .beta.-trans-Ocimene $$ trans-.beta.-Ocimene $$ trans-3,7-Dimethyl-1,3,6-Octatriene $$ Ocimene, trans

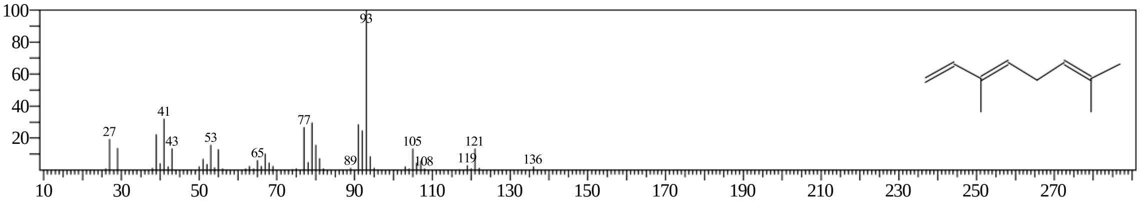

<< Target >>
Line#:15 R.Time:5.680(Scan#:1137) MassPeaks:45
RawMode:Averaged 5.675-5.685(1136-1138) BasePeak:95.20(46463)
BG Mode:Calc. from Peak

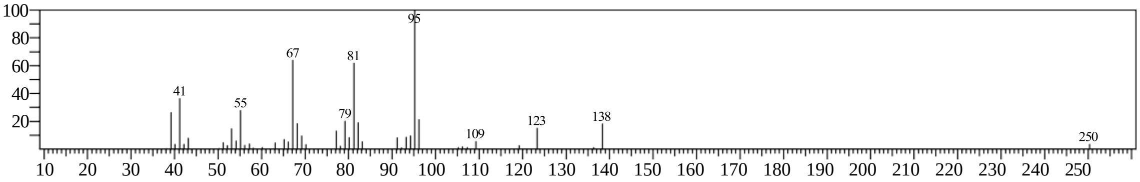

Hit#:1 Entry:9292 Library:NIST107.LIB
SI:93 Formula:C10H18 CAS:0-00-0 MolWeight:138 RetIndex:0
CompName:3-Menthene $$

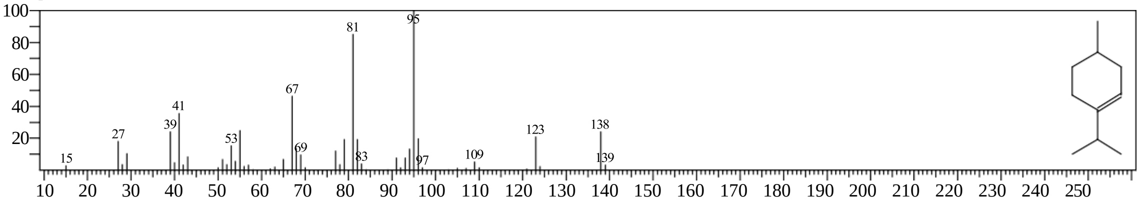

Hit#:2 Entry:9318 Library:NIST107.LIB
SI:93 Formula:C10H18 CAS:500-00-5 MolWeight:138 RetIndex:0
CompName:Cyclohexene, 4-methyl-1-(1-methylethyl)- $$ p-Menth-3-ene $$ .delta.3-p-Menthene $$ Menthomenthene $$ 3-p-Menthene $$ 4-Methyl-1-iso-

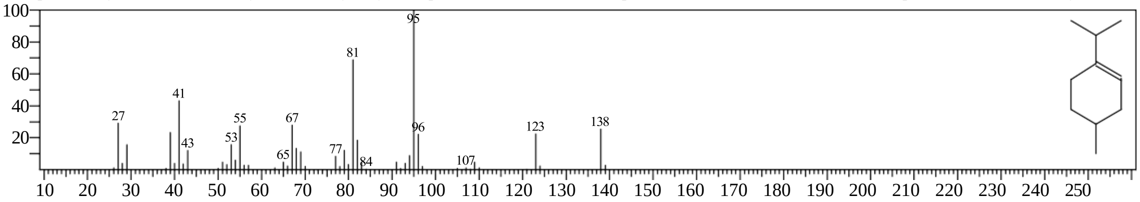

Hit#:3 Entry:9386 Library:NIST107.LIB
SI:92 Formula:C10H18 CAS:18968-23-5 MolWeight:138 RetIndex:0
CompName:Bicyclo[4.1.0]heptane, 3,7,7-trimethyl-, (1.alpha.,3.alpha.,6.alpha.)- $$ Carane, trans- $$ trans-Carane $$

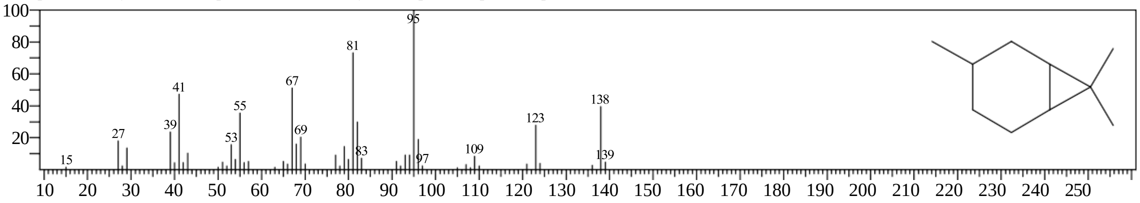

Hit#:4 Entry:9336 Library:NIST107.LIB
SI:92 Formula:C10H18 CAS:2778-68-9 MolWeight:138 RetIndex:0
CompName:Bicyclo[4.1.0]heptane, 3,7,7-trimethyl-, [1S-(1.alpha.,3.beta.,6.alpha.)]- $$ Carane, (1S,3S,6R)-(-)- $$ (-)-cis-Carane $$

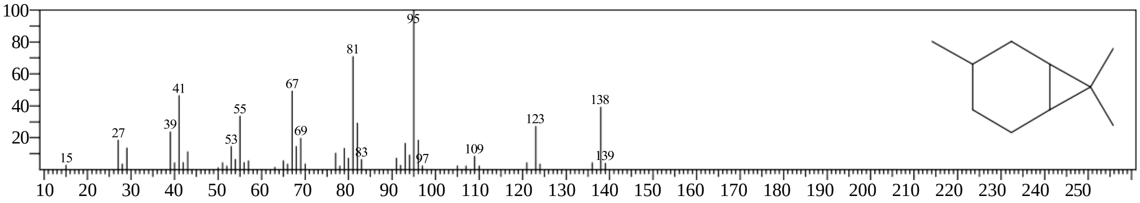

Hit#:5 Entry:9382 Library:NIST107.LIB
SI:91 Formula:C10H18 CAS:5502-88-5 MolWeight:138 RetIndex:0
CompName:Cyclohexene, 1-methyl-4-(1-methylethyl)- $$ .delta.(Sup1)-p-Menthene $$ p-Menth-1-ene $$ Carvomenthene $$ 1-p-Menthene $$ 1-Methyl-4-

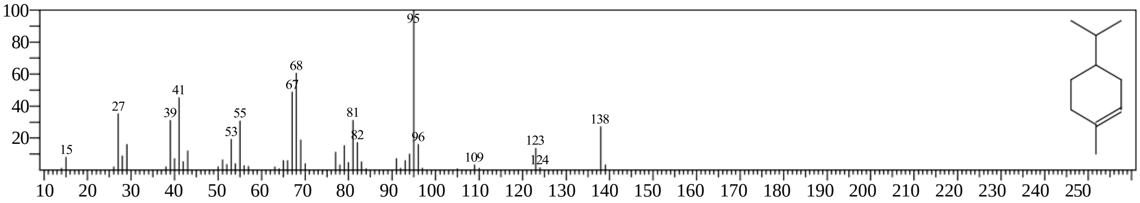

<< Target >>
Line#:16 R.Time:5.810(Scan#:1163) MassPeaks:69
RawMode:Averaged 5.805-5.815(1162-1164) BasePeak:41.15(1388382)
BG Mode:Calc. from Peak

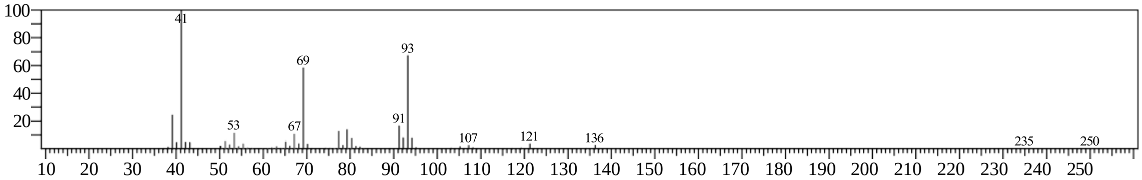

Hit#:1 Entry:8783 Library:NIST107.LIB
SI:95 Formula:C10H16 CAS:123-35-3 MolWeight:136 RetIndex:0
CompName:.beta.-Myrcene $$ 1,6-Octadiene, 7-methyl-3-methylene- $$ Myrcene $$ 7-Methyl-3-methylene-1,6-octadiene $$ 7-Methyl-3-methyleneoctadie

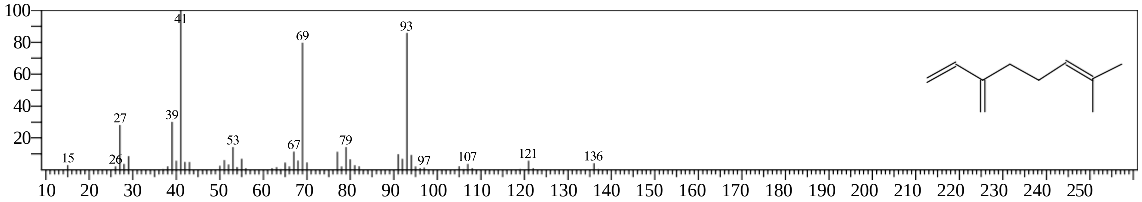

Hit#:2 Entry:41288 Library:NIST107.LIB
SI:89 Formula:C10H17Br CAS:35719-26-7 MolWeight:216 RetIndex:0
CompName:Geranyl bromide $$

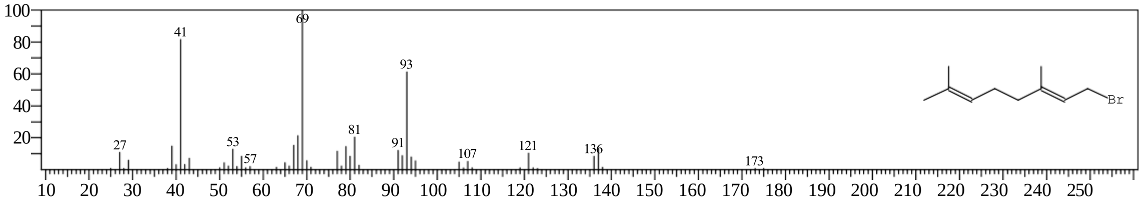

Hit#:3 Entry:8752 Library:NIST107.LIB
SI:87 Formula:C10H16 CAS:127-91-3 MolWeight:136 RetIndex:0
CompName:.beta.-Pinene $$ Bicyclo[3.1.1]heptane, 6,6-dimethyl-2-methylene- $$ 2(10)-Pinene $$ Nopinen $$ Nopinene $$ Pseudopinen $$ Pseudopinene

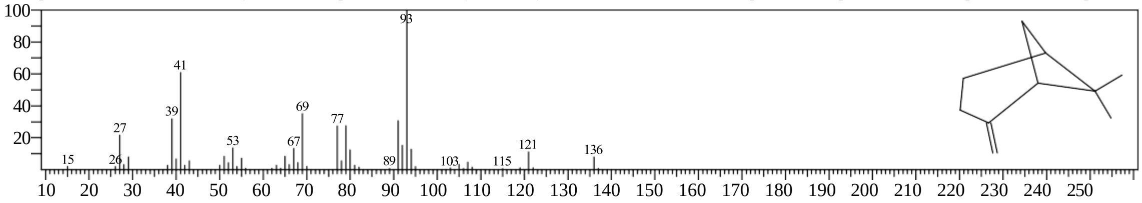

Hit#:4 Entry:34545 Library:NIST107.LIB
SI:87 Formula:C10H16S2 CAS:73188-23-5 MolWeight:200 RetIndex:0
CompName:4-(4-Methylpent-3-enyl)-3,6-dihydro-1,2-dithiin $$

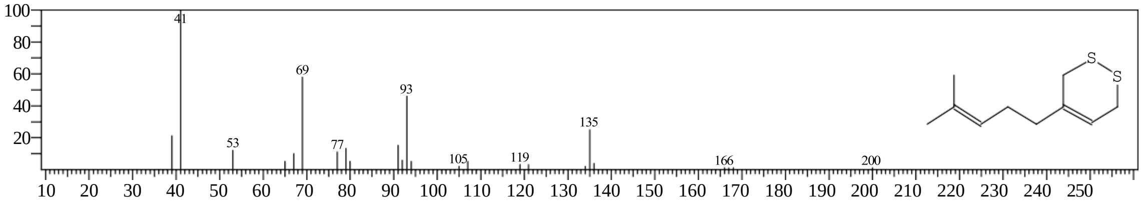

Hit#:5 Entry:8670 Library:NIST107.LIB
SI:85 Formula:C10H16 CAS:18172-67-3 MolWeight:136 RetIndex:0
CompName:Bicyclo[3.1.1]heptane, 6,6-dimethyl-2-methylene-, (1S)- $$ 2(10)-Pinene, (1S,5S)-(-)- $$ (-)-.beta.-Pinene $$ (-)-2(10)-Pinene $$ L-.beta.-pinen

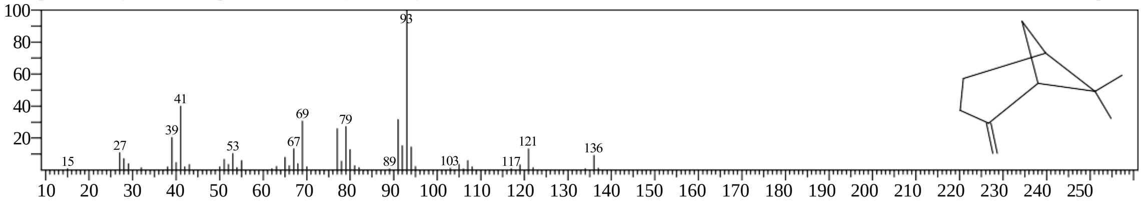

<< Target >>
Line#:17 R.Time:5.935(Scan#:1188) MassPeaks:59
RawMode:Averaged 5.930-5.940(1187-1189) BasePeak:93.20(196909)
BG Mode:Calc. from Peak

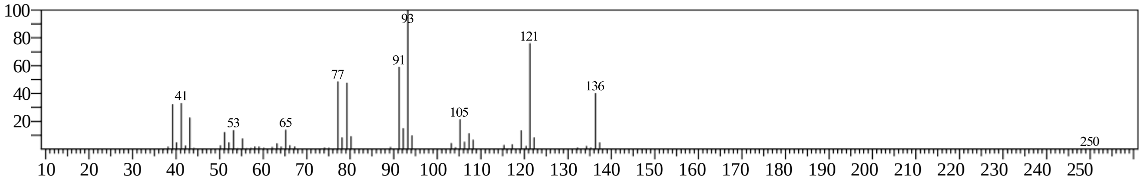

Hit#:1 Entry:8778 Library:NIST107.LIB
SI:95 Formula:C10H16 CAS:99805-90-0 MolWeight:136 RetIndex:0
CompName:Cyclohexene, 4-methyl-3-(1-methylethylidene)- $$

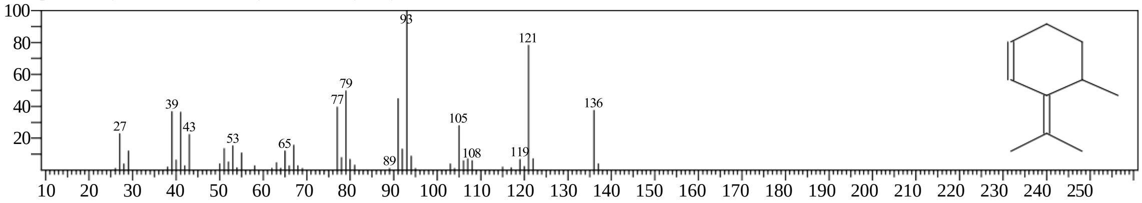

Hit#:2 Entry:8736 Library:NIST107.LIB
SI:94 Formula:C10H16 CAS:586-62-9 MolWeight:136 RetIndex:0
CompName:Cyclohexene, 1-methyl-4-(1-methylethylidene)- $$ p-Mentha-1,4(8)-diene $$ Terpinolen $$ Terpinolene $$ UN 2541 $$

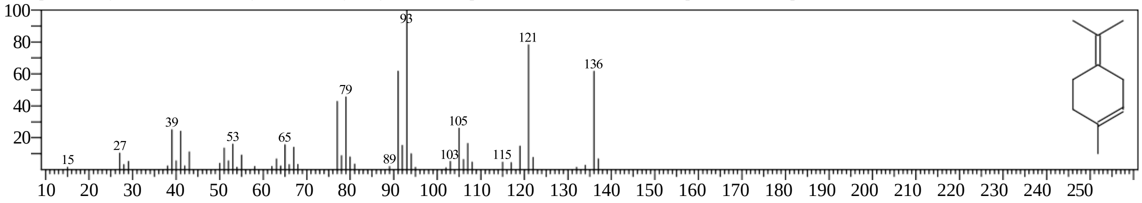

Hit#:3 Entry:8642 Library:NIST107.LIB
SI:94 Formula:C10H16 CAS:0-00-0 MolWeight:136 RetIndex:0
CompName:(+)-2-Caren $$

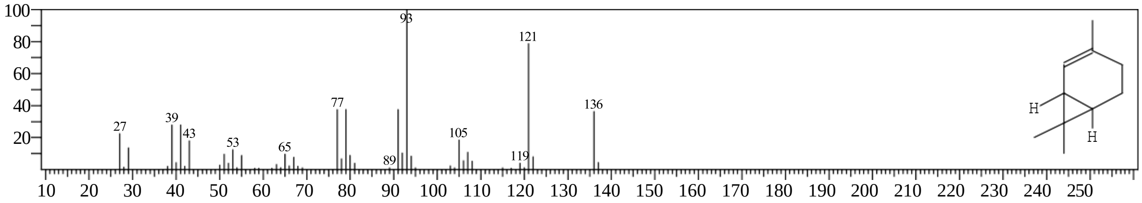

Hit#:4 Entry:8689 Library:NIST107.LIB
SI:93 Formula:C10H16 CAS:99-86-5 MolWeight:136 RetIndex:0
CompName:1,3-Cyclohexadiene, 1-methyl-4-(1-methylethyl)- $$ .alpha.-Terpinen $$ .alpha.-Terpinene $$ p-Mentha-1,3-diene $$ Terpilene $$ 1-Isopropyl-

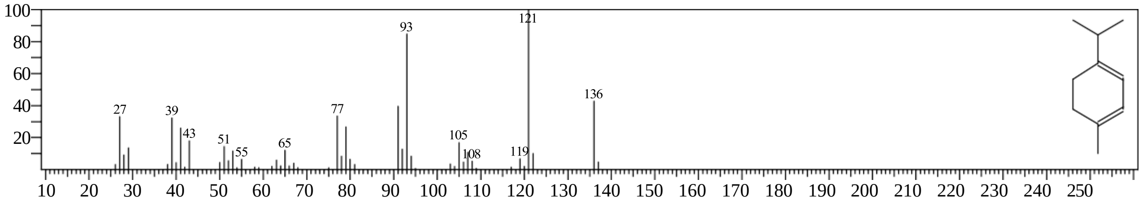

Hit#:5 Entry:8727 Library:NIST107.LIB
SI:92 Formula:C10H16 CAS:554-61-0 MolWeight:136 RetIndex:0
CompName:Bicyclo[4.1.0]hept-2-ene, 3,7,7-trimethyl- $$ 2-Carene $$

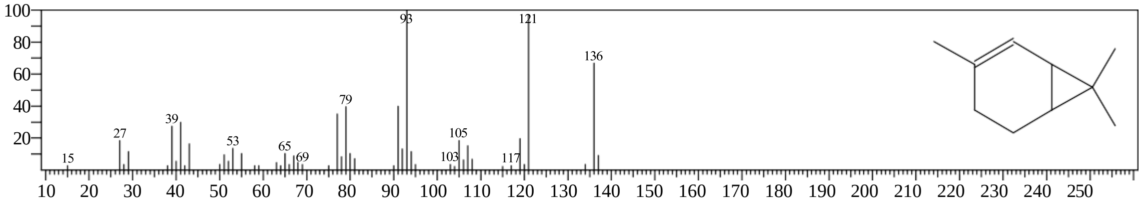

<< Target >>
Line#:18 R.Time:5.985(Scan#:1198) MassPeaks:63
RawMode:Averaged 5.980-5.990(1197-1199) BasePeak:91.20(18865)
BG Mode:Calc. from Peak

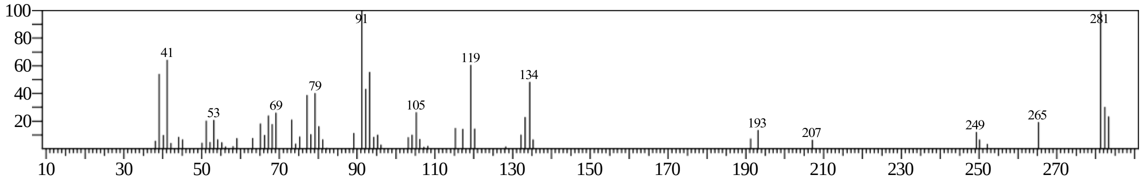

Hit#:1 Entry:18347 Library:NIST107.LIB
SI:75 Formula:C12H18 CAS:51567-09-0 MolWeight:162 RetIndex:0
CompName:Dispiro[2.0.2.5]undecane, 8-methylene- $$

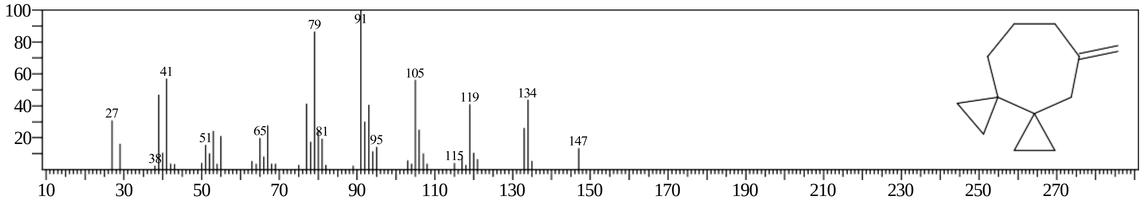

Hit#:2 Entry:8126 Library:NIST107.LIB
SI:75 Formula:C10H14 CAS:934-02-1 MolWeight:134 RetIndex:0
CompName:Bicyclopentyl-1,1'-diene $$

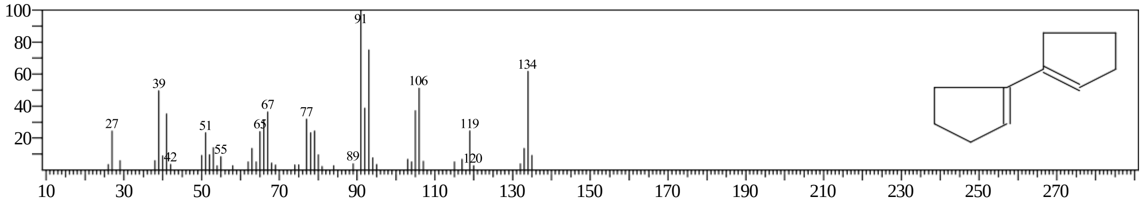

Hit#:3 Entry:18346 Library:NIST107.LIB
SI:75 Formula:C12H18 CAS:51567-08-9 MolWeight:162 RetIndex:0
CompName:Dispiro[2.1.2.4]undecane, 8-methylene- $$

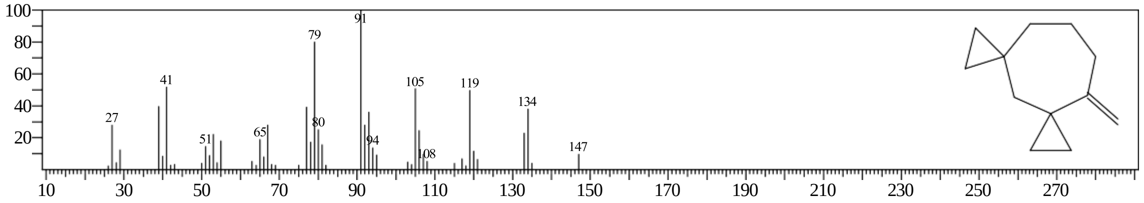

Hit#:4 Entry:8143 Library:NIST107.LIB
SI:74 Formula:C10H14 CAS:49826-54-2 MolWeight:134 RetIndex:0
CompName:Bicyclo[3.2.1]octane, 2,3-bis(methylene)- $$

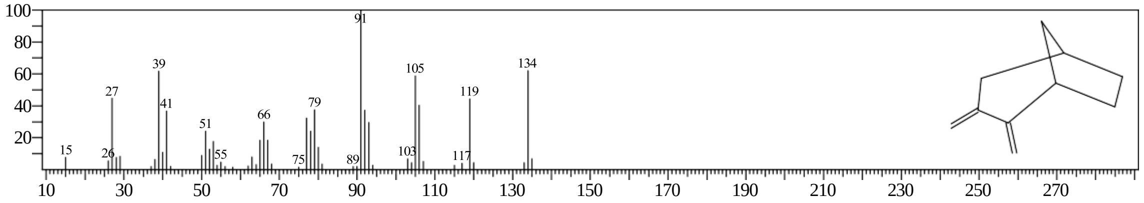

Hit#:5 Entry:8114 Library:NIST107.LIB
SI:74 Formula:C10H14 CAS:68284-24-2 MolWeight:134 RetIndex:0
CompName:Cycloheptane, 1,3,5-tris(methylene)- $$

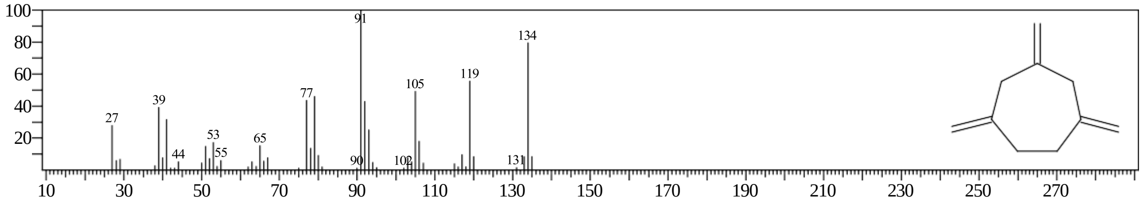

<< Target >>
Line#:19 R.Time:6.125(Scan#:1226) MassPeaks:100
RawMode:Averaged 6.120-6.130(1225-1227) BasePeak:93.15(8329374)
BG Mode:Calc. from Peak

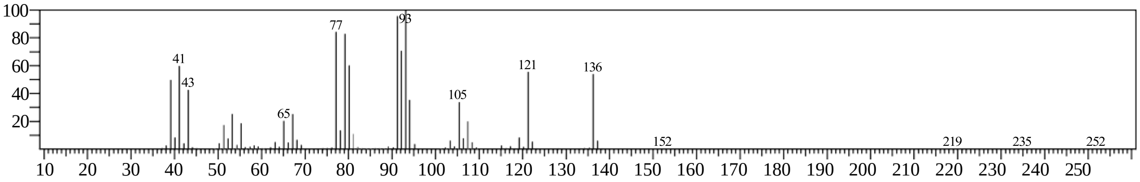

Hit#:1 Entry:8712 Library:NIST107.LIB
SI:91 Formula:C10H16 CAS:28935-76-4 MolWeight:136 RetIndex:0
CompName:3-Octen-5-yne, 2,7-dimethyl-, (Z)- $$

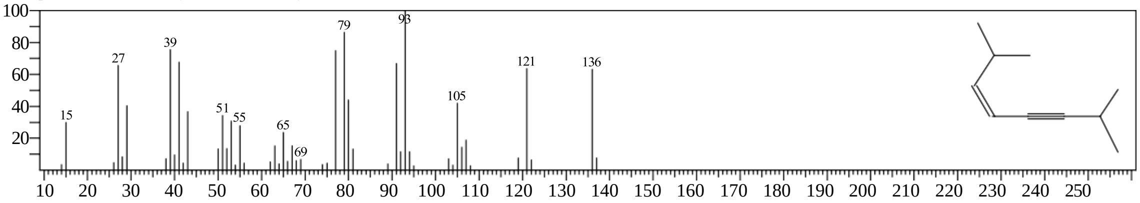

Hit#:2 Entry:8726 Library:NIST107.LIB
SI:91 Formula:C10H16 CAS:55956-33-7 MolWeight:136 RetIndex:0
CompName:3-Octen-5-yne, 2,7-dimethyl-, (E)- $$

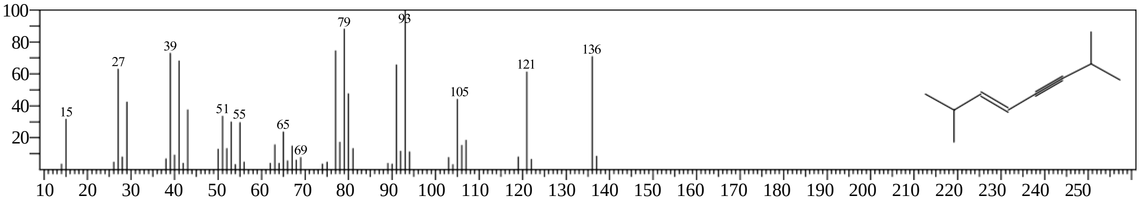

Hit#:3 Entry:8742 Library:NIST107.LIB
SI:88 Formula:C10H16 CAS:24648-33-7 MolWeight:136 RetIndex:0
CompName:2,4,6-Trimethyl-1,3,6-heptatriene $$

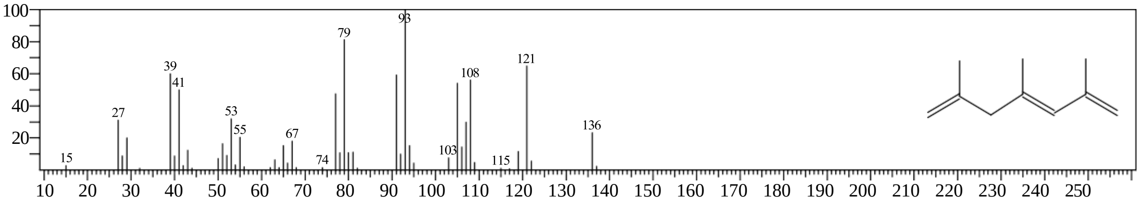

Hit#:4 Entry:8695 Library:NIST107.LIB
SI:87 Formula:C10H16 CAS:471-84-1 MolWeight:136 RetIndex:0
CompName:Bicyclo[2.2.1]heptane, 7,7-dimethyl-2-methylene- $$ Norbornane, 7,7-dimethyl-2-methylene- $$ .alpha.-Fenchene $$ Fenchene $$ 7,7-Dimethy

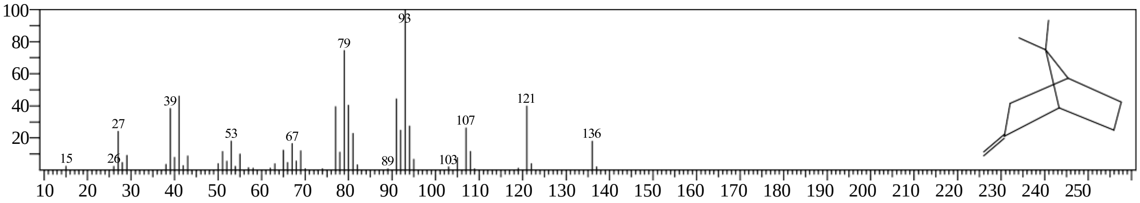

Hit#:5 Entry:8778 Library:NIST107.LIB
SI:87 Formula:C10H16 CAS:99805-90-0 MolWeight:136 RetIndex:0
CompName:Cyclohexene, 4-methyl-3-(1-methylethylidene)- $$

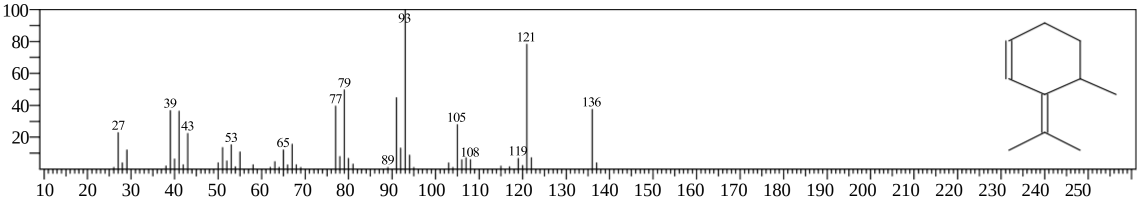

<< Target >>
Line#:20 R.Time:6.190(Scan#:1239) MassPeaks:113
RawMode:Averaged 6.185-6.195(1238-1240) BasePeak:43.15(7679901)
BG Mode:Calc. from Peak

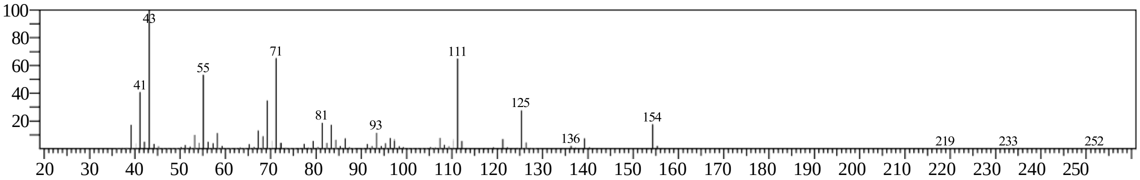

Hit#:1 Entry:15234 Library:NIST107.LIB
SI:96 Formula:C10H18O CAS:470-67-7 MolWeight:154 RetIndex:0
CompName:7-Oxabicyclo[2.2.1]heptane, 1-methyl-4-(1-methylethyl)- $$ p-Menthane, 1,4-epoxy $$ Isocineole $$ 1,4-Cineol $$ 1,4-Cineole $$ 1,4-Epoxy-

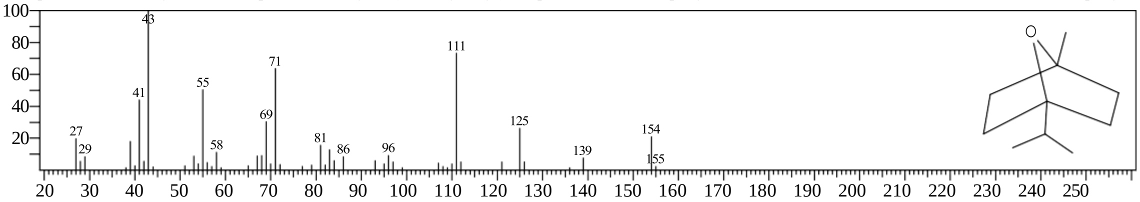

Hit#:2 Entry:15262 Library:NIST107.LIB
SI:86 Formula:C10H18O CAS:32064-70-3 MolWeight:154 RetIndex:0
CompName:2-Heptanone, 3-propylidene- $$

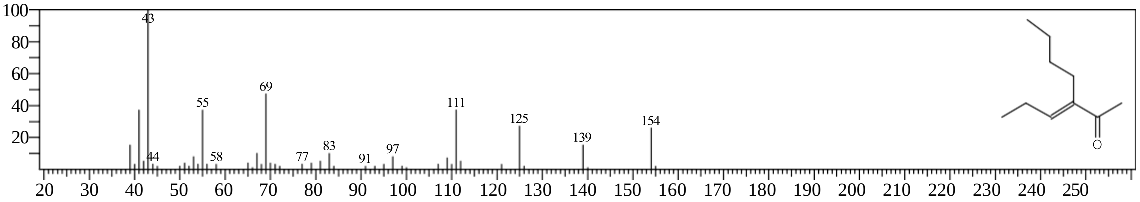

Hit#:3 Entry:15186 Library:NIST107.LIB
SI:84 Formula:C10H18O CAS:7299-40-3 MolWeight:154 RetIndex:0
CompName:cis-.beta.-Terpineol $$ p-Menth-8-en-1-ol, stereoisomer $$ Cyclohexanol, 1-methyl-4-(1-methylethenyl)-, trans- $$

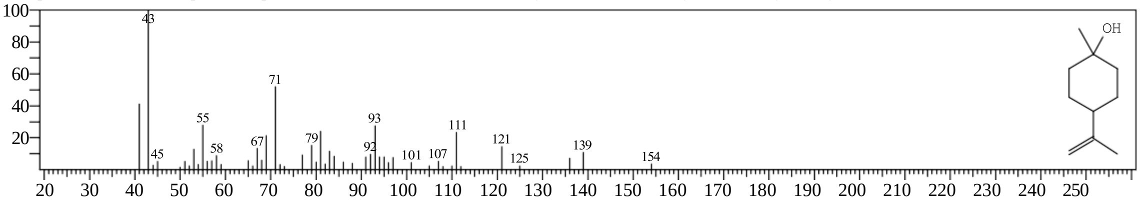

Hit#:4 Entry:15193 Library:NIST107.LIB
SI:84 Formula:C10H18O CAS:7299-41-4 MolWeight:154 RetIndex:0
CompName:Terpineol, Z-.beta.- $$

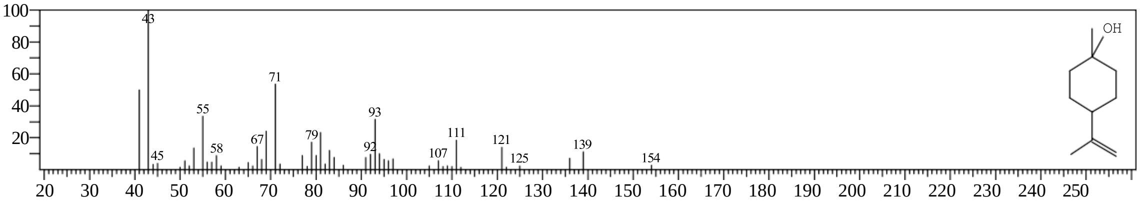

Hit#:5 Entry:15278 Library:NIST107.LIB
SI:84 Formula:C10H18O CAS:35194-33-3 MolWeight:154 RetIndex:0
CompName:7-Decen-2-one $$

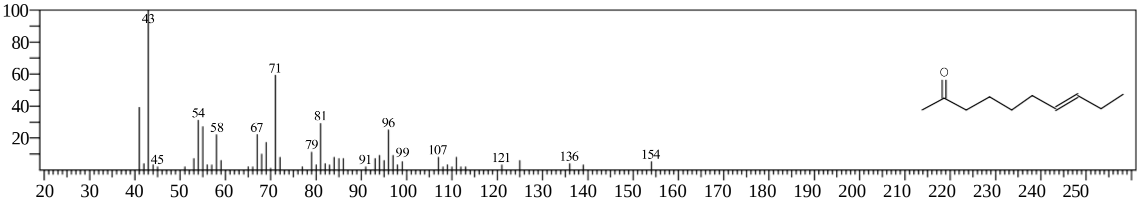

<< Target >>
Line#:21 R.Time:6.350(Scan#:1271) MassPeaks:61
RawMode:Averaged 6.345-6.355(1270-1272) BasePeak:119.25(1890092)
BG Mode:Calc. from Peak

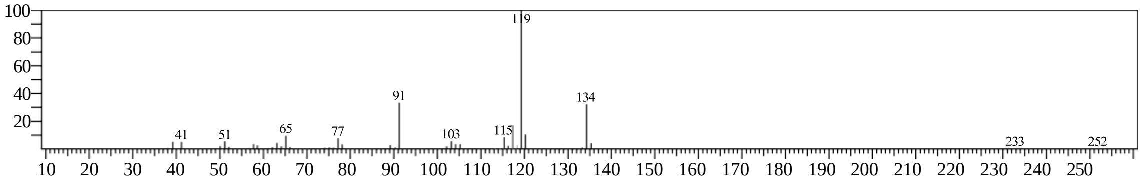

Hit#:1 Entry:8141 Library:NIST107.LIB
SI:92 Formula:C10H14 CAS:527-84-4 MolWeight:134 RetIndex:0
CompName:Benzene, 1-methyl-2-(1-methylethyl)- $$ o-Cymene $$ o-Cymol $$ o-Isopropyltoluene $$ 1-Isopropyl-2-methylbenzene $$ 1-Methyl-2-isoprop

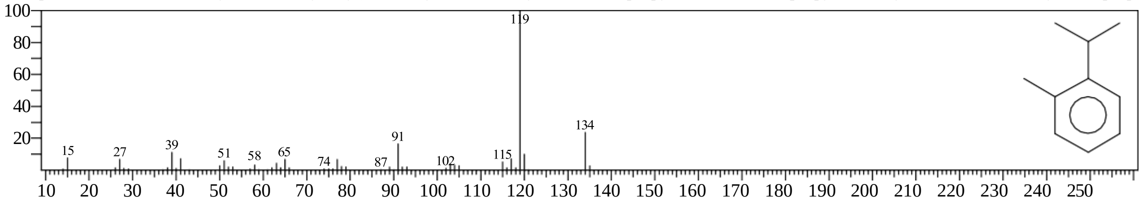

Hit#:2 Entry:8138 Library:NIST107.LIB
SI:91 Formula:C10H14 CAS:535-77-3 MolWeight:134 RetIndex:0
CompName:Benzene, 1-methyl-3-(1-methylethyl)- $$ m-Cymene $$ .beta.-Cymene $$ m-Cymol $$ m-Isopropyltoluene $$ m-Methylisopropylbenzene $$ 1

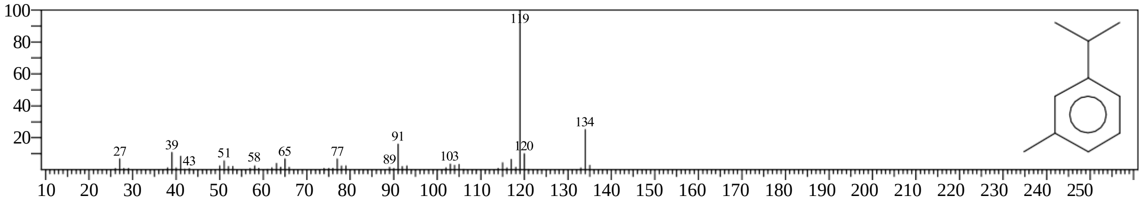

Hit#:3 Entry:8105 Library:NIST107.LIB
SI:91 Formula:C10H14 CAS:99-87-6 MolWeight:134 RetIndex:0
CompName:Benzene, 1-methyl-4-(1-methylethyl)- $$ p-Cymene $$ p-Cimene $$ p-Cymol $$ p-Isopropyltoluene $$ p-Methylisopropylbenzene $$ Campho

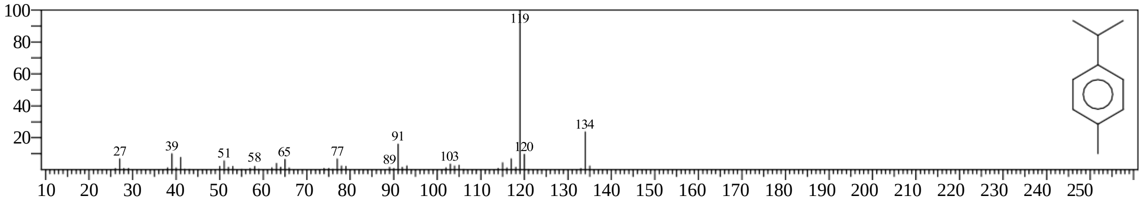

Hit#:4 Entry:8103 Library:NIST107.LIB
SI:91 Formula:C10H14 CAS:2870-04-4 MolWeight:134 RetIndex:0
CompName:Benzene, 2-ethyl-1,3-dimethyl- $$ m-Xylene, 2-ethyl- $$ 1,3-Dimethyl-2-ethylbenzene $$ 2-Ethyl-1,3-dimethylbenzene $$

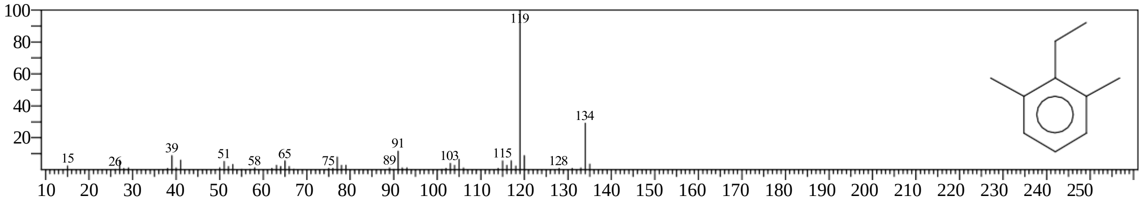

Hit#:5 Entry:8117 Library:NIST107.LIB
SI:90 Formula:C10H14 CAS:933-98-2 MolWeight:134 RetIndex:0
CompName:Benzene, 1-ethyl-2,3-dimethyl- $$ o-Xylene, 3-ethyl- $$ 1-Ethyl-2,3-dimethylbenzene $$ 1,2-Dimethyl-3-ethylbenzene $$ 3-Ethyl-o-xylene $$

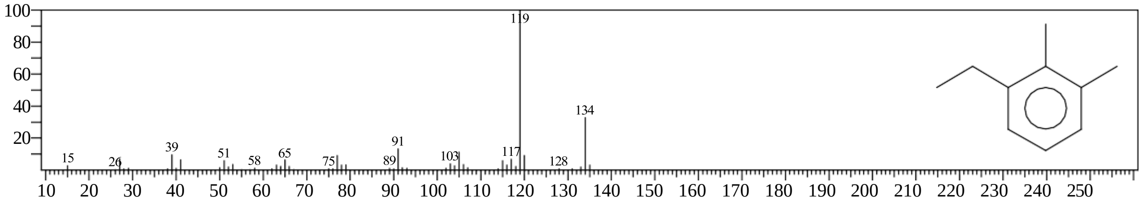

<< Target >>
Line#:22 R.Time:6.440(Scan#:1289) MassPeaks:78
RawMode:Averaged 6.435-6.445(1288-1290) BasePeak:67.15(7087671)
BG Mode:Calc. from Peak

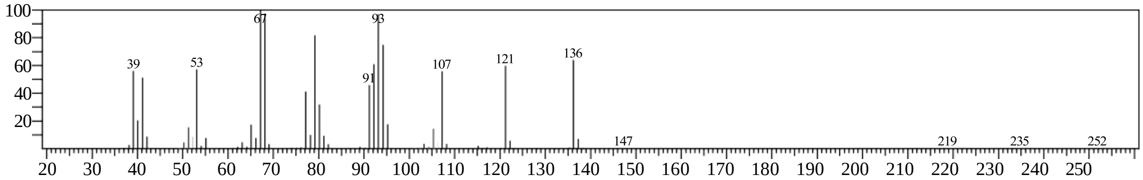

Hit#:1 Entry:8641 Library:NIST107.LIB
SI:87 Formula:C10H16 CAS:0-00-0 MolWeight:136 RetIndex:0
CompName:Isolimonene $$

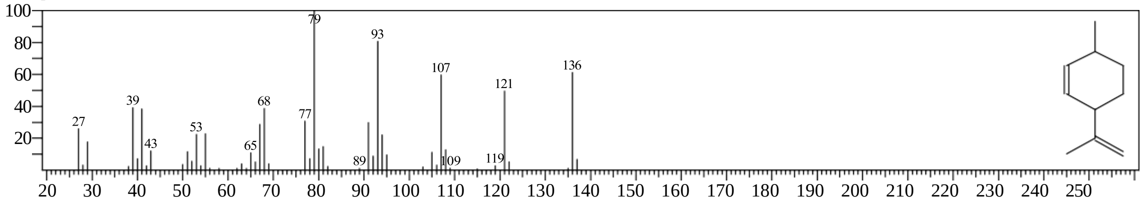

Hit#:2 Entry:8772 Library:NIST107.LIB
SI:87 Formula:C10H16 CAS:15402-94-5 MolWeight:136 RetIndex:0
CompName:Cycloheptene, 5-ethylidene-1-methyl- $$

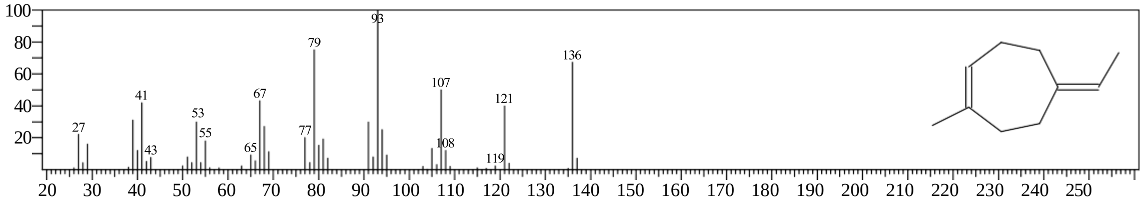

Hit#:3 Entry:8645 Library:NIST107.LIB
SI:87 Formula:C10H16 CAS:0-00-0 MolWeight:136 RetIndex:0
CompName:Bicyclo[4.3.0]nonane, 3-methylene- $$

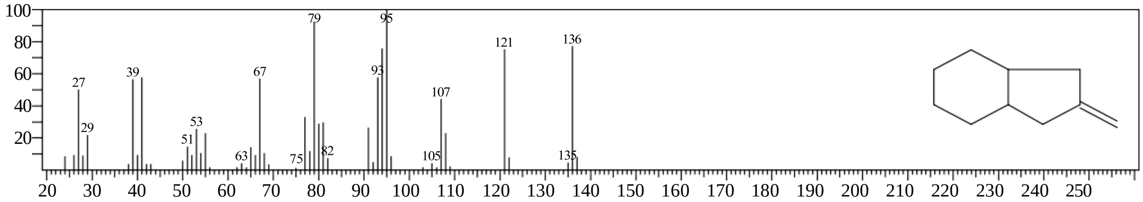

Hit#:4 Entry:8755 Library:NIST107.LIB
SI:86 Formula:C10H16 CAS:53282-47-6 MolWeight:136 RetIndex:0
CompName:Bicyclo[4.1.0]heptane, 7-(1-methylethylidene)- $$

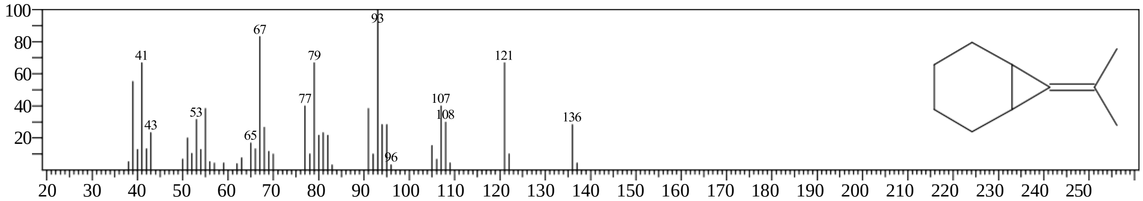

Hit#:5 Entry:8681 Library:NIST107.LIB
SI:85 Formula:C10H16 CAS:5113-87-1 MolWeight:136 RetIndex:0
CompName:Cyclohexene, 3-methyl-6-(1-methylethenyl)-, (3R-trans)- $$ p-Mentha-2,8-diene, (1R,4R)-(+)- $$ (+)-(1R,4R)-trans-Isolimonene $$ (+)-trans-I

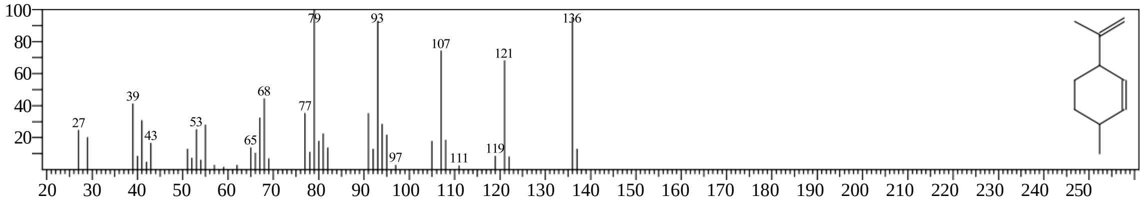

<< Target >>
Line#:23 R.Time:6.465(Scan#:1294) MassPeaks:91
RawMode:Averaged 6.460-6.470(1293-1295) BasePeak:43.10(3699331)
BG Mode:Calc. from Peak

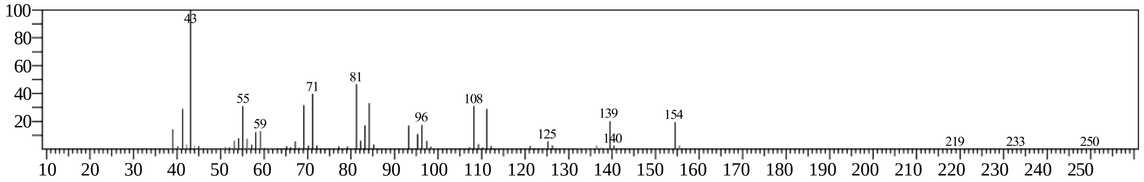

Hit#:1 Entry:15148 Library:NIST107.LIB
SI:89 Formula:C10H18O CAS:470-82-6 MolWeight:154 RetIndex:0
CompName:Eucalyptol $$ Cineole $$ 2-Oxabicyclo[2.2.2]octane, 1,3,3-trimethyl- $$ p-Menthane, 1,8-epoxy- $$ p-Cineole $$ Cajeputol $$ Cucalyptol $$

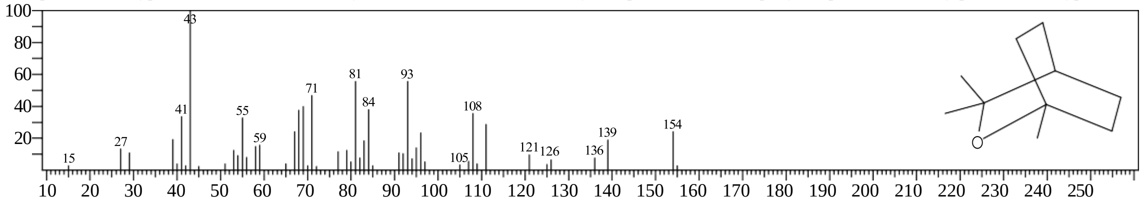

Hit#:2 Entry:15234 Library:NIST107.LIB
SI:85 Formula:C10H18O CAS:470-67-7 MolWeight:154 RetIndex:0
CompName:7-Oxabicyclo[2.2.1]heptane, 1-methyl-4-(1-methylethyl)- $$ p-Menthane, 1,4-epoxy $$ Isocineole $$ 1,4-Cineol $$ 1,4-Cineole $$ 1,4-Epoxy-

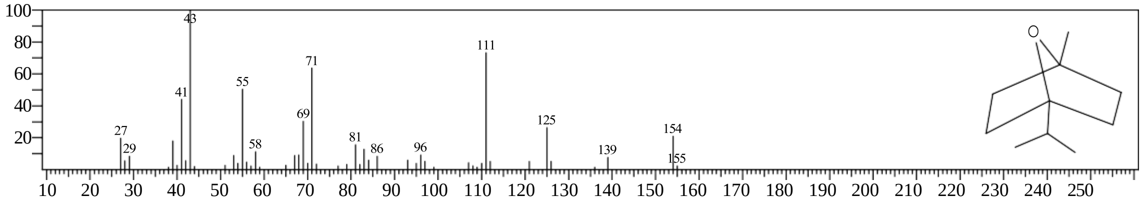

Hit#:3 Entry:54570 Library:NIST107.LIB
SI:84 Formula:C12H17F3O2 CAS:0-00-0 MolWeight:250 RetIndex:0
CompName:Trifluoroacetyl-.alpha.-terpineol $$

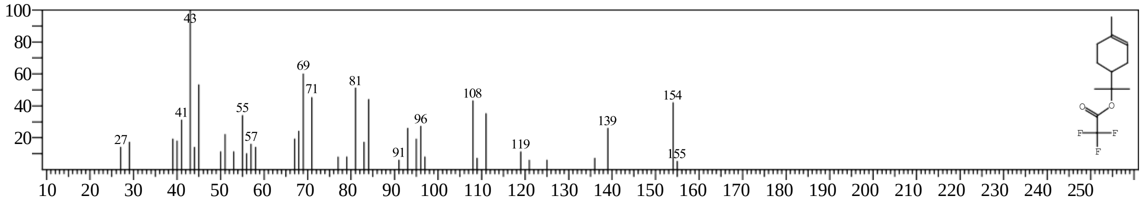

Hit#:4 Entry:22751 Library:NIST107.LIB
SI:83 Formula:C10H20O2 CAS:80-53-5 MolWeight:172 RetIndex:0
CompName:Cyclohexanemethanol, 4-hydroxy-.alpha.,.alpha.,4-trimethyl- $$ p-Menthane-1,8-diol $$ Terpin $$ 1,8-Terpin $$

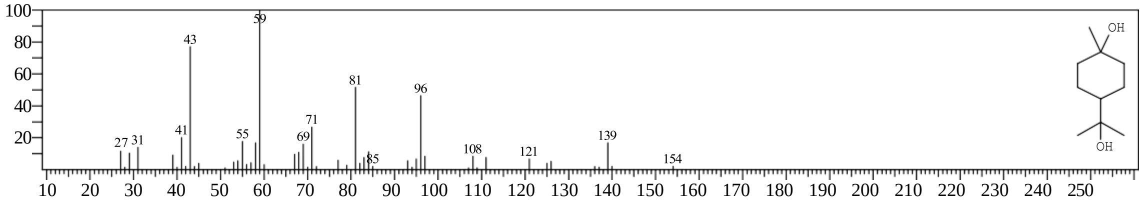

Hit#:5 Entry:40660 Library:NIST107.LIB
SI:82 Formula:C12H22O3 CAS:58315-86-9 MolWeight:214 RetIndex:0
CompName:3-Acetoxy-p-menthan-1-ol $$

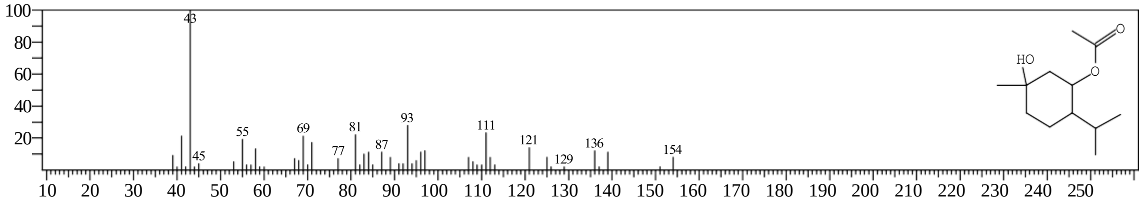

<< Target >>
Line#:24 R.Time:7.080(Scan#:1417) MassPeaks:47
RawMode:Averaged 7.075-7.085(1416-1418) BasePeak:79.15(34387)
BG Mode:Calc. from Peak

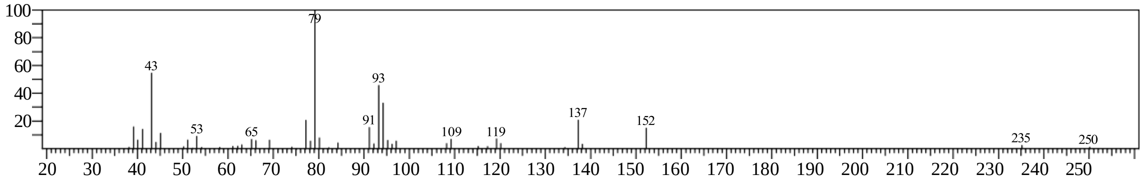

Hit#:1 Entry:8540 Library:NIST107.LIB
SI:77 Formula:C9H12O CAS:0-00-0 MolWeight:136 RetIndex:0
CompName:6-Propenylbicyclo[3.1.0]hexan-2-one $$

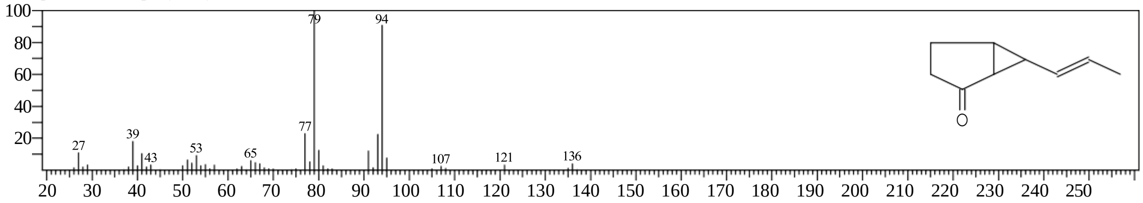

Hit#:2 Entry:1178 Library:NIST107.LIB
SI:77 Formula:C7H10 CAS:59219-48-6 MolWeight:94 RetIndex:0
CompName:Cyclopentane, 1,3-bis(methylene)- $$

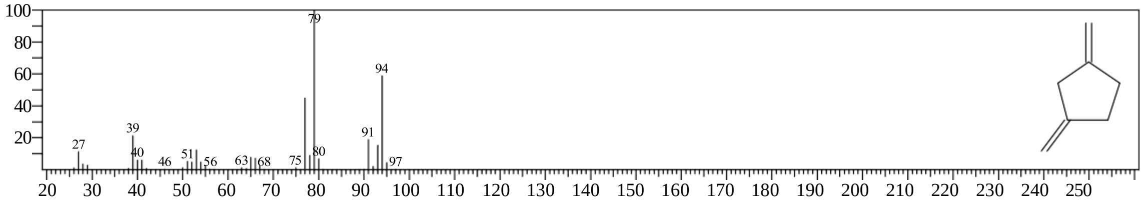

Hit#:3 Entry:1177 Library:NIST107.LIB
SI:76 Formula:C7H10 CAS:24587-27-7 MolWeight:94 RetIndex:0
CompName:1,3,5-Hexatriene, 3-methyl-, (Z)- $$

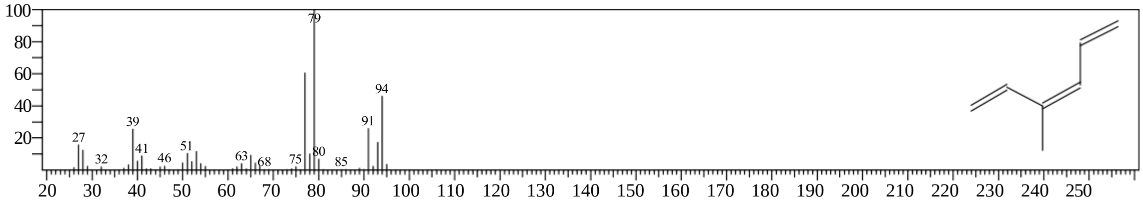

Hit#:4 Entry:1202 Library:NIST107.LIB
SI:76 Formula:C7H10 CAS:4125-18-2 MolWeight:94 RetIndex:0
CompName:1,3-Cyclopentadiene, 5,5-dimethyl- $$ 5,5-Dimethylcyclopentadiene $$

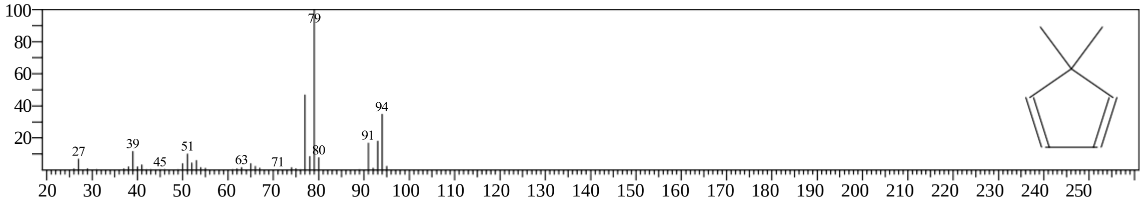

Hit#:5 Entry:1188 Library:NIST107.LIB
SI:76 Formula:C7H10 CAS:4054-38-0 MolWeight:94 RetIndex:0
CompName:1,3-Cycloheptadiene $$

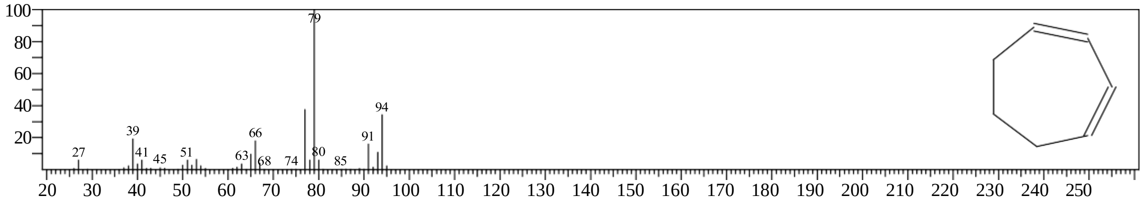

<< Target >>
Line#:25 R.Time:7.135(Scan#:1428) MassPeaks:57
RawMode:Averaged 7.130-7.140(1427-1429) BasePeak:43.10(69312)
BG Mode:Calc. from Peak

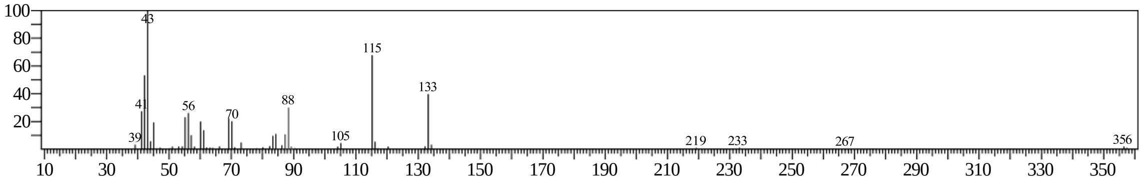

Hit#:1 Entry:17203 Library:NIST107.LIB
SI:84 Formula:C7H12O4 CAS:105-53-3 MolWeight:160 RetIndex:0
CompName:Propanedioic acid, diethyl ester $$ Malonic acid, diethyl ester $$ Carbethoxyacetic ester $$ Dicarbethoxymethane $$ Diethyl malonate $$ Dieth

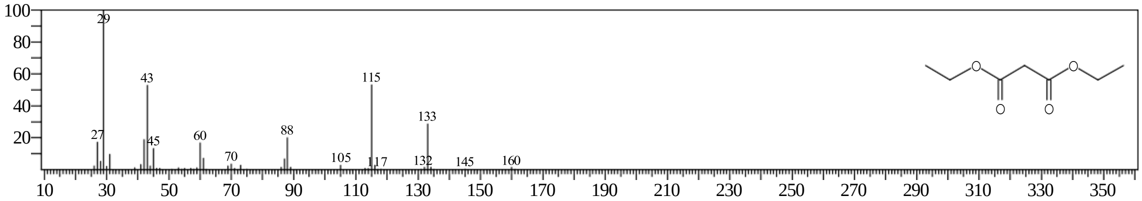

Hit#:2 Entry:29290 Library:NIST107.LIB
SI:75 Formula:C9H16O4 CAS:93117-74-9 MolWeight:188 RetIndex:0
CompName:tert-Butyl ethyl malonate $$

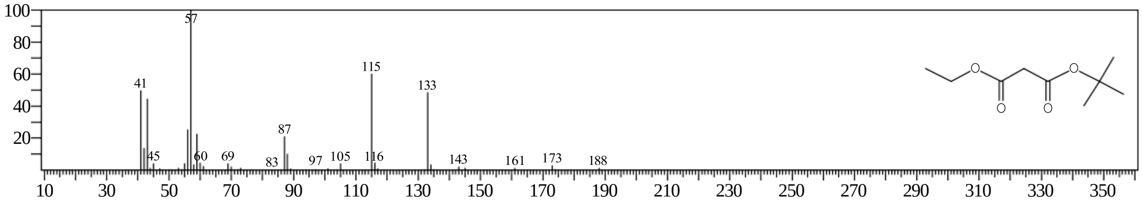

Hit#:3 Entry:23653 Library:NIST107.LIB
SI:74 Formula:C6H9NO5 CAS:0-00-0 MolWeight:175 RetIndex:0
CompName:4-Nitro-3-oxobutyric acid, ethyl ester $$

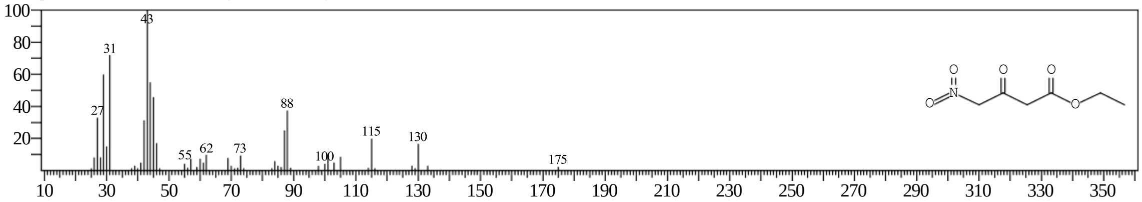

Hit#:4 Entry:7707 Library:NIST107.LIB
SI:73 Formula:C4H7NO4 CAS:56-84-8 MolWeight:133 RetIndex:0
CompName:Aspartic acid $$ L-Aspartic acid $$ Aspartic acid, L- $$ .alpha.-Aminosuccinic acid $$ (S)-Aspartic acid $$ (2S)-Aspartic acid $$ Asparagic aci

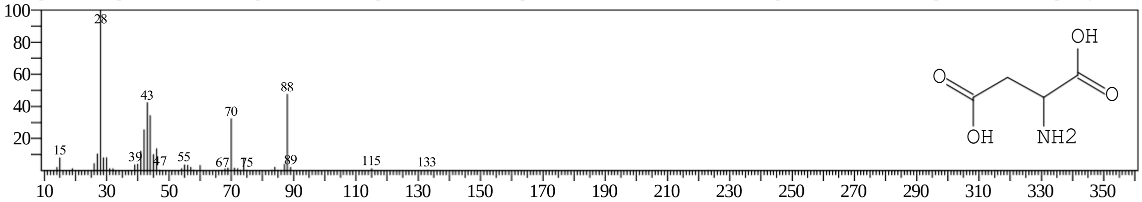

Hit#:5 Entry:29301 Library:NIST107.LIB
SI:73 Formula:C9H16O4 CAS:32864-38-3 MolWeight:188 RetIndex:0
CompName:t-Butylethyl malonate $$ tert-Butyl ethyl malonate $$ Propanedioic acid, 1,1-dimethylethyl ethyl ester $$

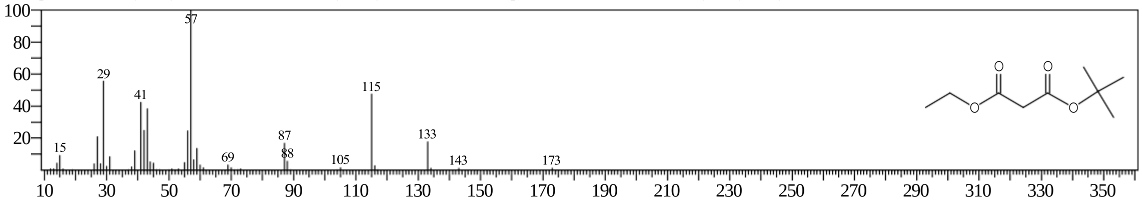

<< Target >>
Line#:26 R.Time:7.245(Scan#:1450) MassPeaks:76
RawMode:Averaged 7.240-7.250(1449-1451) BasePeak:132.30(62457)
BG Mode:Calc. from Peak

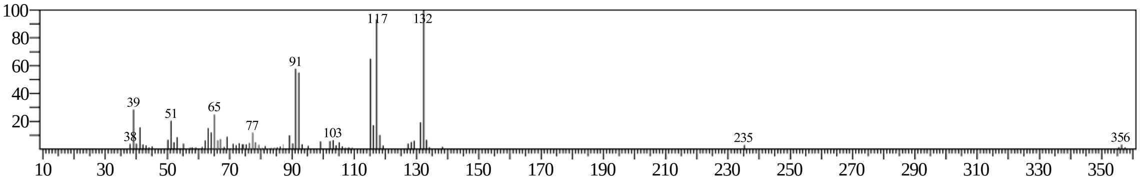

Hit#:1 Entry:7658 Library:NIST107.LIB
SI:91 Formula:C10H12 CAS:1195-32-0 MolWeight:132 RetIndex:0
CompName:Benzene, 1-methyl-4-(1-methylethenyl)- $$ Styrene, p,.alpha.-dimethyl- $$ .alpha.,p-Dimethylstyrene $$ .alpha.,4-Dimethylstyrene $$ p-Isoprop

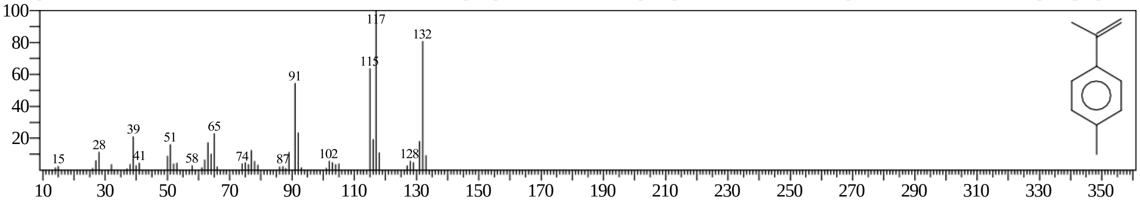

Hit#:2 Entry:7661 Library:NIST107.LIB
SI:89 Formula:C10H12 CAS:7399-49-7 MolWeight:132 RetIndex:0
CompName:o-Isopropenyltoluene $$ 1-Methyl-2-iso-propenylbenzene $$ Benzene, 1-methyl-2-(1-methylethenyl)- $$ o-Methyl-.alpha.-methylstyrene $$ o,.

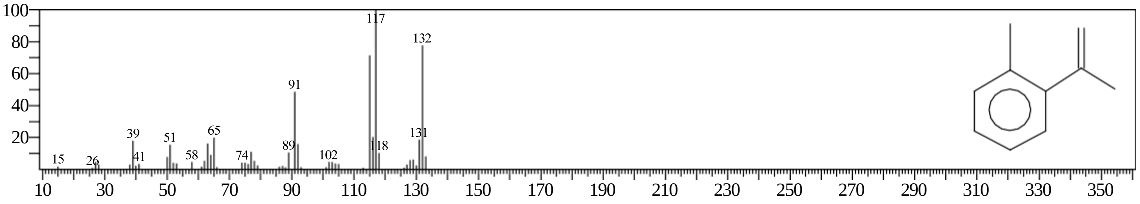

Hit#:3 Entry:7665 Library:NIST107.LIB
SI:86 Formula:C10H12 CAS:2234-20-0 MolWeight:132 RetIndex:0
CompName:2,4-Dimethylstyrene $$ Benzene, 1-ethenyl-2,4-dimethyl- $$ Styrene, 2,4-dimethyl- $$ 1-Vinyl-2,4-dimethylbenzene $$ 1,3-Dimethyl-4-ethenyl

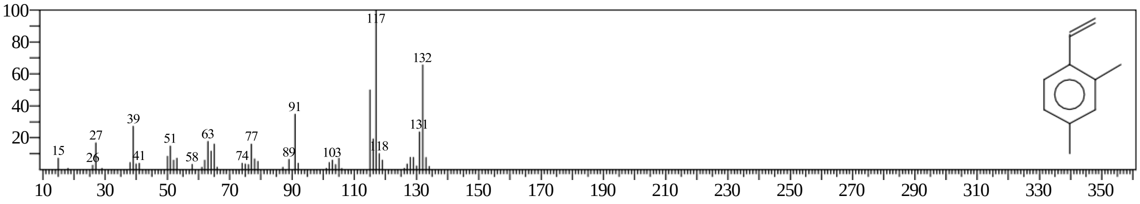

Hit#:4 Entry:7653 Library:NIST107.LIB
SI:86 Formula:C10H12 CAS:0-00-0 MolWeight:132 RetIndex:0
CompName:5,8-Dimethylenebicyclo[2.2.2]oct-2-ene $$

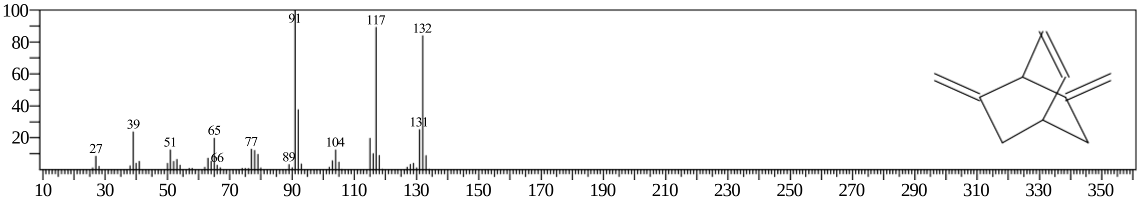

Hit#:5 Entry:7692 Library:NIST107.LIB
SI:86 Formula:C10H12 CAS:768-49-0 MolWeight:132 RetIndex:0
CompName:Benzene, (2-methyl-1-propenyl)- $$ Benzene, (2-methylpropenyl)- $$ .beta.-Methylisoallylbenzene $$ .beta.,.beta.-Dimethylstyrene $$ (2-Meth

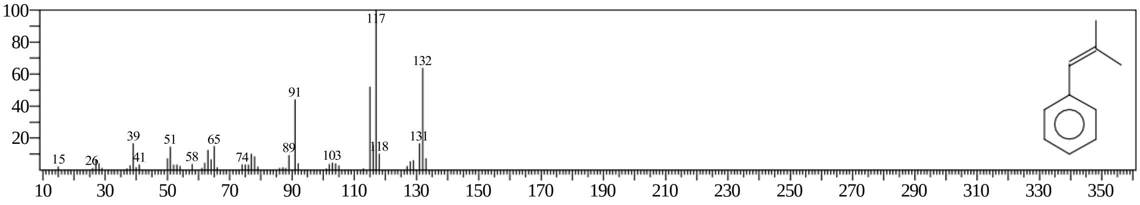

<< Target >>
Line#:27 R.Time:7.345(Scan#:1470) MassPeaks:97
RawMode:Averaged 7.340-7.350(1469-1471) BasePeak:117.20(107669)
BG Mode:Calc. from Peak

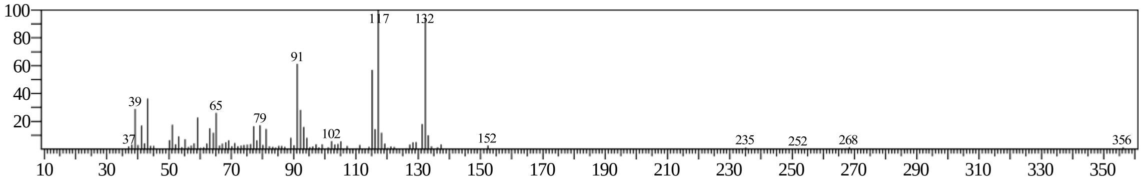

Hit#:1 Entry:7658 Library:NIST107.LIB
SI:85 Formula:C10H12 CAS:1195-32-0 MolWeight:132 RetIndex:0
CompName:Benzene, 1-methyl-4-(1-methylethenyl)- $$ Styrene, p,.alpha.-dimethyl- $$ .alpha.,p-Dimethylstyrene $$ .alpha.,4-Dimethylstyrene $$ p-Isoprop

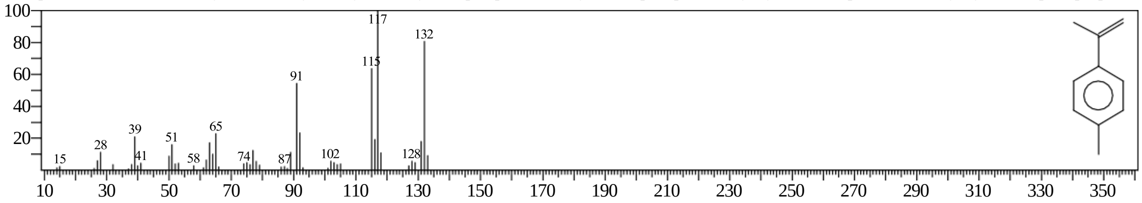

Hit#:2 Entry:7661 Library:NIST107.LIB
SI:84 Formula:C10H12 CAS:7399-49-7 MolWeight:132 RetIndex:0
CompName:o-Isopropenyltoluene $$ 1-Methyl-2-iso-propenylbenzene $$ Benzene, 1-methyl-2-(1-methylethenyl)- $$ o-Methyl-.alpha.-methylstyrene $$ o,.

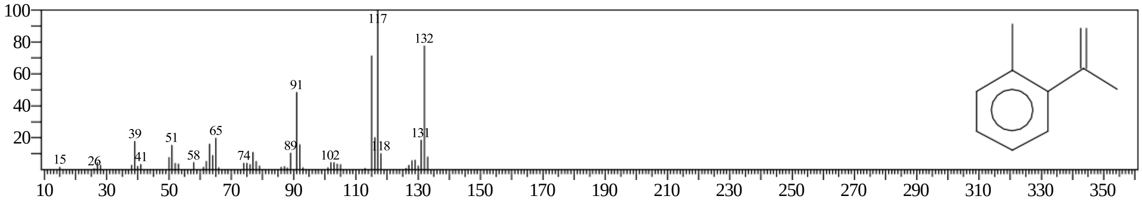

Hit#:3 Entry:17556 Library:NIST107.LIB
SI:83 Formula:C12H16 CAS:56282-43-0 MolWeight:160 RetIndex:0
CompName:Benzene, (1-cyclopropyl-1-methylethyl)- $$

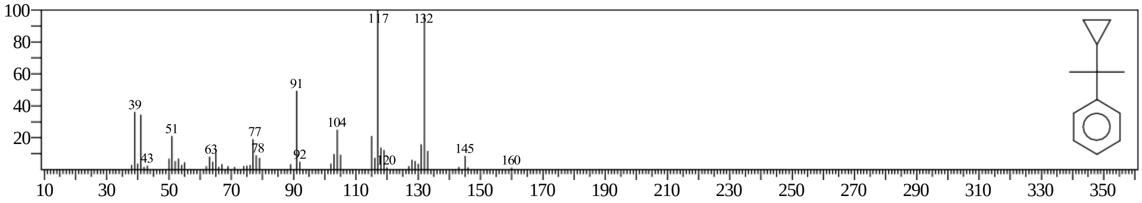

Hit#:4 Entry:7665 Library:NIST107.LIB
SI:82 Formula:C10H12 CAS:2234-20-0 MolWeight:132 RetIndex:0
CompName:2,4-Dimethylstyrene $$ Benzene, 1-ethenyl-2,4-dimethyl- $$ Styrene, 2,4-dimethyl- $$ 1-Vinyl-2,4-dimethylbenzene $$ 1,3-Dimethyl-4-ethenyl

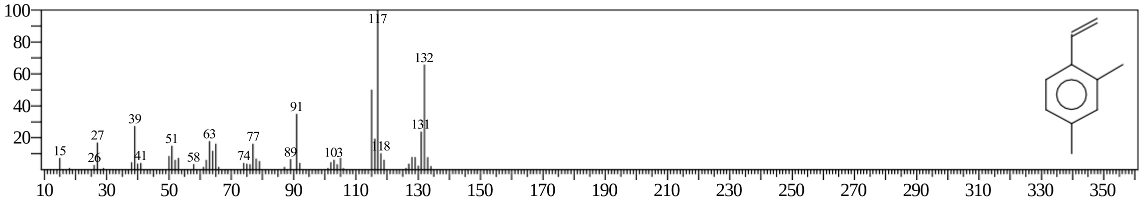

Hit#:5 Entry:7679 Library:NIST107.LIB
SI:81 Formula:C10H12 CAS:26444-18-8 MolWeight:132 RetIndex:0
CompName:Benzene, methyl(1-methylethenyl)- $$ Styrene, ar,.alpha.-dimethyl- $$ Isopropenyltoluene $$

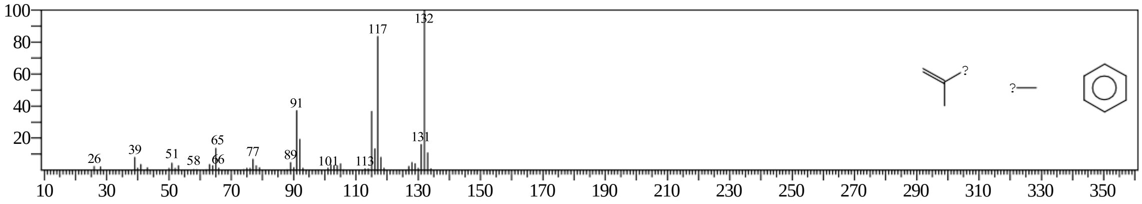

<< Target >>
Line#:28 R.Time:7.470(Scan#:1495) MassPeaks:74
RawMode:Averaged 7.465-7.475(1494-1496) BasePeak:108.25(75337)
BG Mode:Calc. from Peak

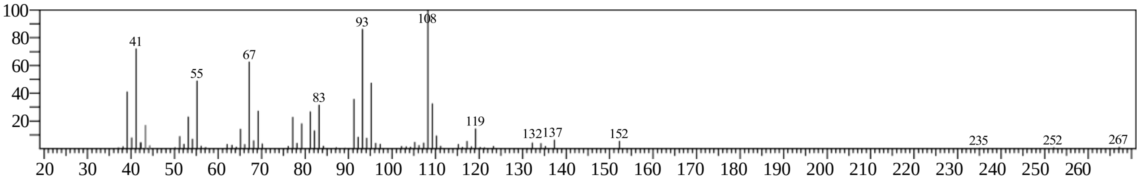

Hit#:1 Entry:21854 Library:NIST107.LIB
SI:86 Formula:C10H18O2 CAS:10359-41-8 MolWeight:170 RetIndex:0
CompName:Bicyclo[2.2.1]heptane-2,5-diol, 1,7,7-trimethyl-, (2-endo,5-exo)- $$

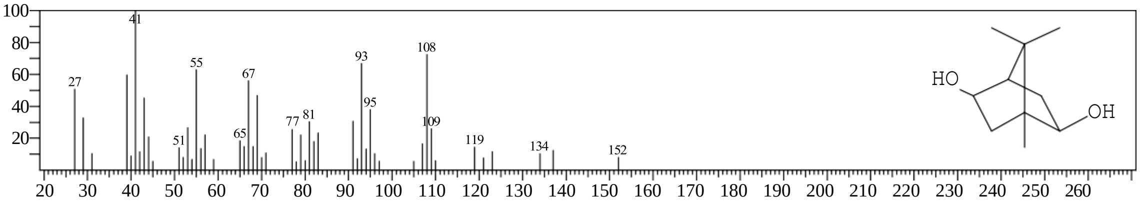

Hit#:2 Entry:14274 Library:NIST107.LIB
SI:83 Formula:C10H16O CAS:1686-14-2 MolWeight:152 RetIndex:0
CompName:3-Oxatricyclo[4.1.1.02,4]octane, 2,7,7-trimethyl- $$ Pinane, 2,3-epoxy- $$ .alpha.-Pinene epoxide $$ .alpha.-Pinene oxide $$ 2,3-Epoxypinane

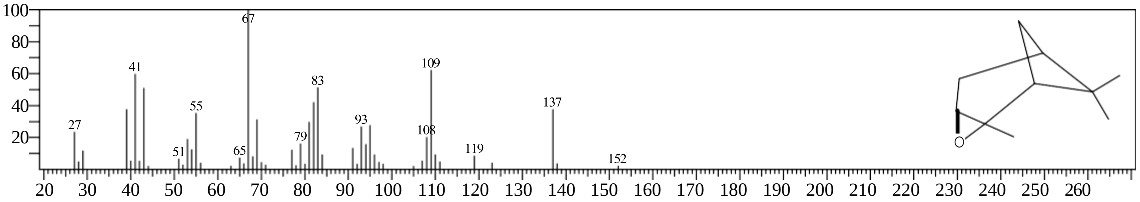

Hit#:3 Entry:14285 Library:NIST107.LIB
SI:83 Formula:C10H16O CAS:3767-44-0 MolWeight:152 RetIndex:0
CompName:Bicyclo[2.2.1]heptan-2-one, 5,5,6-trimethyl-, endo- $$ 2-Norbornanone, 5,5,6-trimethyl-, endo- $$

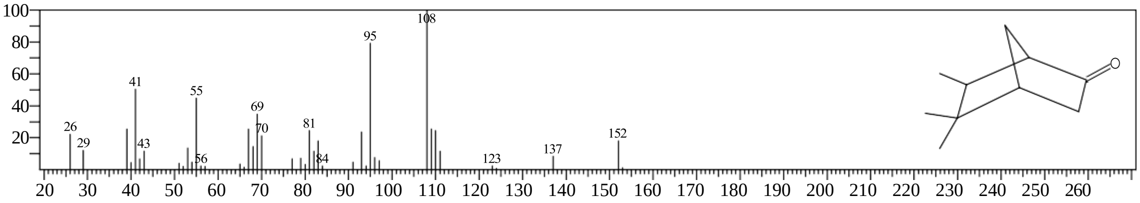

Hit#:4 Entry:14185 Library:NIST107.LIB
SI:83 Formula:C10H16O CAS:1753-35-1 MolWeight:152 RetIndex:0
CompName:(E)-3(10)-Caren-4-ol $$

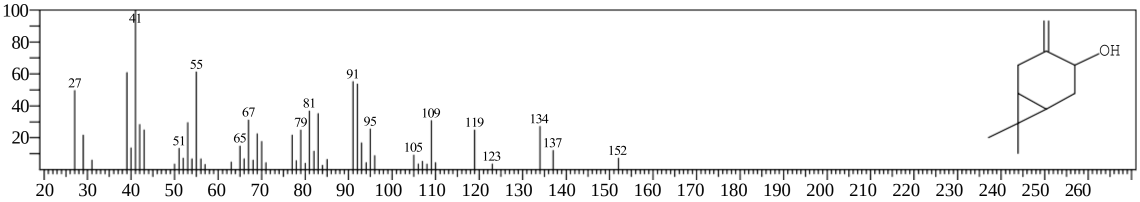

Hit#:5 Entry:14240 Library:NIST107.LIB
SI:83 Formula:C10H16O CAS:77954-13-3 MolWeight:152 RetIndex:0
CompName:Benzofuran, octahydro-6-methyl-3-methylene- $$

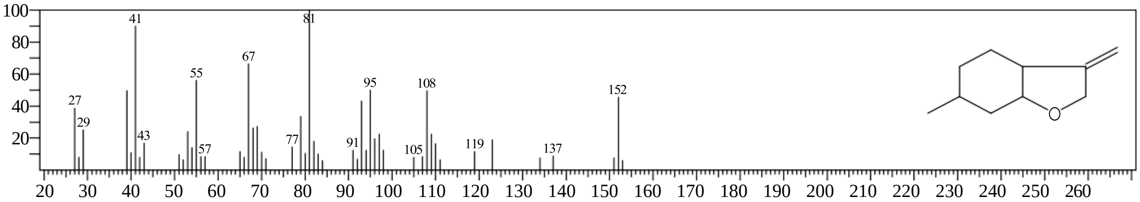

<< Target >>
Line#:29 R.Time:7.535(Scan#:1508) MassPeaks:89
RawMode:Averaged 7.530-7.540(1507-1509) BasePeak:119.25(225801)
BG Mode:Calc. from Peak

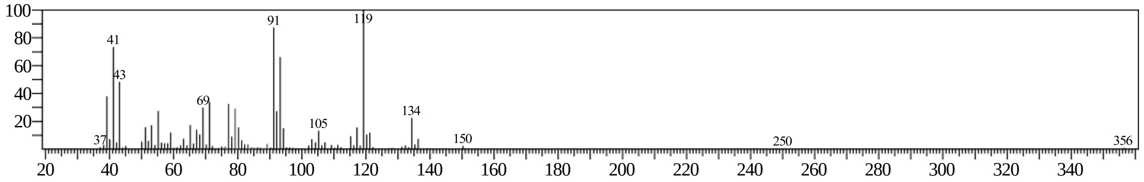

Hit#:1 Entry:31119 Library:NIST107.LIB
SI:81 Formula:C13H20O CAS:0-00-0 MolWeight:192 RetIndex:0
CompName:(+)-3-Carene, 2-(acetylmethyl)- $$

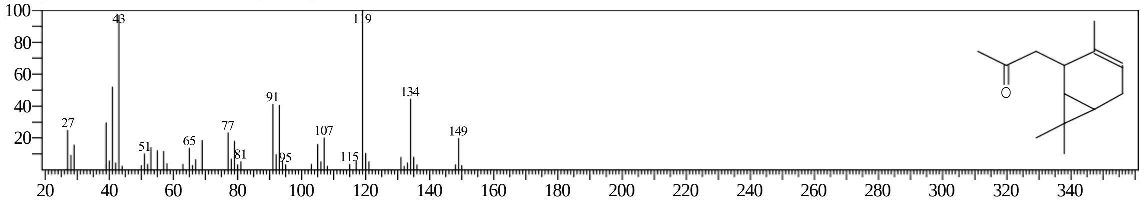

Hit#:2 Entry:36502 Library:NIST107.LIB
SI:80 Formula:C15H24 CAS:17699-05-7 MolWeight:204 RetIndex:0
CompName:Bicyclo[3.1.1]hept-2-ene, 2,6-dimethyl-6-(4-methyl-3-pentenyl)- $$ 2-Norpinene, 2,6-dimethyl-6-(4-methyl-3-pentenyl)- $$ .alpha.-Bergamoten

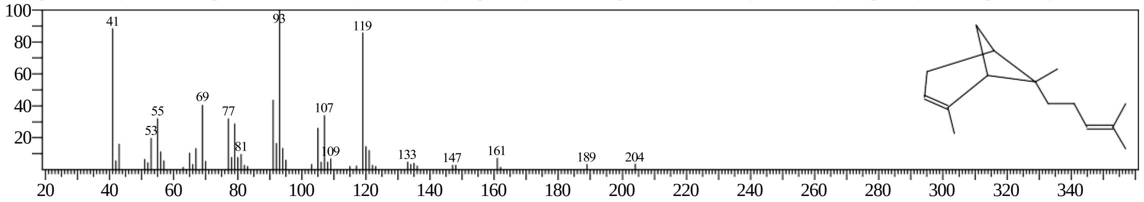

Hit#:3 Entry:18158 Library:NIST107.LIB
SI:80 Formula:C10H14N2 CAS:0-00-0 MolWeight:162 RetIndex:0
CompName:1,4-Methano-1H-cyclopenta[d]pyridazine, 4,4a,5,7a-tetrahydro-8,8-dimethyl-, (1.alpha.,4.alpha.,4a.alpha.,7a.alpha.)- $$

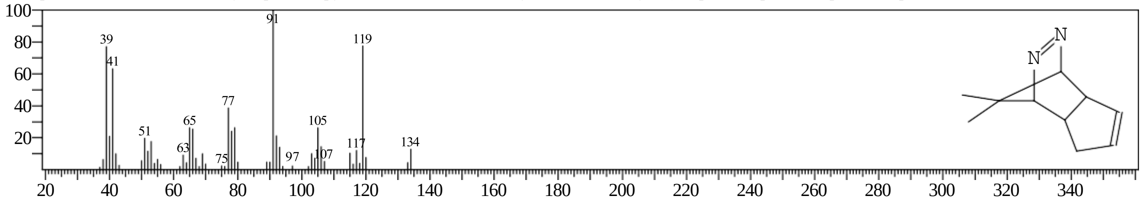

Hit#:4 Entry:8782 Library:NIST107.LIB
SI:80 Formula:C10H16 CAS:502-99-8 MolWeight:136 RetIndex:0
CompName:1,3,7-Octatriene, 3,7-dimethyl- $$ Ocimene $$ 2,6-Dimethyl-1,5,7-octatriene $$

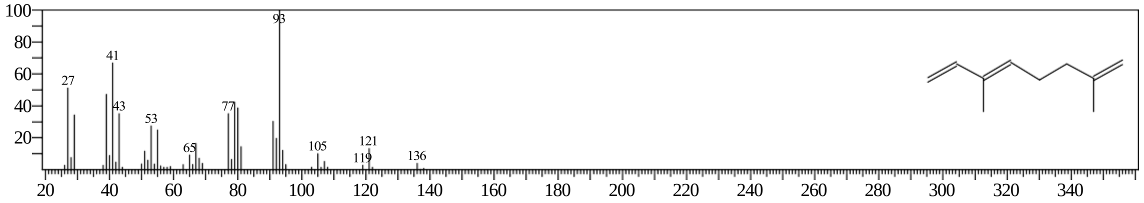

Hit#:5 Entry:18346 Library:NIST107.LIB
SI:79 Formula:C12H18 CAS:51567-08-9 MolWeight:162 RetIndex:0
CompName:Dispiro[2.1.2.4]undecane, 8-methylene- $$

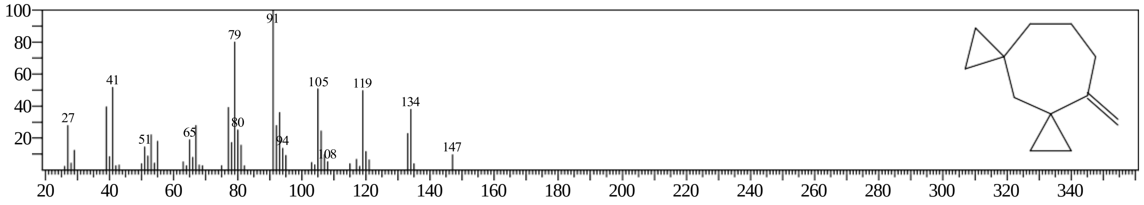

<< Target >>
Line#:30 R.Time:7.670(Scan#:1535) MassPeaks:64
RawMode:Averaged 7.665-7.675(1534-1536) BasePeak:139.30(217428)
BG Mode:Calc. from Peak

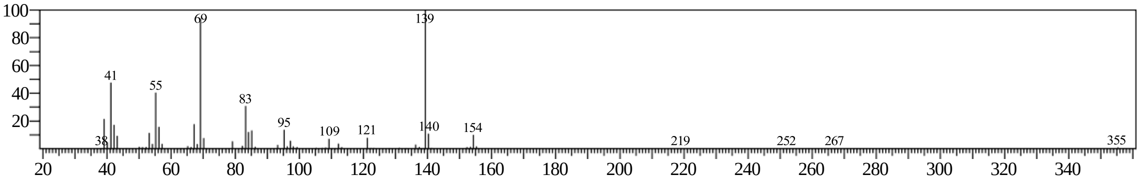

Hit#:1 Entry:15195 Library:NIST107.LIB
SI:87 Formula:C10H18O CAS:16409-43-1 MolWeight:154 RetIndex:0
CompName:2H-Pyran, tetrahydro-4-methyl-2-(2-methyl-1-propenyl)- $$ 2H-Pyran, tetrahydro-4-methyl-2-(2-methylpropenyl)- $$ Rose oxide $$ Rosoxide

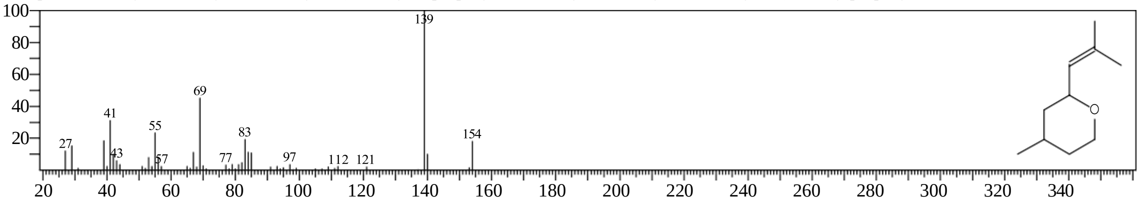

Hit#:2 Entry:15336 Library:NIST107.LIB
SI:79 Formula:C11H22 CAS:55499-08-6 MolWeight:154 RetIndex:0
CompName:Nonane, 2-methyl-3-methylene- $$ 2-Isopropyl-1-octene $$

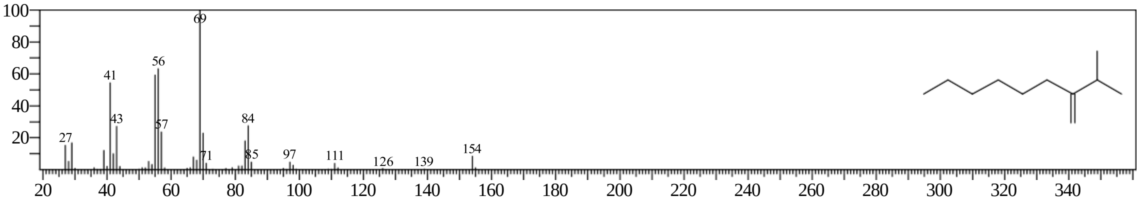

Hit#:3 Entry:27127 Library:NIST107.LIB
SI:78 Formula:C13H26 CAS:56851-45-7 MolWeight:182 RetIndex:0
CompName:2-Dodecene, 4-methyl- $$

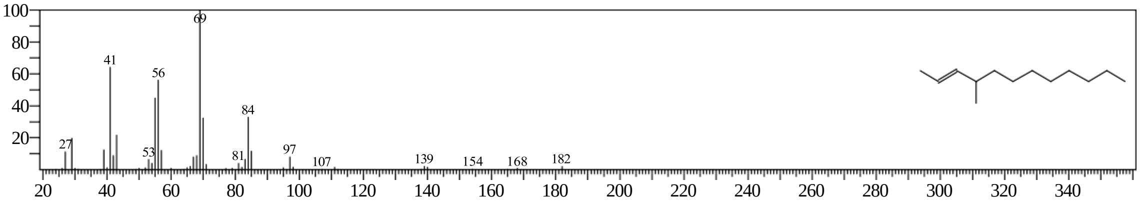

Hit#:4 Entry:15174 Library:NIST107.LIB
SI:78 Formula:C10H18O CAS:491-07-6 MolWeight:154 RetIndex:0
CompName:Cyclohexanone, 5-methyl-2-(1-methylethyl)-, cis- $$ p-Menthan-3-one, cis- $$ cis-p-Menthan-3-one $$ Isomenthone $$ p-Menthan-3-one, (Z)-

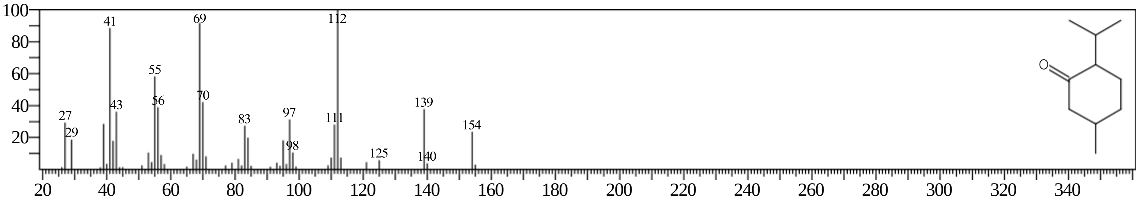

Hit#:5 Entry:15141 Library:NIST107.LIB
SI:78 Formula:C10H18O CAS:10458-14-7 MolWeight:154 RetIndex:0
CompName:Cyclohexanone, 5-methyl-2-(1-methylethyl)- $$ p-Menthan-3-one $$ Menthone $$ 2-Isopropyl-5-methylcyclohexanone $$ dl-Menthone $$ p-M

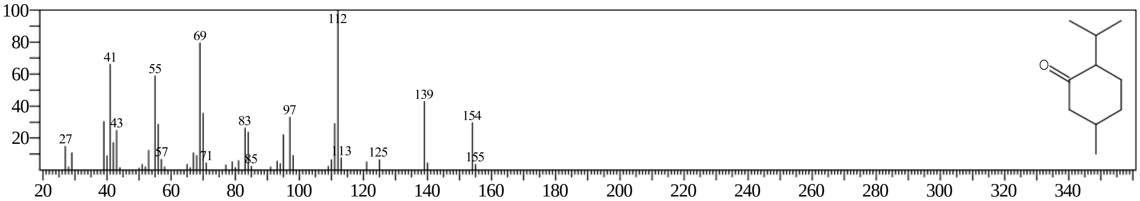

<< Target >>
Line#:31 R.Time:7.715(Scan#:1544) MassPeaks:93
RawMode:Averaged 7.710-7.720(1543-1545) BasePeak:81.20(153822)
BG Mode:Calc. from Peak

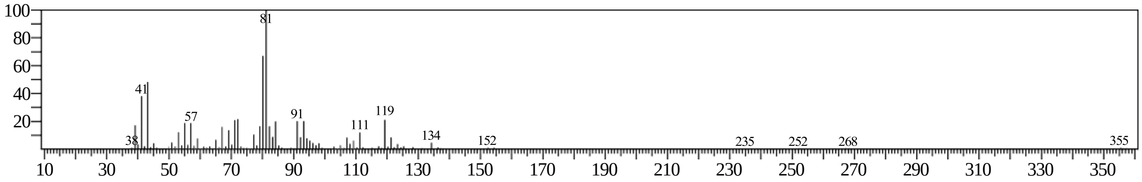

Hit#:1 Entry:15277 Library:NIST107.LIB
SI:89 Formula:C10H18O CAS:1632-73-1 MolWeight:154 RetIndex:0
CompName:Bicyclo[2.2.1]heptan-2-ol, 1,3,3-trimethyl- $$ 2-Norbornanol, 1,3,3-trimethyl- $$ D-Fenchyl alcohol $$ Fenchol $$ Fenchyl alcohol $$ 1,3,3-T

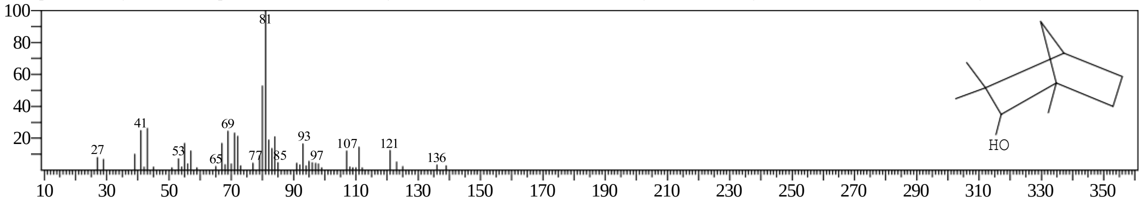

Hit#:2 Entry:15082 Library:NIST107.LIB
SI:88 Formula:C10H18O CAS:0-00-0 MolWeight:154 RetIndex:0
CompName:Fenchol $$

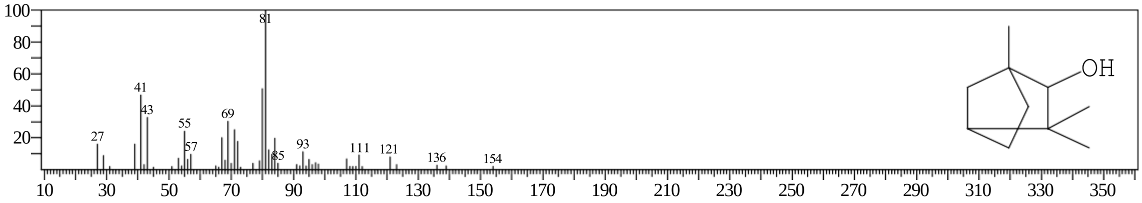

Hit#:3 Entry:15176 Library:NIST107.LIB
SI:87 Formula:C10H18O CAS:2217-02-9 MolWeight:154 RetIndex:0
CompName:Bicyclo[2.2.1]heptan-2-ol, 1,3,3-trimethyl-, (1R-endo)- $$ (1R)-endo-(+)-Fenchyl alcohol $$

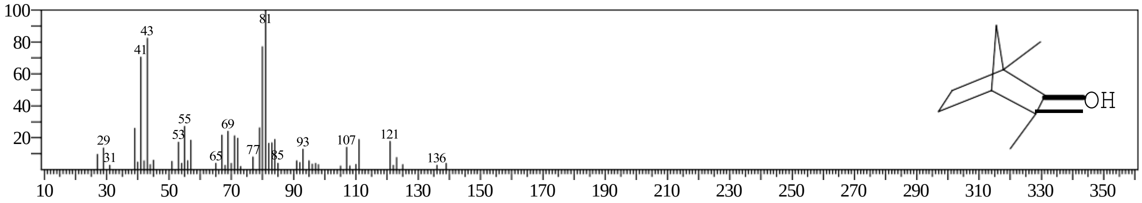

Hit#:4 Entry:15209 Library:NIST107.LIB
SI:87 Formula:C10H18O CAS:22627-95-8 MolWeight:154 RetIndex:0
CompName:Fenchol, exo- $$

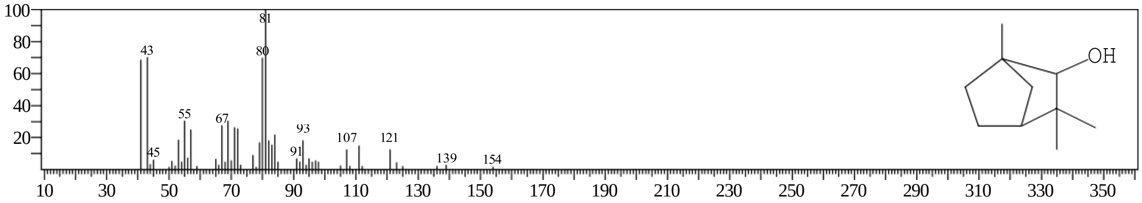

Hit#:5 Entry:33031 Library:NIST107.LIB
SI:83 Formula:C12H20O2 CAS:4057-31-2 MolWeight:196 RetIndex:0
CompName:Bicyclo[2.2.1]heptan-2-ol, 1,3,3-trimethyl-, acetate, endo- $$ 2-Norbornanol, 1,3,3-trimethyl-, acetate, endo- $$ .alpha.-Fenchyl acetate $$

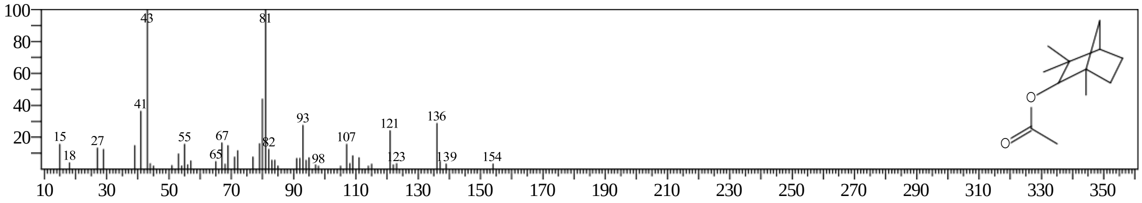

<< Target >>
Line#:32 R.Time:7.835(Scan#:1568) MassPeaks:93
RawMode:Averaged 7.830-7.840(1567-1569) BasePeak:91.20(7385912)
BG Mode:Calc. from Peak

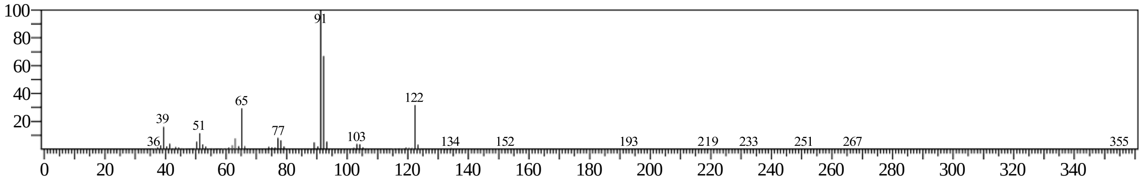

Hit#:1 Entry:5058 Library:NIST107.LIB
SI:95 Formula:C8H10O CAS:60-12-8 MolWeight:122 RetIndex:0
CompName:Phenylethyl Alcohol $$ Benzeneethanol $$ Phenethyl alcohol $$ .beta.-Hydroxyethylbenzene $$ .beta.-Phenethyl alcohol $$ .beta.-Phenylethan

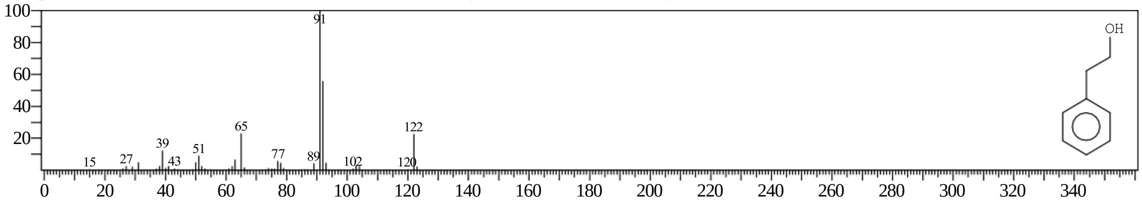

Hit#:2 Entry:5009 Library:NIST107.LIB
SI:87 Formula:C7H10N2 CAS:555-96-4 MolWeight:122 RetIndex:0
CompName:Hydrazine, (phenylmethyl)- $$ Hydrazine, benzyl- $$ Benzylhydrazine $$ Benzyl hydrazide $$ Benzyl hydrazid $$ Nako TMT $$ Pe $$ Pelago

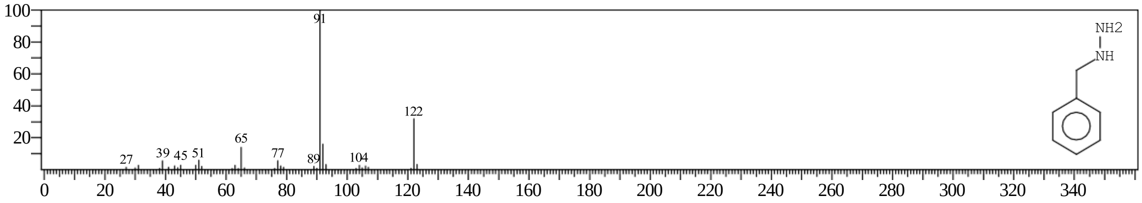

Hit#:3 Entry:16518 Library:NIST107.LIB
SI:86 Formula:C7H11ClN2 CAS:1073-62-7 MolWeight:158 RetIndex:0
CompName:Hydrazine, (phenylmethyl)-, monohydrochloride $$ Hydrazine, benzyl-, monohydrochloride $$ Benzylhydrazine hydrochloride $$ Z 102 $$ Hy

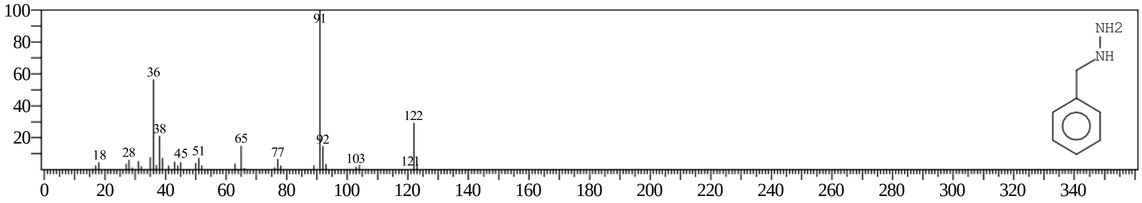

Hit#:4 Entry:1121 Library:NIST107.LIB
SI:85 Formula:C7H8 CAS:544-25-2 MolWeight:92 RetIndex:0
CompName:1,3,5-Cycloheptatriene $$ Cyclohepta-1,3,5-triene $$ Cycloheptatriene $$ Tropilidene $$ Tropilidin $$ UN 2603 $$

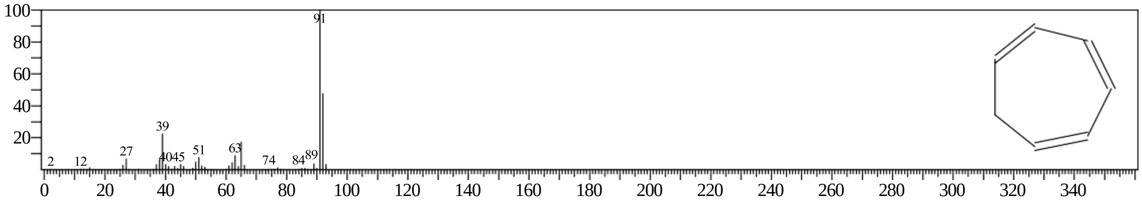

Hit#:5 Entry:8024 Library:NIST107.LIB
SI:85 Formula:C9H10O CAS:0-00-0 MolWeight:134 RetIndex:0
CompName:Bicyclo[2.2.2]oct-7-en-2-one, 5-methylene- $$

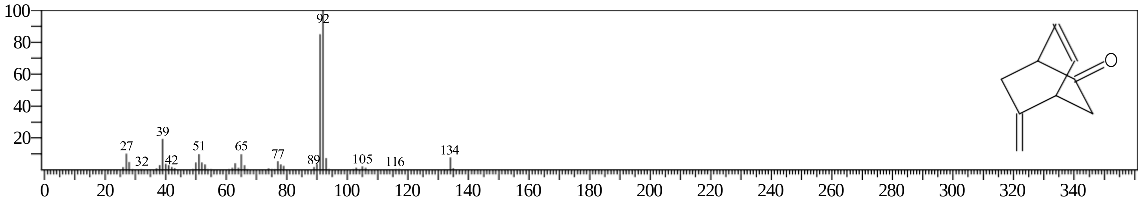

<< Target >>
Line#:33 R.Time:8.015(Scan#:1604) MassPeaks:80
RawMode:Averaged 8.010-8.020(1603-1605) BasePeak:67.15(158521)
BG Mode:Calc. from Peak

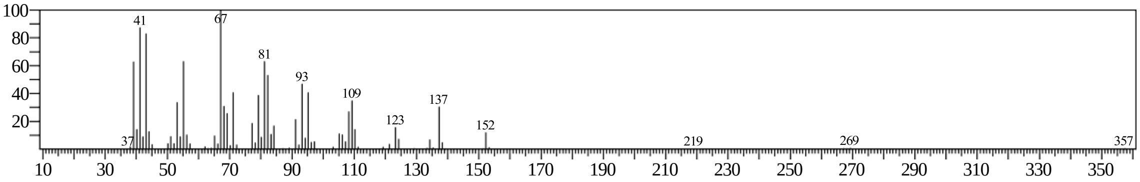

Hit#:1 Entry:14243 Library:NIST107.LIB
SI:86 Formula:C10H16O CAS:43108-69-6 MolWeight:152 RetIndex:0
CompName:Cyclohexanone, 2-(2-methylpropylidene)- $$

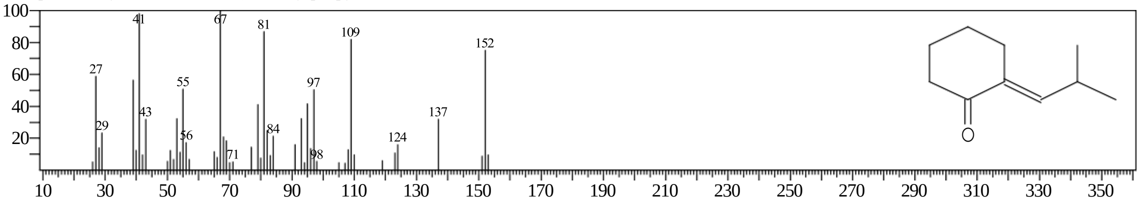

Hit#:2 Entry:21854 Library:NIST107.LIB
SI:85 Formula:C10H18O2 CAS:10359-41-8 MolWeight:170 RetIndex:0
CompName:Bicyclo[2.2.1]heptane-2,5-diol, 1,7,7-trimethyl-, (2-endo,5-exo)- $$

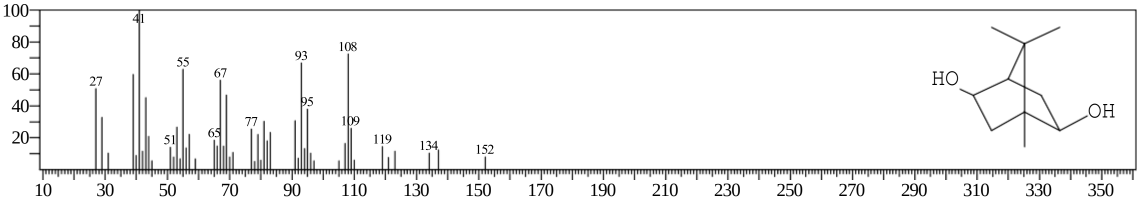

Hit#:3 Entry:14203 Library:NIST107.LIB
SI:85 Formula:C10H16O CAS:7764-50-3 MolWeight:152 RetIndex:0
CompName:Cyclohexanone, 2-methyl-5-(1-methylethenyl)- $$ p-Menth-8-en-2-one $$ Dihydrocarvone $$ (+)-Dihydrocarvone $$ D-Dihydrocarvone $$ 2-

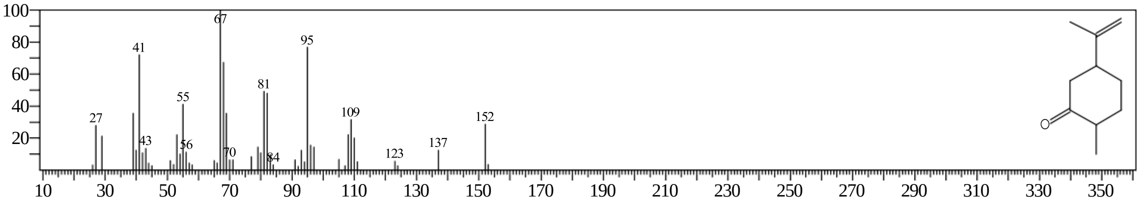

Hit#:4 Entry:14240 Library:NIST107.LIB
SI:85 Formula:C10H16O CAS:77954-13-3 MolWeight:152 RetIndex:0
CompName:Benzofuran, octahydro-6-methyl-3-methylene- $$

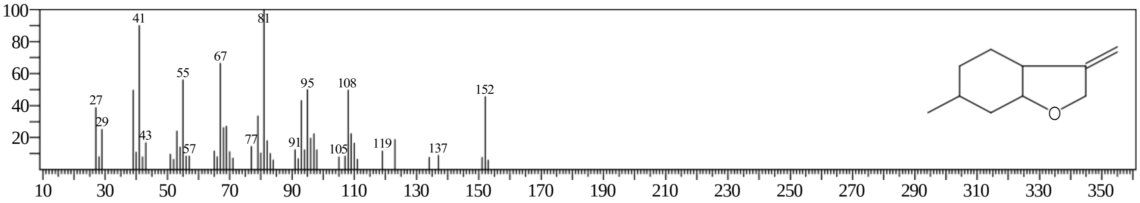

Hit#:5 Entry:14297 Library:NIST107.LIB
SI:84 Formula:C10H16O CAS:4832-16-0 MolWeight:152 RetIndex:0
CompName:1-Decalone (cis-trans) $$ 1(2H)-Naphthalenone, octahydro- $$ Bicyclo[4.4.0]-2-decanone $$

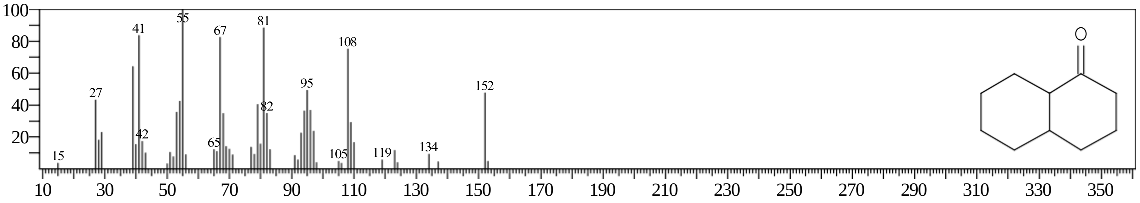

<< Target >>
Line#:34 R.Time:8.045(Scan#:1610) MassPeaks:89
RawMode:Averaged 8.040-8.050(1609-1611) BasePeak:43.15(119859)
BG Mode:Calc. from Peak

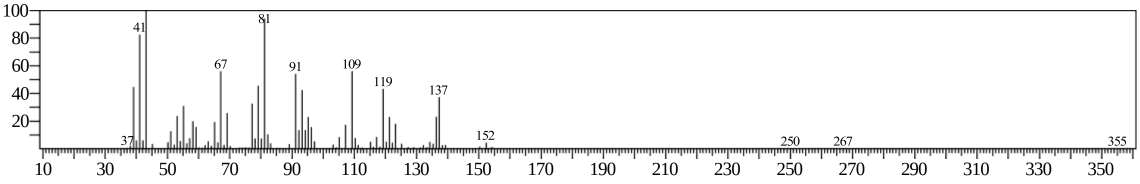

Hit#:1 Entry:14130 Library:NIST107.LIB
SI:82 Formula:C10H16O CAS:0-00-0 MolWeight:152 RetIndex:0
CompName:2,4,6-Trimethyl-3-cyclohexen-1-carboxaldehyde $$

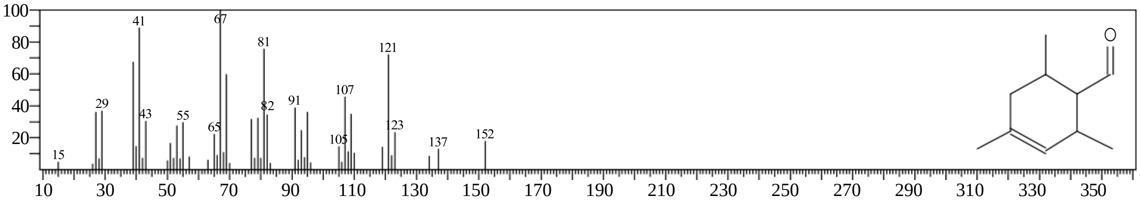

Hit#:2 Entry:14261 Library:NIST107.LIB
SI:82 Formula:C10H16O CAS:7212-40-0 MolWeight:152 RetIndex:0
CompName:2-Cyclohexen-1-ol, 1-methyl-4-(1-methylethenyl)-, trans- $$

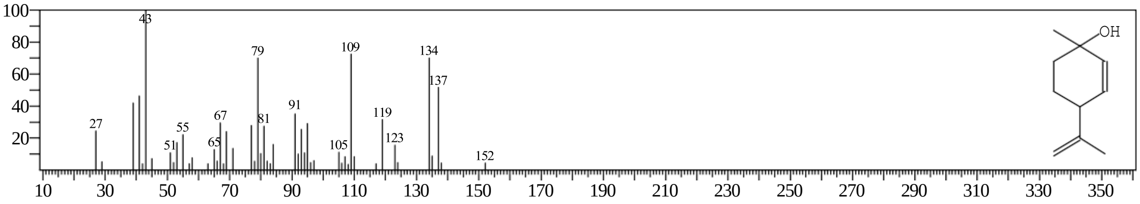

Hit#:3 Entry:14131 Library:NIST107.LIB
SI:82 Formula:C10H16O CAS:0-00-0 MolWeight:152 RetIndex:0
CompName:trans-p-2,8-Menthadien-1-ol $$

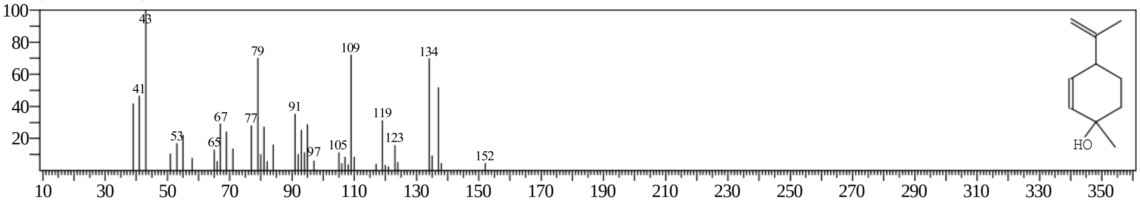

Hit#:4 Entry:14198 Library:NIST107.LIB
SI:82 Formula:C10H16O CAS:18881-04-4 MolWeight:152 RetIndex:0
CompName:Bicyclo[3.1.1]hept-3-en-2-ol, 4,6,6-trimethyl-, [1S-(1.alpha.,2.beta.,5.alpha.)]- $$ (S)-cis-Verbenol $$

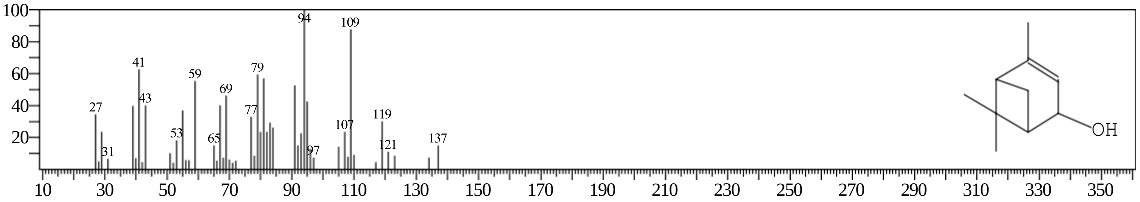

Hit#:5 Entry:14195 Library:NIST107.LIB
SI:82 Formula:C10H16O CAS:6909-20-2 MolWeight:152 RetIndex:0
CompName:Carane, 4,5-epoxy-, (E)- $$

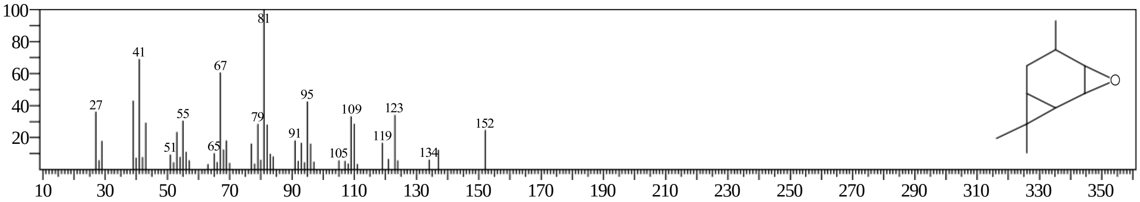

<< Target >>
Line#:35 R.Time:8.090(Scan#:1619) MassPeaks:100
RawMode:Averaged 8.085-8.095(1618-1620) BasePeak:67.15(214814)
BG Mode:Calc. from Peak

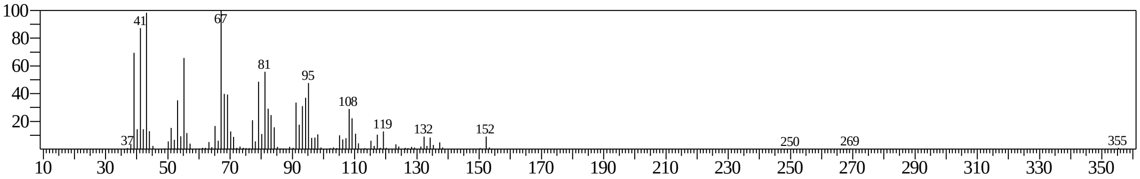

Hit#:1 Entry:14297 Library:NIST107.LIB
SI:86 Formula:C10H16O CAS:4832-16-0 MolWeight:152 RetIndex:0
CompName:1-Decalone (cis-trans) $$ 1(2H)-Naphthalenone, octahydro- $$ Bicyclo[4.4.0]-2-decanone $$

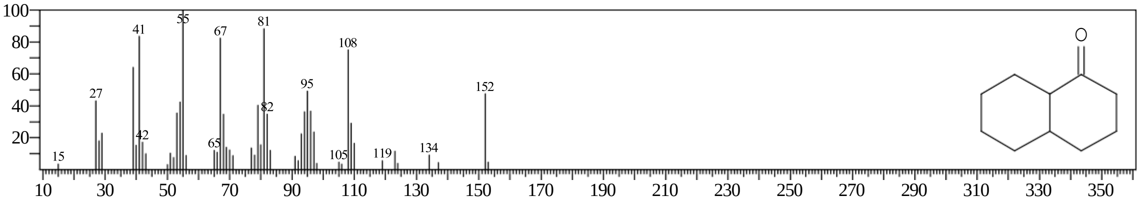

Hit#:2 Entry:21854 Library:NIST107.LIB
SI:85 Formula:C10H18O2 CAS:10359-41-8 MolWeight:170 RetIndex:0
CompName:Bicyclo[2.2.1]heptane-2,5-diol, 1,7,7-trimethyl-, (2-endo,5-exo)- $$

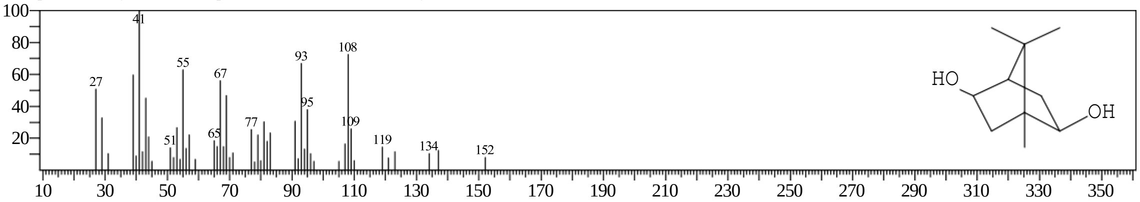

Hit#:3 Entry:9252 Library:NIST107.LIB
SI:85 Formula:C9H14O CAS:86951-58-8 MolWeight:138 RetIndex:0
CompName:Cyclopentanol, 1-(methylenecyclopropyl)- $$

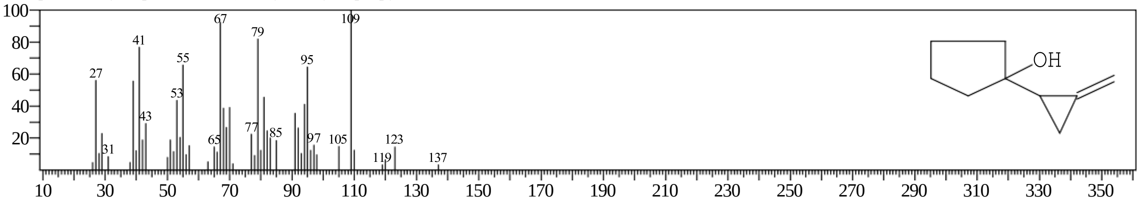

Hit#:4 Entry:14240 Library:NIST107.LIB
SI:85 Formula:C10H16O CAS:77954-13-3 MolWeight:152 RetIndex:0
CompName:Benzofuran, octahydro-6-methyl-3-methylene- $$

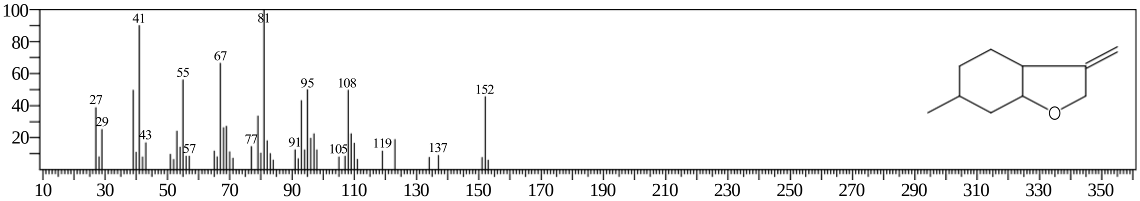

Hit#:5 Entry:10002 Library:NIST107.LIB
SI:84 Formula:C9H16O CAS:5921-73-3 MolWeight:140 RetIndex:0
CompName:2-Nonyn-1-ol $$

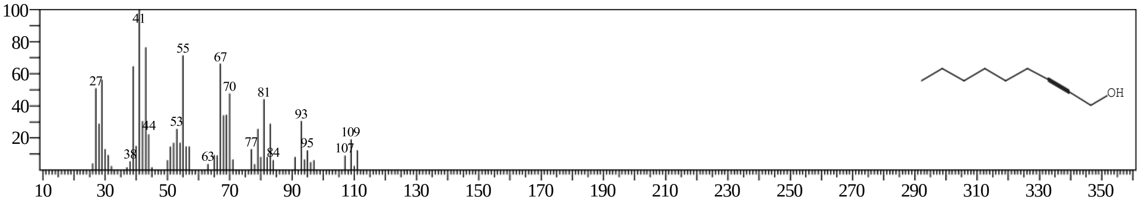

<< Target >>
Line#:36 R.Time:8.195(Scan#:1640) MassPeaks:117
RawMode:Averaged 8.190-8.200(1639-1641) BasePeak:95.20(5707636)
BG Mode:Calc. from Peak

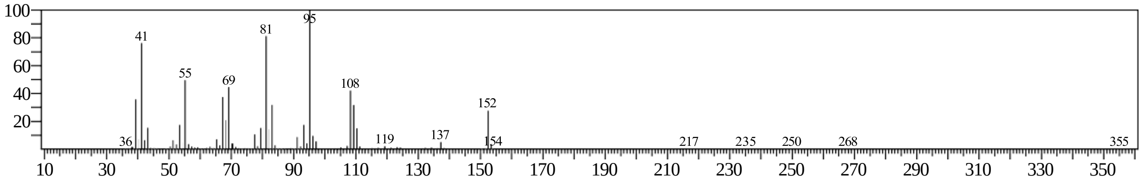

Hit#:1 Entry:14320 Library:NIST107.LIB
SI:96 Formula:C10H16O CAS:76-22-2 MolWeight:152 RetIndex:0
CompName:Camphor $$ Bicyclo[2.2.1]heptan-2-one, 1,7,7-trimethyl- $$ Camphor, natural $$ Root bark oil $$ Spirit of camphor $$ 1,7,7-Trimethylbicyclo

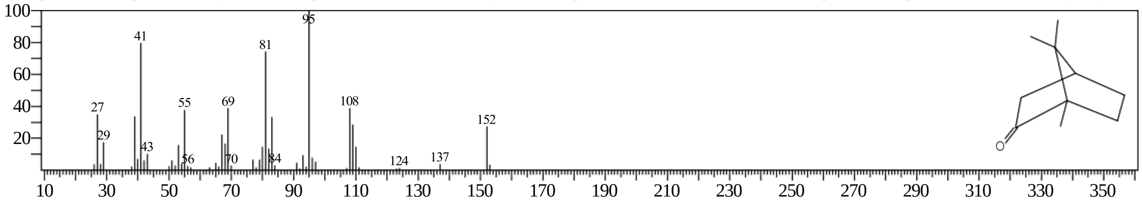

Hit#:2 Entry:14253 Library:NIST107.LIB
SI:96 Formula:C10H16O CAS:464-48-2 MolWeight:152 RetIndex:0
CompName:Bicyclo[2.2.1]heptan-2-one, 1,7,7-trimethyl-, (1S)- $$ (-)-Alcanfor $$ (-)-Camphor $$ Camphor, (1S,4S)-(-)- $$ L-camphor $$ Levo-(-)-camph

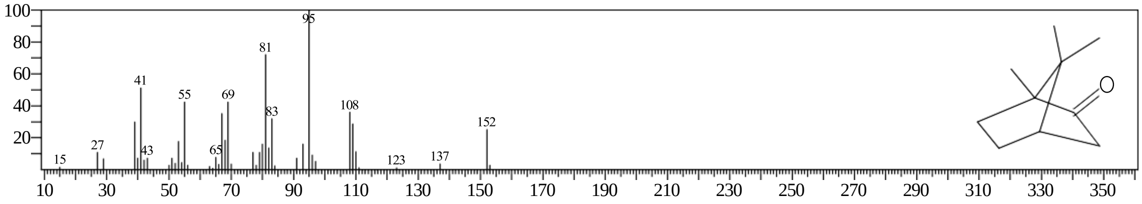

Hit#:3 Entry:14256 Library:NIST107.LIB
SI:95 Formula:C10H16O CAS:21368-68-3 MolWeight:152 RetIndex:0
CompName:Bicyclo[2.2.1]heptan-2-one, 1,7,7-trimethyl-, (.+/-.)- $$ Camphor, (.+/-.)- $$ (.+/-.)-Camphor $$ DL-Camphor $$ Bicyclo[2.2.1]heptan-2-one, 1

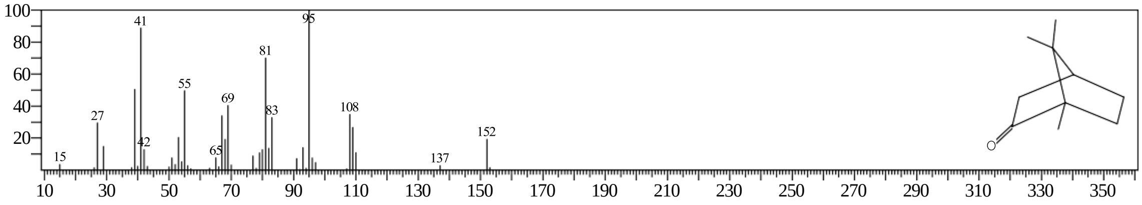

Hit#:4 Entry:14259 Library:NIST107.LIB
SI:92 Formula:C10H16O CAS:464-49-3 MolWeight:152 RetIndex:0
CompName:Bicyclo[2.2.1]heptan-2-one, 1,7,7-trimethyl-, (1R)- $$ CAMPHOR, (1R,4R)-(+)- $$ Alcanfor $$ (+)-2-Bornanone $$ d-2-Bornanone $$ d-2-C

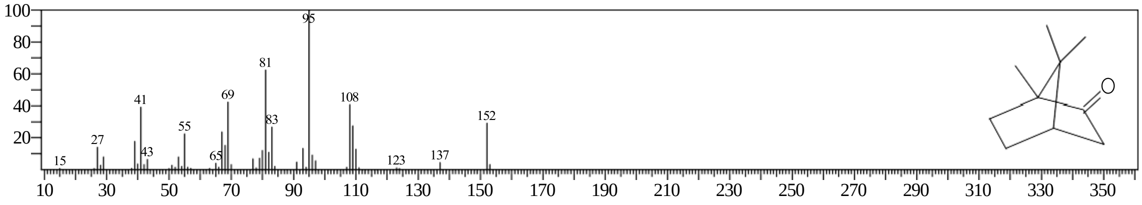

Hit#:5 Entry:49487 Library:NIST107.LIB
SI:92 Formula:C14H20O3 CAS:0-00-0 MolWeight:236 RetIndex:0
CompName:Spirobicyclo[2.2.1]heptane-2,2'-(1',3'-dioxa-2'-oxocyclohex-5'-ene)], 1,6',7,7-tetramethyl- $$

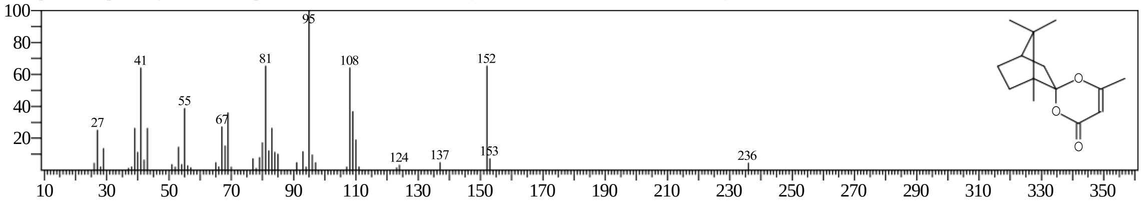

<< Target >>
Line#:37 R.Time:8.380(Scan#:1677) MassPeaks:104
RawMode:Averaged 8.375-8.385(1676-1678) BasePeak:95.20(5196033)
BG Mode:Calc. from Peak

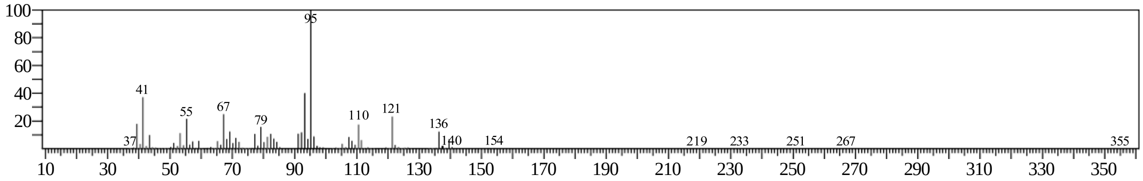

Hit#:1 Entry:26955 Library:NIST107.LIB
SI:91 Formula:C11H18O2 CAS:7492-41-3 MolWeight:182 RetIndex:0
CompName:Bicyclo[2.2.1]heptan-2-ol, 1,7,7-trimethyl-, formate, endo- $$ Borneol, formate $$ Bornyl formate $$

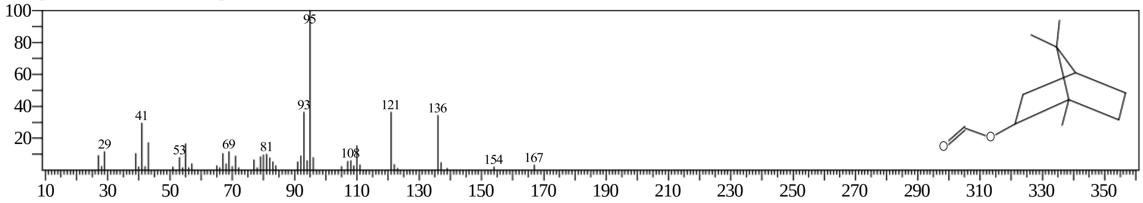

Hit#:2 Entry:26943 Library:NIST107.LIB
SI:91 Formula:C11H18O2 CAS:1200-67-5 MolWeight:182 RetIndex:0
CompName:Isobornyl formate $$ Bicyclo[2.2.1]heptan-2-ol, 1,7,7-trimethyl-, formate, exo- $$

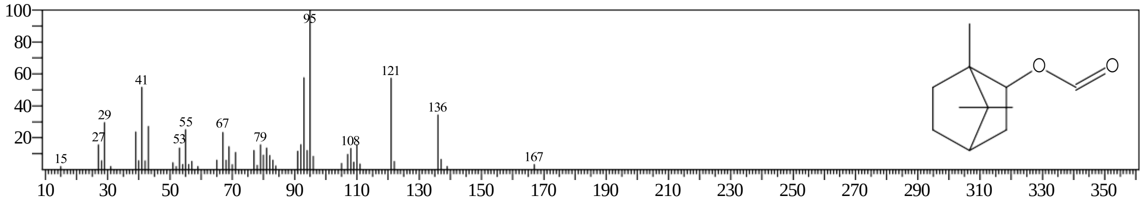

Hit#:3 Entry:15207 Library:NIST107.LIB
SI:90 Formula:C10H18O CAS:10385-78-1 MolWeight:154 RetIndex:0
CompName:Borneol $$

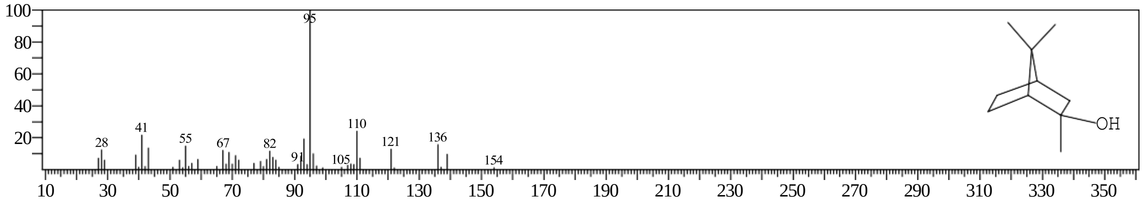

Hit#:4 Entry:15200 Library:NIST107.LIB
SI:90 Formula:C10H18O CAS:124-76-5 MolWeight:154 RetIndex:0
CompName:Isoborneol $$ Bicyclo[2.2.1]heptan-2-ol, 1,7,7-trimethyl-, exo- $$ exo-2-Hydroxy-1,7,7-trimethylnorbornane $$ Isobornyl alcohol $$ exo-1,7,7

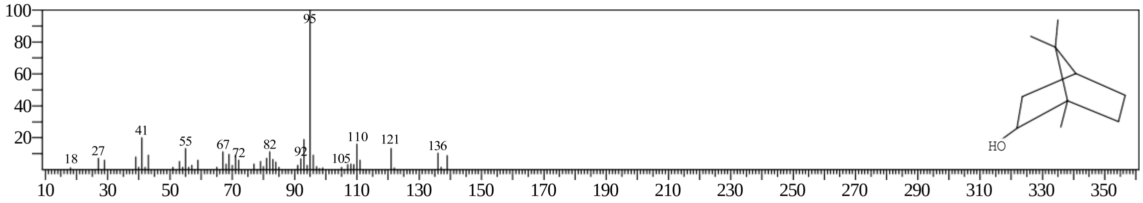

Hit#:5 Entry:84119 Library:NIST107.LIB
SI:89 Formula:C14H17F7O2 CAS:0-00-0 MolWeight:350 RetIndex:0
CompName:Borneol,heptafluorobutyrate (ester) $$

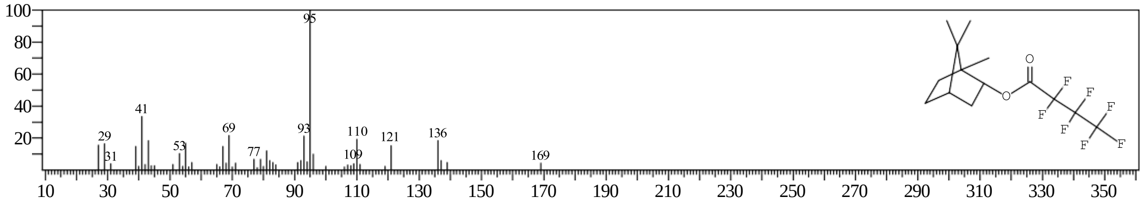

<< Target >>
Line#:38 R.Time:8.460(Scan#:1693) MassPeaks:56
RawMode:Averaged 8.455-8.465(1692-1694) BasePeak:87.15(1010528)
BG Mode:Calc. from Peak

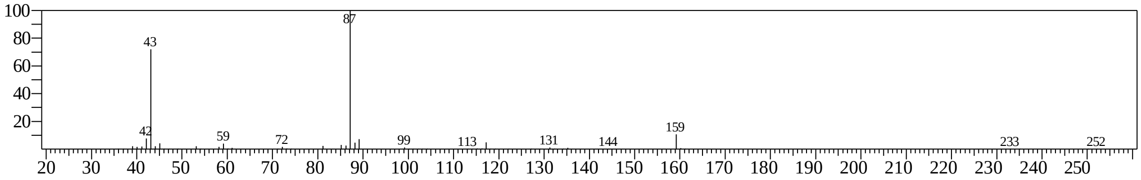

Hit#:1 Entry:23256 Library:NIST107.LIB
SI:95 Formula:C8H14O4 CAS:6413-10-1 MolWeight:174 RetIndex:0
CompName:Ethyl acetoacetate ethylene acetal $$ 1,3-Dioxolane-2-acetic acid, 2-methyl-, ethyl ester $$ Ethyl acetoacetate ethylene ketal $$ Ethyl acetoaceta

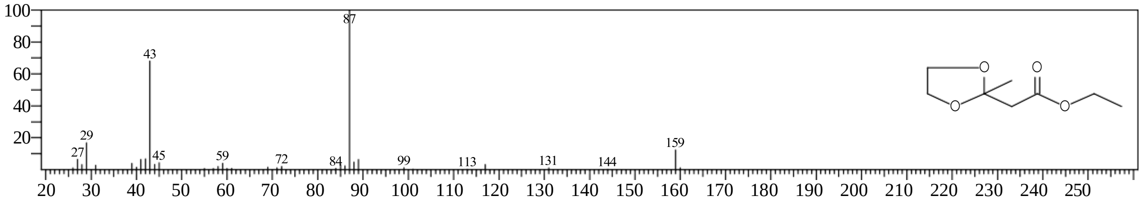

Hit#:2 Entry:48611 Library:NIST107.LIB
SI:88 Formula:C10H18O6 CAS:111-21-7 MolWeight:234 RetIndex:0
CompName:Ethanol, 2,2'-[1,2-ethanediylbis(oxy)]bis-, diacetate $$ Triethylene glycol, diacetate $$ Triglycol diacetate $$ Ethylenebis-(2-oxyethyl acetate) $

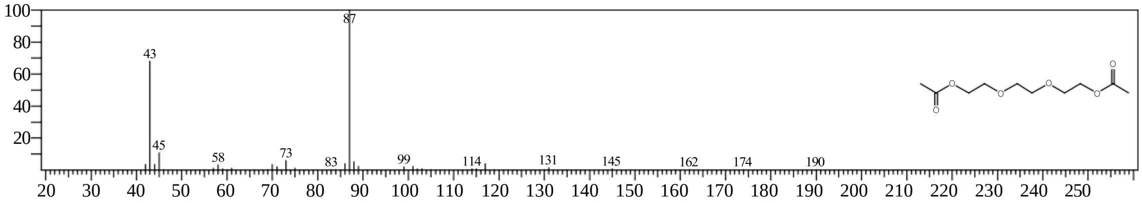

Hit#:3 Entry:30047 Library:NIST107.LIB
SI:87 Formula:C8H14O5 CAS:628-68-2 MolWeight:190 RetIndex:0
CompName:Ethanol, 2,2'-oxybis-, diacetate $$ Diethylene glycol, diacetate $$ Oxydiethylene acetate $$ Diglycol, diacetate $$

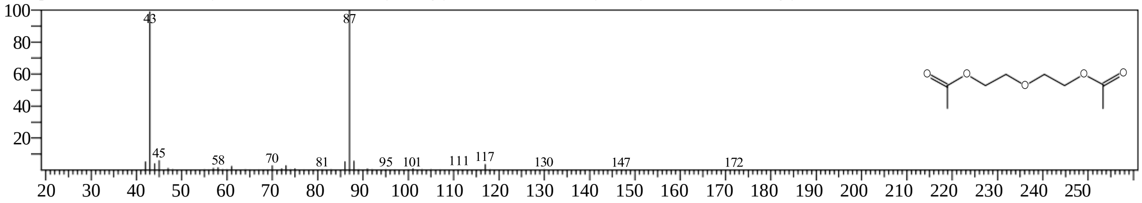

Hit#:4 Entry:7552 Library:NIST107.LIB
SI:84 Formula:C6H12O3 CAS:5754-32-5 MolWeight:132 RetIndex:0
CompName:1,3-Dioxolane-2-ethanol, 2-methyl- $$ 4-Hydroxy-2-butanone ethylene ketal $$

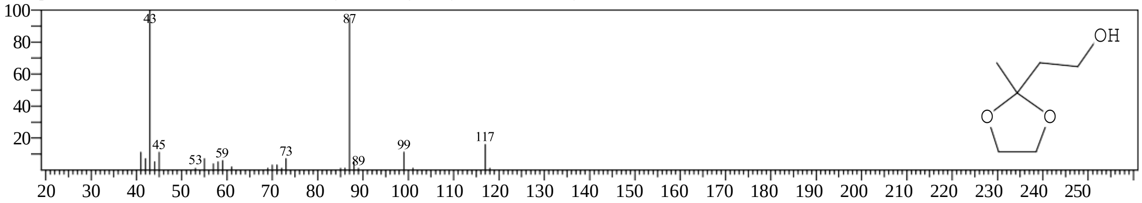

Hit#:5 Entry:23389 Library:NIST107.LIB
SI:84 Formula:C9H18O3 CAS:36651-23-7 MolWeight:174 RetIndex:0
CompName:1,3-Dioxolane-2-pentanol, 2-methyl- $$ 7-Hydroxy-2-heptanone ethyleneketal $$

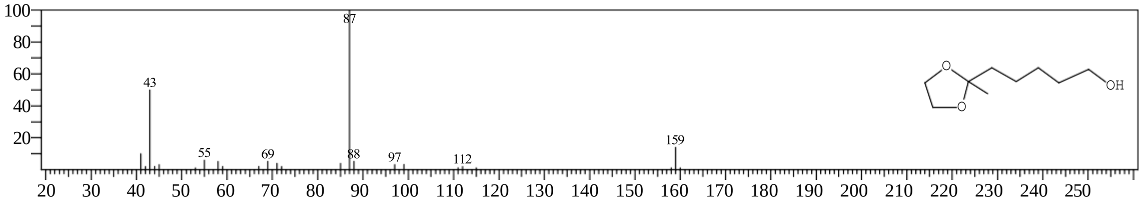

<< Target >>
Line#:39 R.Time:8.575(Scan#:1716) MassPeaks:109
RawMode:Averaged 8.570-8.580(1715-1717) BasePeak:108.15(8244128)
BG Mode:Calc. from Peak

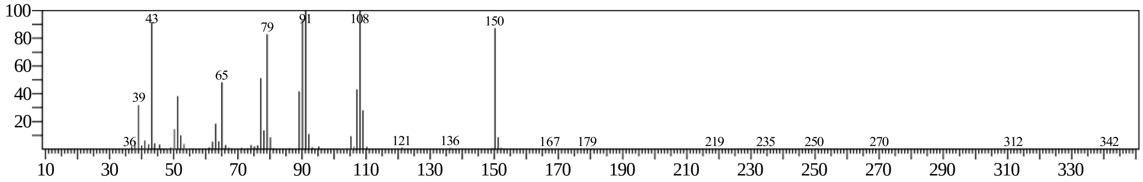

Hit#:1 Entry:13622 Library:NIST107.LIB
SI:80 Formula:C8H9NO2 CAS:621-84-1 MolWeight:151 RetIndex:0
CompName:Benzylcarbamate $$ Carbamic acid benzyl ester $$ Carbamic acid, phenylmethyl ester $$

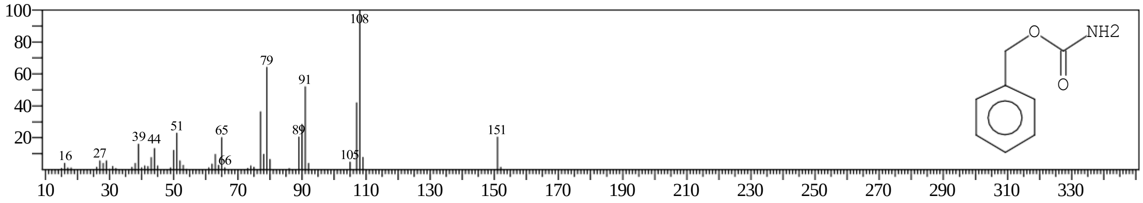

Hit#:2 Entry:13213 Library:NIST107.LIB
SI:79 Formula:C9H10O2 CAS:140-11-4 MolWeight:150 RetIndex:0
CompName:Acetic acid, phenylmethyl ester $$ Acetic acid, benzyl ester $$ .alpha.-Acetoxytoluene $$ Benzyl acetate $$ Benzyl ethanoate $$ NCI-C06508

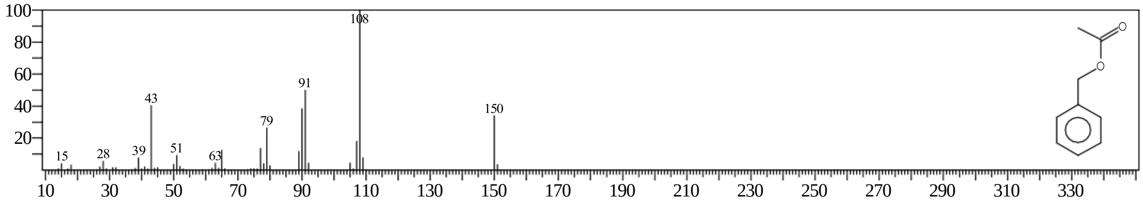

Hit#:3 Entry:8461 Library:NIST107.LIB
SI:79 Formula:C8H8O2 CAS:104-57-4 MolWeight:136 RetIndex:0
CompName:Formic acid, phenylmethyl ester $$ Benzyl alcohol, formate $$ Benzyl formate $$ Benzyl methanoate $$ Formic acid, benzyl ester $$ Benzyles

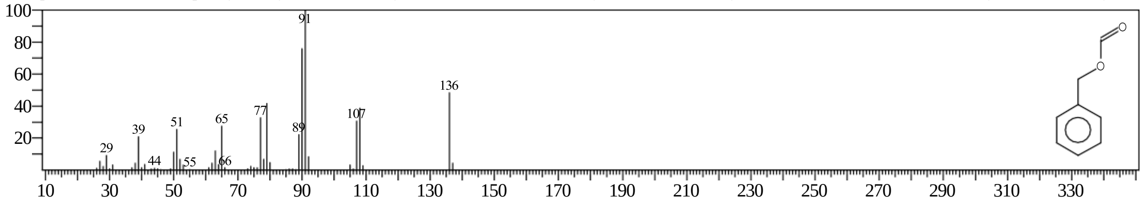

Hit#:4 Entry:86568 Library:NIST107.LIB
SI:78 Formula:C13H19N2O8P CAS:0-00-0 MolWeight:362 RetIndex:0
CompName:Benzyl-diseryl phosphate $$

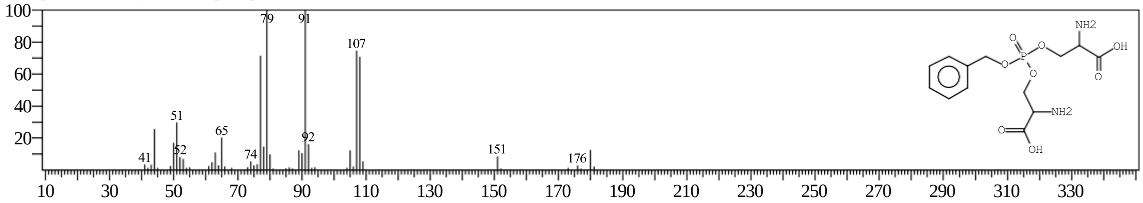

Hit#:5 Entry:44363 Library:NIST107.LIB
SI:78 Formula:C11H13NO4 CAS:4132-86-9 MolWeight:223 RetIndex:0
CompName:DL-Alanine, N-[(phenylmethoxy)carbonyl]- $$ Alanine, N-carboxy-, N-benzyl ester, DL- $$ N(Benzyloxycarbonyl)-DL-alanine $$ N-Carboben

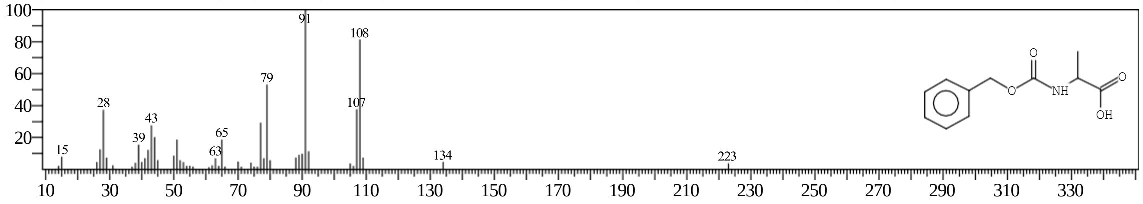

<< Target >>
Line#:40 R.Time:8.710(Scan#:1743) MassPeaks:112
RawMode:Averaged 8.705-8.715(1742-1744) BasePeak:91.15(1117294)
BG Mode:Calc. from Peak

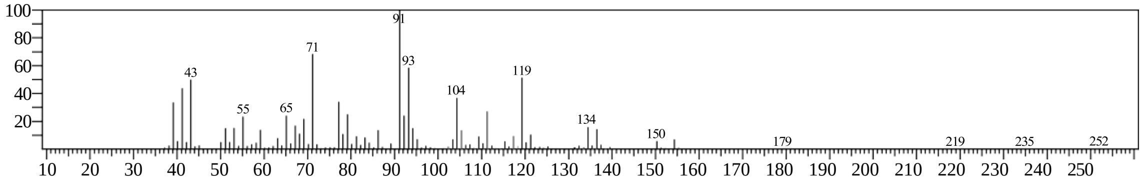

Hit#:1 Entry:15228 Library:NIST107.LIB
SI:77 Formula:C10H18O CAS:15537-55-0 MolWeight:154 RetIndex:0
CompName:Bicyclo[3.1.0]hexan-2-ol, 2-methyl-5-(1-methylethyl)-, (1.alpha.,2.beta.,5.alpha.)- $$

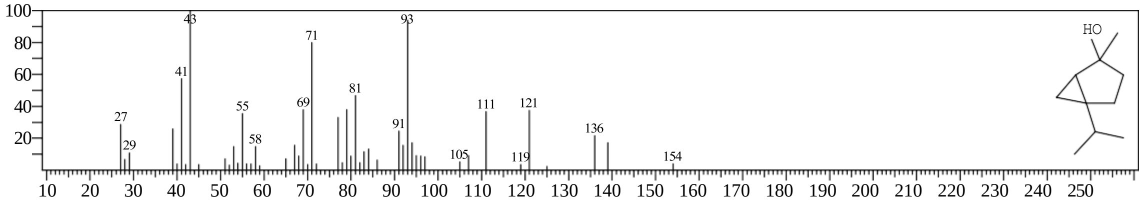

Hit#:2 Entry:31120 Library:NIST107.LIB
SI:76 Formula:C13H20O CAS:0-00-0 MolWeight:192 RetIndex:0
CompName:(+)-3-Carene, 10-(acetylmethyl)- $$

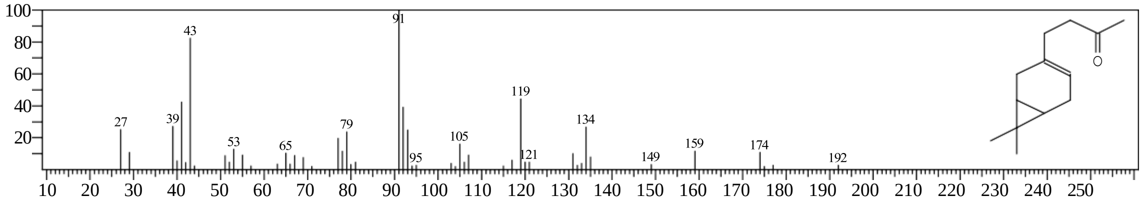

Hit#:3 Entry:15222 Library:NIST107.LIB
SI:75 Formula:C10H18O CAS:15826-82-1 MolWeight:154 RetIndex:0
CompName:cis-Sabinenehydrate $$

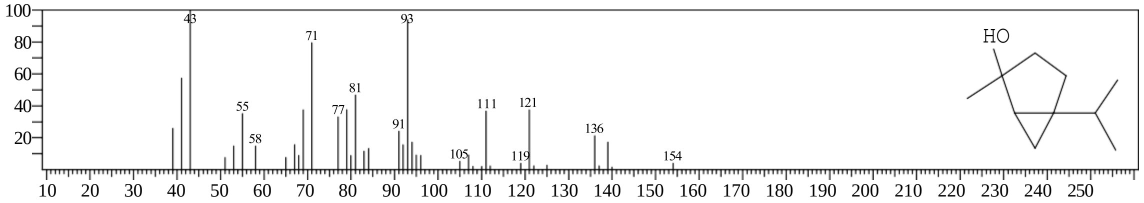

Hit#:4 Entry:14319 Library:NIST107.LIB
SI:75 Formula:C10H16O CAS:1820-09-3 MolWeight:152 RetIndex:0
CompName:Bicyclo[3.1.1]hept-3-en-2-ol, 4,6,6-trimethyl-, (1.alpha.,2.alpha.,5.alpha.)- $$ 2-Pinen-4-ol, trans- $$ trans-Verbenol $$

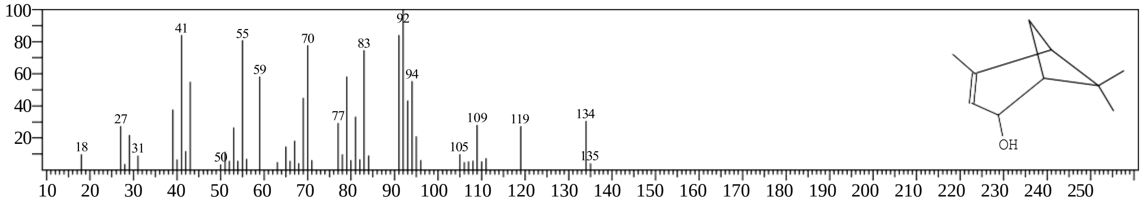

Hit#:5 Entry:20304 Library:NIST107.LIB
SI:74 Formula:C9H13NO2 CAS:80255-20-5 MolWeight:167 RetIndex:0
CompName:Cyclohexene, 1-(2-nitro-2-propenyl)- $$

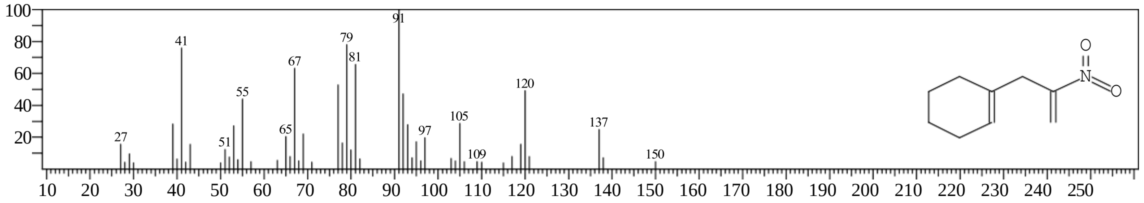

<< Target >>
Line#:41 R.Time:8.955(Scan#:1792) MassPeaks:120
RawMode:Averaged 8.950-8.960(1791-1793) BasePeak:59.15(7942697)
BG Mode:Calc. from Peak

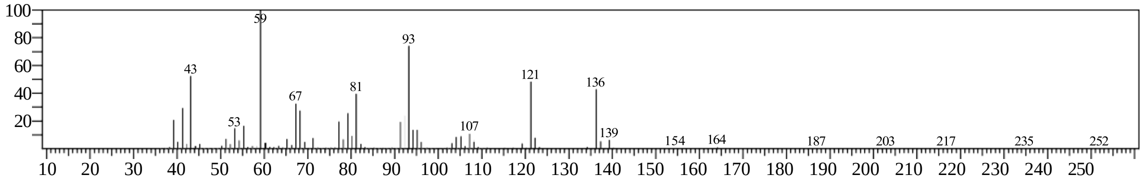

Hit#:1 Entry:15089 Library:NIST107.LIB
SI:91 Formula:C10H18O CAS:0-00-0 MolWeight:154 RetIndex:0
CompName:(+)-.alpha.-Terpineol (p-menth-1-en-8-ol) $$

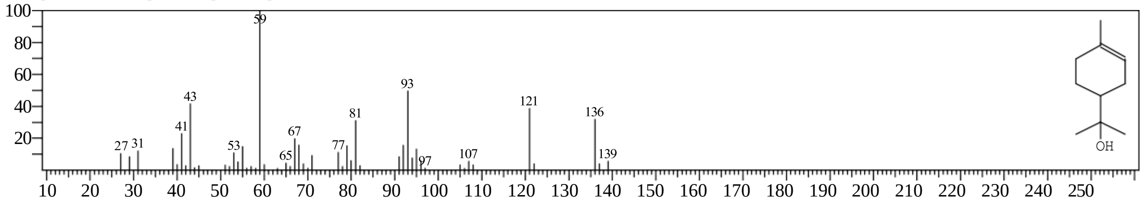

Hit#:2 Entry:15216 Library:NIST107.LIB
SI:91 Formula:C10H18O CAS:10482-56-1 MolWeight:154 RetIndex:0
CompName:3-Cyclohexene-1-methanol, .alpha.,.alpha.,4-trimethyl-, (S)- $$ p-Menth-1-en-8-ol, (S)-(-)- $$ .alpha.-Terpieol $$ .alpha.-Terpineol, (-)- $$ (-)-.a

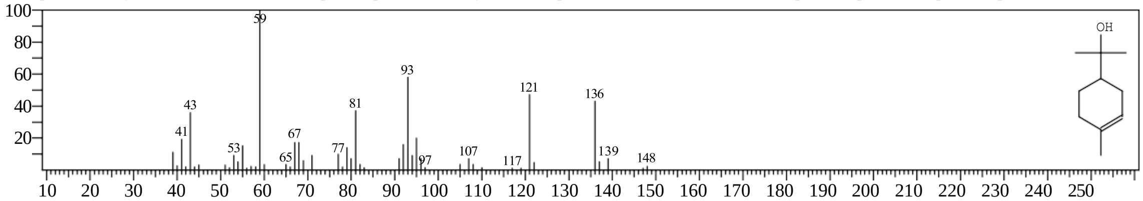

Hit#:3 Entry:15307 Library:NIST107.LIB
SI:90 Formula:C10H18O CAS:98-55-5 MolWeight:154 RetIndex:0
CompName:3-Cyclohexene-1-methanol, .alpha.,.alpha.4-trimethyl- $$ p-Menth-1-en-8-ol $$ .alpha.-Terpineol $$ Terpineol schlechthin $$ Terpineol $$ TER

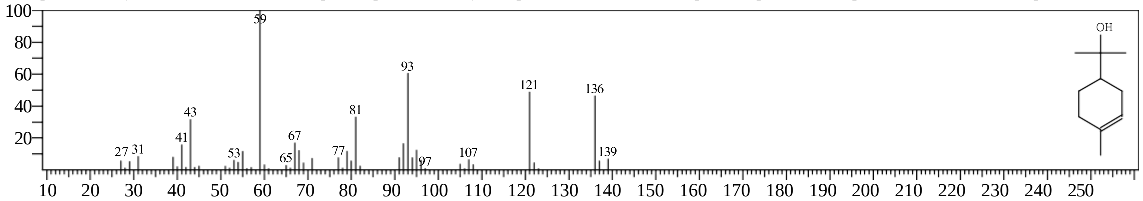

Hit#:4 Entry:15085 Library:NIST107.LIB
SI:89 Formula:C10H18O CAS:0-00-0 MolWeight:154 RetIndex:0
CompName:(-)-.alpha.-Terpineol (p-menth-1-en-8-ol) $$

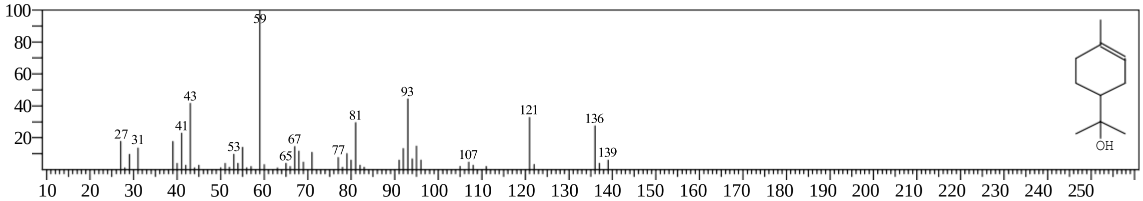

Hit#:5 Entry:33010 Library:NIST107.LIB
SI:85 Formula:C12H20O2 CAS:80-26-2 MolWeight:196 RetIndex:0
CompName:3-Cyclohexene-1-methanol, .alpha.,.alpha.,4-trimethyl-, acetate $$ p-Menth-1-en-8-ol, acetate $$ .alpha.-Terpineol acetate $$ .alpha.-Terpinyl ac

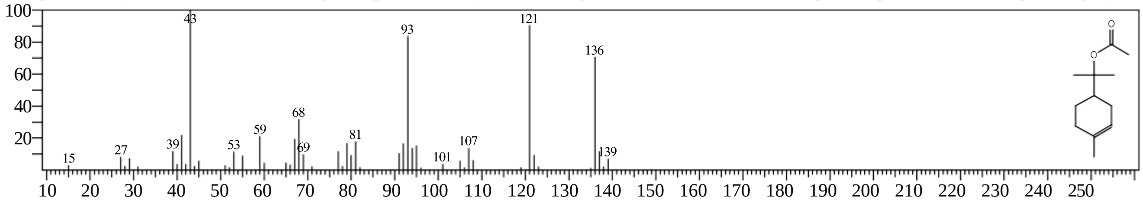

<< Target >>
Line#:42 R.Time:9.030(Scan#:1807) MassPeaks:86
RawMode:Averaged 9.025-9.035(1806-1808) BasePeak:121.25(261258)
BG Mode:Calc. from Peak

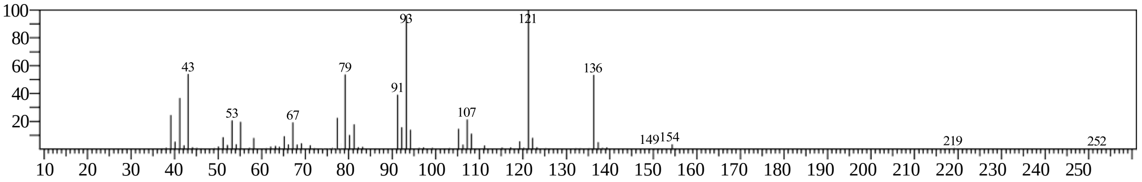

Hit#:1 Entry:8690 Library:NIST107.LIB
SI:91 Formula:C10H16 CAS:24524-57-0 MolWeight:136 RetIndex:0
CompName:Bicyclo[3.1.0]hexane, 6-isopropylidene-1-methyl- $$ Bicyclo[3.1.0]hexane, 1-methyl-6-(1-methylethylidene)- $$

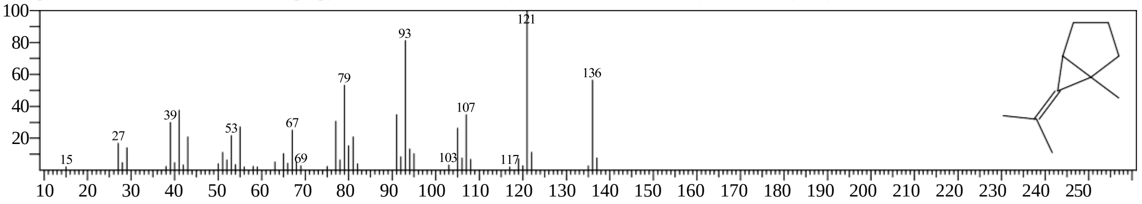

Hit#:2 Entry:8778 Library:NIST107.LIB
SI:90 Formula:C10H16 CAS:99805-90-0 MolWeight:136 RetIndex:0
CompName:Cyclohexene, 4-methyl-3-(1-methylethylidene)- $$

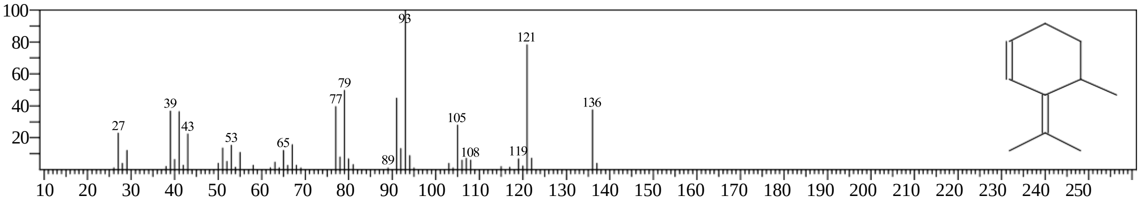

Hit#:3 Entry:8642 Library:NIST107.LIB
SI:90 Formula:C10H16 CAS:0-00-0 MolWeight:136 RetIndex:0
CompName:(+)-2-Caren $$

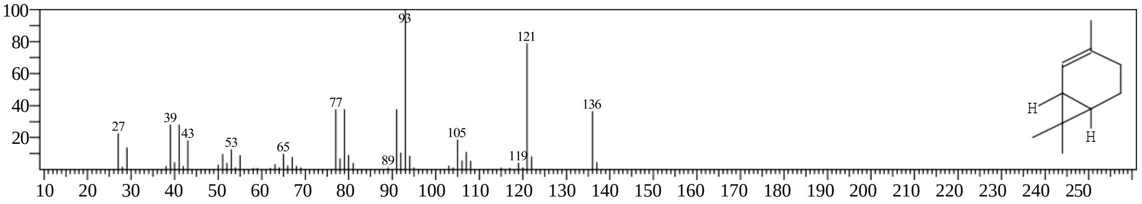

Hit#:4 Entry:8785 Library:NIST107.LIB
SI:89 Formula:C10H16 CAS:29050-33-7 MolWeight:136 RetIndex:0
CompName:(+)-4-Carene $$

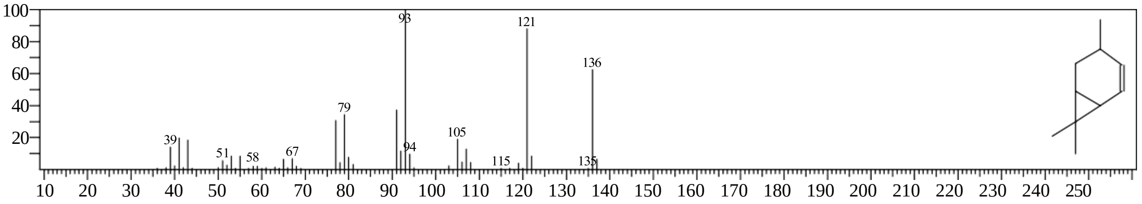

Hit#:5 Entry:8781 Library:NIST107.LIB
SI:89 Formula:C10H16 CAS:56816-08-1 MolWeight:136 RetIndex:0
CompName:Cyclohexene, 5-methyl-3-(1-methylethenyl)-, trans-(-)- $$

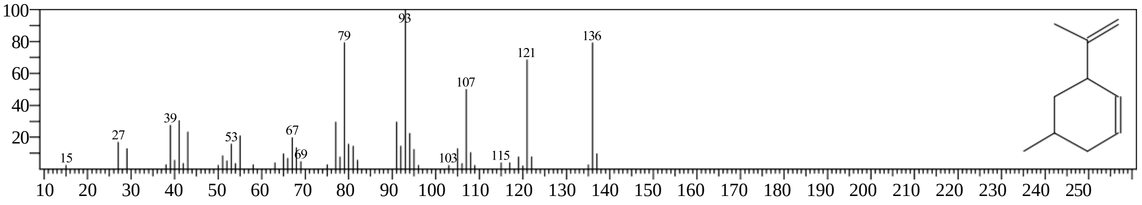

<< Target >>
Line#:43 R.Time:9.145(Scan#:1830) MassPeaks:65
RawMode:Averaged 9.140-9.150(1829-1831) BasePeak:119.25(92391)
BG Mode:Calc. from Peak

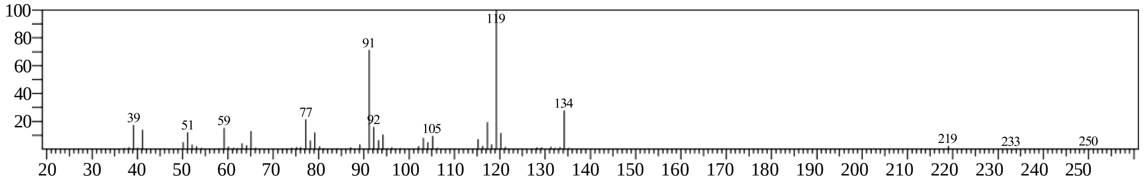

Hit#:1 Entry:14151 Library:NIST107.LIB
SI:90 Formula:C10H16O CAS:0-00-0 MolWeight:152 RetIndex:0
CompName:(E)-3-Caren-2-ol $$

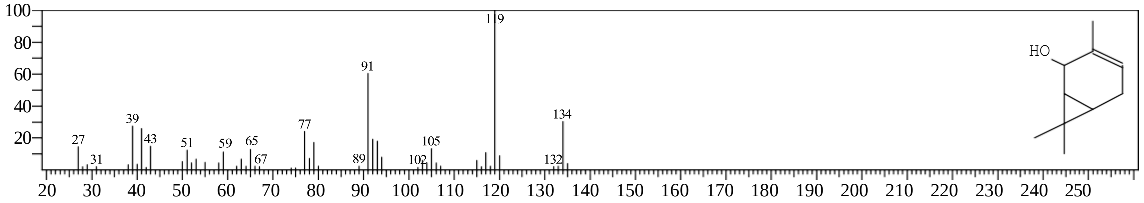

Hit#:2 Entry:8112 Library:NIST107.LIB
SI:88 Formula:C10H14 CAS:21195-59-5 MolWeight:134 RetIndex:0
CompName:1,3,8-p-Menthatriene $$ 1,3-Cyclohexadiene, 2-methyl-5-(1-methylethenyl)- $$

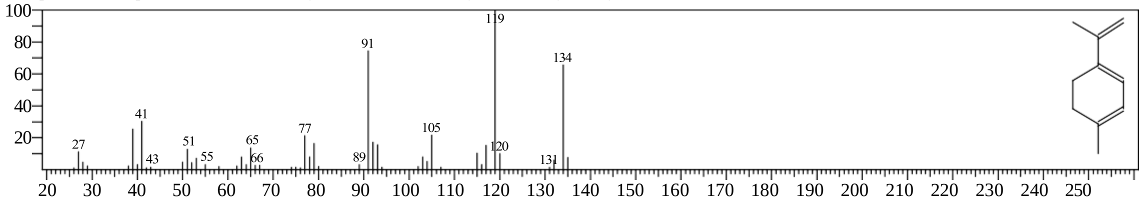

Hit#:3 Entry:8099 Library:NIST107.LIB
SI:88 Formula:C10H14 CAS:62338-57-2 MolWeight:134 RetIndex:0
CompName:1,4-Cyclohexadiene, 3-ethenyl-1,2-dimethyl- $$

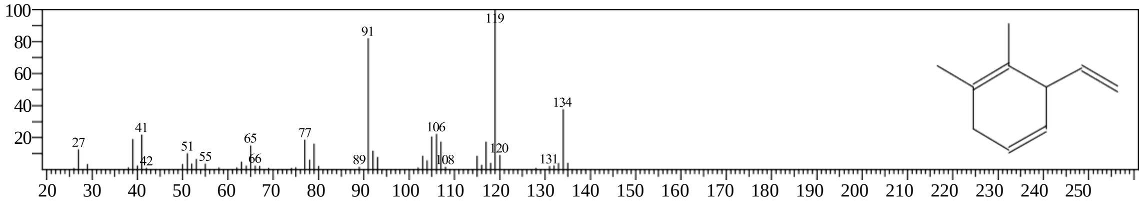

Hit#:4 Entry:8101 Library:NIST107.LIB
SI:86 Formula:C10H14 CAS:98-06-6 MolWeight:134 RetIndex:0
CompName:Benzene, tert-butyl- $$ Benzene, (1,1-dimethylethyl)- $$ tert-Butylbenzene $$ Dimethylethylbenzene $$ Phenyltrimethylmethane $$ Pseudobut

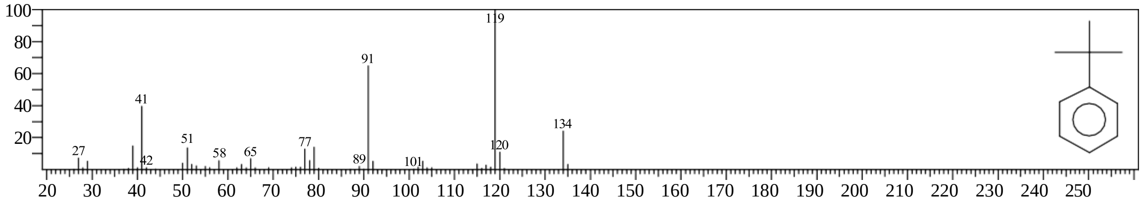

Hit#:5 Entry:14236 Library:NIST107.LIB
SI:86 Formula:C10H16O CAS:20053-58-1 MolWeight:152 RetIndex:0
CompName:2,3-Epoxycarane, (E)- $$

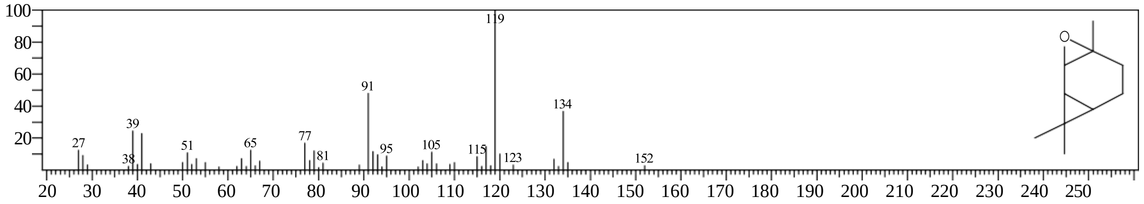

<< Target >>
Line#:44 R.Time:9.170(Scan#:1835) MassPeaks:64
RawMode:Averaged 9.165-9.175(1834-1836) BasePeak:43.10(67255)
BG Mode:Calc. from Peak

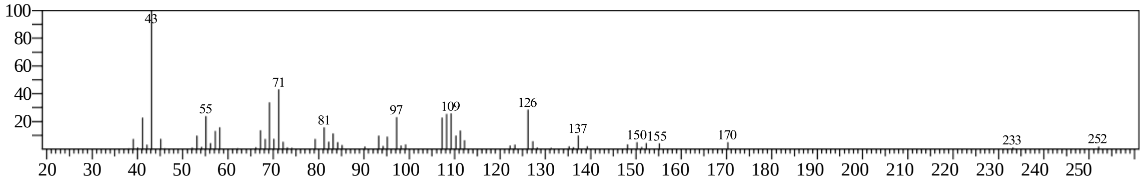

Hit#:1 Entry:21883 Library:NIST107.LIB
SI:83 Formula:C10H18O2 CAS:18679-48-6 MolWeight:170 RetIndex:0
CompName:2-Oxabicyclo[2.2.2]octan-6-ol, 1,3,3-trimethyl- $$

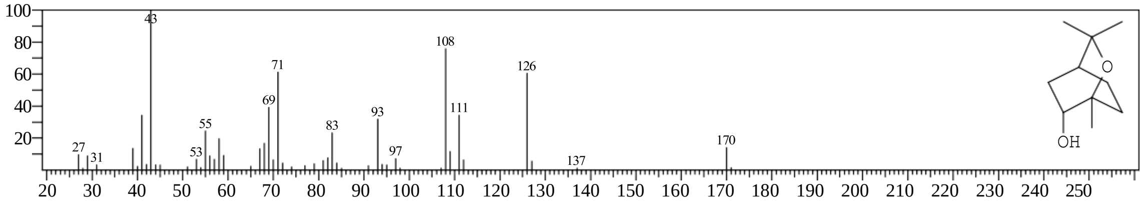

Hit#:2 Entry:39836 Library:NIST107.LIB
SI:82 Formula:C12H20O3 CAS:57709-95-2 MolWeight:212 RetIndex:0
CompName:2-Oxabicyclo[2.2.2]octan-6-ol, 1,3,3-trimethyl-, acetate $$

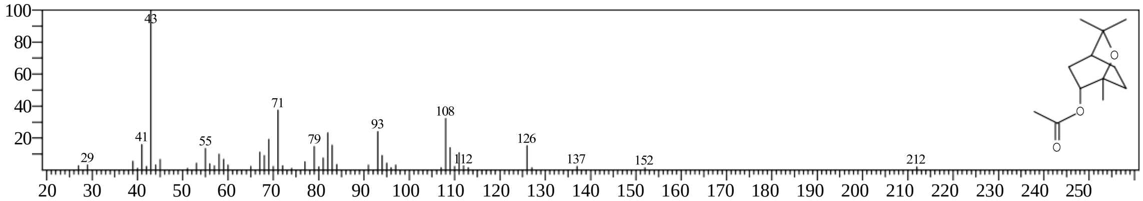

Hit#:3 Entry:21843 Library:NIST107.LIB
SI:80 Formula:C10H18O2 CAS:92999-78-5 MolWeight:170 RetIndex:0
CompName:exo-2-Hydroxycineole $$

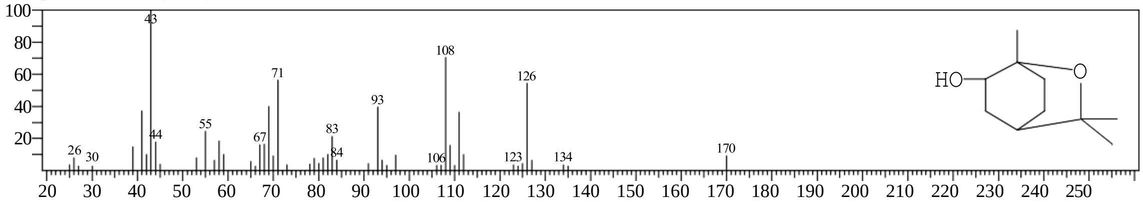

Hit#:4 Entry:21867 Library:NIST107.LIB
SI:80 Formula:C10H18O2 CAS:1946-00-5 MolWeight:170 RetIndex:0
CompName:1,2-Cyclohexanediol, 1-methyl-4-(1-methylethenyl)- $$

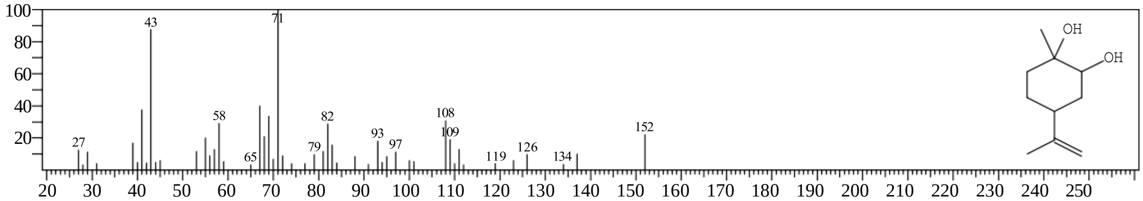

Hit#:5 Entry:21892 Library:NIST107.LIB
SI:79 Formula:C10H18O2 CAS:18680-27-8 MolWeight:170 RetIndex:0
CompName:(1S,2S,3R,5S)-(+)-Pinanediol $$

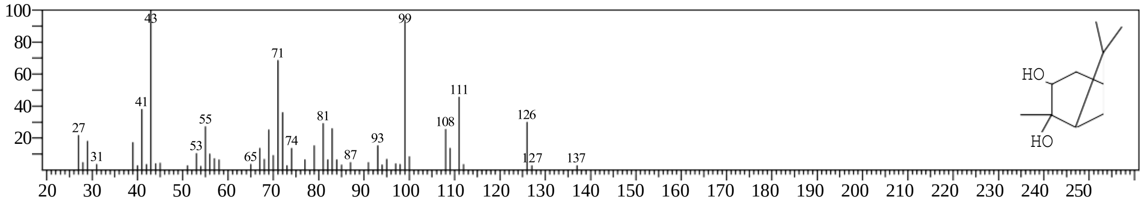

<< Target >>
Line#:45 R.Time:9.350(Scan#:1871) MassPeaks:99
RawMode:Averaged 9.345-9.355(1870-1872) BasePeak:91.15(189536)
BG Mode:Calc. from Peak

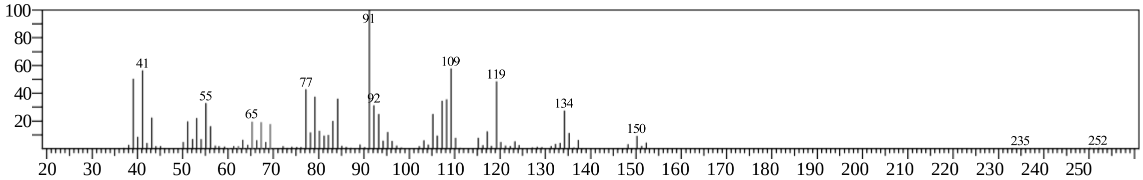

Hit#:1 Entry:14188 Library:NIST107.LIB
SI:88 Formula:C10H16O CAS:1197-06-4 MolWeight:152 RetIndex:0
CompName:2-Cyclohexen-1-ol, 2-methyl-5-(1-methylethenyl)-, cis- $$ p-Mentha-6,8-dien-2-ol, cis- $$ cis-Carveol $$

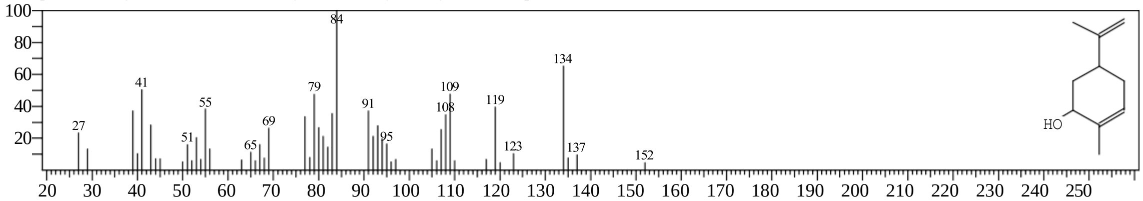

Hit#:2 Entry:32046 Library:NIST107.LIB
SI:85 Formula:C12H18O2 CAS:7111-29-7 MolWeight:194 RetIndex:0
CompName:2-Cyclohexen-1-ol, 2-methyl-5-(1-methylethenyl)-, acetate, (1R-cis)- $$ p-Mentha-6,8-dien-2-ol, acetate, cis-L- $$ (-)-cis-Carvyl Acetate $$ p-M

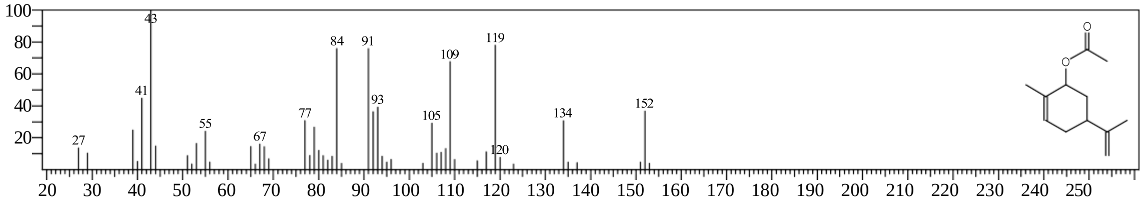

Hit#:3 Entry:32063 Library:NIST107.LIB
SI:84 Formula:C12H18O2 CAS:7053-79-4 MolWeight:194 RetIndex:0
CompName:2-Cyclohexen-1-ol, 2-methyl-5-(1-methylethenyl)-, acetate, (1R-trans)- $$ 2-Cyclohexen-1-ol, 2-methyl-5-(1-methylethenyl)-, acetate, (1S-trans

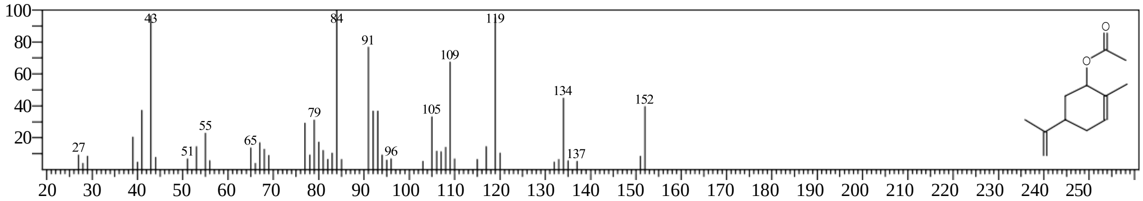

Hit#:4 Entry:14213 Library:NIST107.LIB
SI:83 Formula:C10H16O CAS:35907-10-9 MolWeight:152 RetIndex:0
CompName:Cyclohexanol, 2-methylene-5-(1-methylethenyl)- $$

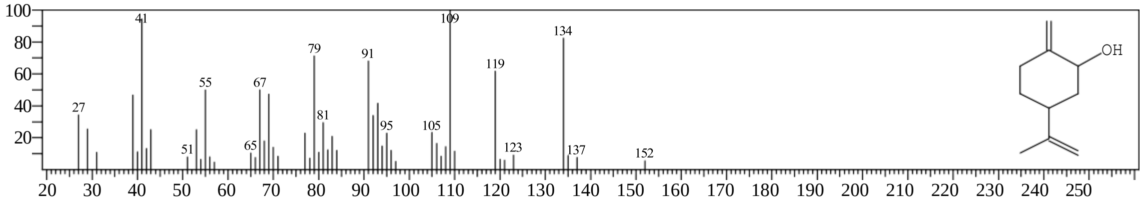

Hit#:5 Entry:32049 Library:NIST107.LIB
SI:83 Formula:C12H18O2 CAS:97-42-7 MolWeight:194 RetIndex:0
CompName:2-Cyclohexen-1-ol, 2-methyl-5-(1-methylethenyl)-, acetate $$ p-Mentha-6,8-dien-2-ol, acetate $$ p-Mentha-6,8-dien-2-ol, acetate, (+)- $$ Carv

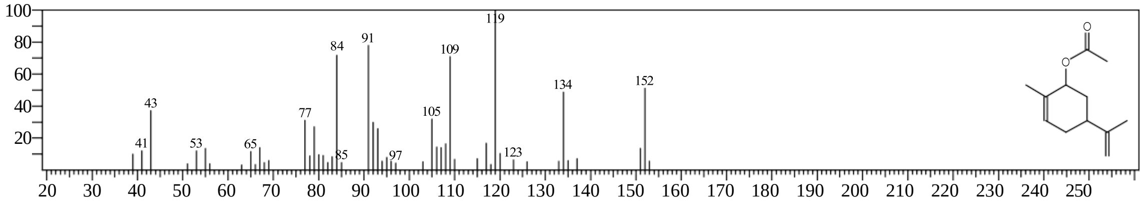

<< Target >>
Line#:46 R.Time:9.485(Scan#:1898) MassPeaks:116
RawMode:Averaged 9.480-9.490(1897-1899) BasePeak:41.15(4202061)
BG Mode:Calc. from Peak

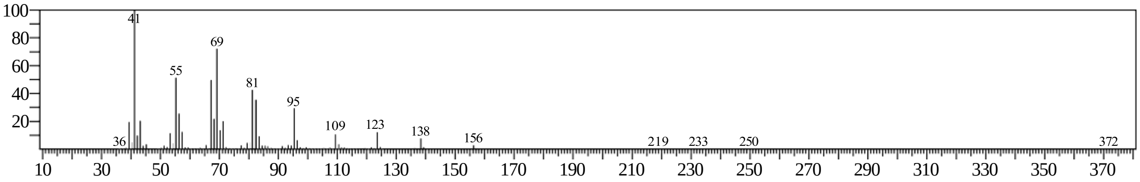

Hit#:1 Entry:16160 Library:NIST107.LIB
SI:96 Formula:C10H20O CAS:106-22-9 MolWeight:156 RetIndex:0
CompName:6-Octen-1-ol, 3,7-dimethyl- $$ .beta.-Citronellol $$ Cephrol $$ Citronellol $$ Rodinol $$ 3,7-Dimethyl-6-octen-1-ol $$ Elenol $$ RHODINOL

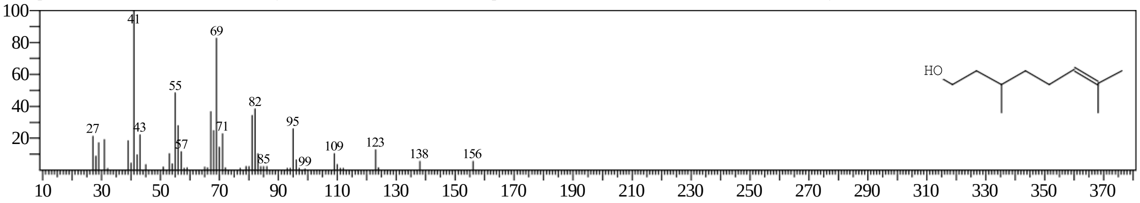

Hit#:2 Entry:16139 Library:NIST107.LIB
SI:95 Formula:C10H20O CAS:1117-61-9 MolWeight:156 RetIndex:0
CompName:6-Octen-1-ol, 3,7-dimethyl-, (R)- $$ (R)-(+)-.beta.-Citronellol $$

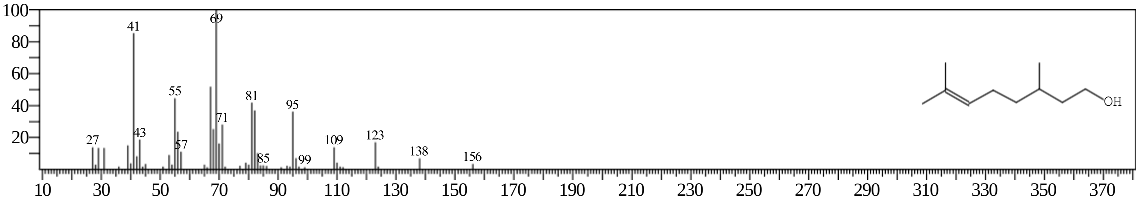

Hit#:3 Entry:16047 Library:NIST107.LIB
SI:92 Formula:C10H20O CAS:0-00-0 MolWeight:156 RetIndex:0
CompName:2-Octen-1-ol, 3,7-dimethyl- $$

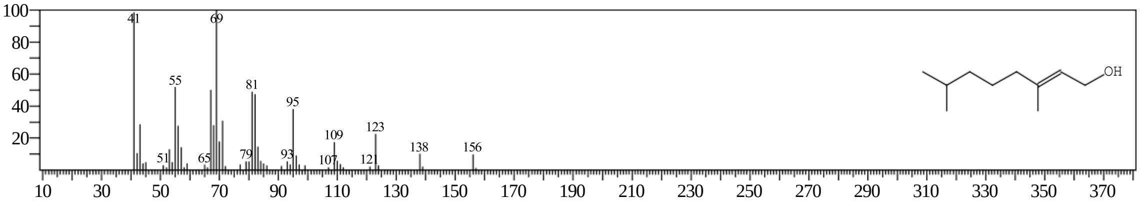

Hit#:4 Entry:34000 Library:NIST107.LIB
SI:92 Formula:C13H26O CAS:4361-23-3 MolWeight:198 RetIndex:0
CompName:Tetrahydroionol $$ Cyclohexanepropanol, .alpha.,2,2,6-tetramethyl- $$ 4-(2,2,6-Trimethylcyclohexyl)-2-butanol $$

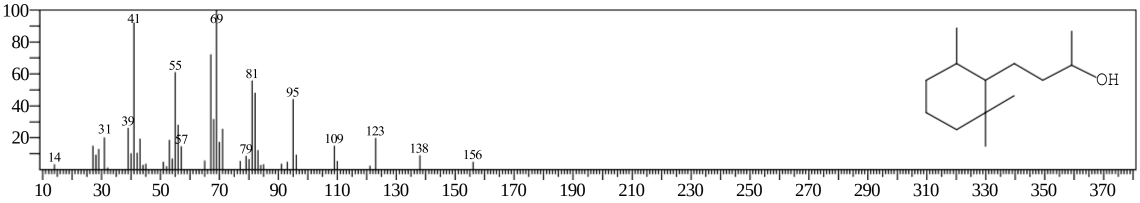

Hit#:5 Entry:27805 Library:NIST107.LIB
SI:91 Formula:C11H20O2 CAS:105-85-1 MolWeight:184 RetIndex:0
CompName:6-Octen-1-ol, 3,7-dimethyl-, formate $$ Citronellyl formate $$ Formic acid, citronellyl ester $$ Formic acid, 3,7-dimethyl-6-octen-1-yl ester $$

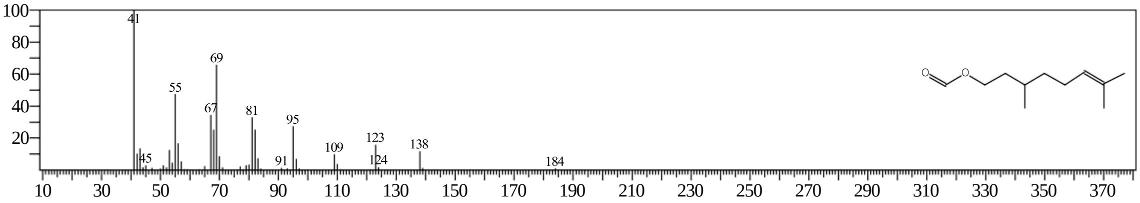

<< Target >>
Line#:47 R.Time:9.625(Scan#:1926) MassPeaks:93
RawMode:Averaged 9.620-9.630(1925-1927) BasePeak:43.15(266650)
BG Mode:Calc. from Peak

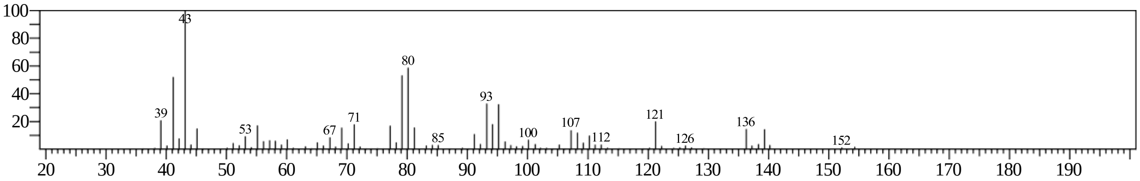

Hit#:1 Entry:32972 Library:NIST107.LIB
SI:83 Formula:C12H20O2 CAS:0-00-0 MolWeight:196 RetIndex:0
CompName:Bornyl acetate $$

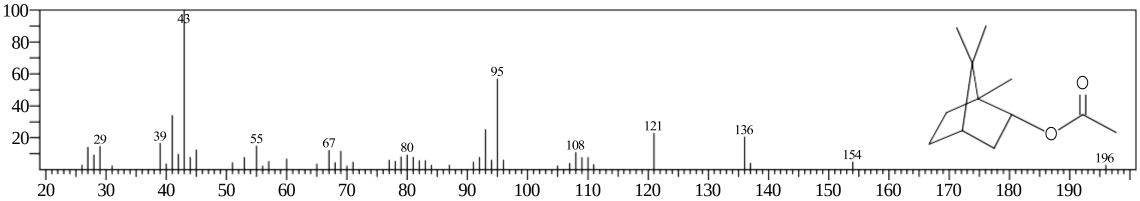

Hit#:2 Entry:15143 Library:NIST107.LIB
SI:82 Formula:C10H18O CAS:29803-81-4 MolWeight:154 RetIndex:0
CompName:2-Cyclohexen-1-ol, 1-methyl-4-(1-methylethyl)-, trans- $$

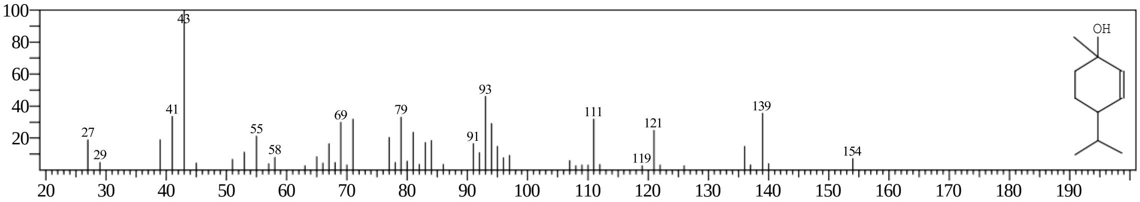

Hit#:3 Entry:15154 Library:NIST107.LIB
SI:81 Formula:C10H18O CAS:29803-82-5 MolWeight:154 RetIndex:0
CompName:2-Cyclohexen-1-ol, 1-methyl-4-(1-methylethyl)-, cis- $$

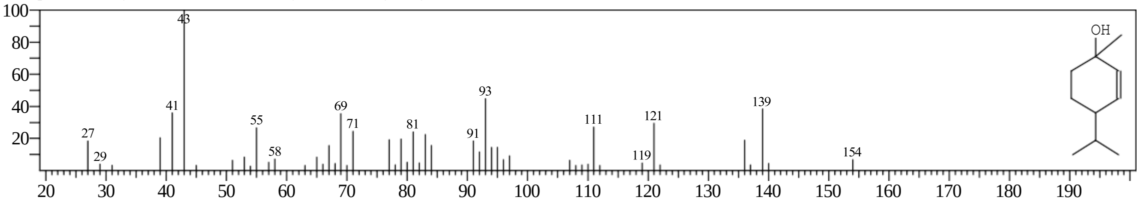

Hit#:4 Entry:33021 Library:NIST107.LIB
SI:81 Formula:C12H20O2 CAS:109010-10-8 MolWeight:196 RetIndex:0
CompName:Isopulegol acetate $$

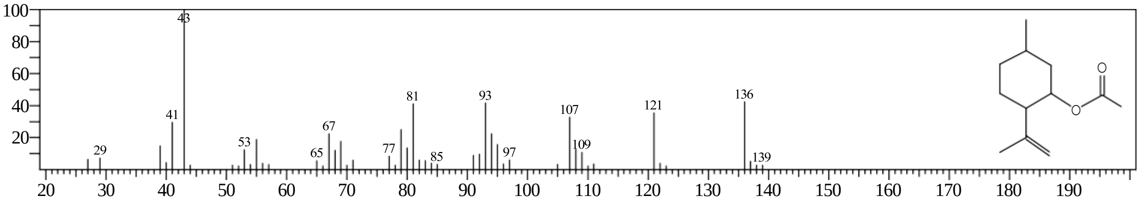

Hit#:5 Entry:15193 Library:NIST107.LIB
SI:81 Formula:C10H18O CAS:7299-41-4 MolWeight:154 RetIndex:0
CompName:Terpineol, Z-.beta.- $$

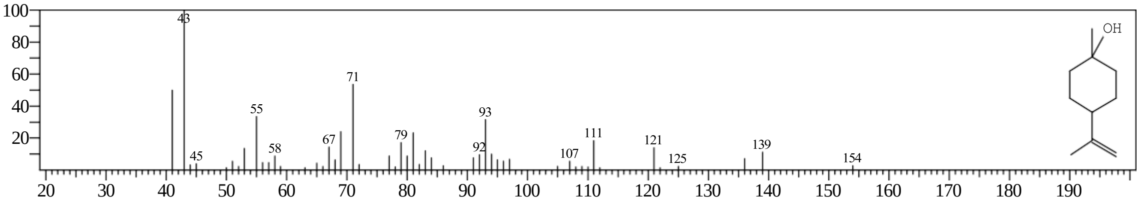

<< Target >>
Line#:48 R.Time:9.685(Scan#:1938) MassPeaks:70
RawMode:Averaged 9.680-9.690(1937-1939) BasePeak:82.15(242809)
BG Mode:Calc. from Peak

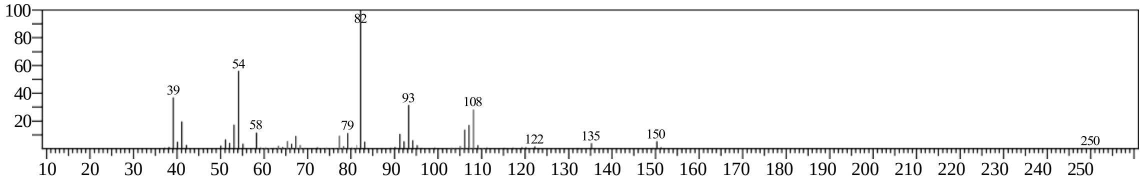

Hit#:1 Entry:13435 Library:NIST107.LIB
SI:95 Formula:C10H14O CAS:99-49-0 MolWeight:150 RetIndex:0
CompName:2-Cyclohexen-1-one, 2-methyl-5-(1-methylethenyl)- $$ p-Mentha-6,8-dien-2-one $$ Carvol $$ Carvone $$ Karvon $$ 1-Carvone $$ .delta.(sup

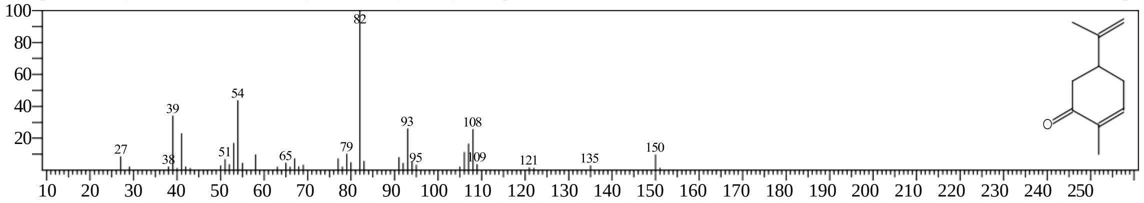

Hit#:2 Entry:13383 Library:NIST107.LIB
SI:94 Formula:C10H14O CAS:2244-16-8 MolWeight:150 RetIndex:0
CompName:2-Cyclohexen-1-one, 2-methyl-5-(1-methylethenyl)-, (S)- $$ p-Mentha-6,8-dien-2-one, (S)-(+)- $$ (+)-Carvone $$ (S)-(+)-Carvone $$ (S)-Carv

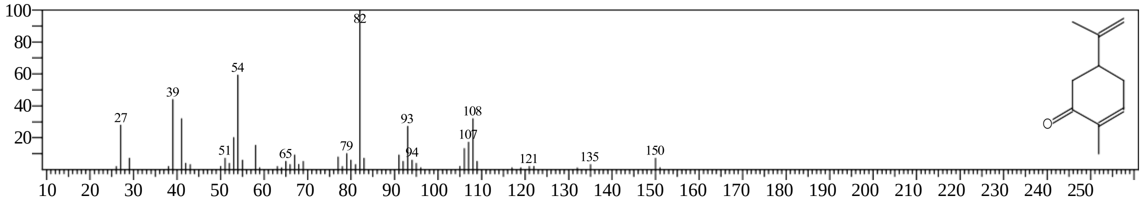

Hit#:3 Entry:13430 Library:NIST107.LIB
SI:93 Formula:C10H14O CAS:6485-40-1 MolWeight:150 RetIndex:0
CompName:2-Cyclohexen-1-one, 2-methyl-5-(1-methylethenyl)-, (R)- $$ p-Mentha-6,8-dien-2-one, (R)-(-)- $$ (-)-(R)-Carvone $$ (-)-p-Mentha-6,8-dien-2-

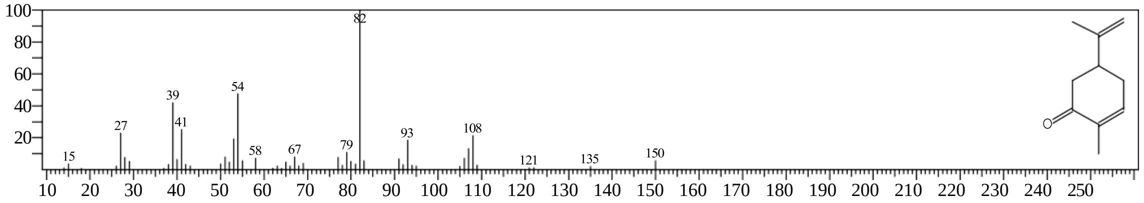

Hit#:4 Entry:13462 Library:NIST107.LIB
SI:84 Formula:C10H14O CAS:16750-82-6 MolWeight:150 RetIndex:0
CompName:2-Cyclohexen-1-one, 3-methyl-6-(1-methylethenyl)-, (S)- $$ p-Mentha-1,8-dien-3-one, (+)- $$ 2-Cyclohexen-1-one, 3-methyl-6-(1-methylethen

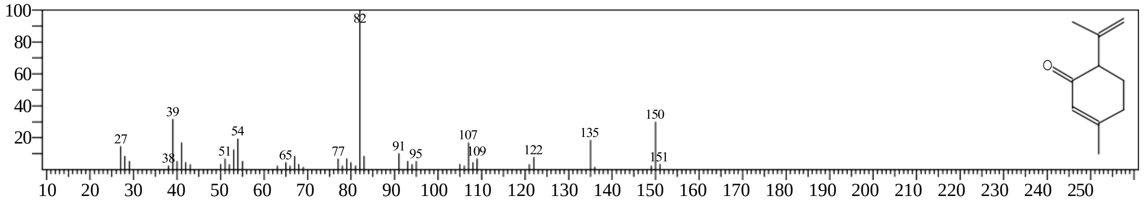

Hit#:5 Entry:8649 Library:NIST107.LIB
SI:79 Formula:C10H16 CAS:0-00-0 MolWeight:136 RetIndex:0
CompName:1,5-Cyclooctadiene, 1,3-dimethyl- $$

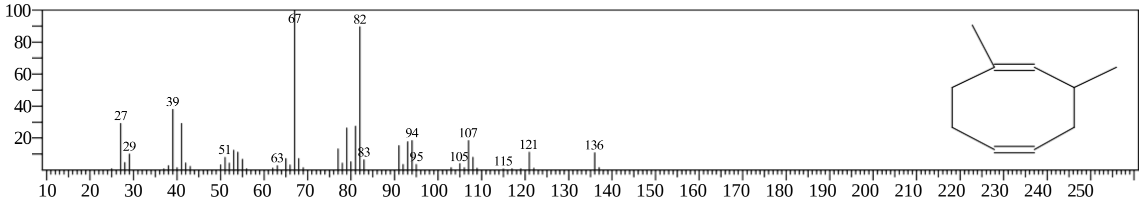

<< Target >>
Line#:49 R.Time:9.735(Scan#:1948) MassPeaks:97
RawMode:Averaged 9.730-9.740(1947-1949) BasePeak:80.15(923030)
BG Mode:Calc. from Peak

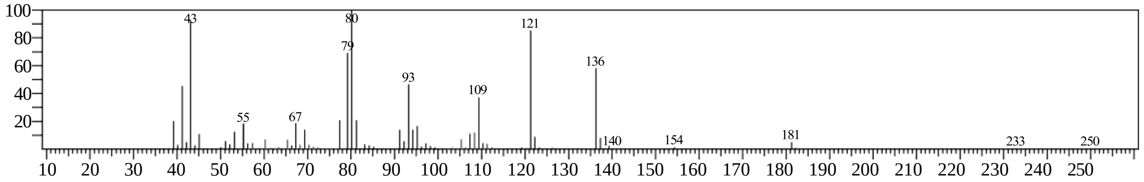

Hit#:1 Entry:33021 Library:NIST107.LIB
SI:84 Formula:C12H20O2 CAS:109010-10-8 MolWeight:196 RetIndex:0
CompName:Isopulegol acetate $$

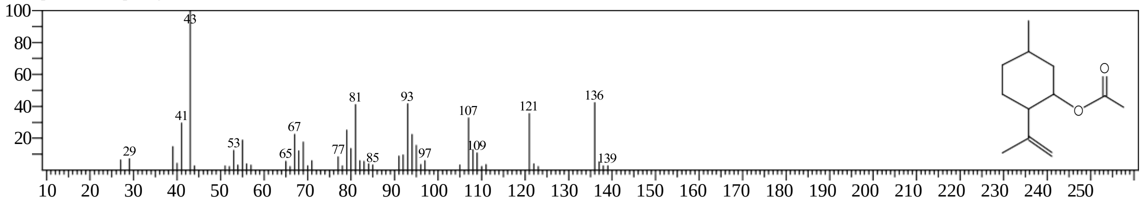

Hit#:2 Entry:32945 Library:NIST107.LIB
SI:83 Formula:C12H20O2 CAS:0-00-0 MolWeight:196 RetIndex:0
CompName:Cyclopentane, 1-acetoxymethyl-3-isopropenyl-2-methyl- $$

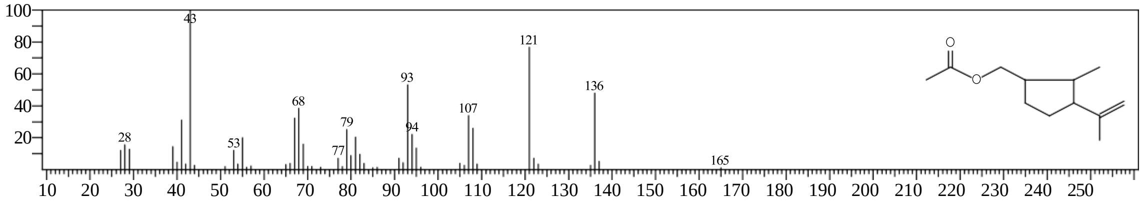

Hit#:3 Entry:32950 Library:NIST107.LIB
SI:83 Formula:C12H20O2 CAS:0-00-0 MolWeight:196 RetIndex:0
CompName:.alpha.-Cyclogeraniol acetate $$

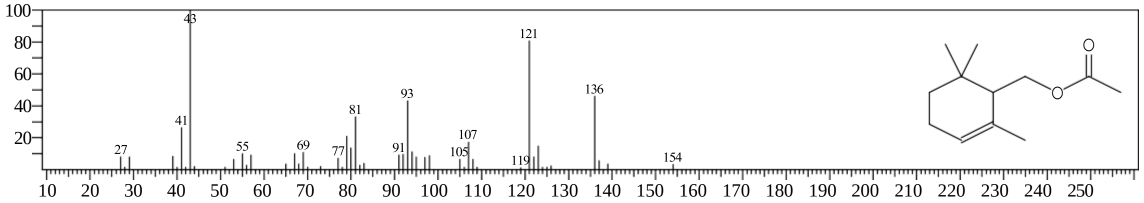

Hit#:4 Entry:33010 Library:NIST107.LIB
SI:82 Formula:C12H20O2 CAS:80-26-2 MolWeight:196 RetIndex:0
CompName:3-Cyclohexene-1-methanol, .alpha.,.alpha.,4-trimethyl-, acetate $$ p-Menth-1-en-8-ol, acetate $$ .alpha.-Terpineol acetate $$ .alpha.-Terpinyl ac

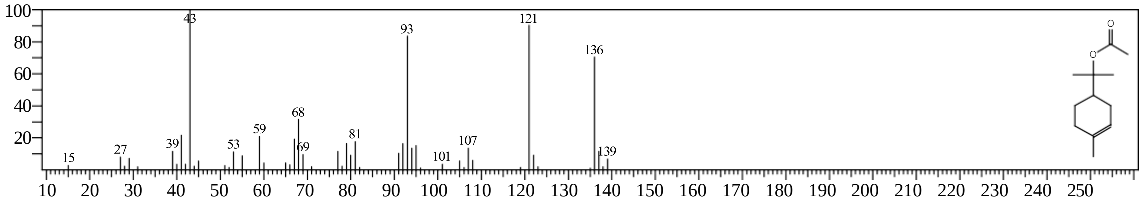

Hit#:5 Entry:15171 Library:NIST107.LIB
SI:82 Formula:C10H18O CAS:15714-11-1 MolWeight:154 RetIndex:0
CompName:p-Menth-4(8)-en-9-ol $$

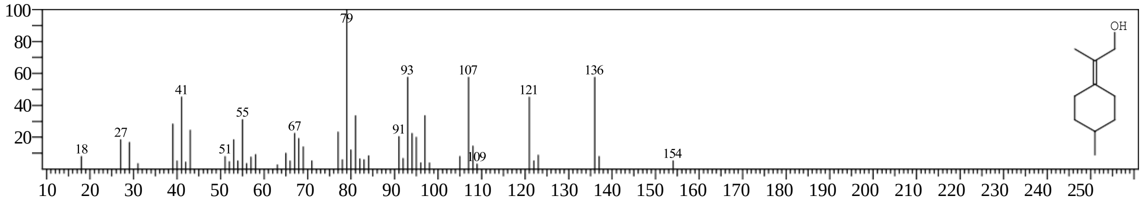

<< Target >>
Line#:50 R.Time:9.830(Scan#:1967) MassPeaks:97
RawMode:Averaged 9.825-9.835(1966-1968) BasePeak:108.25(616676)
BG Mode:Calc. from Peak

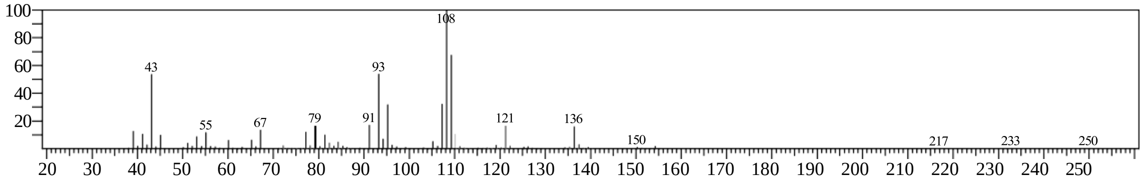

Hit#:1 Entry:5830 Library:NIST107.LIB
SI:82 Formula:C8H14O CAS:0-00-0 MolWeight:126 RetIndex:0
CompName:3-Cyclohexene-1-methanol, 6-methyl- $$

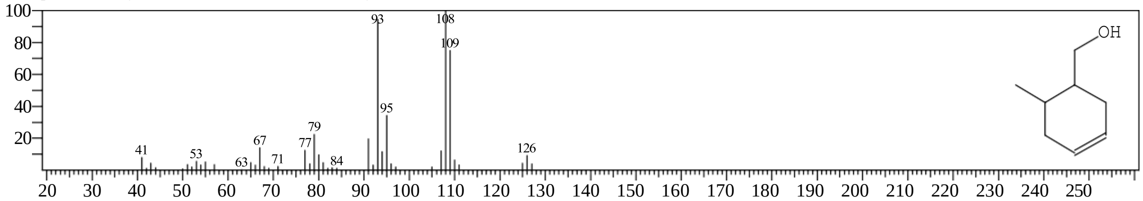

Hit#:2 Entry:20865 Library:NIST107.LIB
SI:81 Formula:C10H16O2 CAS:5688-48-2 MolWeight:168 RetIndex:0
CompName:3-Cyclohexene-1-carboxylic acid, 3,4-dimethyl-, methyl ester $$

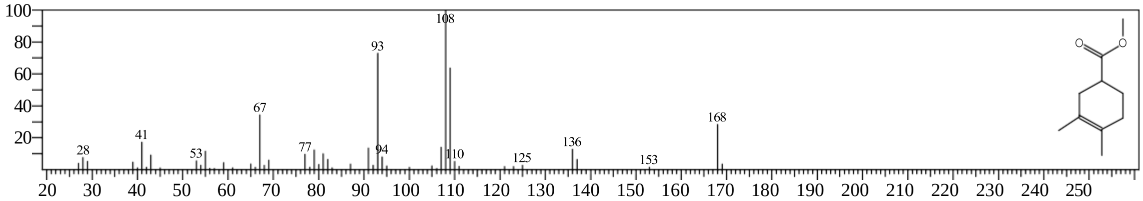

Hit#:3 Entry:14233 Library:NIST107.LIB
SI:81 Formula:C10H16O CAS:4501-58-0 MolWeight:152 RetIndex:0
CompName:3-Cyclopentene-1-acetaldehyde, 2,2,3-trimethyl- $$ .alpha.-Campholenal $$ .alpha.-Campholenic aldehyde $$ 2,2,3-Trimethyl-3-cyclopentaceta

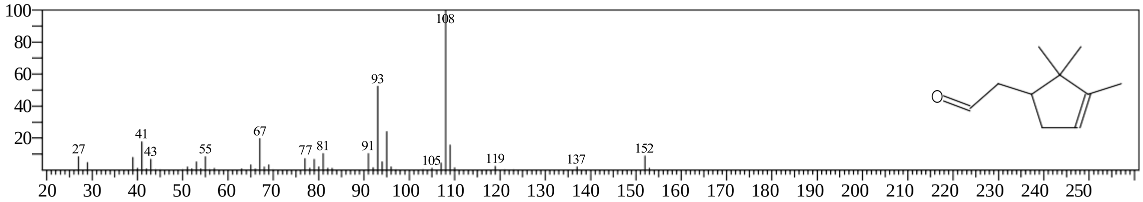

Hit#:4 Entry:14166 Library:NIST107.LIB
SI:81 Formula:C10H16O CAS:0-00-0 MolWeight:152 RetIndex:0
CompName:1,7,7-Trimethylbicyclo[2.2.1]hept-5-en-2-ol $$

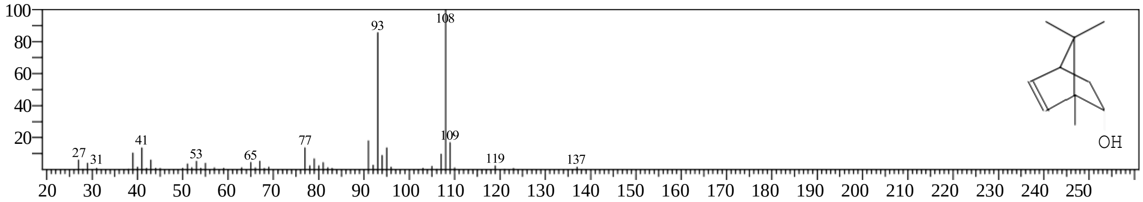

Hit#:5 Entry:38194 Library:NIST107.LIB
SI:80 Formula:C13H20O2 CAS:52210-15-8 MolWeight:208 RetIndex:0
CompName:2-Cyclohexen-1-one, 4-(3-hydroxy-1-butenyl)-3,5,5-trimethyl-, [r-[r@,R@-(E)]]- $$

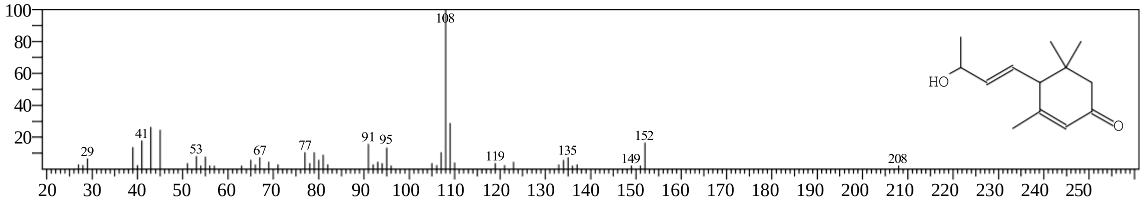

<< Target >>
Line#:51 R.Time:9.865(Scan#:1974) MassPeaks:57
RawMode:Averaged 9.860-9.870(1973-1975) BasePeak:41.15(297250)
BG Mode:Calc. from Peak

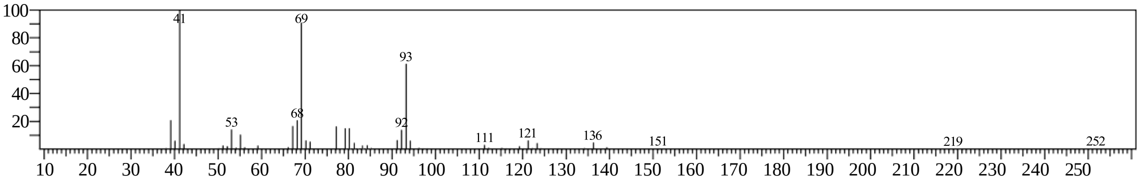

Hit#:1 Entry:15115 Library:NIST107.LIB
SI:90 Formula:C10H18O CAS:106-25-2 MolWeight:154 RetIndex:0
CompName:2,6-Octadien-1-ol, 3,7-dimethyl-, (Z)- $$ cis-Geraniol $$ Geranyl Alcohol $$ cis-3,7-Dimethyl-2,6-octadien-1-ol $$ Nerol $$ Neryl alcohol $$

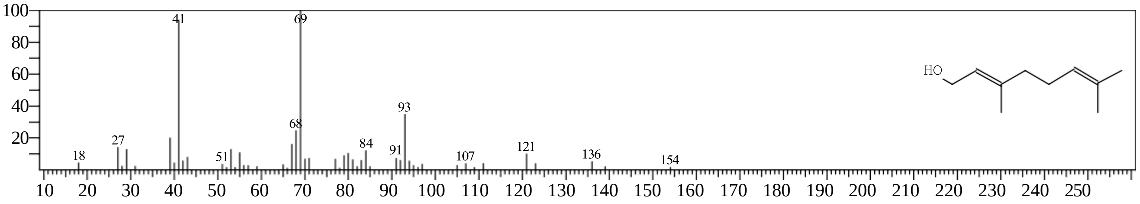

Hit#:2 Entry:8783 Library:NIST107.LIB
SI:89 Formula:C10H16 CAS:123-35-3 MolWeight:136 RetIndex:0
CompName:.beta.-Myrcene $$ 1,6-Octadiene, 7-methyl-3-methylene- $$ Myrcene $$ 7-Methyl-3-methylene-1,6-octadiene $$ 7-Methyl-3-methyleneoctadie

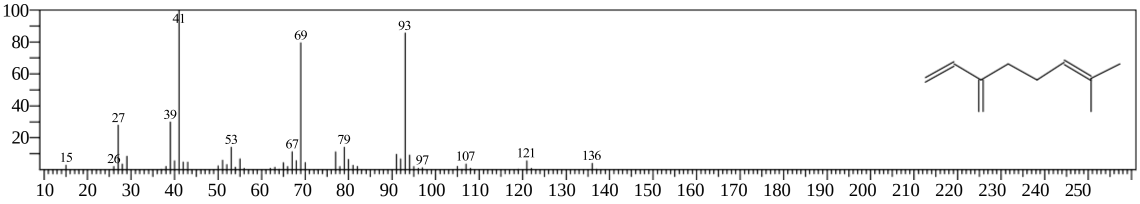

Hit#:3 Entry:41288 Library:NIST107.LIB
SI:87 Formula:C10H17Br CAS:35719-26-7 MolWeight:216 RetIndex:0
CompName:Geranyl bromide $$

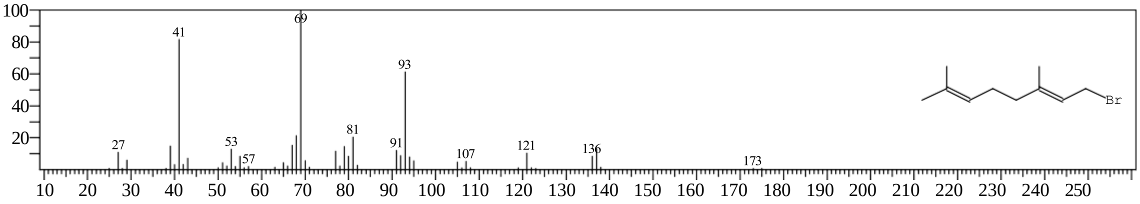

Hit#:4 Entry:32145 Library:NIST107.LIB
SI:84 Formula:C13H22O CAS:139694-23-8 MolWeight:194 RetIndex:0
CompName:2,6-Octadiene, 3,7-dimethyl-1-(2-propenyloxy)- $$

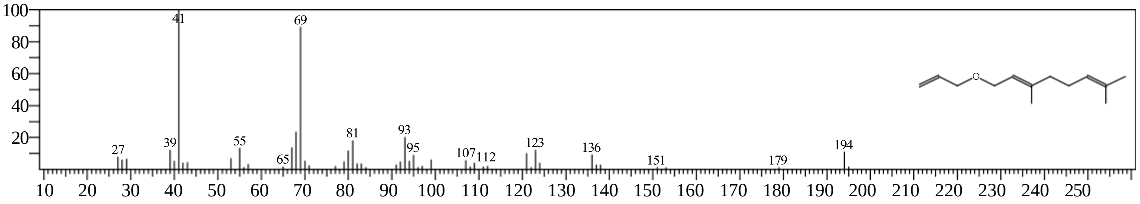

Hit#:5 Entry:15311 Library:NIST107.LIB
SI:83 Formula:C10H18O CAS:22410-74-8 MolWeight:154 RetIndex:0
CompName:2,6-Octadien-1-ol, 2,7-dimethyl- $$

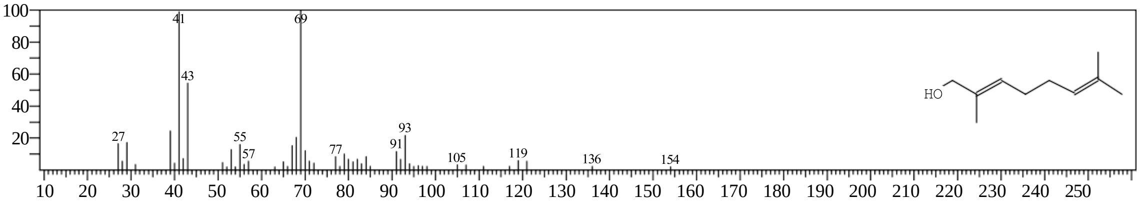

<< Target >>
Line#:52 R.Time:9.895(Scan#:1980) MassPeaks:54
RawMode:Averaged 9.890-9.900(1979-1981) BasePeak:91.15(475758)
BG Mode:Calc. from Peak

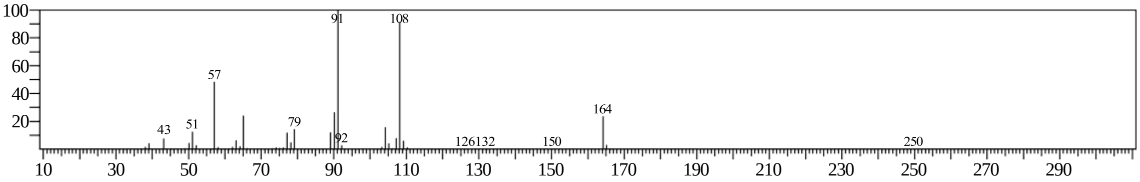

Hit#:1 Entry:18874 Library:NIST107.LIB
SI:92 Formula:C10H12O2 CAS:122-63-4 MolWeight:164 RetIndex:0
CompName:Propanoic acid, phenylmethyl ester $$ Propionic acid, benzyl ester $$ Benzyl propionate $$ Benzyl propionate fcc $$

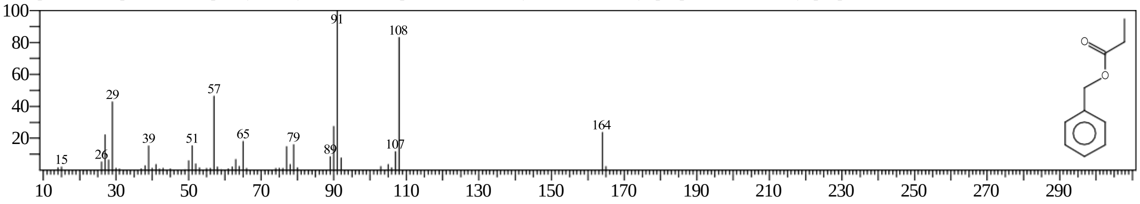

Hit#:2 Entry:19350 Library:NIST107.LIB
SI:83 Formula:C9H11NO2 CAS:30379-59-0 MolWeight:165 RetIndex:0
CompName:Carbamic acid, methyl-, phenylmethyl ester $$

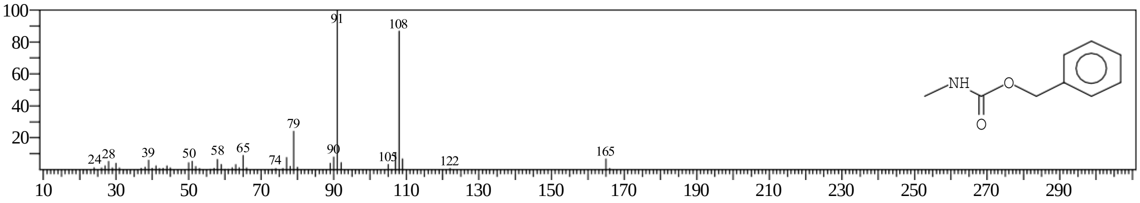

Hit#:3 Entry:25884 Library:NIST107.LIB
SI:81 Formula:C10H12O3 CAS:2051-96-9 MolWeight:180 RetIndex:0
CompName:Benzyl 3-hydroxypropanoate $$ Propanoic acid, 2-hydroxy-, phenylmethyl ester $$

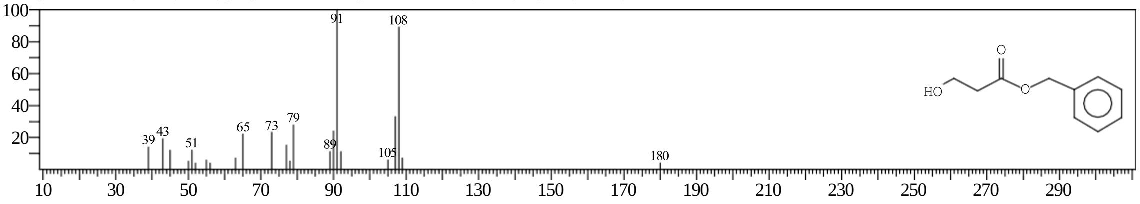

Hit#:4 Entry:38473 Library:NIST107.LIB
SI:81 Formula:C11H15NO3 CAS:0-00-0 MolWeight:209 RetIndex:0
CompName:N-CBZ-propan-3-ol-1-amine $$

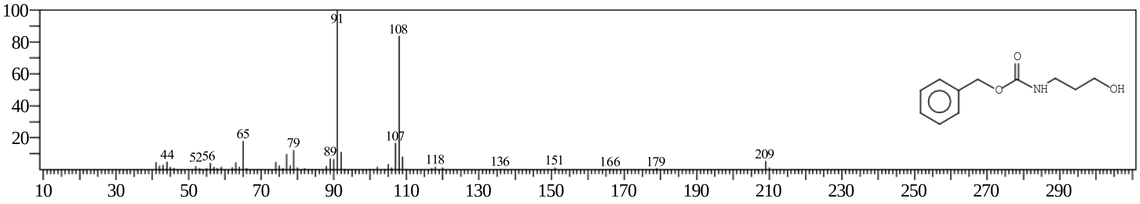

Hit#:5 Entry:73053 Library:NIST107.LIB
SI:81 Formula:C13H14F3NO4 CAS:0-00-0 MolWeight:305 RetIndex:0
CompName:N-(Benzyloxycarbonyl)-3,3,3-trifluoroalanine ethyl ester $$

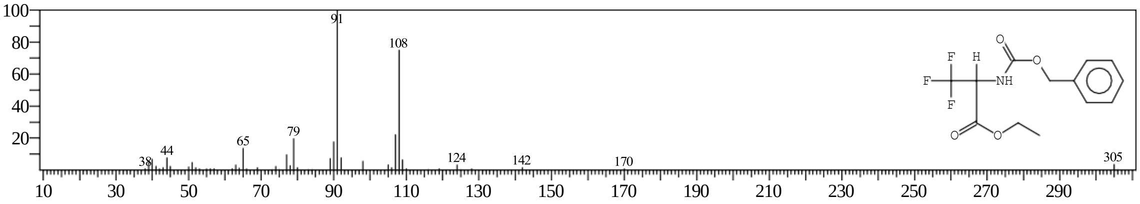

<< Target >>
Line#:53 R.Time:9.990(Scan#:1999) MassPeaks:108
RawMode:Averaged 9.985-9.995(1998-2000) BasePeak:95.20(633749)
BG Mode:Calc. from Peak

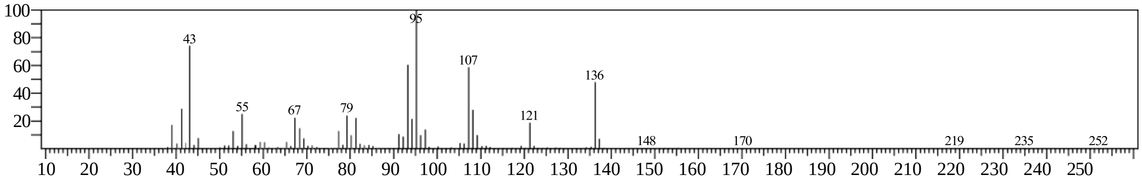

Hit#:1 Entry:33021 Library:NIST107.LIB
SI:87 Formula:C12H20O2 CAS:109010-10-8 MolWeight:196 RetIndex:0
CompName:Isopulegol acetate $$

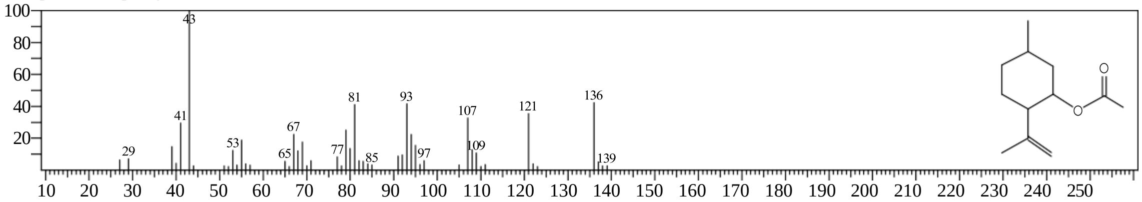

Hit#:2 Entry:33022 Library:NIST107.LIB
SI:86 Formula:C12H20O2 CAS:125-12-2 MolWeight:196 RetIndex:0
CompName:Isobornyl acetate $$ Bicyclo[2.2.1]heptan-2-ol, 1,7,7-trimethyl-, acetate, exo- $$ Isoborneol, acetate $$ Acetic acid, isobornyl ester $$ Pichtosin

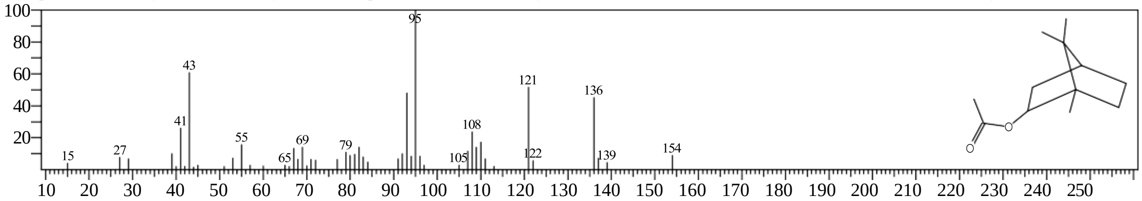

Hit#:3 Entry:26943 Library:NIST107.LIB
SI:86 Formula:C11H18O2 CAS:1200-67-5 MolWeight:182 RetIndex:0
CompName:Isobornyl formate $$ Bicyclo[2.2.1]heptan-2-ol, 1,7,7-trimethyl-, formate, exo- $$

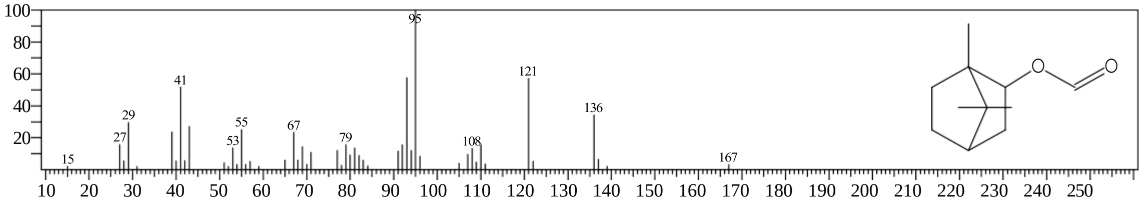

Hit#:4 Entry:32945 Library:NIST107.LIB
SI:85 Formula:C12H20O2 CAS:0-00-0 MolWeight:196 RetIndex:0
CompName:Cyclopentane, 1-acetoxymethyl-3-isopropenyl-2-methyl- $$

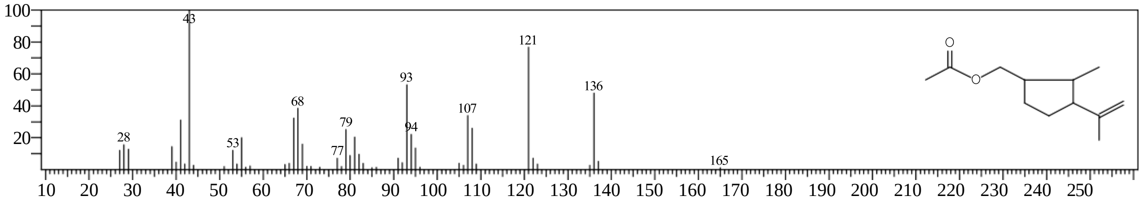

Hit#:5 Entry:33030 Library:NIST107.LIB
SI:85 Formula:C12H20O2 CAS:10198-23-9 MolWeight:196 RetIndex:0
CompName:Cyclohexanol, 1-methyl-4-(1-methylethenyl)-, acetate $$ p-Menth-8-en-1-ol, acetate $$ .beta.-Terpinyl acetate $$

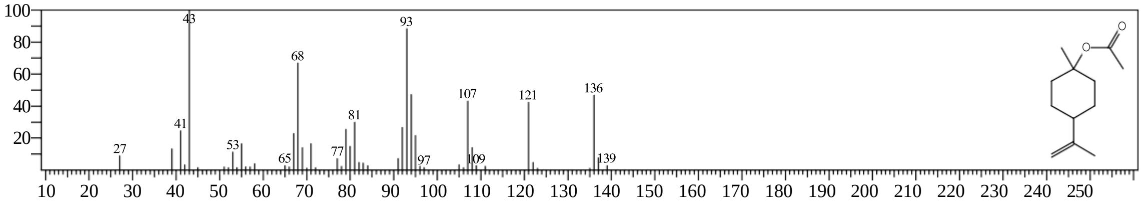

<< Target >>
Line#:54 R.Time:10.145(Scan#:2030) MassPeaks:61
RawMode:Averaged 10.140-10.150(2029-2031) BasePeak:41.15(342973)
BG Mode:Calc. from Peak

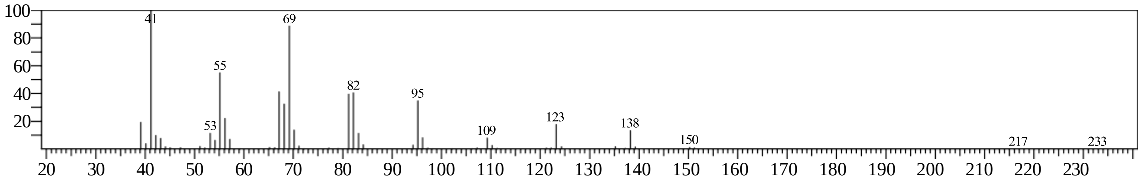

Hit#:1 Entry:16160 Library:NIST107.LIB
SI:93 Formula:C10H20O CAS:106-22-9 MolWeight:156 RetIndex:0
CompName:6-Octen-1-ol, 3,7-dimethyl- $$ .beta.-Citronellol $$ Cephrol $$ Citronellol $$ Rodinol $$ 3,7-Dimethyl-6-octen-1-ol $$ Elenol $$ RHODINOL

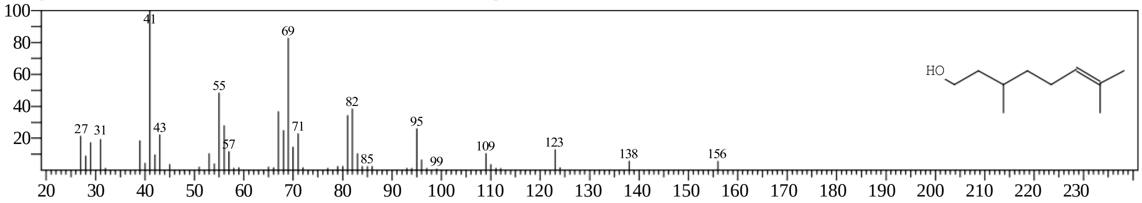

Hit#:2 Entry:16139 Library:NIST107.LIB
SI:92 Formula:C10H20O CAS:1117-61-9 MolWeight:156 RetIndex:0
CompName:6-Octen-1-ol, 3,7-dimethyl-, (R)- $$ (R)-(+)-.beta.-Citronellol $$

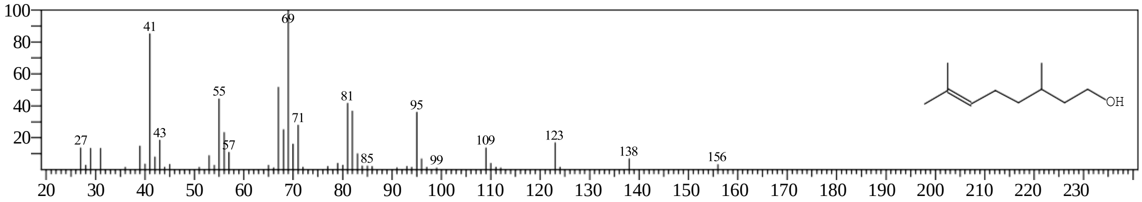

Hit#:3 Entry:27805 Library:NIST107.LIB
SI:91 Formula:C11H20O2 CAS:105-85-1 MolWeight:184 RetIndex:0
CompName:6-Octen-1-ol, 3,7-dimethyl-, formate $$ Citronellyl formate $$ Formic acid, citronellyl ester $$ Formic acid, 3,7-dimethyl-6-octen-1-yl ester $$

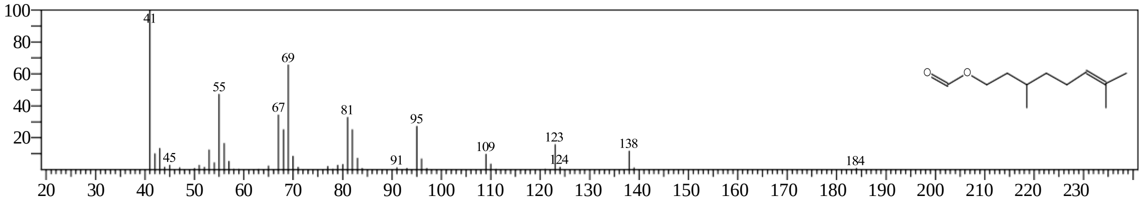

Hit#:4 Entry:9344 Library:NIST107.LIB
SI:91 Formula:C10H18 CAS:62238-28-2 MolWeight:138 RetIndex:0
CompName:1,6-Heptadiene, 2,5,5-trimethyl- $$

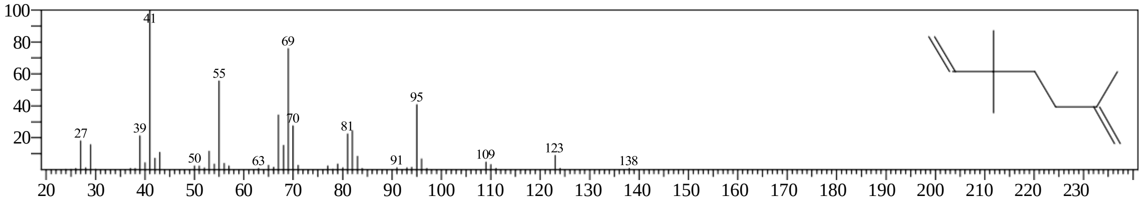

Hit#:5 Entry:9301 Library:NIST107.LIB
SI:90 Formula:C10H18 CAS:0-00-0 MolWeight:138 RetIndex:0
CompName:3,7-Dimethyl-octa-1,6-diene $$

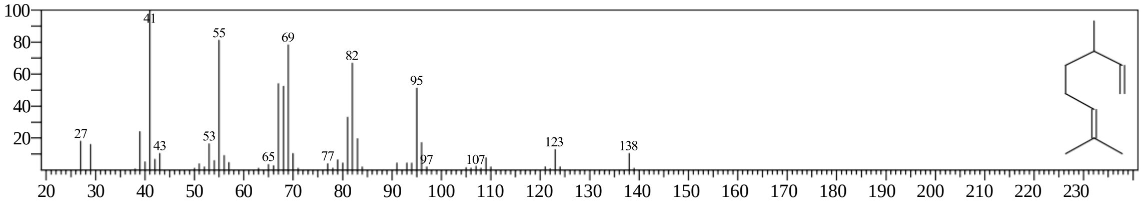

<< Target >>
Line#:55 R.Time:10.390(Scan#:2079) MassPeaks:137
RawMode:Averaged 10.385-10.395(2078-2080) BasePeak:57.15(8375994)
BG Mode:Calc. from Peak

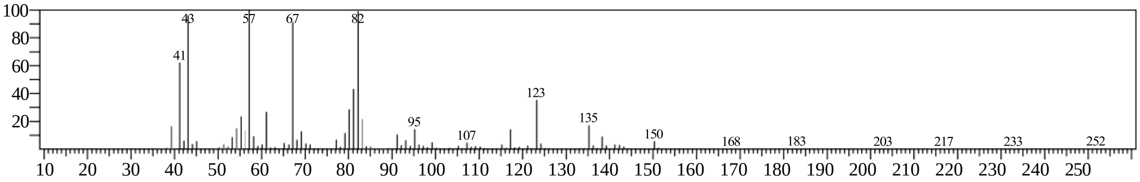

Hit#:1 Entry:33888 Library:NIST107.LIB
SI:86 Formula:C12H22O2 CAS:32210-23-4 MolWeight:198 RetIndex:0
CompName:4-tert-Butylcyclohexyl acetate $$ (4-tert-Butylcyclohexyl)acetate,c&t $$ Acetic acid p-tert-butylcyclohexyl ester,c&t $$ Cyclohexanol, 4-(1,1-d

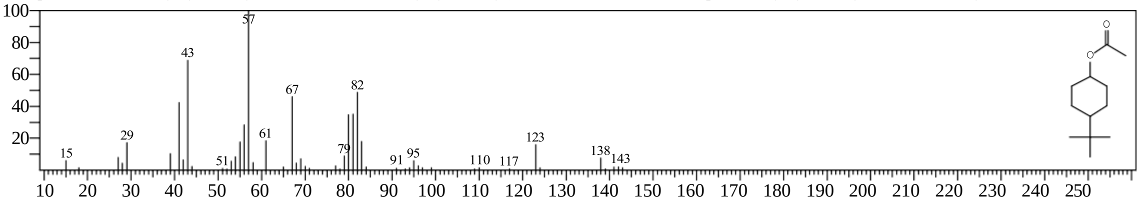

Hit#:2 Entry:16101 Library:NIST107.LIB
SI:82 Formula:C10H20O CAS:13491-79-7 MolWeight:156 RetIndex:0
CompName:Cyclohexanol, 2-(1,1-dimethylethyl)- $$ Cyclohexanol, 2-tert-butyl- $$ o-tert-Butylcyclohexanol $$ 2-tert-Butylcyclohexanol $$

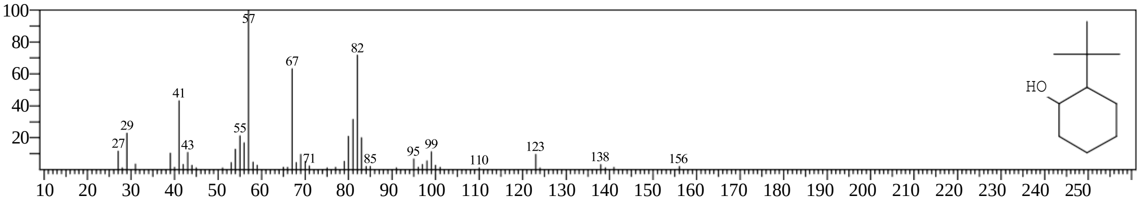

Hit#:3 Entry:16150 Library:NIST107.LIB
SI:80 Formula:C10H20O CAS:98-52-2 MolWeight:156 RetIndex:0
CompName:Cyclohexanol, 4-(1,1-dimethylethyl)- $$ Cyclohexanol, 4-tert-butyl- $$ p-tert-Butylcyclohexanol $$ 4-tert-Butylcyclohexanol $$ 4-tert-Butylcy

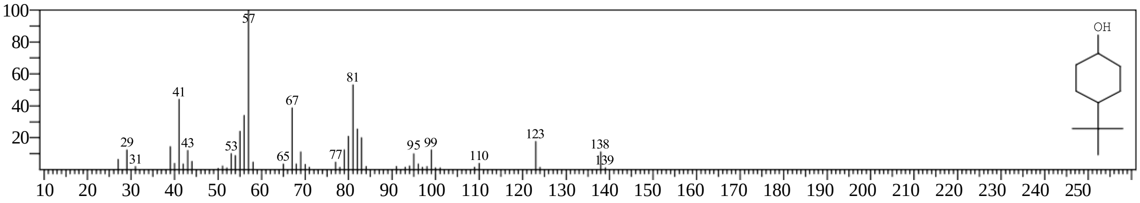

Hit#:4 Entry:9375 Library:NIST107.LIB
SI:80 Formula:C10H18 CAS:89656-98-4 MolWeight:138 RetIndex:0
CompName:Cyclohexane, (2-methyl-1-propenyl)- $$

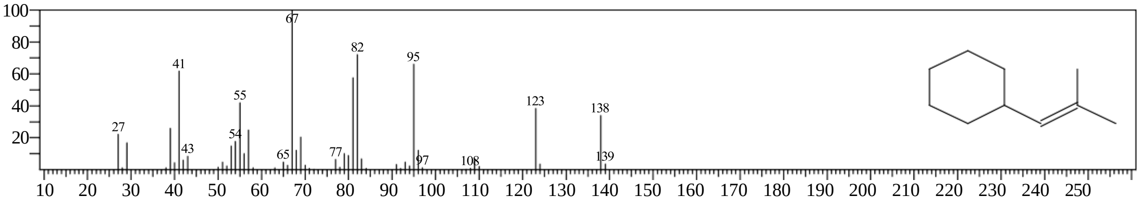

Hit#:5 Entry:23479 Library:NIST107.LIB
SI:78 Formula:C10H22O2 CAS:19781-07-8 MolWeight:174 RetIndex:0
CompName:2,7-Dimethyl-2,7-octanediol $$

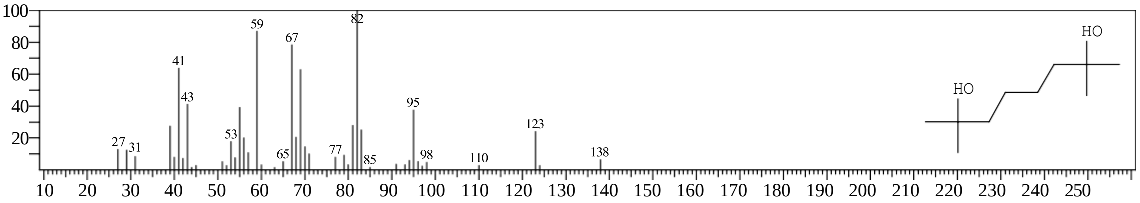

<< Target >>
Line#:56 R.Time:10.545(Scan#:2110) MassPeaks:113
RawMode:Averaged 10.540-10.550(2109-2111) BasePeak:43.10(261314)
BG Mode:Calc. from Peak

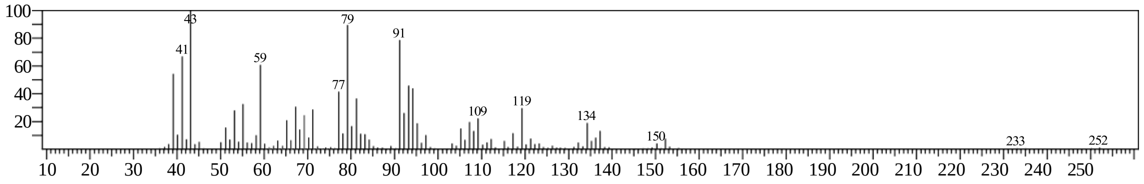

Hit#:1 Entry:14319 Library:NIST107.LIB
SI:84 Formula:C10H16O CAS:1820-09-3 MolWeight:152 RetIndex:0
CompName:Bicyclo[3.1.1]hept-3-en-2-ol, 4,6,6-trimethyl-, (1.alpha.,2.alpha.,5.alpha.)- $$ 2-Pinen-4-ol, trans- $$ trans-Verbenol $$

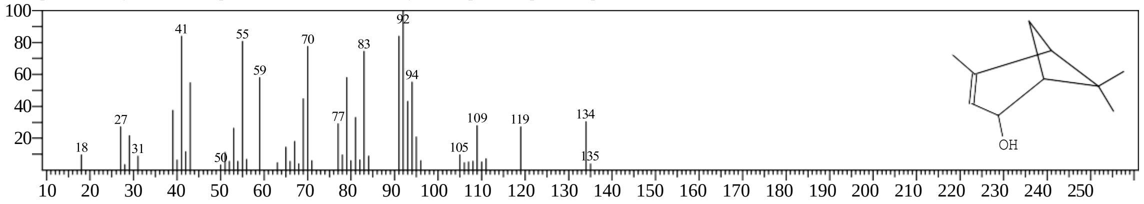

Hit#:2 Entry:14198 Library:NIST107.LIB
SI:83 Formula:C10H16O CAS:18881-04-4 MolWeight:152 RetIndex:0
CompName:Bicyclo[3.1.1]hept-3-en-2-ol, 4,6,6-trimethyl-, [1S-(1.alpha.,2.beta.,5.alpha.)]- $$ (S)-cis-Verbenol $$

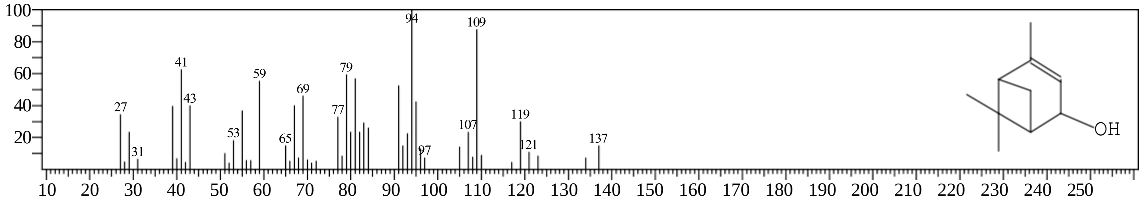

Hit#:3 Entry:14261 Library:NIST107.LIB
SI:81 Formula:C10H16O CAS:7212-40-0 MolWeight:152 RetIndex:0
CompName:2-Cyclohexen-1-ol, 1-methyl-4-(1-methylethenyl)-, trans- $$

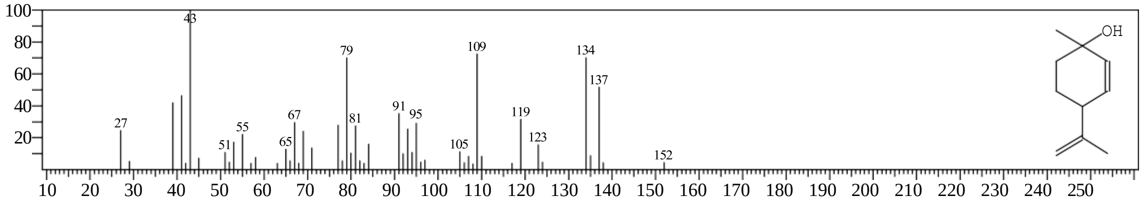

Hit#:4 Entry:14135 Library:NIST107.LIB
SI:81 Formula:C10H16O CAS:0-00-0 MolWeight:152 RetIndex:0
CompName:trans-p-Mentha-2,8-dienol $$

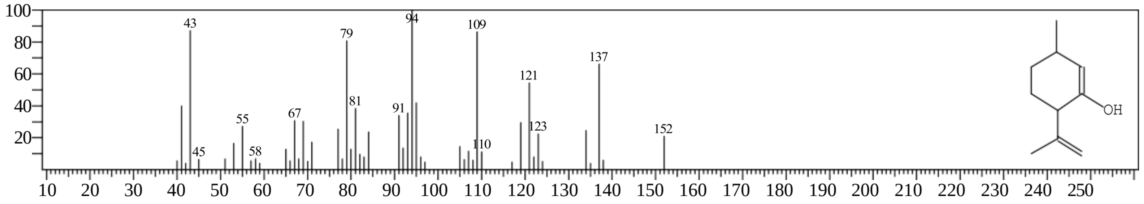

Hit#:5 Entry:14213 Library:NIST107.LIB
SI:81 Formula:C10H16O CAS:35907-10-9 MolWeight:152 RetIndex:0
CompName:Cyclohexanol, 2-methylene-5-(1-methylethenyl)- $$

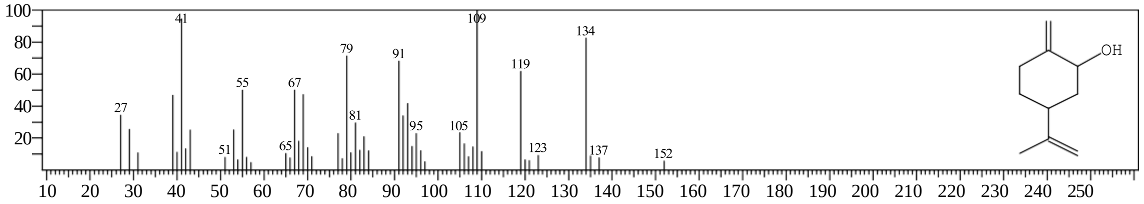

<< Target >>
Line#:57 R.Time:10.635(Scan#:2128) MassPeaks:112
RawMode:Averaged 10.630-10.640(2127-2129) BasePeak:57.15(2864312)
BG Mode:Calc. from Peak

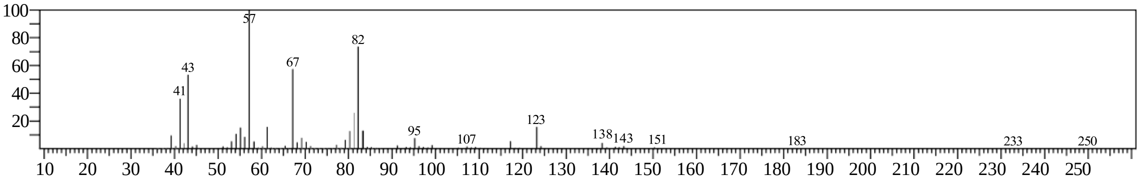

Hit#:1 Entry:33888 Library:NIST107.LIB
SI:92 Formula:C12H22O2 CAS:32210-23-4 MolWeight:198 RetIndex:0
CompName:4-tert-Butylcyclohexyl acetate $$ (4-tert-Butylcyclohexyl)acetate,c&t $$ Acetic acid p-tert-butylcyclohexyl ester,c&t $$ Cyclohexanol, 4-(1,1-d

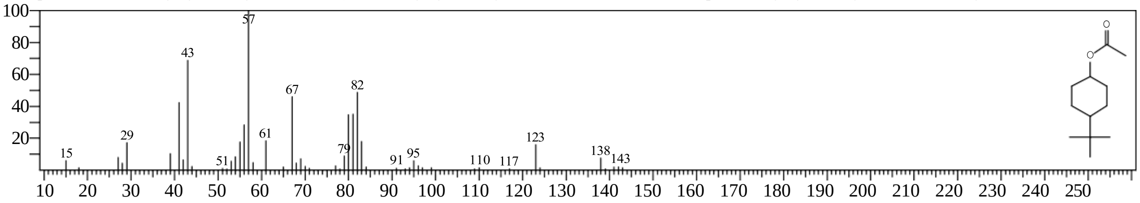

Hit#:2 Entry:16101 Library:NIST107.LIB
SI:90 Formula:C10H20O CAS:13491-79-7 MolWeight:156 RetIndex:0
CompName:Cyclohexanol, 2-(1,1-dimethylethyl)- $$ Cyclohexanol, 2-tert-butyl- $$ o-tert-Butylcyclohexanol $$ 2-tert-Butylcyclohexanol $$

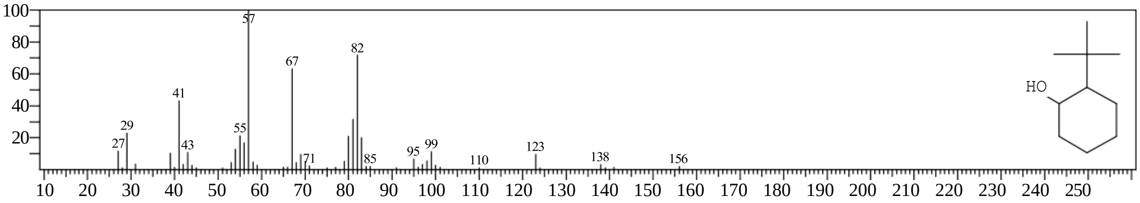

Hit#:3 Entry:9423 Library:NIST107.LIB
SI:84 Formula:C10H18 CAS:2228-98-0 MolWeight:138 RetIndex:0
CompName:Cyclohexene, 4-(1,1-dimethylethyl)- $$ Cyclohexene, 4-tert-butyl- $$ 4-tert-Butyl-1-cyclohexene $$ 4-tert-Butylcyclohexene $$

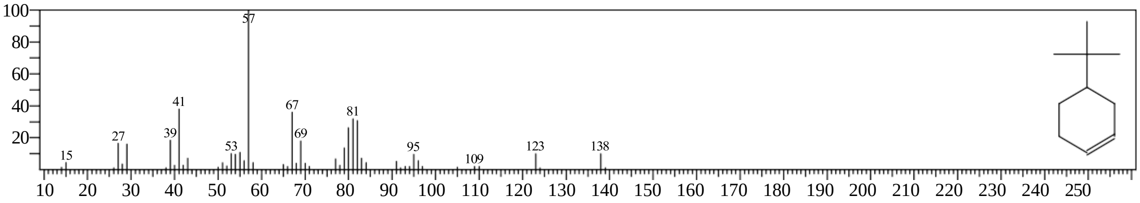

Hit#:4 Entry:16150 Library:NIST107.LIB
SI:84 Formula:C10H20O CAS:98-52-2 MolWeight:156 RetIndex:0
CompName:Cyclohexanol, 4-(1,1-dimethylethyl)- $$ Cyclohexanol, 4-tert-butyl- $$ p-tert-Butylcyclohexanol $$ 4-tert-Butylcyclohexanol $$ 4-tert-Butylcy

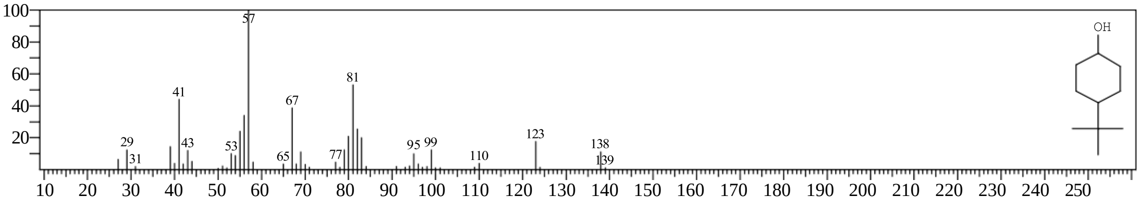

Hit#:5 Entry:16104 Library:NIST107.LIB
SI:83 Formula:C10H20O CAS:937-05-3 MolWeight:156 RetIndex:0
CompName:Cyclohexanol, 4-(1,1-dimethylethyl)-, cis- $$ Cyclohexanol, 4-tert-butyl-, cis- $$ cis-4-tert-Butylcyclohexanol $$

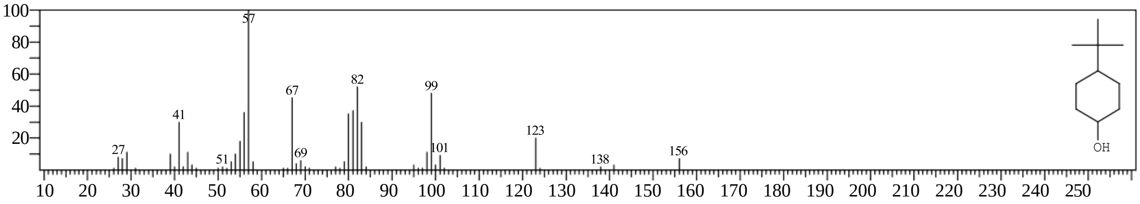

<< Target >>
Line#:58 R.Time:10.765(Scan#:2154) MassPeaks:123
RawMode:Averaged 10.760-10.770(2153-2155) BasePeak:43.10(131720)
BG Mode:Calc. from Peak

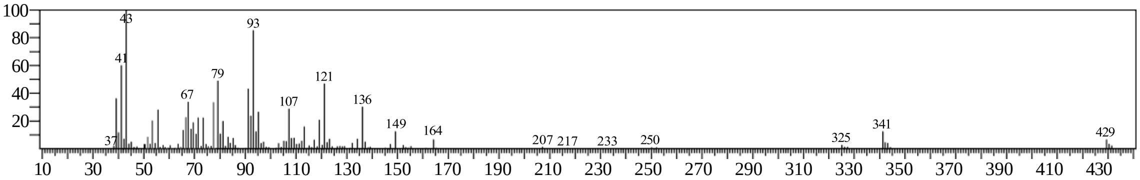

Hit#:1 Entry:63070 Library:NIST107.LIB
SI:79 Formula:C17H23NO2 CAS:7149-26-0 MolWeight:273 RetIndex:0
CompName:1,6-Octadien-3-ol, 3,7-dimethyl-, 2-aminobenzoate $$ Anthranilic acid, 1,5-dimethyl-1-vinyl-4-hexenyl ester $$ Linalyl anthranilate $$ Anthrani

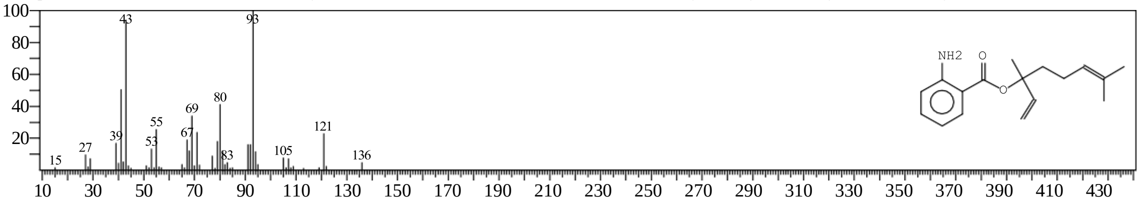

Hit#:2 Entry:8688 Library:NIST107.LIB
SI:79 Formula:C10H16 CAS:16022-04-1 MolWeight:136 RetIndex:0
CompName:Bicyclo[3.1.1]heptane, 6,6-dimethyl-3-methylene- $$ Norpinane, 6,6-dimethyl-3-methylene- $$

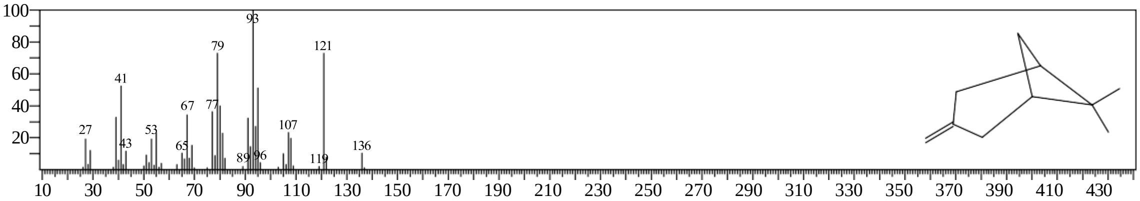

Hit#:3 Entry:8740 Library:NIST107.LIB
SI:79 Formula:C10H16 CAS:42123-66-0 MolWeight:136 RetIndex:0
CompName:1,3,6-Heptatriene, 2,5,6-trimethyl- $$

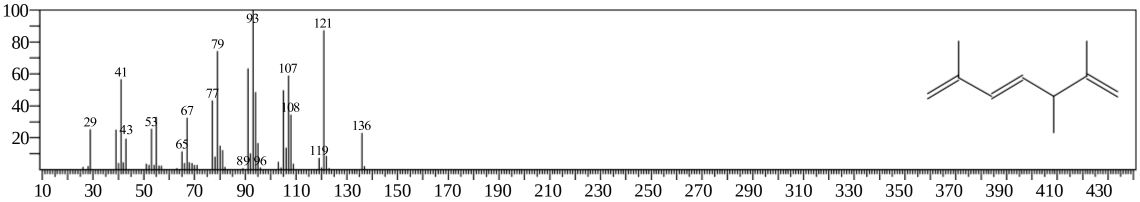

Hit#:4 Entry:32945 Library:NIST107.LIB
SI:79 Formula:C12H20O2 CAS:0-00-0 MolWeight:196 RetIndex:0
CompName:Cyclopentane, 1-acetoxymethyl-3-isopropenyl-2-methyl- $$

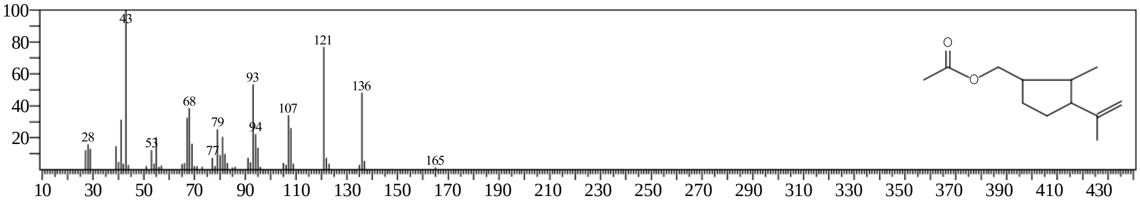

Hit#:5 Entry:8732 Library:NIST107.LIB
SI:79 Formula:C10H16 CAS:13837-95-1 MolWeight:136 RetIndex:0
CompName:Cyclohexane, 1-methylene-3-(1-methylethenyl)-, (R)- $$ m-Mentha-1(7),8-diene, (R)-(-)- $$

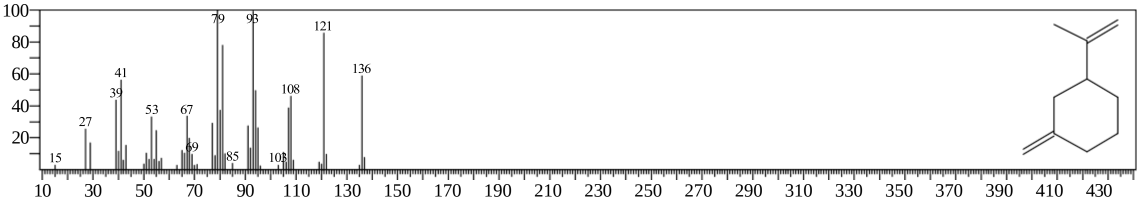

<< Target >>
Line#:59 R.Time:10.845(Scan#:2170) MassPeaks:111
RawMode:Averaged 10.840-10.850(2169-2171) BasePeak:43.10(925140)
BG Mode:Calc. from Peak

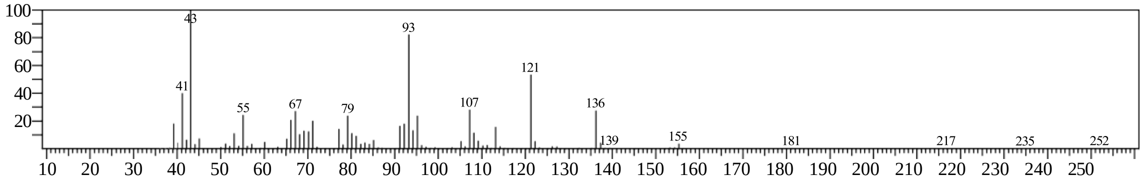

Hit#:1 Entry:33021 Library:NIST107.LIB
SI:87 Formula:C12H20O2 CAS:109010-10-8 MolWeight:196 RetIndex:0
CompName:Isopulegol acetate $$

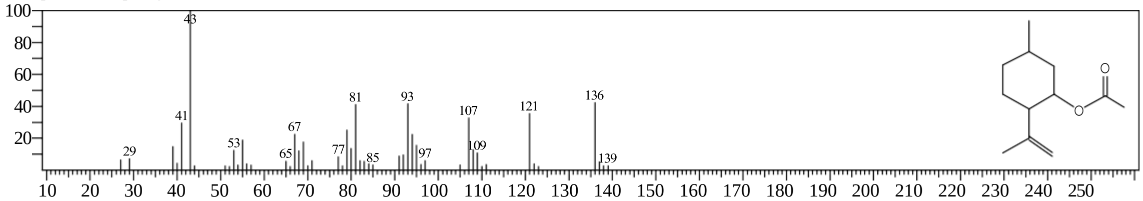

Hit#:2 Entry:63070 Library:NIST107.LIB
SI:87 Formula:C17H23NO2 CAS:7149-26-0 MolWeight:273 RetIndex:0
CompName:1,6-Octadien-3-ol, 3,7-dimethyl-, 2-aminobenzoate $$ Anthranilic acid, 1,5-dimethyl-1-vinyl-4-hexenyl ester $$ Linalyl anthranilate $$ Anthrani

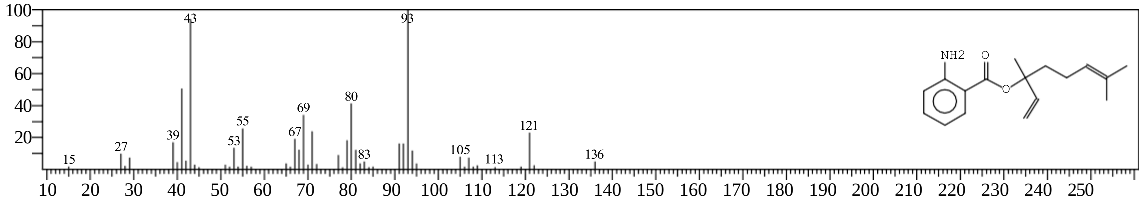

Hit#:3 Entry:33030 Library:NIST107.LIB
SI:87 Formula:C12H20O2 CAS:10198-23-9 MolWeight:196 RetIndex:0
CompName:Cyclohexanol, 1-methyl-4-(1-methylethenyl)-, acetate $$ p-Menth-8-en-1-ol, acetate $$ .beta.-Terpinyl acetate $$

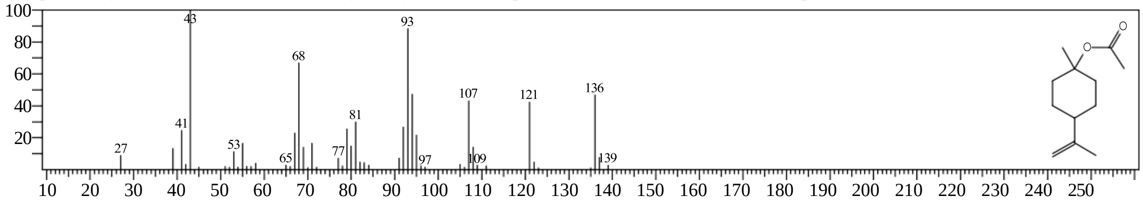

Hit#:4 Entry:32945 Library:NIST107.LIB
SI:86 Formula:C12H20O2 CAS:0-00-0 MolWeight:196 RetIndex:0
CompName:Cyclopentane, 1-acetoxymethyl-3-isopropenyl-2-methyl- $$

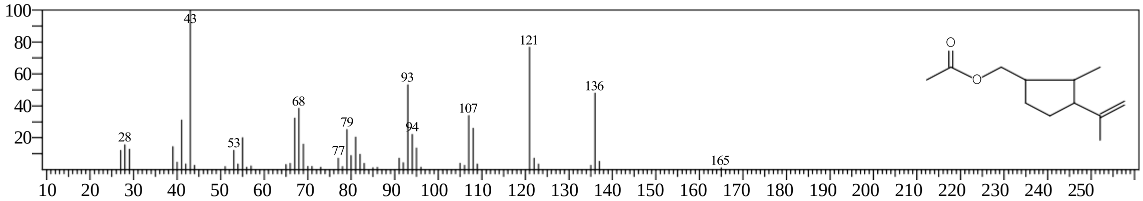

Hit#:5 Entry:33018 Library:NIST107.LIB
SI:85 Formula:C12H20O2 CAS:29021-36-1 MolWeight:196 RetIndex:0
CompName:Bicyclo[3.1.1]heptane-2-methanol, 6,6-dimethyl-, acetate $$

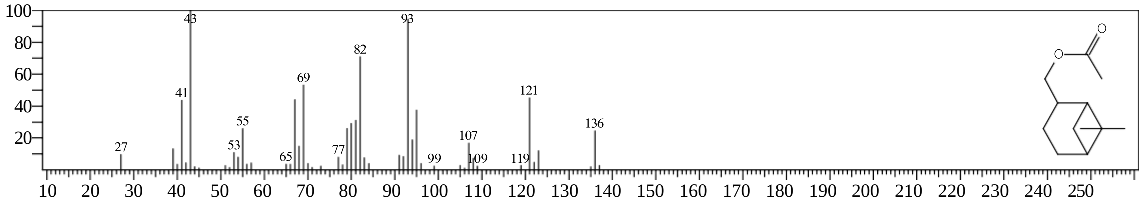

<< Target >>
Line#:60 R.Time:10.970(Scan#:2195) MassPeaks:119
RawMode:Averaged 10.965-10.975(2194-2196) BasePeak:93.20(426312)
BG Mode:Calc. from Peak

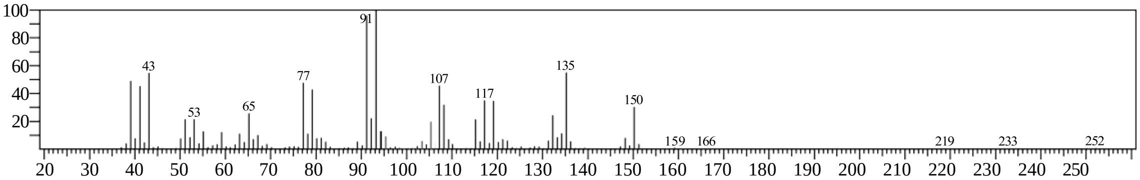

Hit#:1 Entry:13393 Library:NIST107.LIB
SI:82 Formula:C10H14O CAS:99172-18-6 MolWeight:150 RetIndex:0
CompName:3,5-Heptadienal, 2-ethylidene-6-methyl- $$

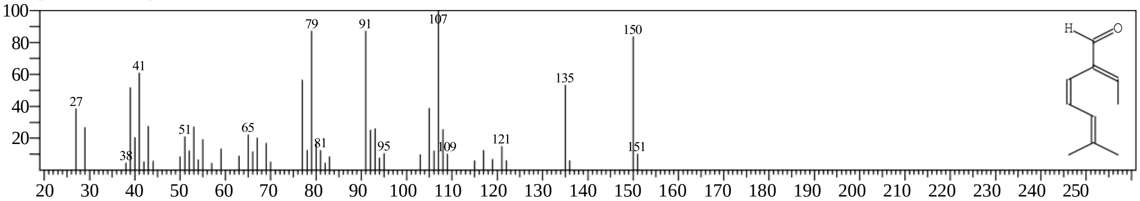

Hit#:2 Entry:13342 Library:NIST107.LIB
SI:82 Formula:C10H14O CAS:0-00-0 MolWeight:150 RetIndex:0
CompName:2-Isopropylidene-3-methylhexa-3,5-dienal $$

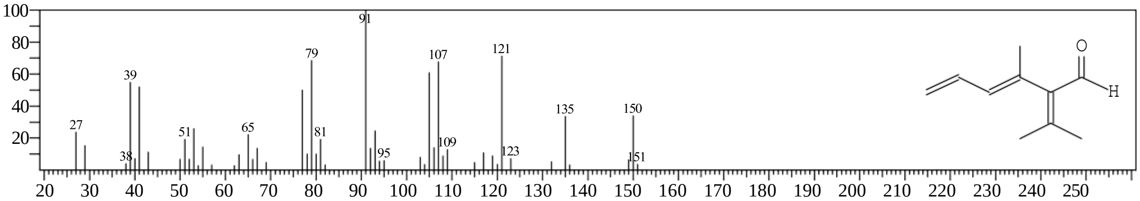

Hit#:3 Entry:24316 Library:NIST107.LIB
SI:82 Formula:C12H16O CAS:0-00-0 MolWeight:176 RetIndex:0
CompName:4,4-Diallyl-cyclohex-2-enone $$

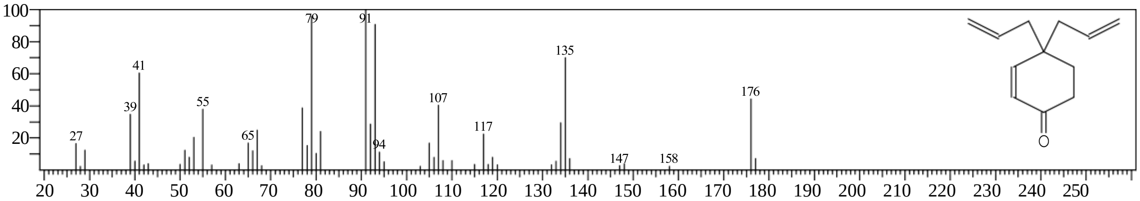

Hit#:4 Entry:13451 Library:NIST107.LIB
SI:81 Formula:C10H14O CAS:18309-32-5 MolWeight:150 RetIndex:0
CompName:D-Verbenone $$ Bicyclo[3.1.1]hept-3-en-2-one, 4,6,6-trimethyl-, (1R)- $$ 2-Pinen-4-one, (1R,5R)-(+)- $$ (+)-Verbenone $$ Bicyclo[3.1.1]hep

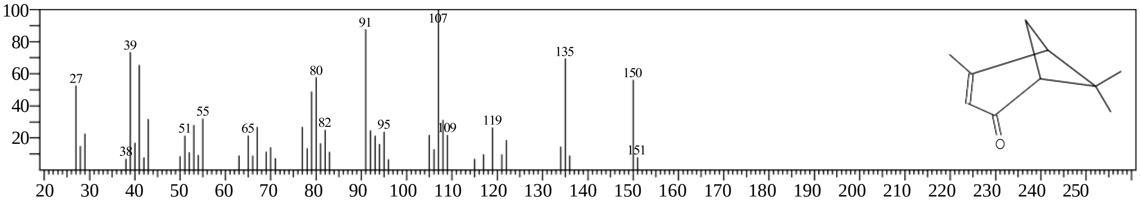

Hit#:5 Entry:13333 Library:NIST107.LIB
SI:80 Formula:C10H14O CAS:0-00-0 MolWeight:150 RetIndex:0
CompName:5-Isopropenyl-2-methylcyclopent-1-enecarboxaldehyde $$

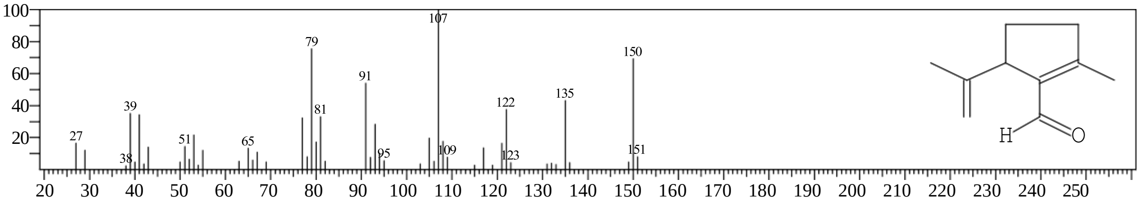

<< Target >>
Line#:61 R.Time:11.045(Scan#:2210) MassPeaks:104
RawMode:Averaged 11.040-11.050(2209-2211) BasePeak:91.20(252049)
BG Mode:Calc. from Peak

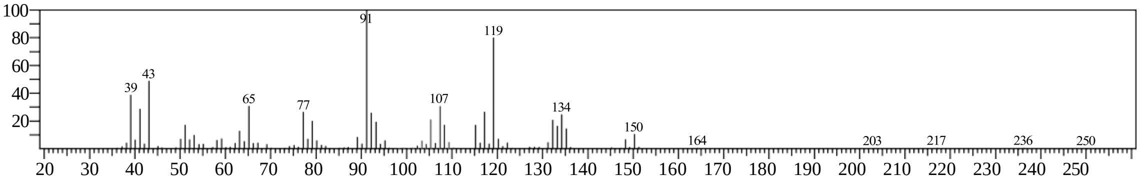

Hit#:1 Entry:14299 Library:NIST107.LIB
SI:86 Formula:C10H16O CAS:99-48-9 MolWeight:152 RetIndex:0
CompName:2-Cyclohexen-1-ol, 2-methyl-5-(1-methylethenyl)- $$ p-Mentha-1,8-dien-6-ol $$ p-Mentha-6,8-dien-2-ol $$ Carveol $$ L-p-Mentha-6,8-dien-2

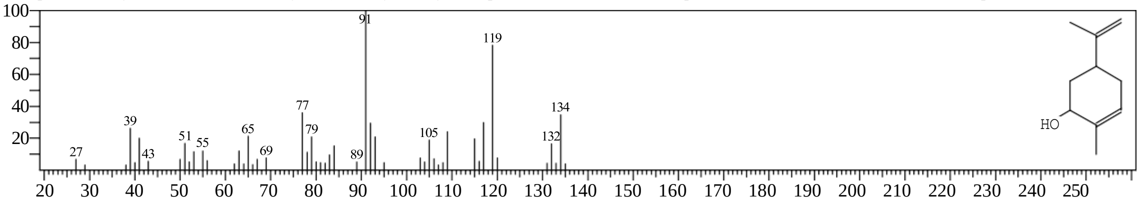

Hit#:2 Entry:14151 Library:NIST107.LIB
SI:84 Formula:C10H16O CAS:0-00-0 MolWeight:152 RetIndex:0
CompName:(E)-3-Caren-2-ol $$

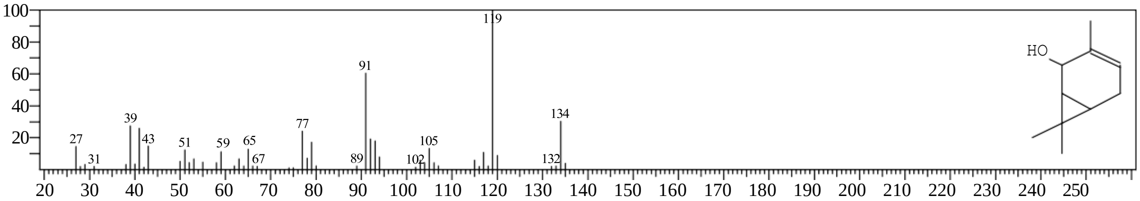

Hit#:3 Entry:8112 Library:NIST107.LIB
SI:82 Formula:C10H14 CAS:21195-59-5 MolWeight:134 RetIndex:0
CompName:1,3,8-p-Menthatriene $$ 1,3-Cyclohexadiene, 2-methyl-5-(1-methylethenyl)- $$

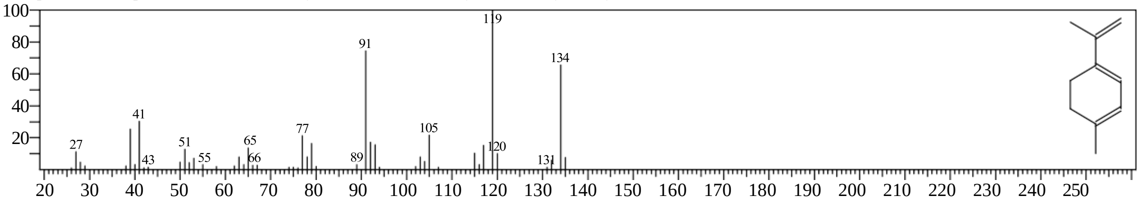

Hit#:4 Entry:8114 Library:NIST107.LIB
SI:81 Formula:C10H14 CAS:68284-24-2 MolWeight:134 RetIndex:0
CompName:Cycloheptane, 1,3,5-tris(methylene)- $$

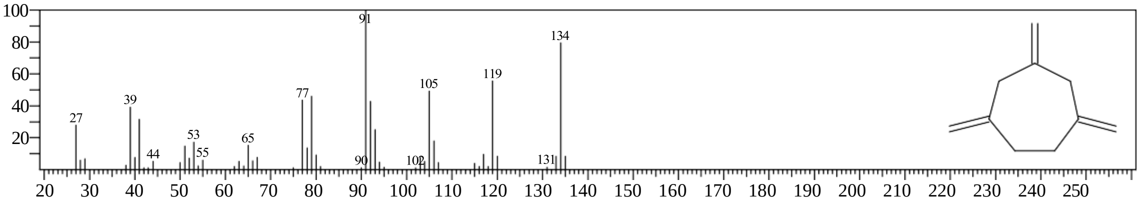

Hit#:5 Entry:8144 Library:NIST107.LIB
SI:81 Formula:C10H14 CAS:460-01-5 MolWeight:134 RetIndex:0
CompName:2,6-Dimethyl-1,3,5,7-octatetraene, E $$

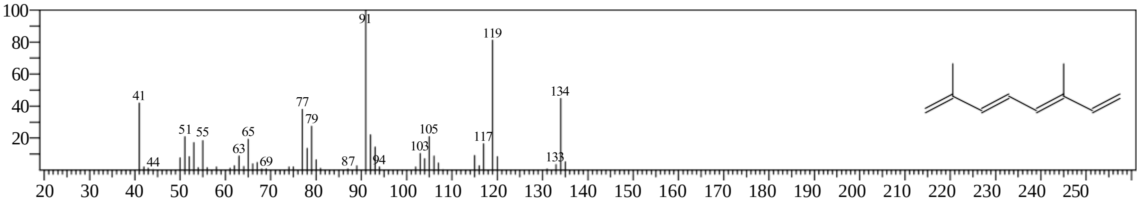

<< Target >>
Line#:62 R.Time:11.125(Scan#:2226) MassPeaks:55
RawMode:Averaged 11.120-11.130(2225-2227) BasePeak:41.10(171605)
BG Mode:Calc. from Peak

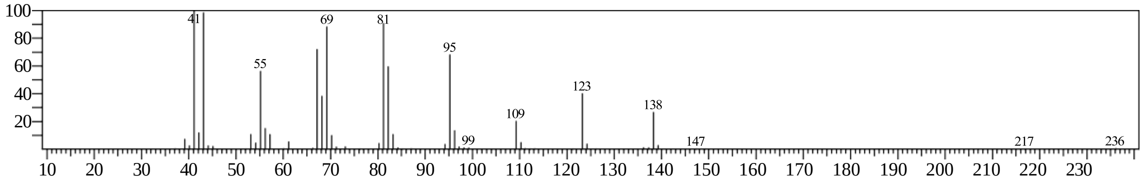

Hit#:1 Entry:9331 Library:NIST107.LIB
SI:95 Formula:C10H18 CAS:2492-22-0 MolWeight:138 RetIndex:0
CompName:cis-2,6-Dimethyl-2,6-octadiene $$

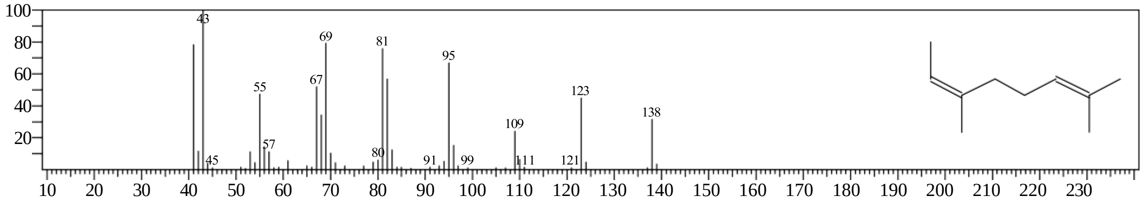

Hit#:2 Entry:45927 Library:NIST107.LIB
SI:92 Formula:C14H26O2 CAS:141-16-2 MolWeight:226 RetIndex:0
CompName:Butanoic acid, 3,7-dimethyl-6-octenyl ester $$ Butyric acid, 3,7-dimethyl-6-octenyl ester $$ Citronellyl butyrate $$ Citronellyl n-butyrate $$ Na

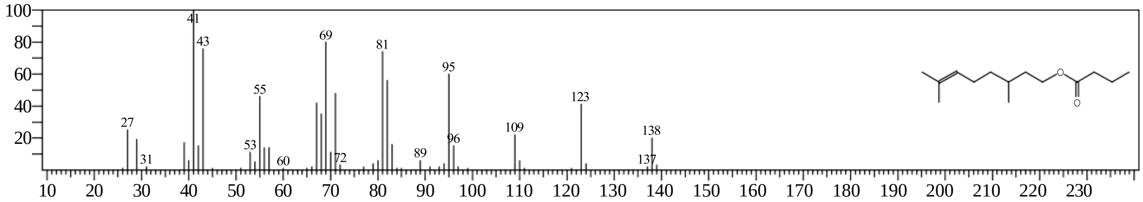

Hit#:3 Entry:33889 Library:NIST107.LIB
SI:92 Formula:C12H22O2 CAS:150-84-5 MolWeight:198 RetIndex:0
CompName:6-Octen-1-ol, 3,7-dimethyl-, acetate $$ Citronellol acetate $$ Citronellyl acetate $$ Natural rhodinol, acetylated $$ 3,7-Dimethyl-6-octenyl acet

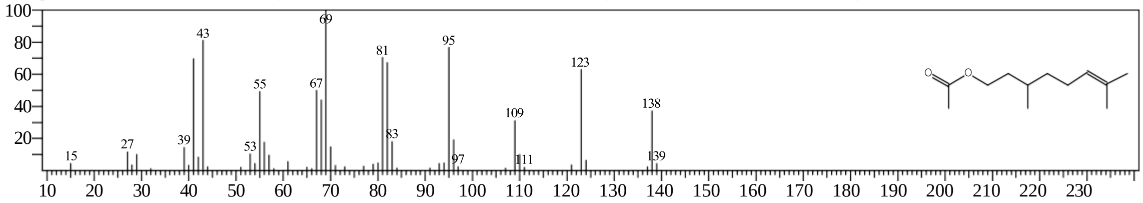

Hit#:4 Entry:39940 Library:NIST107.LIB
SI:91 Formula:C13H24O2 CAS:141-14-0 MolWeight:212 RetIndex:0
CompName:6-Octen-1-ol, 3,7-dimethyl-, propanoate $$ 6-Octen-1-ol, 3,7-dimethyl-, propionate $$ Citronellyl propionate $$

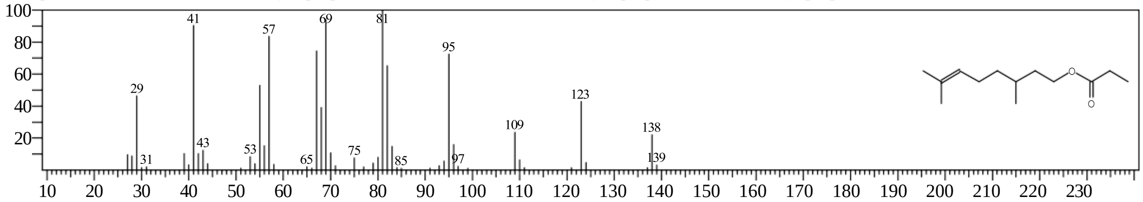

Hit#:5 Entry:9352 Library:NIST107.LIB
SI:88 Formula:C10H18 CAS:10281-56-8 MolWeight:138 RetIndex:0
CompName:R(-)3,7-Dimethyl-1,6-octadiene $$

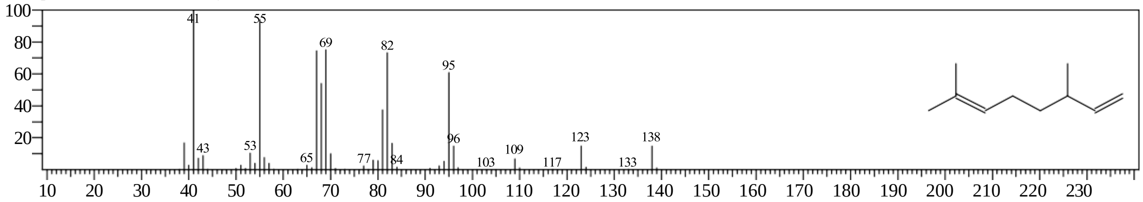

<< Target >>
Line#:63 R.Time:11.290(Scan#:2259) MassPeaks:98
RawMode:Averaged 11.285-11.295(2258-2260) BasePeak:91.15(55120)
BG Mode:Calc. from Peak

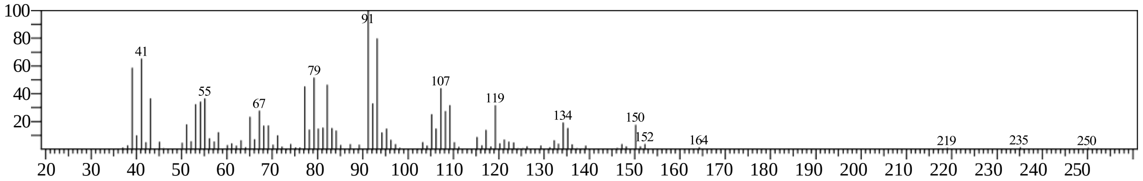

Hit#:1 Entry:13451 Library:NIST107.LIB
SI:85 Formula:C10H14O CAS:18309-32-5 MolWeight:150 RetIndex:0
CompName:D-Verbenone $$ Bicyclo[3.1.1]hept-3-en-2-one, 4,6,6-trimethyl-, (1R)- $$ 2-Pinen-4-one, (1R,5R)-(+)- $$ (+)-Verbenone $$ Bicyclo[3.1.1]hep

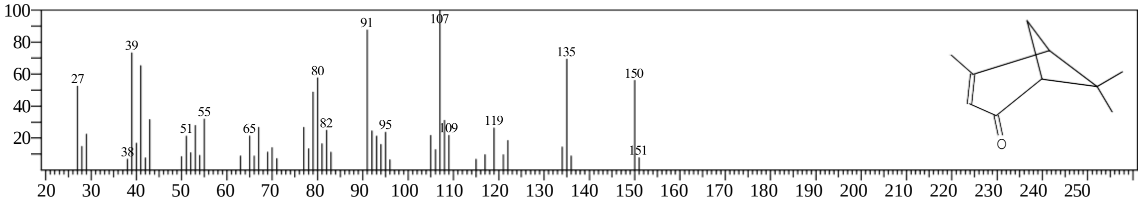

Hit#:2 Entry:30396 Library:NIST107.LIB
SI:82 Formula:C14H22 CAS:62338-42-5 MolWeight:190 RetIndex:0
CompName:Cyclobutene, 4,4-dimethyl-1-(2,7-octadienyl)- $$

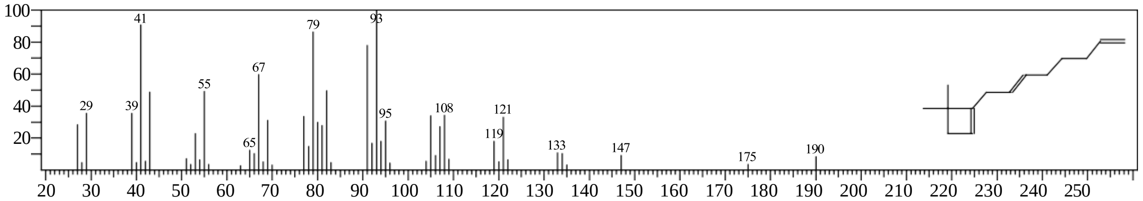

Hit#:3 Entry:30410 Library:NIST107.LIB
SI:82 Formula:C14H22 CAS:62338-27-6 MolWeight:190 RetIndex:0
CompName:1,5,9,11-Tridecatetraene, 12-methyl-, (E,E)- $$

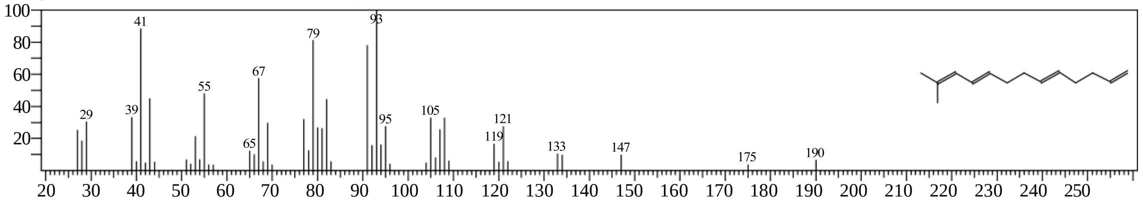

Hit#:4 Entry:25171 Library:NIST107.LIB
SI:82 Formula:C12H18O CAS:0-00-0 MolWeight:178 RetIndex:0
CompName:4-Isopropyl-trans-bicyclo[4.3.0]-2-nonen-8-one, (4R,S)- $$

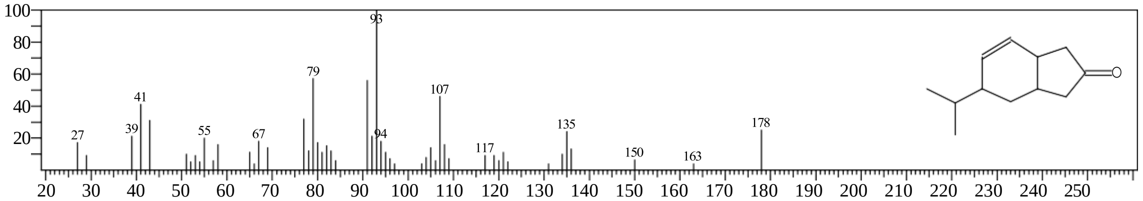

Hit#:5 Entry:25166 Library:NIST107.LIB
SI:82 Formula:C12H18O CAS:0-00-0 MolWeight:178 RetIndex:0
CompName:trans-2-Isopropylbicylclo[4.3.0]non-3-ene-8-one $$

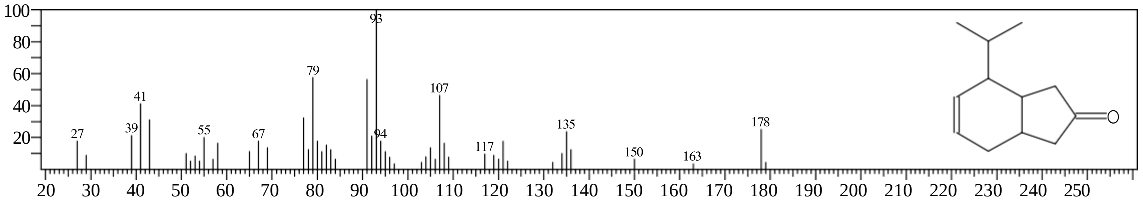

<< Target >>
Line#:64 R.Time:11.355(Scan#:2272) MassPeaks:124
RawMode:Averaged 11.350-11.360(2271-2273) BasePeak:91.20(944596)
BG Mode:Calc. from Peak

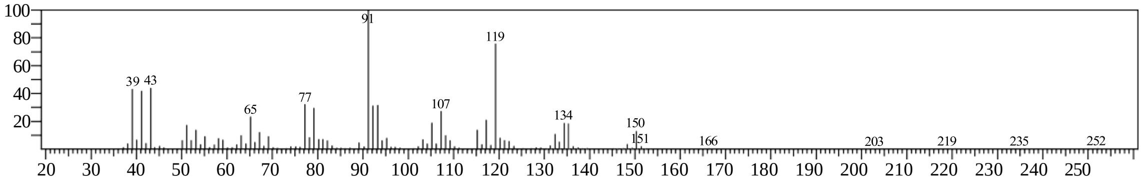

Hit#:1 Entry:14299 Library:NIST107.LIB
SI:86 Formula:C10H16O CAS:99-48-9 MolWeight:152 RetIndex:0
CompName:2-Cyclohexen-1-ol, 2-methyl-5-(1-methylethenyl)- $$ p-Mentha-1,8-dien-6-ol $$ p-Mentha-6,8-dien-2-ol $$ Carveol $$ L-p-Mentha-6,8-dien-2

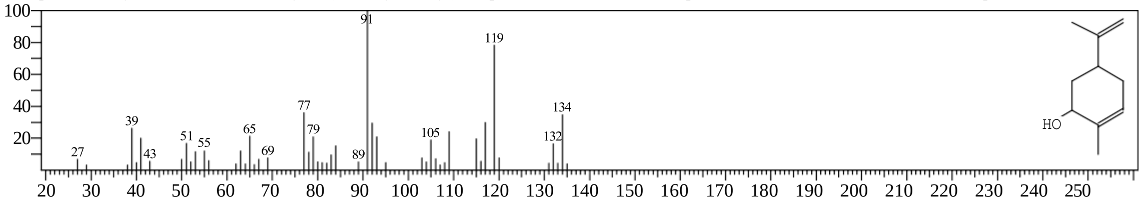

Hit#:2 Entry:18346 Library:NIST107.LIB
SI:83 Formula:C12H18 CAS:51567-08-9 MolWeight:162 RetIndex:0
CompName:Dispiro[2.1.2.4]undecane, 8-methylene- $$

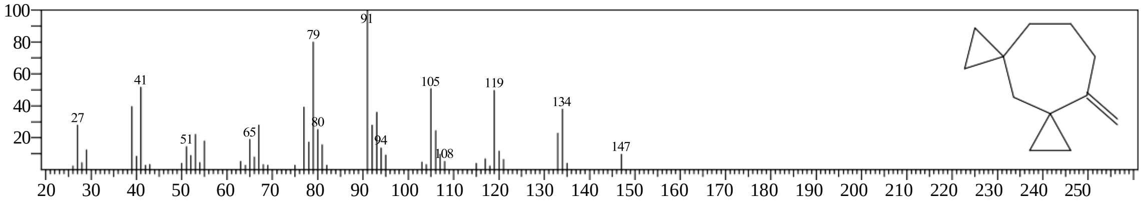

Hit#:3 Entry:31120 Library:NIST107.LIB
SI:83 Formula:C13H20O CAS:0-00-0 MolWeight:192 RetIndex:0
CompName:(+)-3-Carene, 10-(acetylmethyl)- $$

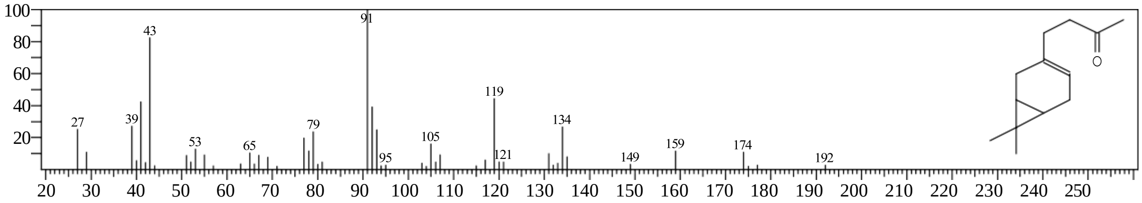

Hit#:4 Entry:18347 Library:NIST107.LIB
SI:82 Formula:C12H18 CAS:51567-09-0 MolWeight:162 RetIndex:0
CompName:Dispiro[2.0.2.5]undecane, 8-methylene- $$

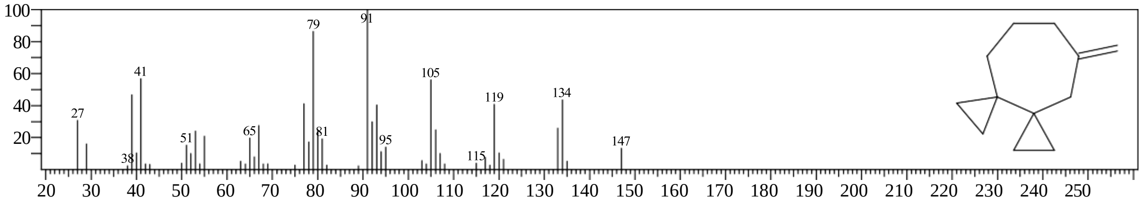

Hit#:5 Entry:31119 Library:NIST107.LIB
SI:82 Formula:C13H20O CAS:0-00-0 MolWeight:192 RetIndex:0
CompName:(+)-3-Carene, 2-(acetylmethyl)- $$

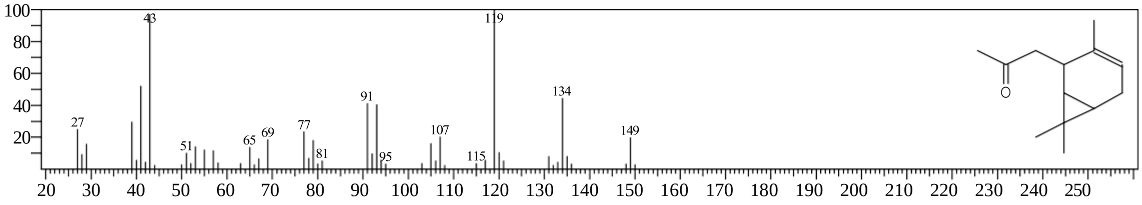

<< Target >>
Line#:65 R.Time:11.530(Scan#:2307) MassPeaks:70
RawMode:Averaged 11.525-11.535(2306-2308) BasePeak:93.20(190533)
BG Mode:Calc. from Peak

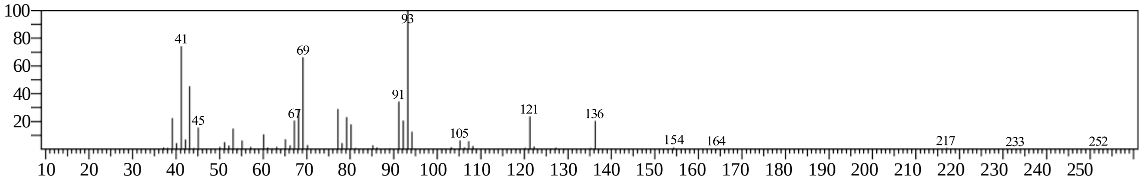

Hit#:1 Entry:45018 Library:NIST107.LIB
SI:90 Formula:C14H24O2 CAS:106-29-6 MolWeight:224 RetIndex:0
CompName:Butanoic acid, 3,7-dimethyl-2,6-octadienyl ester, (E)- $$ Butyric acid, 3,7-dimethyl-2,6-octadienyl ester, (E)- $$ Geraniol butyrate $$ Geranyl b

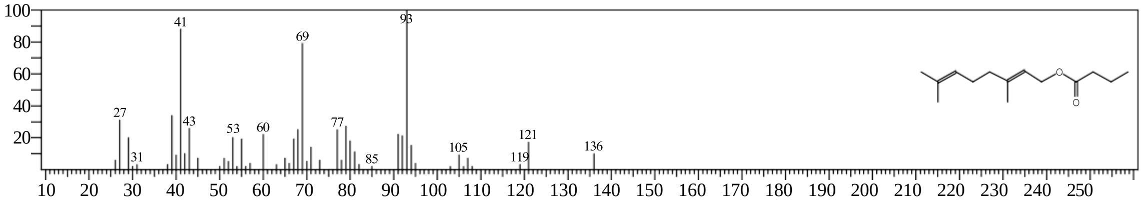

Hit#:2 Entry:8752 Library:NIST107.LIB
SI:87 Formula:C10H16 CAS:127-91-3 MolWeight:136 RetIndex:0
CompName:.beta.-Pinene $$ Bicyclo[3.1.1]heptane, 6,6-dimethyl-2-methylene- $$ 2(10)-Pinene $$ Nopinen $$ Nopinene $$ Pseudopinen $$ Pseudopinene

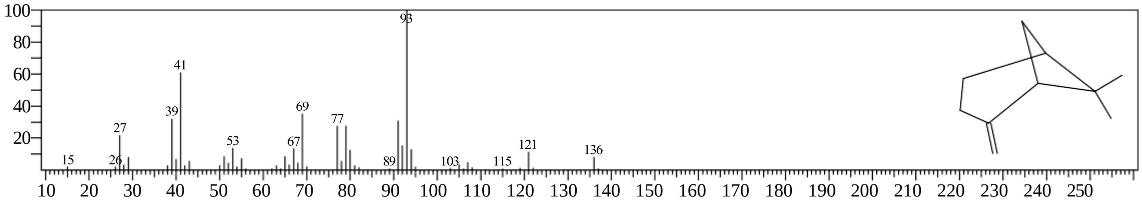

Hit#:3 Entry:33033 Library:NIST107.LIB
SI:87 Formula:C12H20O2 CAS:115-95-7 MolWeight:196 RetIndex:0
CompName:1,6-Octadien-3-ol, 3,7-dimethyl-, acetate $$ Acetic acid linalool ester $$ Bergamiol $$ Bergamol $$ Bergamot mint oil $$ Linalool acetate $$ L

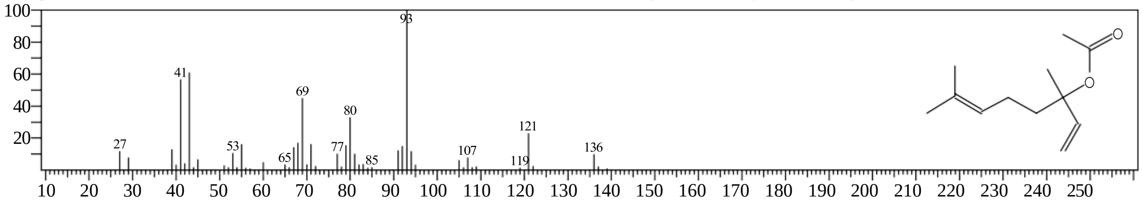

Hit#:4 Entry:8670 Library:NIST107.LIB
SI:86 Formula:C10H16 CAS:18172-67-3 MolWeight:136 RetIndex:0
CompName:Bicyclo[3.1.1]heptane, 6,6-dimethyl-2-methylene-, (1S)- $$ 2(10)-Pinene, (1S,5S)-(-)- $$ (-)-.beta.-Pinene $$ (-)-2(10)-Pinene $$ L-.beta.-pinen

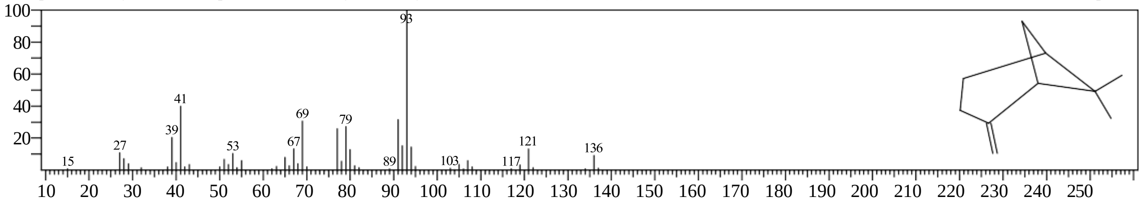

Hit#:5 Entry:8782 Library:NIST107.LIB
SI:85 Formula:C10H16 CAS:502-99-8 MolWeight:136 RetIndex:0
CompName:1,3,7-Octatriene, 3,7-dimethyl- $$ Ocimene $$ 2,6-Dimethyl-1,5,7-octatriene $$

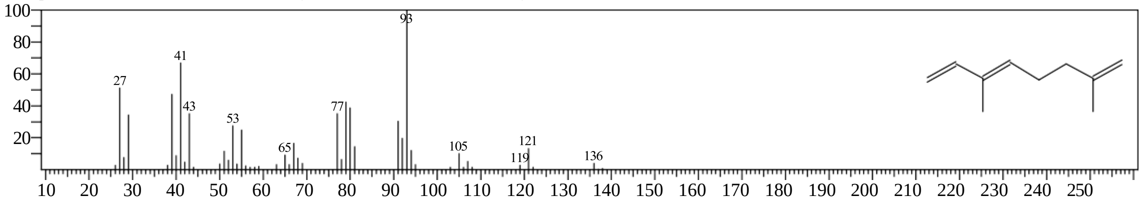

<< Target >>
Line#:66 R.Time:11.835(Scan#:2368) MassPeaks:120
RawMode:Averaged 11.830-11.840(2367-2369) BasePeak:43.10(115411)
BG Mode:Calc. from Peak

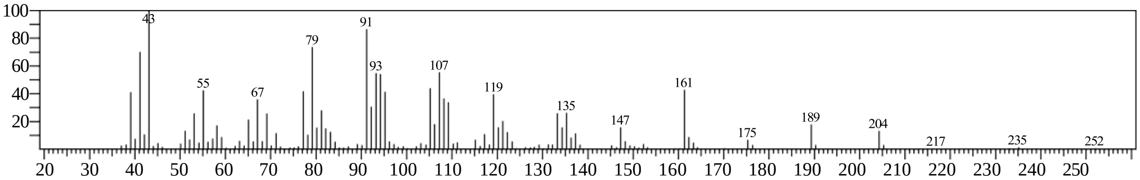

Hit#:1 Entry:36500 Library:NIST107.LIB
SI:88 Formula:C15H24 CAS:80923-88-2 MolWeight:204 RetIndex:0
CompName:1H-Benzocycloheptene, 2,4a,5,6,7,8,9,9a-octahydro-3,5,5-trimethyl-9-methylene- $$

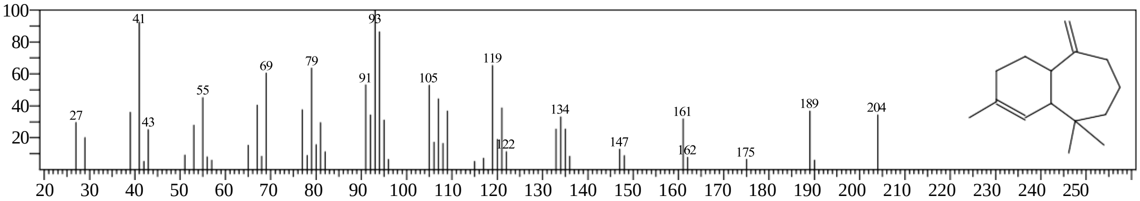

Hit#:2 Entry:36497 Library:NIST107.LIB
SI:87 Formula:C15H24 CAS:3650-28-0 MolWeight:204 RetIndex:0
CompName:1,4-Methano-1H-indene, octahydro-4-methyl-8-methylene-7-(1-methylethyl)-, [1S-(1.alpha.,3a.beta.,4.alpha.,7.alpha.,7a.beta.)]- $$ (+)-Sativen

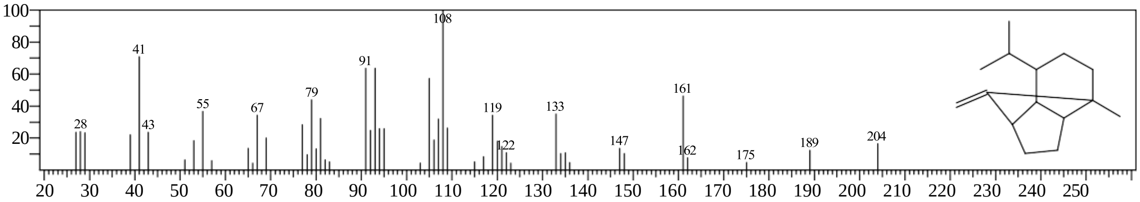

Hit#:3 Entry:36395 Library:NIST107.LIB
SI:86 Formula:C15H24 CAS:3853-83-6 MolWeight:204 RetIndex:0
CompName:1H-Benzocycloheptene, 2,4a,5,6,7,8,9,9a-octahydro-3,5,5-trimethyl-9-methylene-, (4aS-cis)- $$ 1H-Benzocycloheptene, 2,4a.alpha.,5,6,7,8,9,9

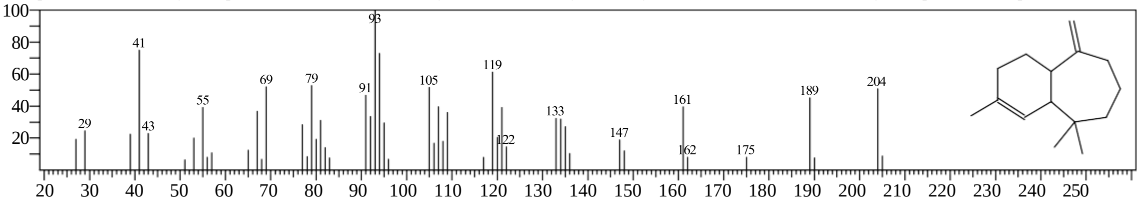

Hit#:4 Entry:36505 Library:NIST107.LIB
SI:85 Formula:C15H24 CAS:72747-25-2 MolWeight:204 RetIndex:0
CompName:1H-Cycloprop[e]azulene, decahydro-1,1,7-trimethyl-4-methylene-, (1a.a $$

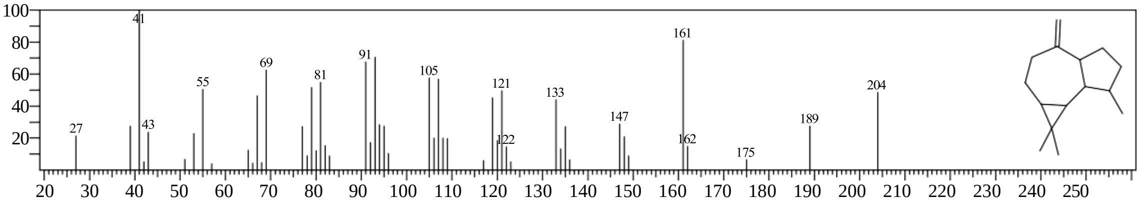

Hit#:5 Entry:36318 Library:NIST107.LIB
SI:85 Formula:C15H24 CAS:0-00-0 MolWeight:204 RetIndex:0
CompName:1R,3Z,9s-4,11,11-Trimethyl-8-methylenebicyclo[7.2.0]undec-3-ene $$

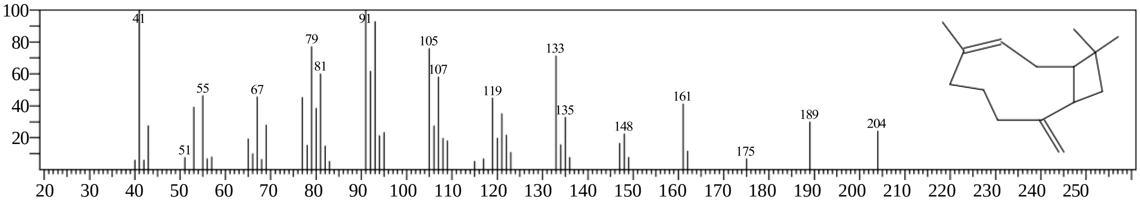

<< Target >>
Line#:67 R.Time:12.330(Scan#:2467) MassPeaks:69
RawMode:Averaged 12.325-12.335(2466-2468) BasePeak:119.25(151122)
BG Mode:Calc. from Peak

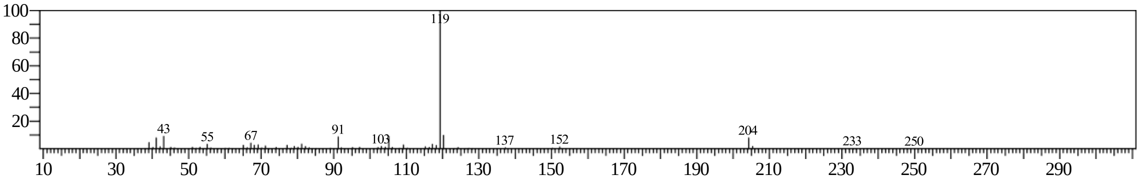

Hit#:1 Entry:36352 Library:NIST107.LIB
SI:86 Formula:C15H24 CAS:0-00-0 MolWeight:204 RetIndex:0
CompName:Dihydrocurcumene $$

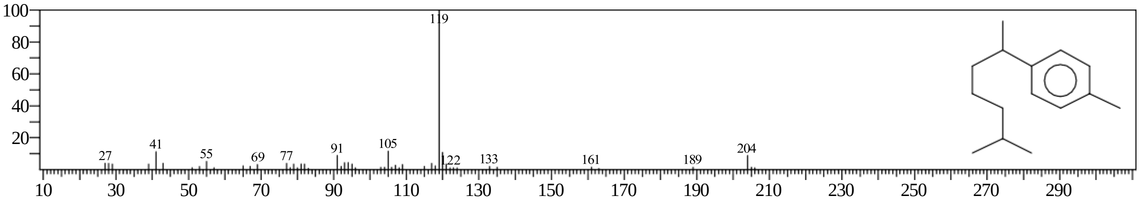

Hit#:2 Entry:36405 Library:NIST107.LIB
SI:86 Formula:C15H24 CAS:1461-02-5 MolWeight:204 RetIndex:0
CompName:Benzene, 1-(1,5-dimethylhexyl)-4-methyl- $$

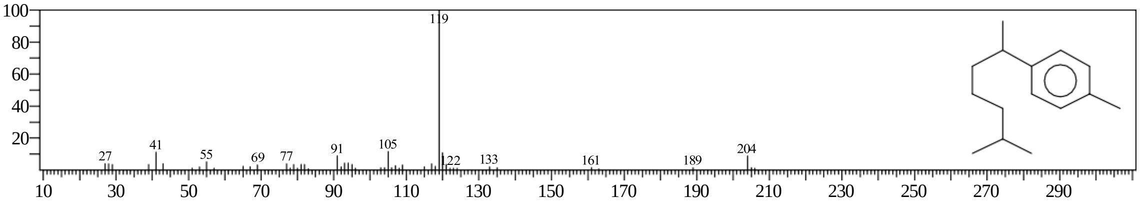

Hit#:3 Entry:72441 Library:NIST107.LIB
SI:86 Formula:C22H38 CAS:29138-94-1 MolWeight:302 RetIndex:0
CompName:Pentadecane, 2-methyl-2-phenyl- $$

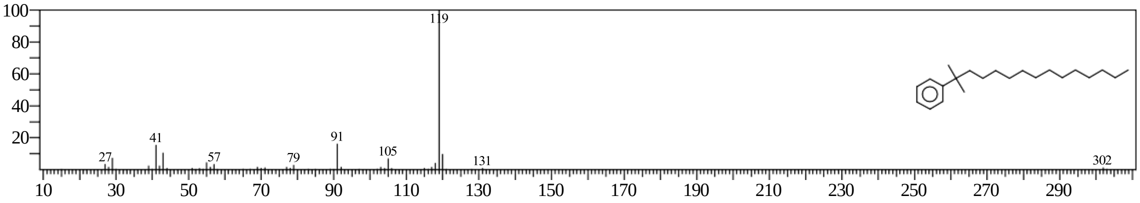

Hit#:4 Entry:63534 Library:NIST107.LIB
SI:86 Formula:C20H34 CAS:27854-41-7 MolWeight:274 RetIndex:0
CompName:Tridecane, 2-methyl-2-phenyl- $$

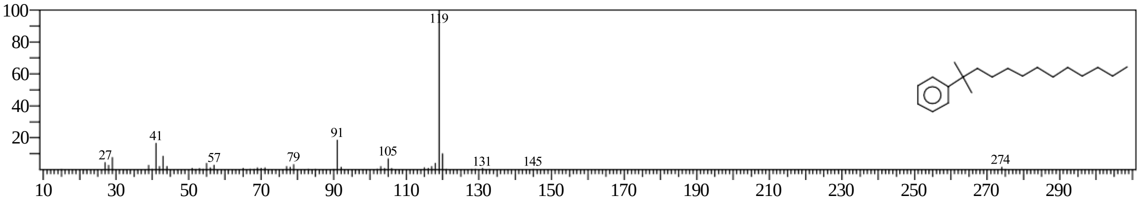

Hit#:5 Entry:53413 Library:NIST107.LIB
SI:85 Formula:C18H30 CAS:27854-40-6 MolWeight:246 RetIndex:0
CompName:Benzene, (1,1-dimethyldecyl)- $$ Undecane, 2-methyl-2-phenyl- $$

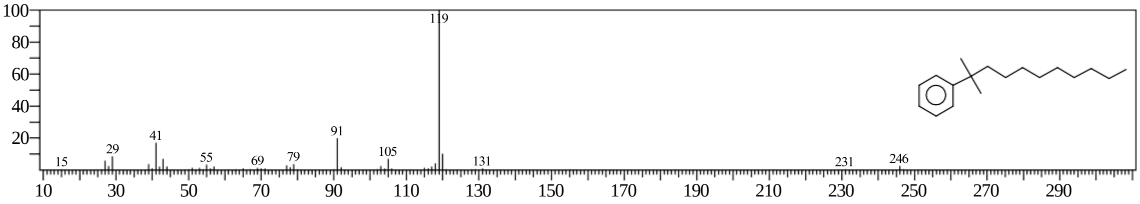

<< Target >>
Line#:68 R.Time:12.385(Scan#:2478) MassPeaks:81
RawMode:Averaged 12.380-12.390(2477-2479) BasePeak:91.15(24914)
BG Mode:Calc. from Peak

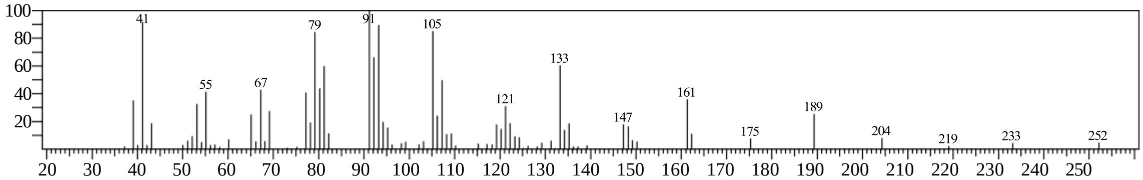

Hit#:1 Entry:36318 Library:NIST107.LIB
SI:92 Formula:C15H24 CAS:0-00-0 MolWeight:204 RetIndex:0
CompName:1R,3Z,9s-4,11,11-Trimethyl-8-methylenebicyclo[7.2.0]undec-3-ene $$

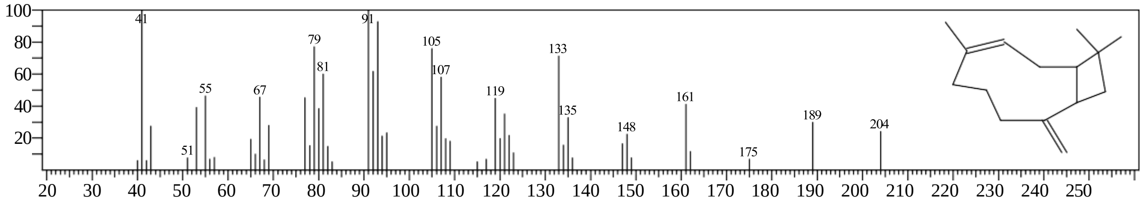

Hit#:2 Entry:36367 Library:NIST107.LIB
SI:91 Formula:C15H24 CAS:0-00-0 MolWeight:204 RetIndex:0
CompName:Humulen-(v1) $$

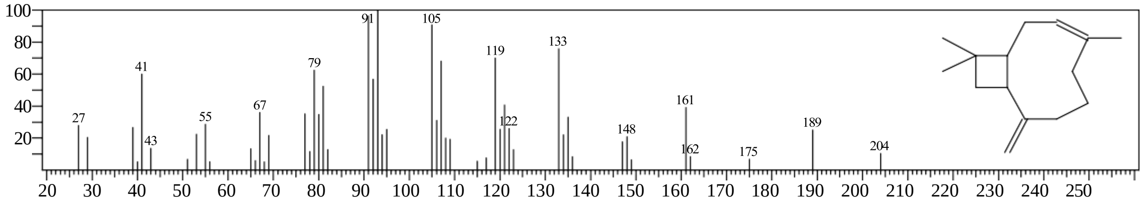

Hit#:3 Entry:36366 Library:NIST107.LIB
SI:88 Formula:C15H24 CAS:0-00-0 MolWeight:204 RetIndex:0
CompName:Bicyclo[5.3.0]decane, 2-methylene-5-(1-methylvinyl)-8-methyl- $$

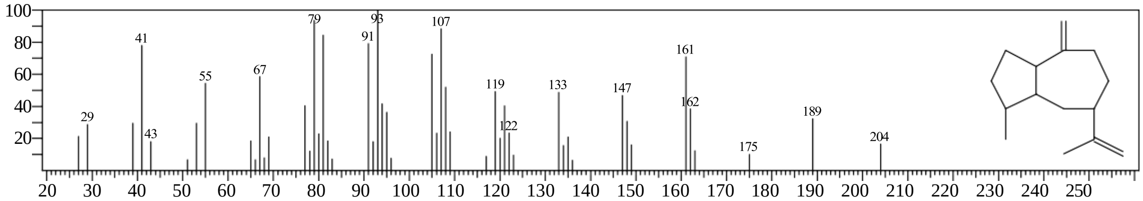

Hit#:4 Entry:36479 Library:NIST107.LIB
SI:88 Formula:C15H24 CAS:13877-93-5 MolWeight:204 RetIndex:0
CompName:Bicyclo[7.2.0]undec-4-ene, 4,11,11-trimethyl-8-methylene- $$ Bicyclo[7.2.0]undec-4-ene, 4,11,11-trimethyl-8-methylene-, (Z)- $$ cis-4,11,11-

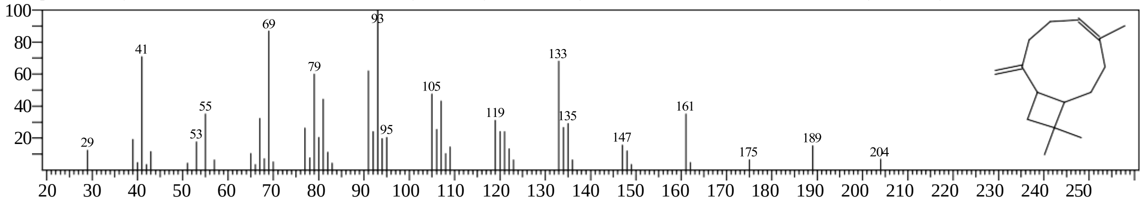

Hit#:5 Entry:36481 Library:NIST107.LIB
SI:88 Formula:C15H24 CAS:489-39-4 MolWeight:204 RetIndex:0
CompName:1H-Cycloprop[e]azulene, decahydro-1,1,7-trimethyl-4-methylene-, [1aR-(1a.alpha.,4a.alpha.,7.alpha.,7a.beta.,7b.alpha.)]- $$ 1H-Cycloprop[e]a

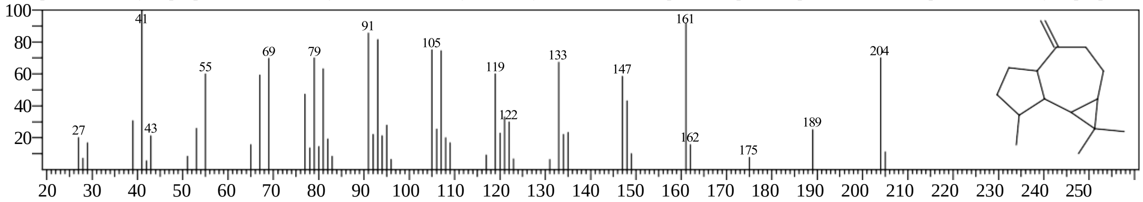

<< Target >>
Line#:69 R.Time:12.445(Scan#:2490) MassPeaks:85
RawMode:Averaged 12.440-12.450(2489-2491) BasePeak:93.20(195904)
BG Mode:Calc. from Peak

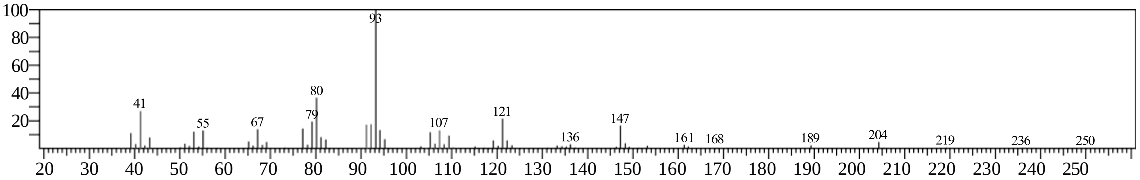

Hit#:1 Entry:36488 Library:NIST107.LIB
SI:95 Formula:C15H24 CAS:6753-98-6 MolWeight:204 RetIndex:0
CompName:.alpha.-Caryophyllene $$ 1,4,8-Cycloundecatriene, 2,6,6,9-tetramethyl-, (E,E,E)- $$ .alpha.-Humulene $$ Humulene $$ Cycloundeca-1,4,8-trie

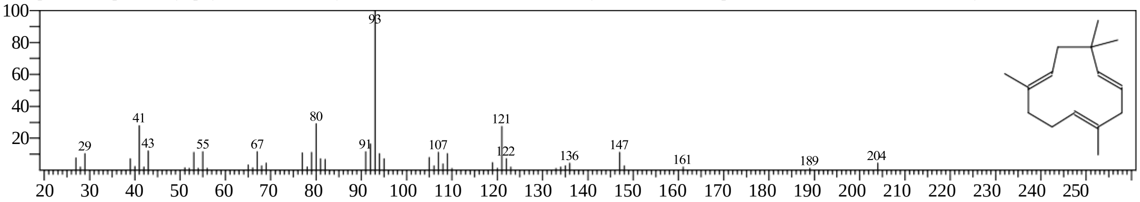

Hit#:2 Entry:36310 Library:NIST107.LIB
SI:92 Formula:C15H24 CAS:0-00-0 MolWeight:204 RetIndex:0
CompName:4,7,10-Cycloundecatriene, 1,1,4,8-tetramethyl-, cis, cis, cis- $$

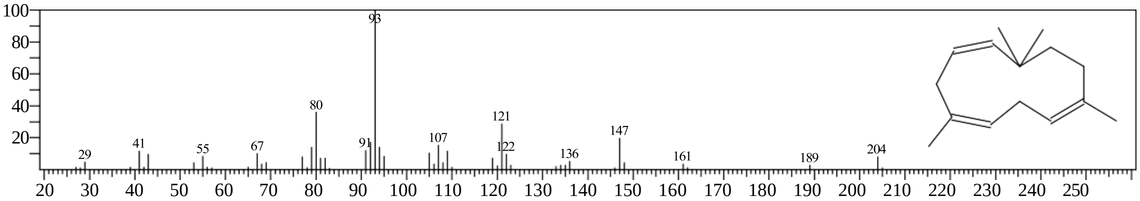

Hit#:3 Entry:36415 Library:NIST107.LIB
SI:88 Formula:C15H24 CAS:17627-44-0 MolWeight:204 RetIndex:0
CompName:Cyclohexene, 4-(1,5-dimethyl-1,4-hexadienyl)-1-methyl- $$ 2,5-Heptadiene, 2-methyl-6-(4-methyl-3-cyclohexen-1-yl)- $$ .alpha.-Bisabolene $

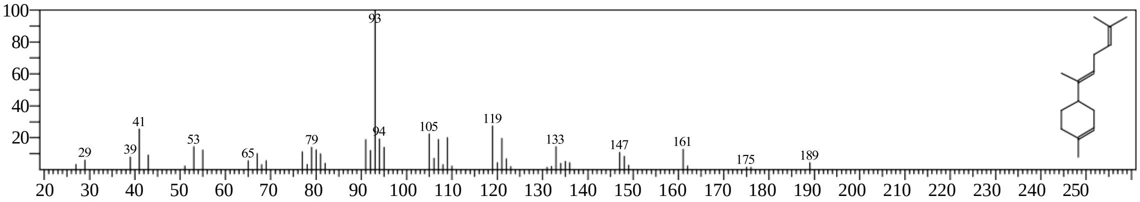

Hit#:4 Entry:8678 Library:NIST107.LIB
SI:86 Formula:C10H16 CAS:488-97-1 MolWeight:136 RetIndex:0
CompName:Tricyclo[2.2.1.02,6]heptane, 1,3,3-trimethyl- $$ Cyclofenchene $$ Cyclofeuchene $$

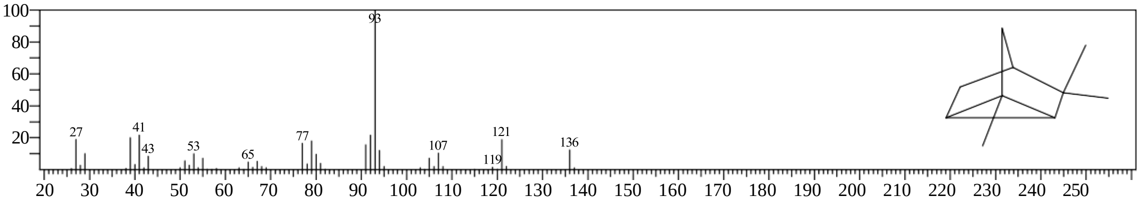

Hit#:5 Entry:36354 Library:NIST107.LIB
SI:85 Formula:C15H24 CAS:0-00-0 MolWeight:204 RetIndex:0
CompName:Cycloundeca-2,6,9-triene, 1,1,5,9-tetramethyl- $$

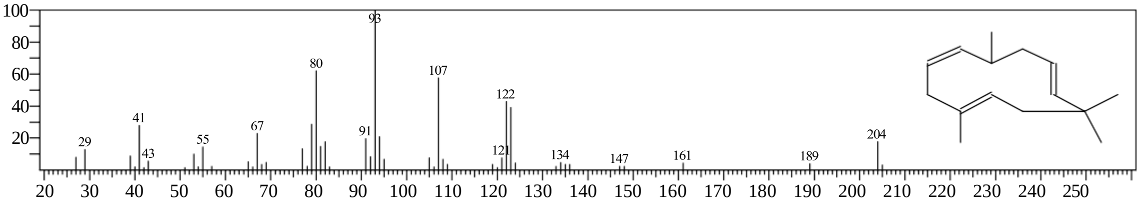

<< Target >>
Line#:70 R.Time:12.730(Scan#:2547) MassPeaks:82
RawMode:Averaged 12.725-12.735(2546-2548) BasePeak:161.30(57138)
BG Mode:Calc. from Peak

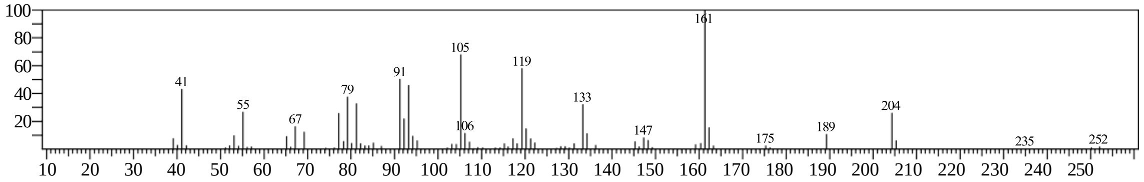

Hit#:1 Entry:36341 Library:NIST107.LIB
SI:88 Formula:C15H24 CAS:0-00-0 MolWeight:204 RetIndex:0
CompName:Isoledene $$

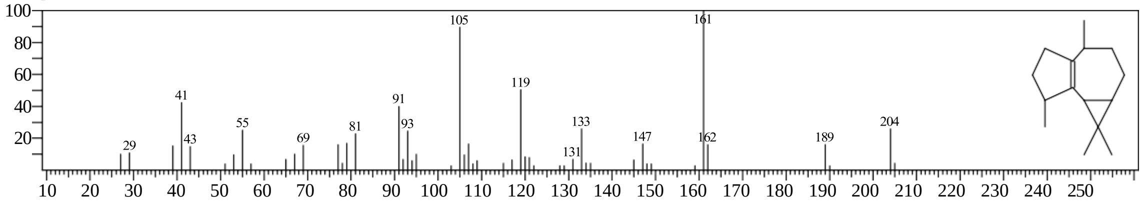

Hit#:2 Entry:36446 Library:NIST107.LIB
SI:88 Formula:C15H24 CAS:30021-74-0 MolWeight:204 RetIndex:0
CompName:Naphthalene, 1,2,3,4,4a,5,6,8a-octahydro-7-methyl-4-methylene-1-(1-methylethyl)-, (1.alpha.,4a.alpha.,8a.alpha.)- $$ .gamma.-Muurolene $$

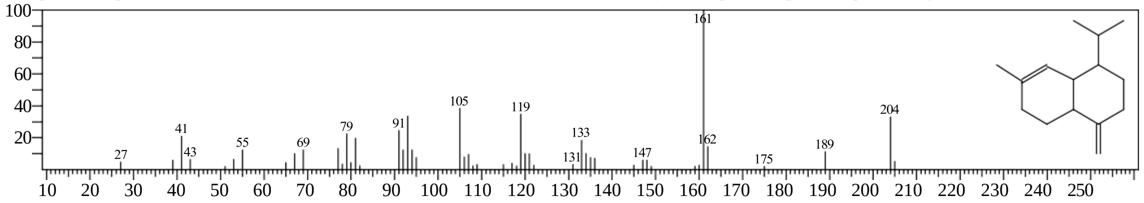

Hit#:3 Entry:36457 Library:NIST107.LIB
SI:87 Formula:C15H24 CAS:3856-25-5 MolWeight:204 RetIndex:0
CompName:Copaene $$ Tricyclo[4.4.0.02,7]dec-3-ene, 1,3-dimethyl-8-(1-methylethyl)-, stereoisomer $$ Tricyclo[4.4.0.02,7]dec-3-ene, 8-isopropyl-1,3-di

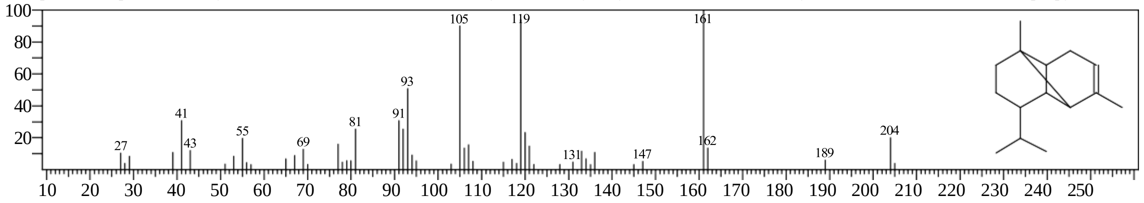

Hit#:4 Entry:36422 Library:NIST107.LIB
SI:87 Formula:C15H24 CAS:23986-74-5 MolWeight:204 RetIndex:0
CompName:Germacrene D $$ 1,6-Cyclodecadiene, 1-methyl-5-methylene-8-(1-methylethyl)-, [s-(E,E)]- $$

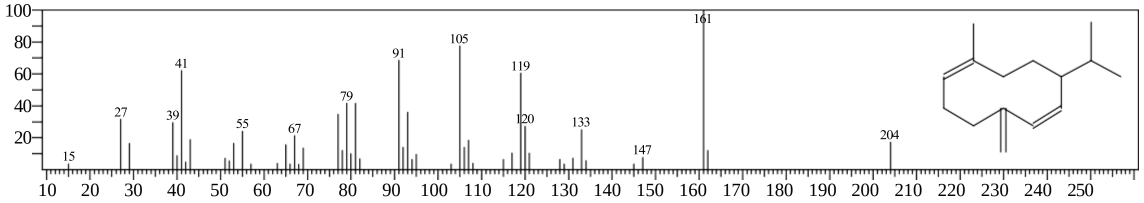

Hit#:5 Entry:36432 Library:NIST107.LIB
SI:87 Formula:C15H24 CAS:17699-14-8 MolWeight:204 RetIndex:0
CompName:.alpha.-Cubebene $$ 1H-Cyclopenta[1,3]cyclopropa[1,2]benzene, 3a,3b,4,5,6,7-hexahydro-3,7-dimethyl-4-(1-methylethyl)-, [3aS-(3a.alpha.,3b.

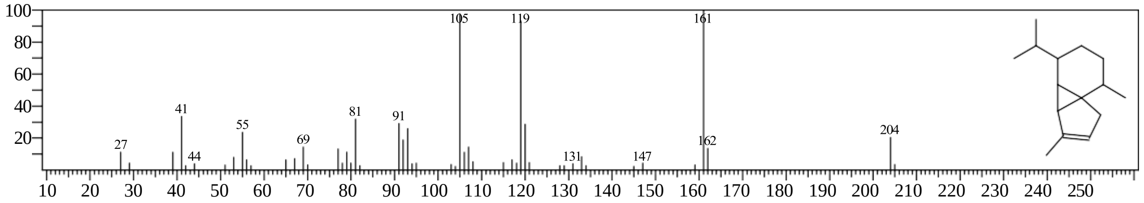

<< Target >>
Line#:71 R.Time:12.780(Scan#:2557) MassPeaks:106
RawMode:Averaged 12.775-12.785(2556-2558) BasePeak:135.25(72667)
BG Mode:Calc. from Peak

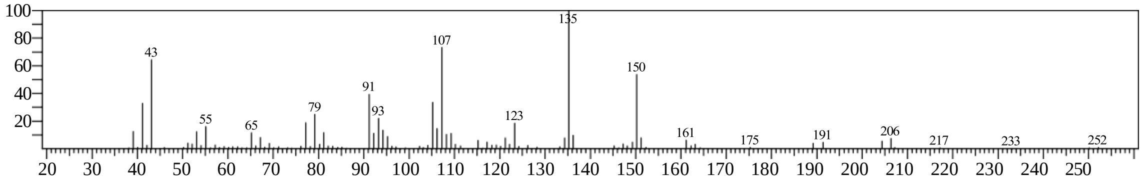

Hit#:1 Entry:37264 Library:NIST107.LIB
SI:88 Formula:C14H22O CAS:127-51-5 MolWeight:206 RetIndex:0
CompName:.alpha. Isomethyl ionone $$ 4-(2,6,6-Trimethyl 2-cyclohexen-1-yl)-3-methyl-3-buten-2-one $$ 3-Buten-2-one, 3-methyl-4-(2,6,6-trimethyl-2-cyc

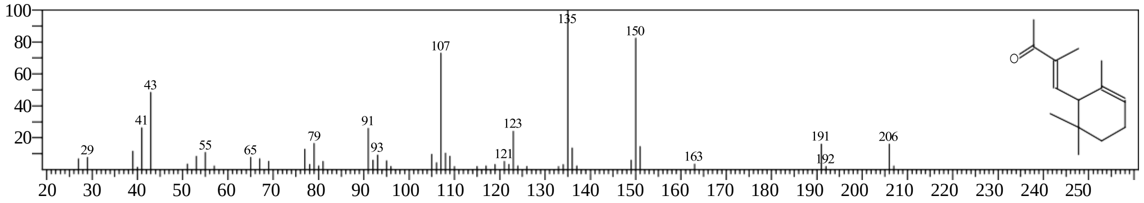

Hit#:2 Entry:37261 Library:NIST107.LIB
SI:85 Formula:C14H22O CAS:79-89-0 MolWeight:206 RetIndex:0
CompName:3-Buten-2-one, 3-methyl-4-(2,6,6-trimethyl-1-cyclohexen-1-yl)- $$ .delta.-Iraldeine $$ Isomethyl-.beta.-ionone $$ 4-(2,6,6-Trimethyl-1-cyclohe

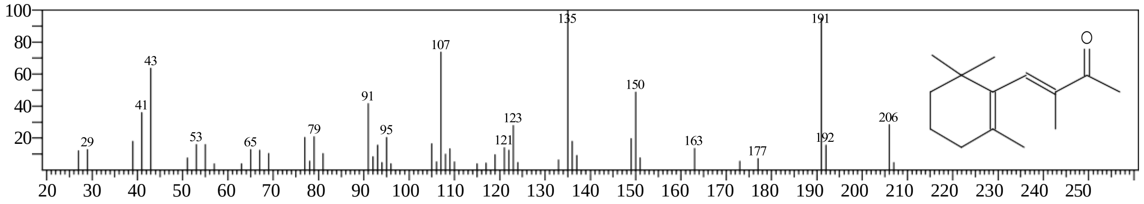

Hit#:3 Entry:13427 Library:NIST107.LIB
SI:83 Formula:C10H14O CAS:24555-40-6 MolWeight:150 RetIndex:0
CompName:Ethanone, 1-(6,6-dimethylbicyclo[3.1.0]hex-2-en-2-yl)- $$

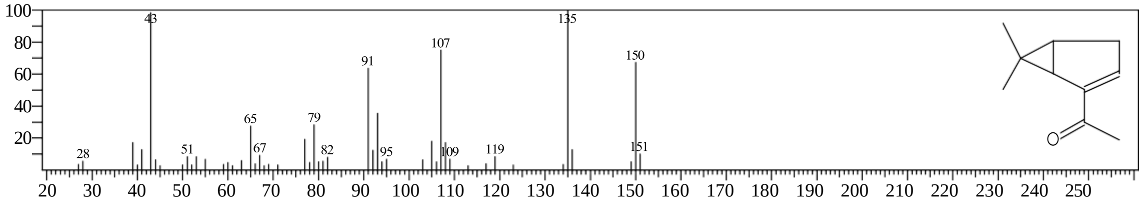

Hit#:4 Entry:48823 Library:NIST107.LIB
SI:82 Formula:C14H22N2O CAS:0-00-0 MolWeight:234 RetIndex:0
CompName:1-[3-(2,6,6-Trimethyl-cyclohex-2-enyl)-4,5-dihydro-3H-pyrazol-4-yl]-ethanone $$

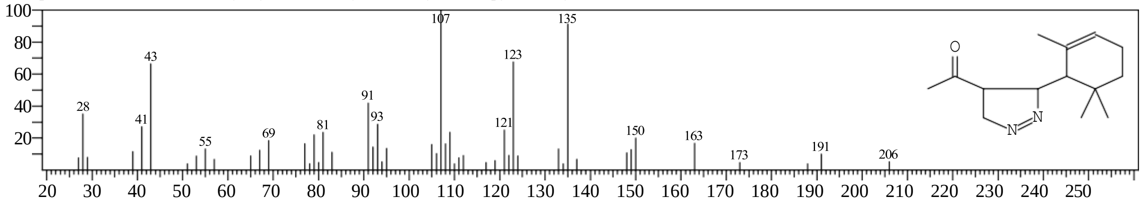

Hit#:5 Entry:37266 Library:NIST107.LIB
SI:82 Formula:C14H22O CAS:114933-28-7 MolWeight:206 RetIndex:0
CompName:3-Penten-2-one, 4-(2,6,6-trimethyl-2-cyclohexen-1-yl)- $$

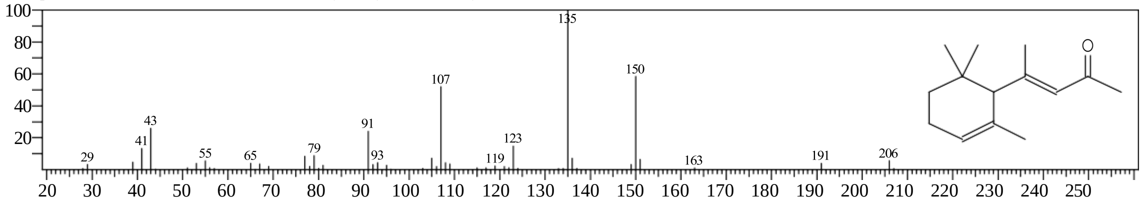

<< Target >>
Line#:72 R.Time:12.875(Scan#:2576) MassPeaks:99
RawMode:Averaged 12.870-12.880(2575-2577) BasePeak:41.10(58949)
BG Mode:Calc. from Peak

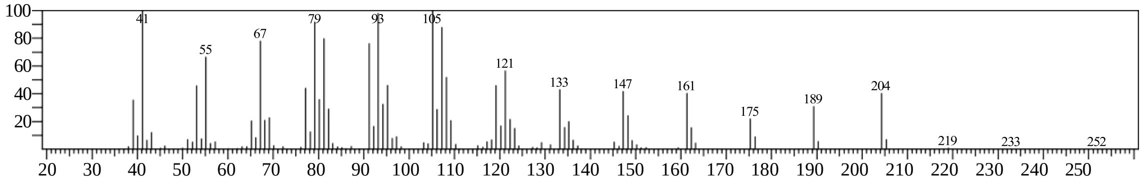

Hit#:1 Entry:36335 Library:NIST107.LIB
SI:95 Formula:C15H24 CAS:0-00-0 MolWeight:204 RetIndex:0
CompName:Eudesma-4(14),11-diene $$

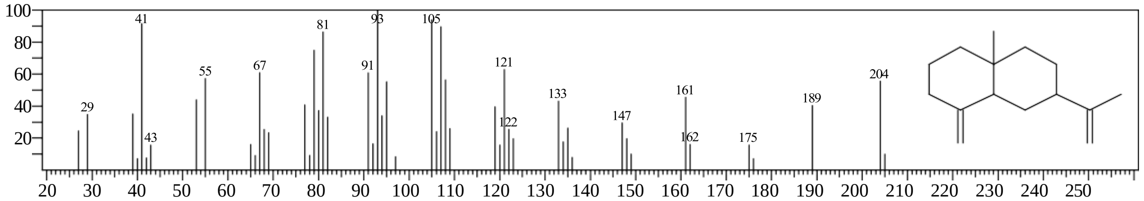

Hit#:2 Entry:36477 Library:NIST107.LIB
SI:94 Formula:C15H24 CAS:17066-67-0 MolWeight:204 RetIndex:0
CompName:Naphthalene, decahydro-4a-methyl-1-methylene-7-(1-methylethenyl)-, [4aR-(4a.alpha.,7.alpha.,8a.beta.)]- $$ Eudesma-4(14),11-diene $$ .beta.

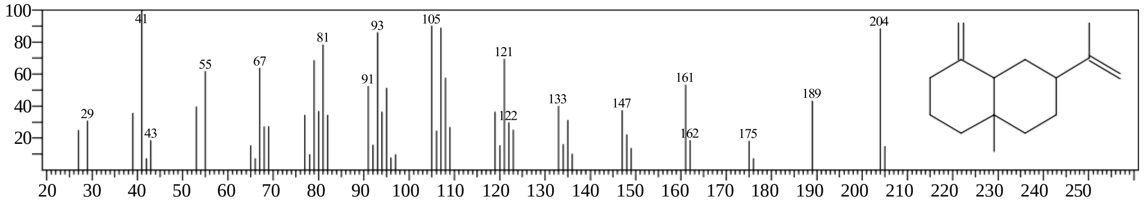

Hit#:3 Entry:36366 Library:NIST107.LIB
SI:92 Formula:C15H24 CAS:0-00-0 MolWeight:204 RetIndex:0
CompName:Bicyclo[5.3.0]decane, 2-methylene-5-(1-methylvinyl)-8-methyl- $$

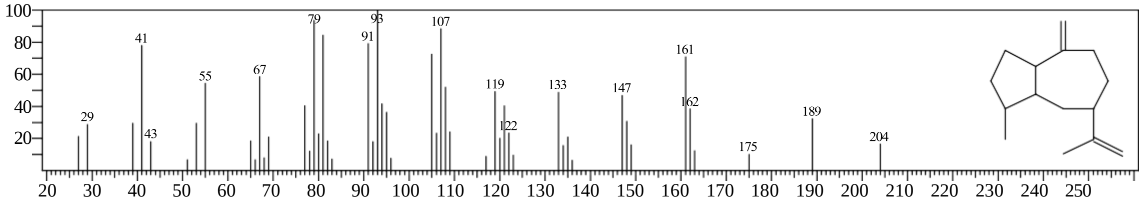

Hit#:4 Entry:36501 Library:NIST107.LIB
SI:92 Formula:C15H24 CAS:10219-75-7 MolWeight:204 RetIndex:0
CompName:Naphthalene, 1,2,3,5,6,7,8,8a-octahydro-1,8a-dimethyl-7-(1-methylethenyl)-, [1S-(1.alpha.,7.alpha.,8a.alpha.)]- $$ Eremophila-1(10),11-diene $

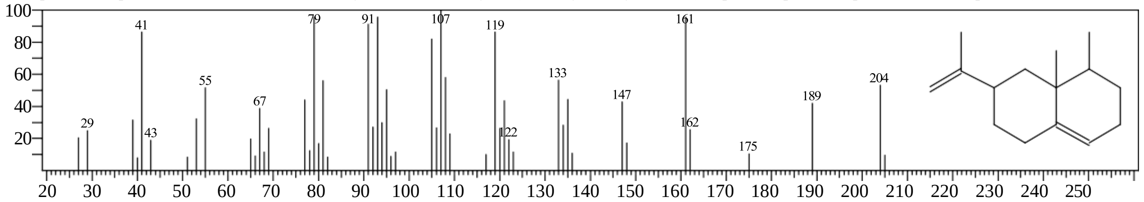

Hit#:5 Entry:36361 Library:NIST107.LIB
SI:90 Formula:C15H24 CAS:0-00-0 MolWeight:204 RetIndex:0
CompName:Cycloheptane, 4-methylene-1-methyl-2-(2-methyl-1-propen-1-yl)-1-vinyl- $$

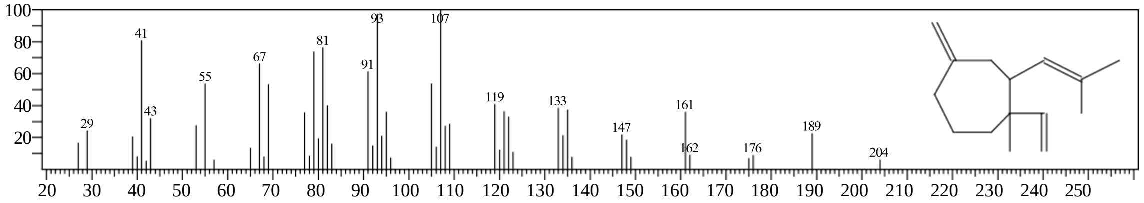

<< Target >>
Line#:73 R.Time:13.000(Scan#:2601) MassPeaks:107
RawMode:Averaged 12.995-13.005(2600-2602) BasePeak:93.20(55015)
BG Mode:Calc. from Peak

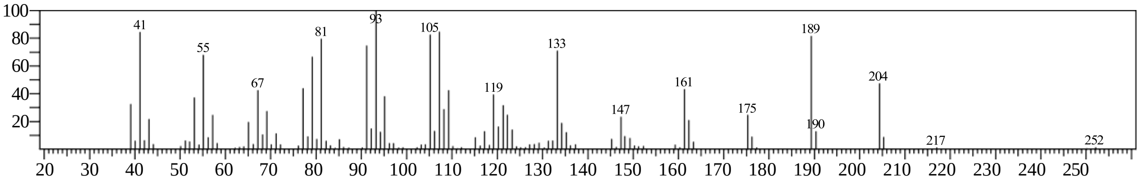

Hit#:1 Entry:36410 Library:NIST107.LIB
SI:90 Formula:C15H24 CAS:473-13-2 MolWeight:204 RetIndex:0
CompName:Naphthalene, 1,2,3,4,4a,5,6,8a-octahydro-4a,8-dimethyl-2-(1-methylethenyl)-, [2R-(2.alpha.,4a.alpha.,8a.beta.)]- $$

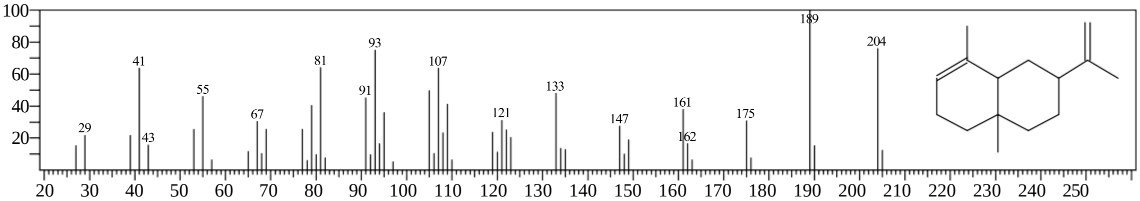

Hit#:2 Entry:36435 Library:NIST107.LIB
SI:90 Formula:C15H24 CAS:18431-82-8 MolWeight:204 RetIndex:0
CompName:Spiro[5.5]undec-2-ene, 3,7,7-trimethyl-11-methylene-, (-)- $$ .beta.-Chamigrene $$ Chamigren $$ Chamigrene $$ Spiro[5.5]undec-2-ene, 3,7,7

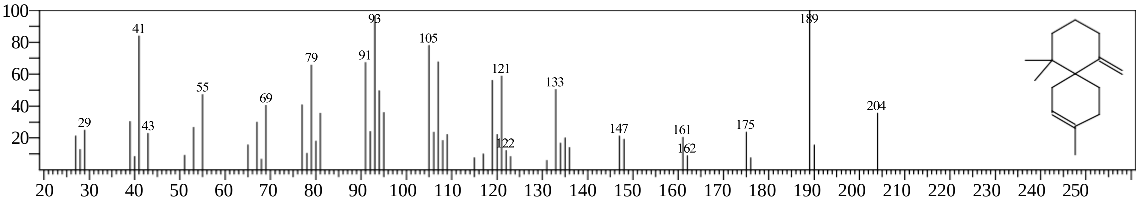

Hit#:3 Entry:36335 Library:NIST107.LIB
SI:89 Formula:C15H24 CAS:0-00-0 MolWeight:204 RetIndex:0
CompName:Eudesma-4(14),11-diene $$

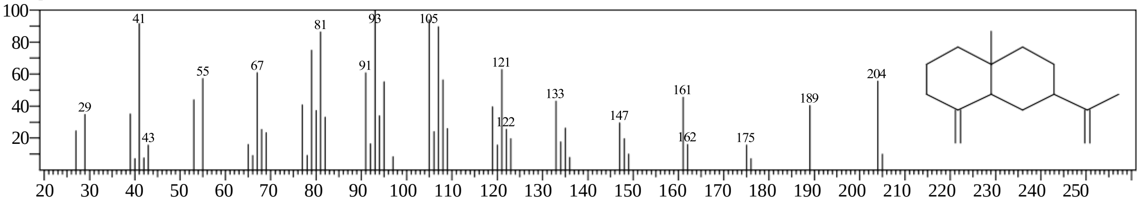

Hit#:4 Entry:36392 Library:NIST107.LIB
SI:89 Formula:C15H24 CAS:22567-17-5 MolWeight:204 RetIndex:0
CompName:Azulene, 1,2,3,3a,4,5,6,7-octahydro-1,4-dimethyl-7-(1-methylethenyl)-, [1R-(1.alpha.,3a.beta.,4.alpha.,7.beta.)]- $$ 1.beta.,4.beta.H,10.beta.H-

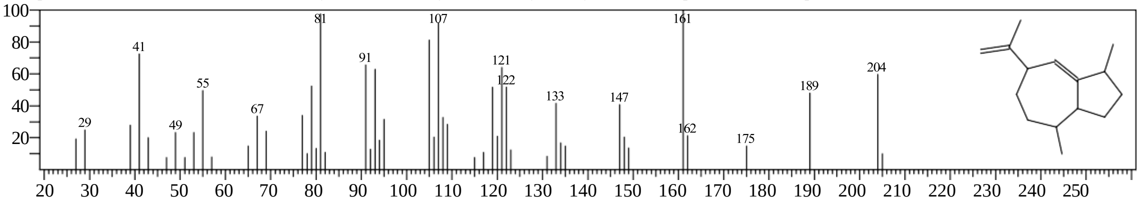

Hit#:5 Entry:36343 Library:NIST107.LIB
SI:88 Formula:C15H24 CAS:0-00-0 MolWeight:204 RetIndex:0
CompName:Longifolene-(V4) $$

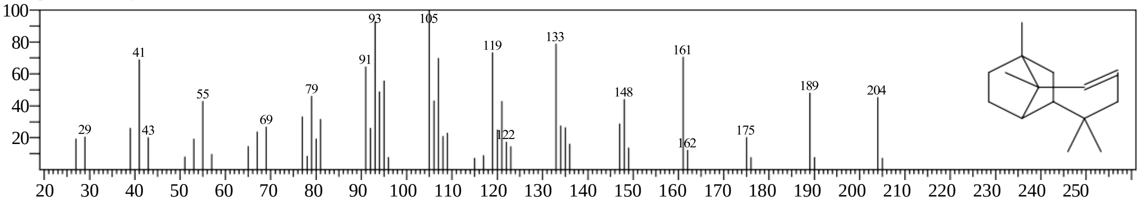

<< Target >>
Line#:74 R.Time:13.060(Scan#:2613) MassPeaks:99
RawMode:Averaged 13.055-13.065(2612-2614) BasePeak:105.20(154185)
BG Mode:Calc. from Peak

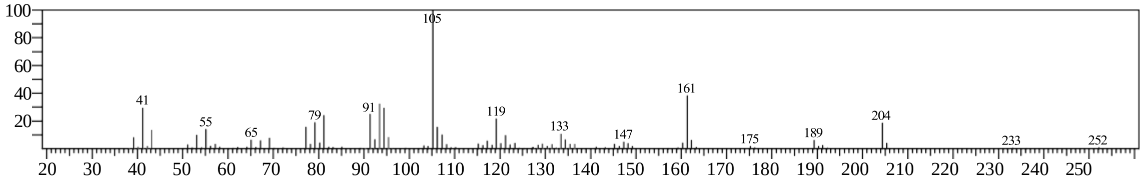

Hit#:1 Entry:36473 Library:NIST107.LIB
SI:96 Formula:C15H24 CAS:483-75-0 MolWeight:204 RetIndex:0
CompName:Naphthalene, 1,2,4a,5,6,8a-hexahydro-4,7-dimethyl-1-(1-methylethyl)- $$

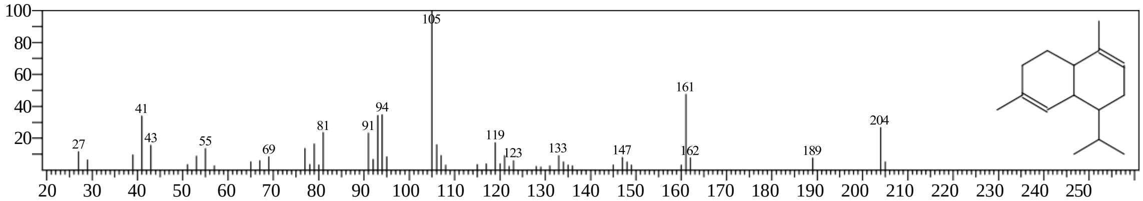

Hit#:2 Entry:36407 Library:NIST107.LIB
SI:95 Formula:C15H24 CAS:10208-80-7 MolWeight:204 RetIndex:0
CompName:.alpha.-Muurolene $$

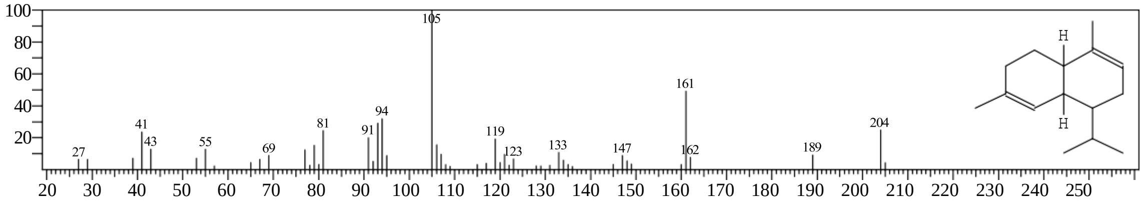

Hit#:3 Entry:36456 Library:NIST107.LIB
SI:92 Formula:C15H24 CAS:31983-22-9 MolWeight:204 RetIndex:0
CompName:Naphthalene, 1,2,4a,5,6,8a-hexahydro-4,7-dimethyl-1-(1-methylethyl)-, (1.alpha.,4a.alpha.,8a.alpha.)- $$ 1.Xi.,6.xi.,7.xi.-Cadina-4,9-diene $$ .a

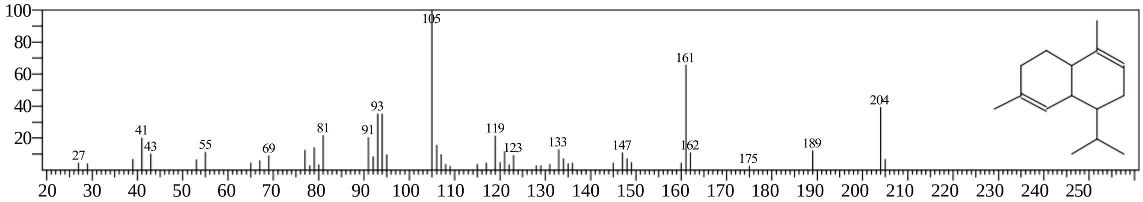

Hit#:4 Entry:36406 Library:NIST107.LIB
SI:91 Formula:C15H24 CAS:24406-05-1 MolWeight:204 RetIndex:0
CompName:Naphthalene, 1,2,4a,5,6,8a-hexahydro-4,7-dimethyl-1-(1-methylethyl)-, [1s-(1.alpha.,4a.beta.,8a.alpha.)]- $$

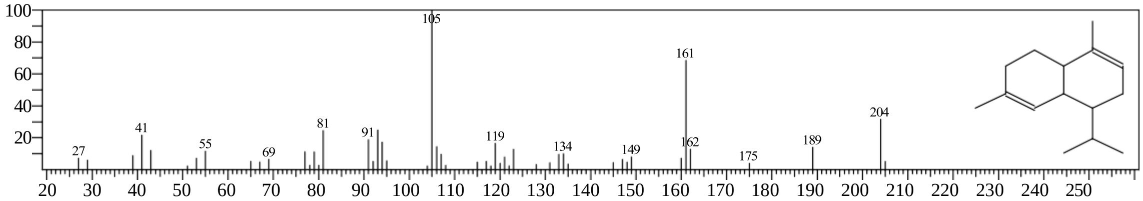

Hit#:5 Entry:36448 Library:NIST107.LIB
SI:90 Formula:C15H24 CAS:17627-24-6 MolWeight:204 RetIndex:0
CompName:Naphthalene, 1,2,4a,5,6,8a-hexahydro-4,7-dimethyl-1-(1-methylethyl)-,[1R-(1.alpha.,4a.alpha.,8a.alpha.)]- $$

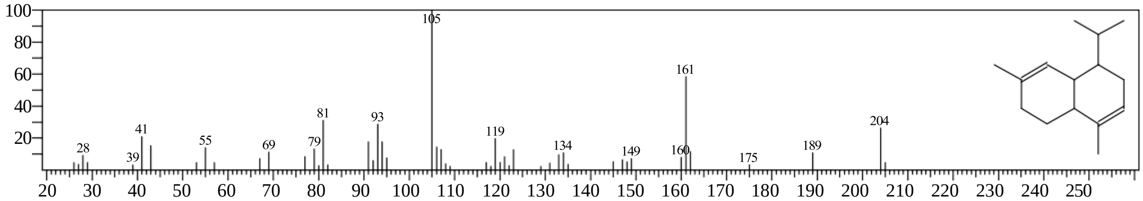

<< Target >>
Line#:75 R.Time:13.135(Scan#:2628) MassPeaks:111
RawMode:Averaged 13.130-13.140(2627-2629) BasePeak:43.10(82678)
BG Mode:Calc. from Peak

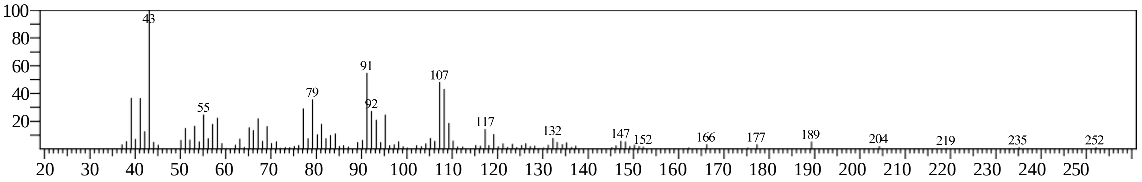

Hit#:1 Entry:19992 Library:NIST107.LIB
SI:79 Formula:C11H18O CAS:0-00-0 MolWeight:166 RetIndex:0
CompName:5-Isopropenyl-1,2-dimethylcyclohex-2-enol $$

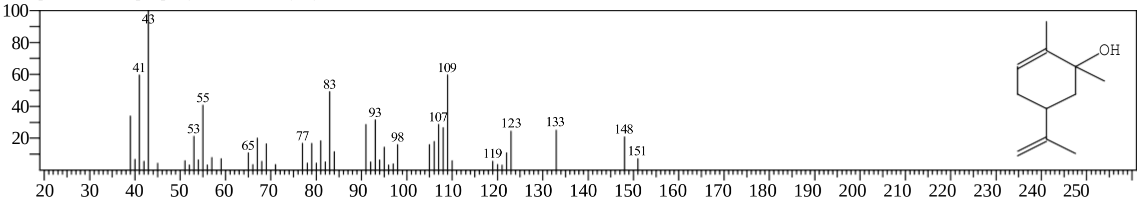

Hit#:2 Entry:14152 Library:NIST107.LIB
SI:78 Formula:C10H16O CAS:0-00-0 MolWeight:152 RetIndex:0
CompName:Thujol $$

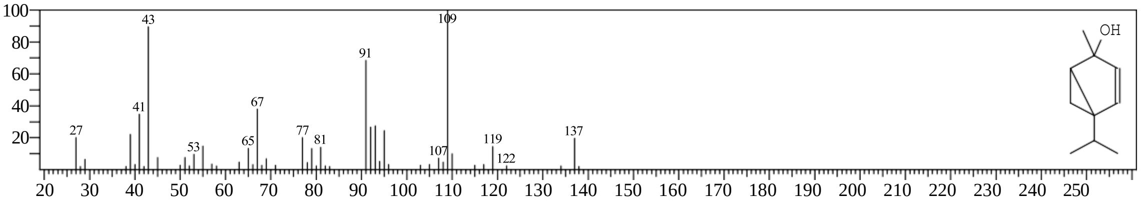

Hit#:3 Entry:20826 Library:NIST107.LIB
SI:78 Formula:C10H16O2 CAS:96-08-2 MolWeight:168 RetIndex:0
CompName:7-Oxabicyclo[4.1.0]heptane, 1-methyl-4-(2-methyloxiranyl)- $$ p-Menthane, 1,2:8,9-diepoxy- $$ .alpha.-Limonene diepoxide $$ Dipentene die

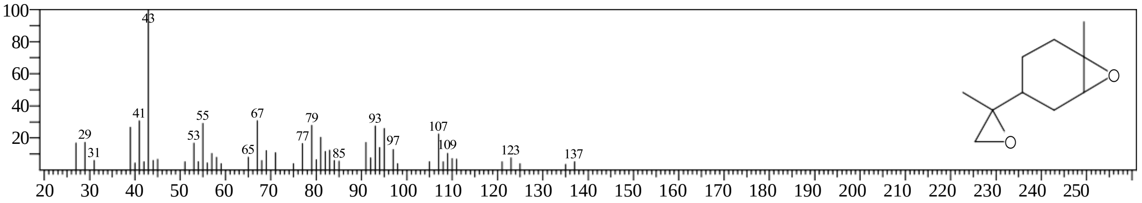

Hit#:4 Entry:14316 Library:NIST107.LIB
SI:78 Formula:C10H16O CAS:515-00-4 MolWeight:152 RetIndex:0
CompName:Bicyclo[3.1.1]hept-2-ene-2-methanol, 6,6-dimethyl- $$ 2-Pinen-10-ol $$ Myrtenol $$

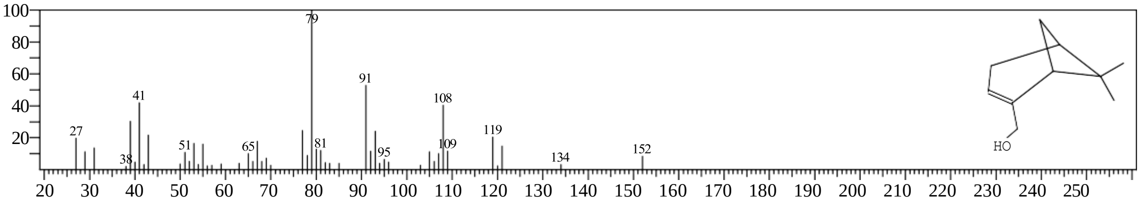

Hit#:5 Entry:8698 Library:NIST107.LIB
SI:77 Formula:C10H16 CAS:61827-88-1 MolWeight:136 RetIndex:0
CompName:3-Decen-1-yne, (Z)- $$

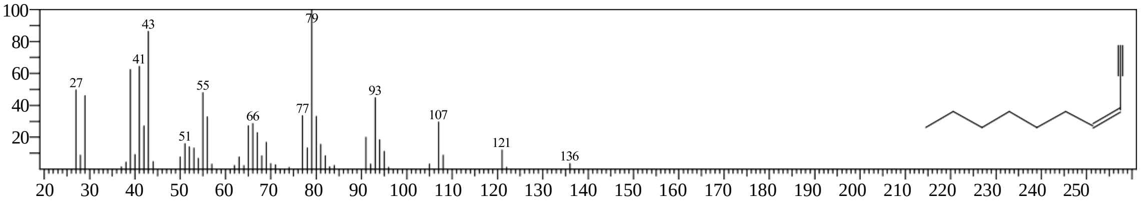

<< Target >>
Line#:76 R.Time:13.335(Scan#:2668) MassPeaks:123
RawMode:Averaged 13.330-13.340(2667-2669) BasePeak:66.15(1417629)
BG Mode:Calc. from Peak

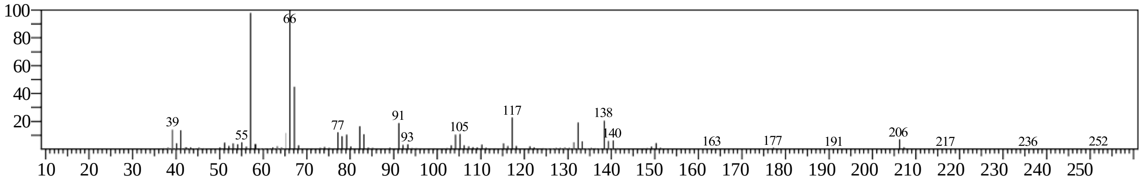

Hit#:1 Entry:37041 Library:NIST107.LIB
SI:85 Formula:C12H14O3 CAS:0-00-0 MolWeight:206 RetIndex:0
CompName:Indan-1,3-diol monopropionate $$

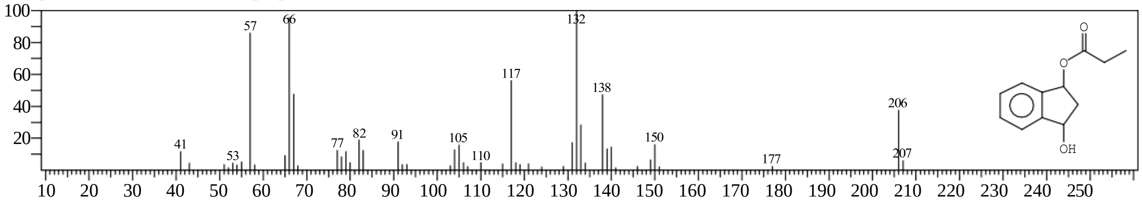

Hit#:2 Entry:13425 Library:NIST107.LIB
SI:79 Formula:C10H14O CAS:70220-93-8 MolWeight:150 RetIndex:0
CompName:Tricyclo[4.2.1.1(2,5)]dec-3-en-9-ol, stereoisomer $$

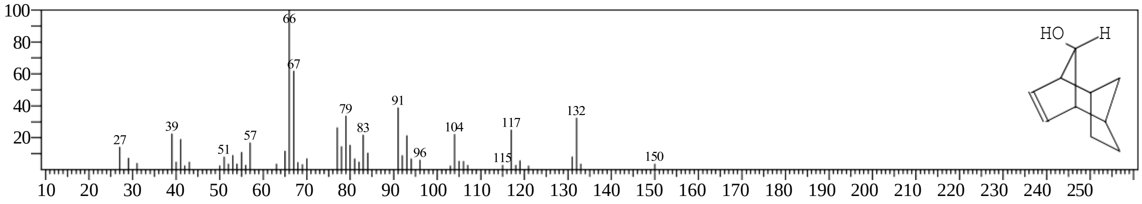

Hit#:3 Entry:13372 Library:NIST107.LIB
SI:79 Formula:C10H14O CAS:27137-33-3 MolWeight:150 RetIndex:0
CompName:Dicyclopentenyl alcohol $$ 4,7-Methano-1H-indenol, 3a,4,5,6,7,7a-hexahydro- $$

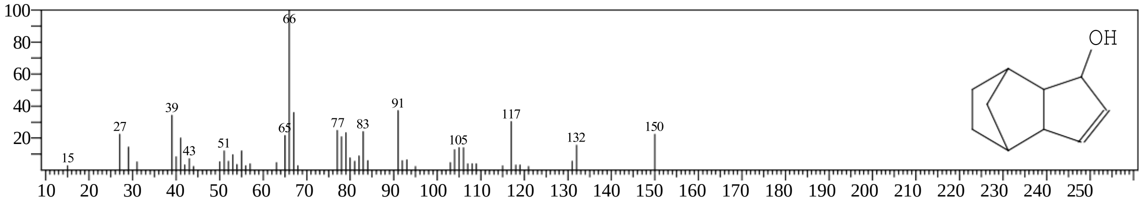

Hit#:4 Entry:31032 Library:NIST107.LIB
SI:78 Formula:C12H16O2 CAS:119405-11-7 MolWeight:192 RetIndex:0
CompName:Tricyclo[4.2.1.1(2,5)]dec-3-en-9-ol, acetate, stereoisomer $$

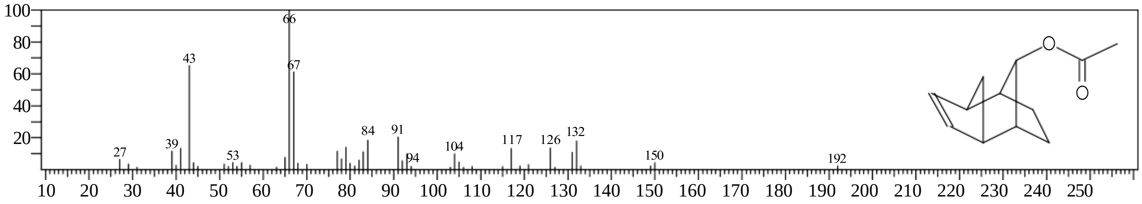

Hit#:5 Entry:13334 Library:NIST107.LIB
SI:76 Formula:C10H14O CAS:0-00-0 MolWeight:150 RetIndex:0
CompName:Tricyclo[4.2.1.1(2,5)]dec-7-en-9-ol $$

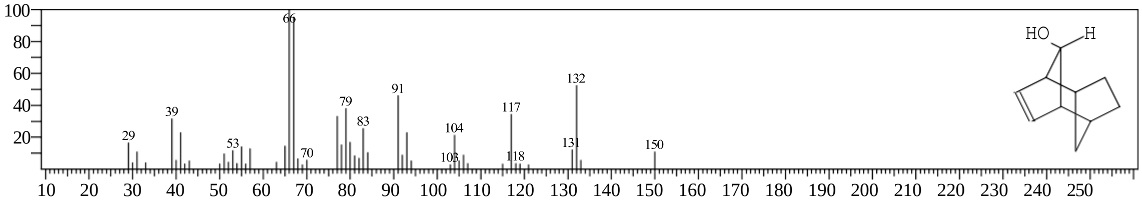

<< Target >>
Line#:77 R.Time:13.405(Scan#:2682) MassPeaks:129
RawMode:Averaged 13.400-13.410(2681-2683) BasePeak:159.30(1067491)
BG Mode:Calc. from Peak

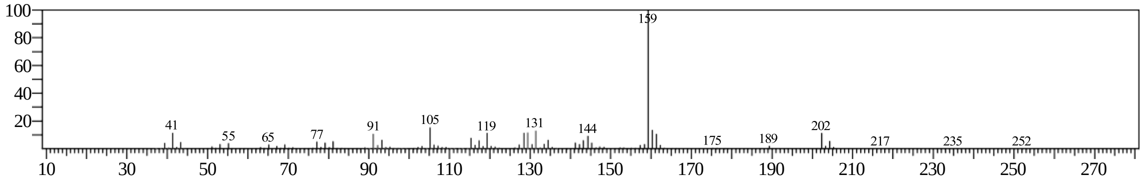

Hit#:1 Entry:35548 Library:NIST107.LIB
SI:85 Formula:C15H22 CAS:483-77-2 MolWeight:202 RetIndex:0
CompName:Naphthalene, 1,2,3,4-tetrahydro-1,6-dimethyl-4-(1-methylethyl)-, (1S-cis)- $$ Cadina-1,3,5-triene $$ (-)-Calamenene $$ L-calamenene $$ Cala

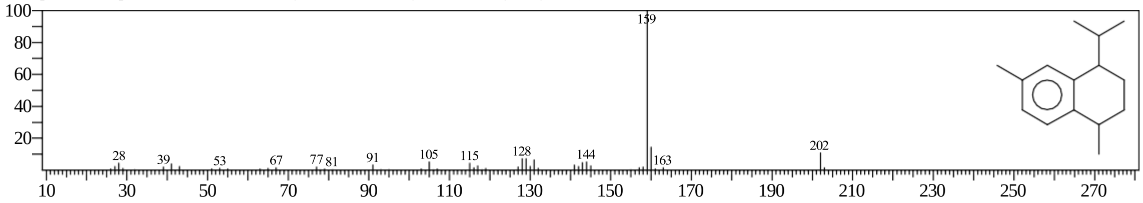

Hit#:2 Entry:23624 Library:NIST107.LIB
SI:78 Formula:C13H18 CAS:475-03-6 MolWeight:174 RetIndex:0
CompName:Naphthalene, 1,2,3,4-tetrahydro-1,1,6-trimethyl- $$ .alpha.-Ionene $$ Ionene $$ Naphthalene, 1,2,3,4-tetrahydro-1,6,6-trimethyl- $$ 1,1,6-Trim

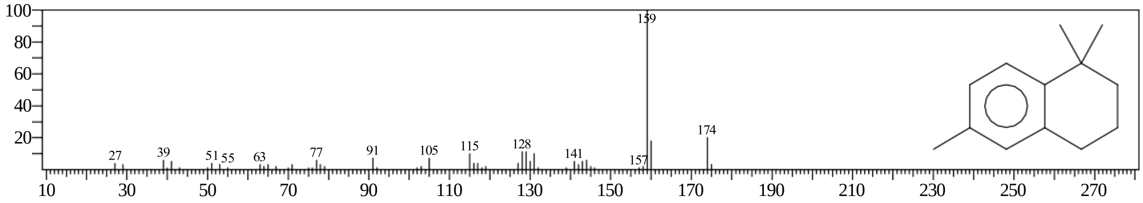

Hit#:3 Entry:23611 Library:NIST107.LIB
SI:77 Formula:C13H18 CAS:30316-36-0 MolWeight:174 RetIndex:0
CompName:Naphthalene, 1,2,3,4-tetrahydro-1,6,8-trimethyl- $$

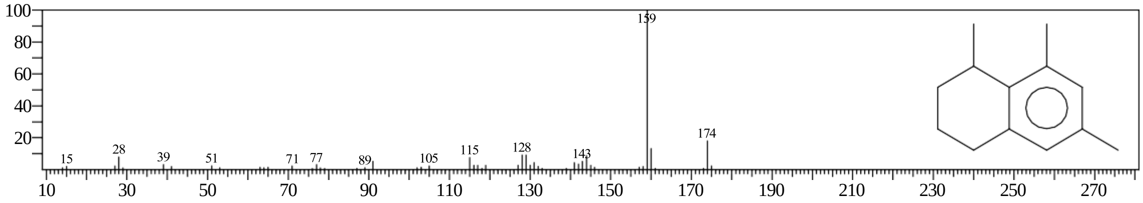

Hit#:4 Entry:62826 Library:NIST107.LIB
SI:77 Formula:C20H32 CAS:55255-58-8 MolWeight:272 RetIndex:0
CompName:Naphthalene, 1,2,3,4-tetrahydro-5,8-dimethyl-1-octyl- $$

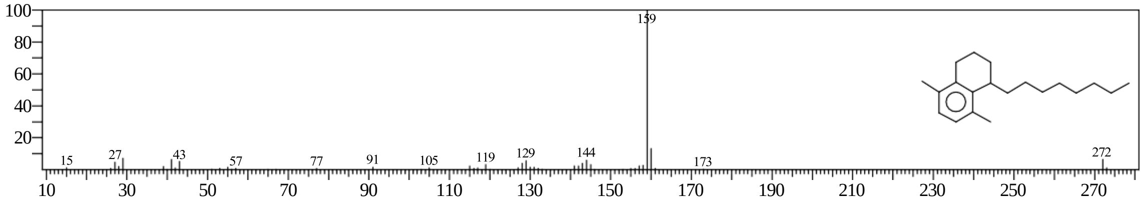

Hit#:5 Entry:23613 Library:NIST107.LIB
SI:75 Formula:C13H18 CAS:21693-51-6 MolWeight:174 RetIndex:0
CompName:Naphthalene, 1,2,3,4-tetrahydro-1,5,8-trimethyl- $$

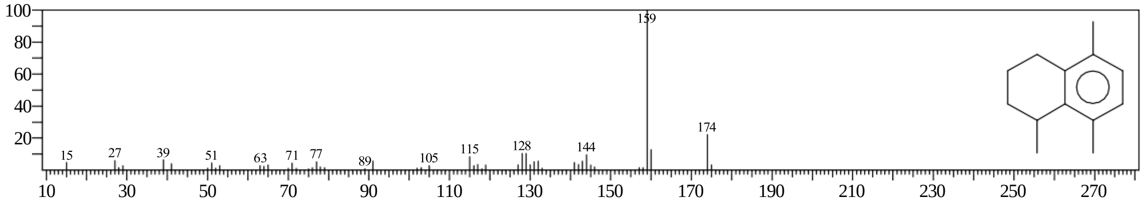

<< Target >>
Line#:78 R.Time:13.510(Scan#:2703) MassPeaks:75
RawMode:Averaged 13.505-13.515(2702-2704) BasePeak:57.10(47655)
BG Mode:Calc. from Peak

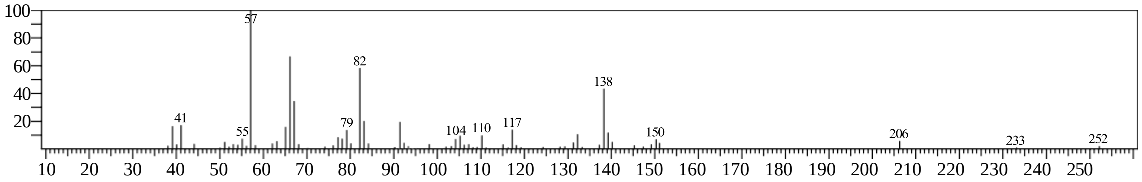

Hit#:1 Entry:37041 Library:NIST107.LIB
SI:78 Formula:C12H14O3 CAS:0-00-0 MolWeight:206 RetIndex:0
CompName:Indan-1,3-diol monopropionate $$

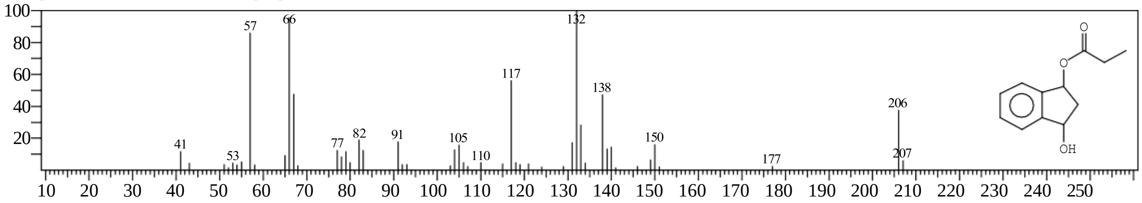

Hit#:2 Entry:13372 Library:NIST107.LIB
SI:72 Formula:C10H14O CAS:27137-33-3 MolWeight:150 RetIndex:0
CompName:Dicyclopentenyl alcohol $$ 4,7-Methano-1H-indenol, 3a,4,5,6,7,7a-hexahydro- $$

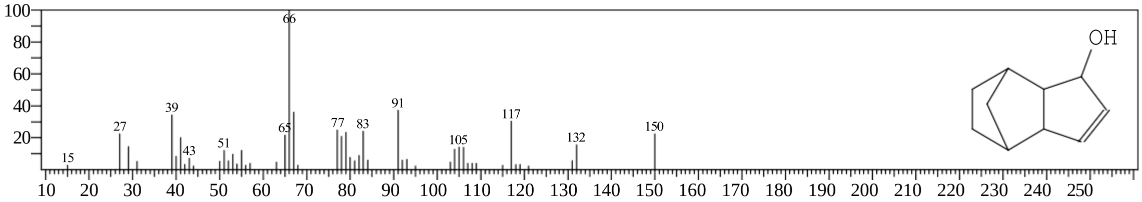

Hit#:3 Entry:13425 Library:NIST107.LIB
SI:70 Formula:C10H14O CAS:70220-93-8 MolWeight:150 RetIndex:0
CompName:Tricyclo[4.2.1.1(2,5)]dec-3-en-9-ol, stereoisomer $$

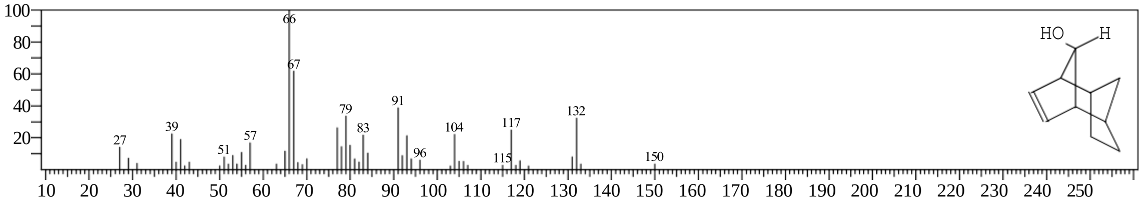

Hit#:4 Entry:32187 Library:NIST107.LIB
SI:69 Formula:C14H26 CAS:0-00-0 MolWeight:194 RetIndex:0
CompName:2,2,7,7-Tetramethyl-4,5-dimethyleneoctane $$

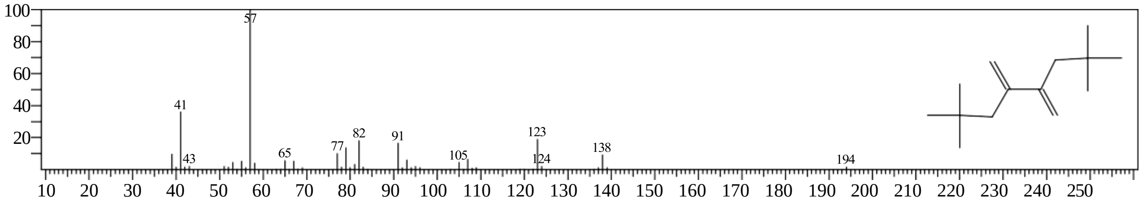

Hit#:5 Entry:13334 Library:NIST107.LIB
SI:69 Formula:C10H14O CAS:0-00-0 MolWeight:150 RetIndex:0
CompName:Tricyclo[4.2.1.1(2,5)]dec-7-en-9-ol $$

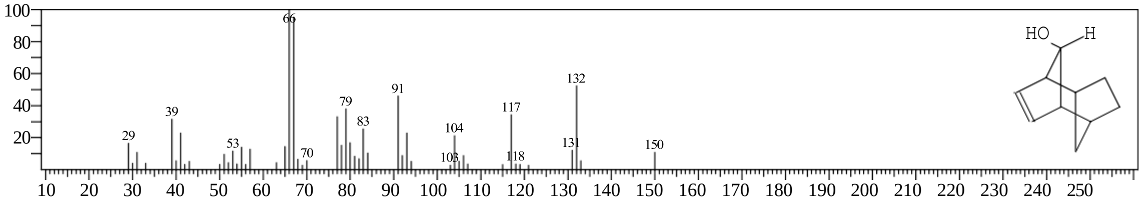

<< Target >>
Line#:79 R.Time:13.625(Scan#:2726) MassPeaks:67
RawMode:Averaged 13.620-13.630(2725-2727) BasePeak:105.20(10559)
BG Mode:Calc. from Peak

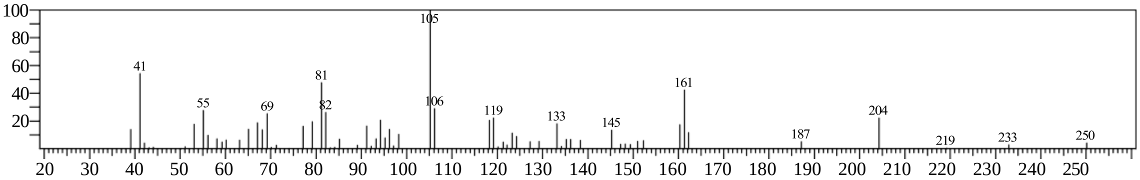

Hit#:1 Entry:36448 Library:NIST107.LIB
SI:73 Formula:C15H24 CAS:17627-24-6 MolWeight:204 RetIndex:0
CompName:Naphthalene, 1,2,4a,5,6,8a-hexahydro-4,7-dimethyl-1-(1-methylethyl)-,[1R-(1.alpha.,4a.alpha.,8a.alpha.)]- $$

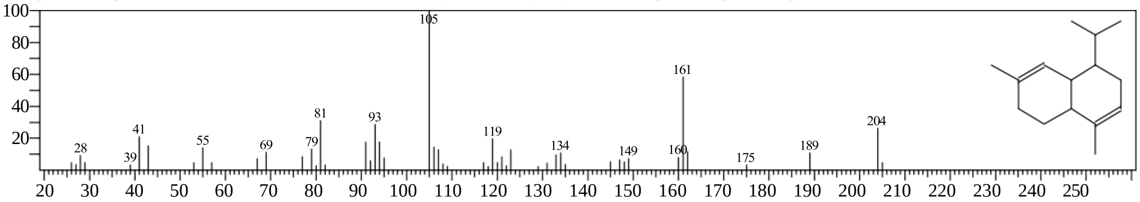

Hit#:2 Entry:36473 Library:NIST107.LIB
SI:73 Formula:C15H24 CAS:483-75-0 MolWeight:204 RetIndex:0
CompName:Naphthalene, 1,2,4a,5,6,8a-hexahydro-4,7-dimethyl-1-(1-methylethyl)- $$

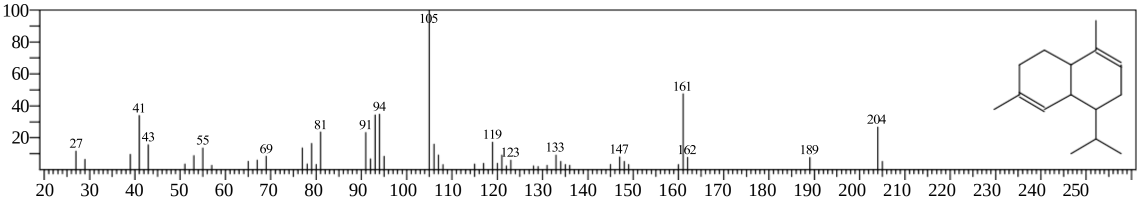

Hit#:3 Entry:36407 Library:NIST107.LIB
SI:72 Formula:C15H24 CAS:10208-80-7 MolWeight:204 RetIndex:0
CompName:.alpha.-Muurolene $$

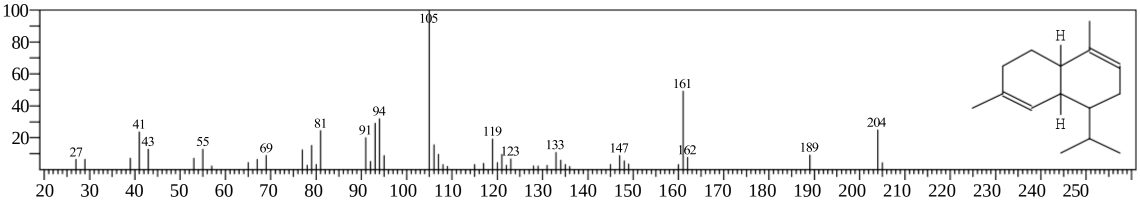

Hit#:4 Entry:36456 Library:NIST107.LIB
SI:71 Formula:C15H24 CAS:31983-22-9 MolWeight:204 RetIndex:0
CompName:Naphthalene, 1,2,4a,5,6,8a-hexahydro-4,7-dimethyl-1-(1-methylethyl)-, (1.alpha.,4a.alpha.,8a.alpha.)- $$ 1.Xi.,6.xi.,7.xi.-Cadina-4,9-diene $$ .a

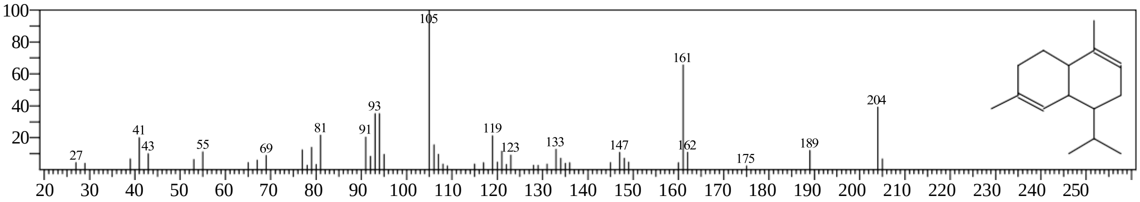

Hit#:5 Entry:36406 Library:NIST107.LIB
SI:71 Formula:C15H24 CAS:24406-05-1 MolWeight:204 RetIndex:0
CompName:Naphthalene, 1,2,4a,5,6,8a-hexahydro-4,7-dimethyl-1-(1-methylethyl)-, [1s-(1.alpha.,4a.beta.,8a.alpha.)]- $$

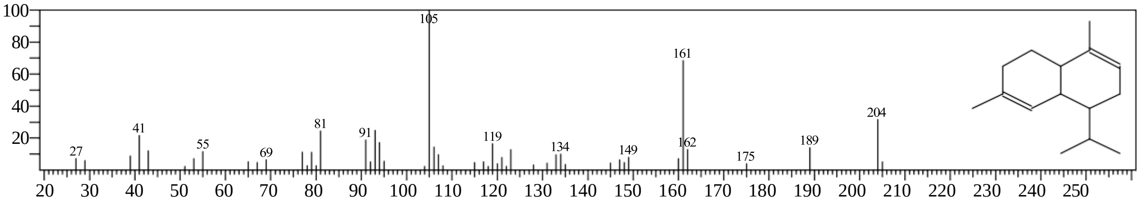

<< Target >>
Line#:80 R.Time:13.670(Scan#:2735) MassPeaks:78
RawMode:Averaged 13.665-13.675(2734-2736) BasePeak:57.15(15936)
BG Mode:Calc. from Peak

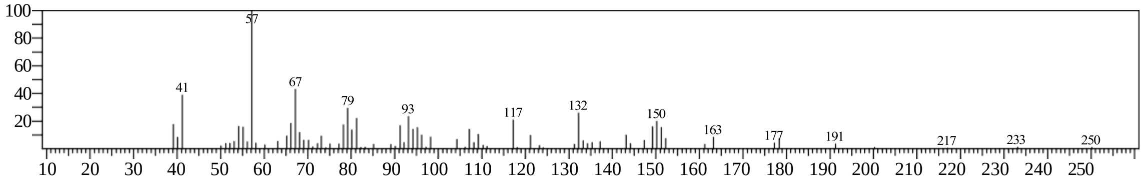

Hit#:1 Entry:20869 Library:NIST107.LIB
SI:73 Formula:C10H16O2 CAS:19213-99-1 MolWeight:168 RetIndex:0
CompName:Tricyclo[3.3.1.1(3,7)]decane-2,4-diol $$

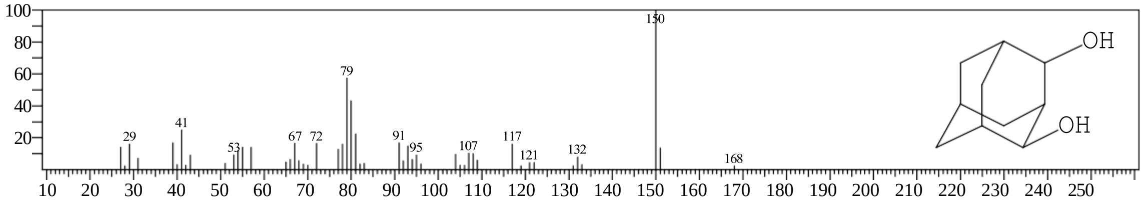

Hit#:2 Entry:19192 Library:NIST107.LIB
SI:72 Formula:C12H20 CAS:55976-09-5 MolWeight:164 RetIndex:0
CompName:Naphthalene, 1,2,3,4,4a,5,6,8a-octahydro-4a,8-dimethyl- $$

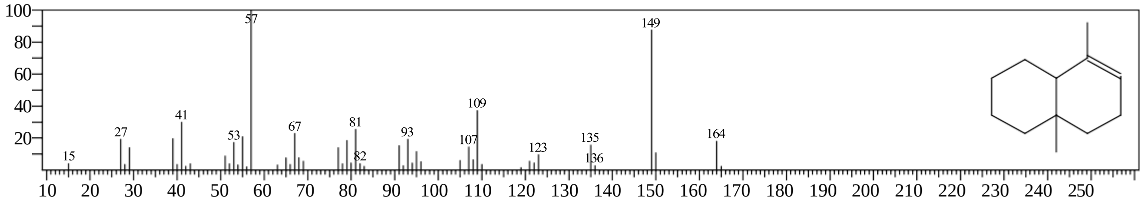

Hit#:3 Entry:13446 Library:NIST107.LIB
SI:72 Formula:C10H14O CAS:73679-80-8 MolWeight:150 RetIndex:0
CompName:Tricyclo[4.4.0.0(3,8)]decan-2-one $$

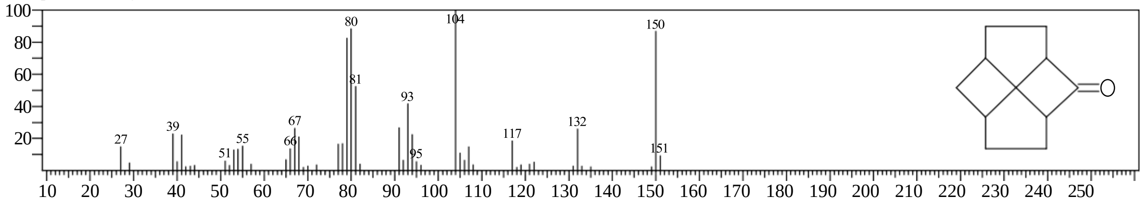

Hit#:4 Entry:59549 Library:NIST107.LIB
SI:71 Formula:C12H15F3O3 CAS:106988-62-9 MolWeight:264 RetIndex:0
CompName:Acetic acid, trifluoro-, octahydro-4-hydroxy-1,5-methano-1H-inden-1-yl ester (1.alpha.,3a.beta.,4.beta.,5.beta.,7a.beta.)- $$

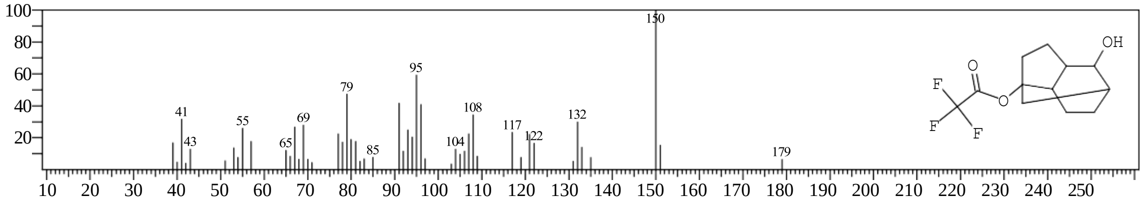

Hit#:5 Entry:38948 Library:NIST107.LIB
SI:71 Formula:C12H18O3 CAS:0-00-0 MolWeight:210 RetIndex:0
CompName:Adamantane, 2-hydroperoxy-2-(2-oxiranyl)- $$

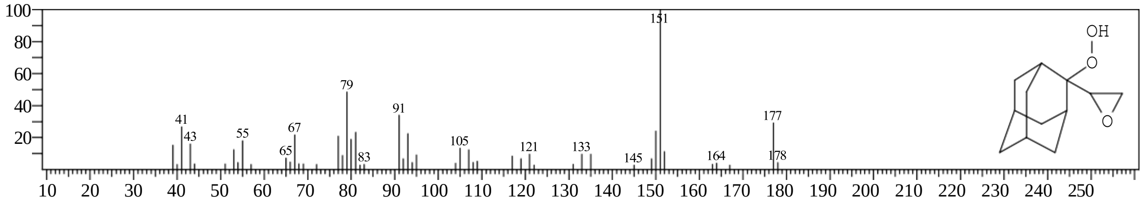

<< Target >>
Line#:81 R.Time:13.770(Scan#:2755) MassPeaks:82
RawMode:Averaged 13.765-13.775(2754-2756) BasePeak:82.20(15105)
BG Mode:Calc. from Peak

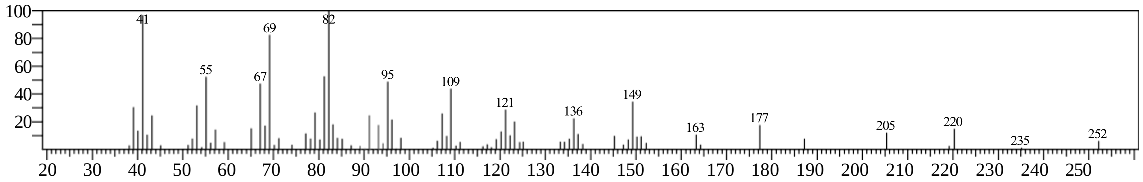

Hit#:1 Entry:37323 Library:NIST107.LIB
SI:82 Formula:C15H26 CAS:13567-54-9 MolWeight:206 RetIndex:0
CompName:1H-3a,7-Methanoazulene, octahydro-3,6,8,8-tetramethyl-, [3R-(3.alpha.,3a.beta.,6.alpha.,7.beta.,8a.alpha.)]- $$ Cedrane $$

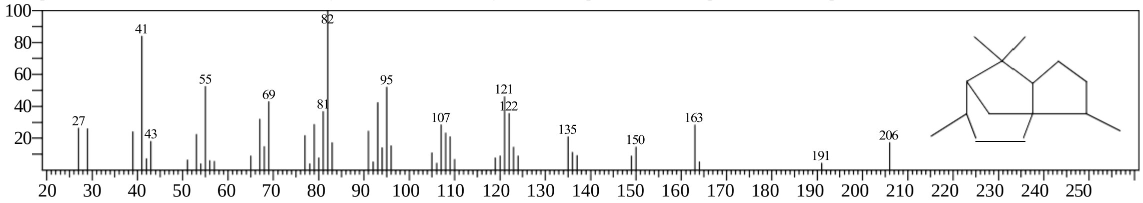

Hit#:2 Entry:43186 Library:NIST107.LIB
SI:81 Formula:C15H24O CAS:0-00-0 MolWeight:220 RetIndex:0
CompName:Diepicedrene-1-oxide $$

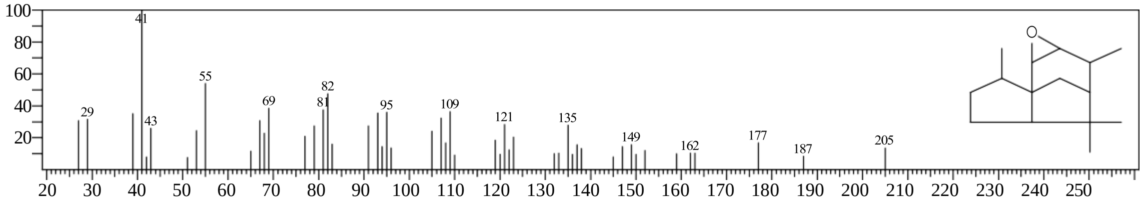

Hit#:3 Entry:37339 Library:NIST107.LIB
SI:81 Formula:C15H26 CAS:62337-96-6 MolWeight:206 RetIndex:0
CompName:Cyclohexane, 1,1,2-trimethyl-3,5-bis(1-methylethenyl)-, (2.alpha.,3.alpha.,5.beta.)- $$

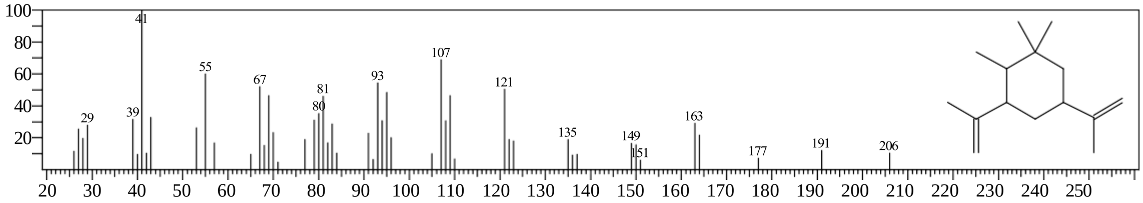

Hit#:4 Entry:37334 Library:NIST107.LIB
SI:80 Formula:C15H26 CAS:69239-71-0 MolWeight:206 RetIndex:0
CompName:6,10-Dimethyl-3-(1-methylethylidene)-1-cyclodecene $$

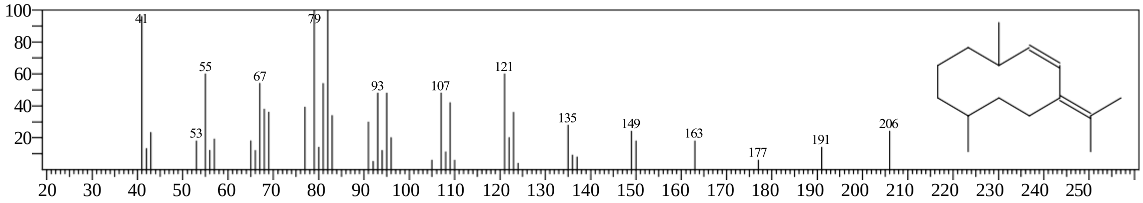

Hit#:5 Entry:43180 Library:NIST107.LIB
SI:80 Formula:C15H24O CAS:0-00-0 MolWeight:220 RetIndex:0
CompName:Diepi-.alpha.-cedrene epoxide $$

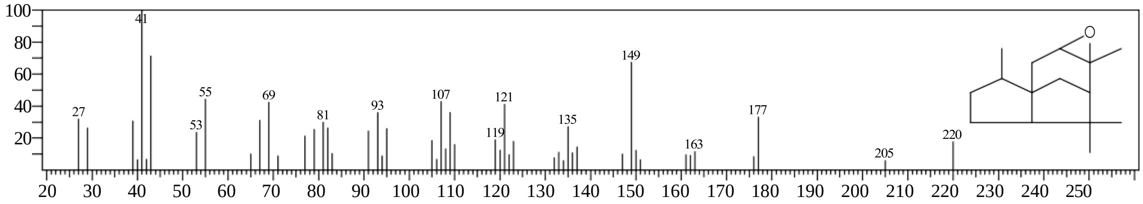

<< Target >>
Line#:82 R.Time:13.895(Scan#:2780) MassPeaks:135
RawMode:Averaged 13.890-13.900(2779-2781) BasePeak:41.15(227290)
BG Mode:Calc. from Peak

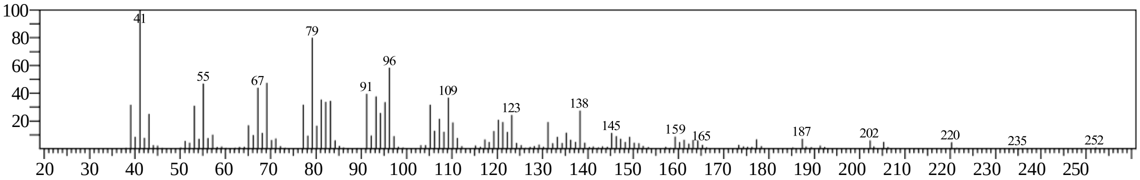

Hit#:1 Entry:43194 Library:NIST107.LIB
SI:87 Formula:C15H24O CAS:0-00-0 MolWeight:220 RetIndex:0
CompName:Caryophyllene oxide $$

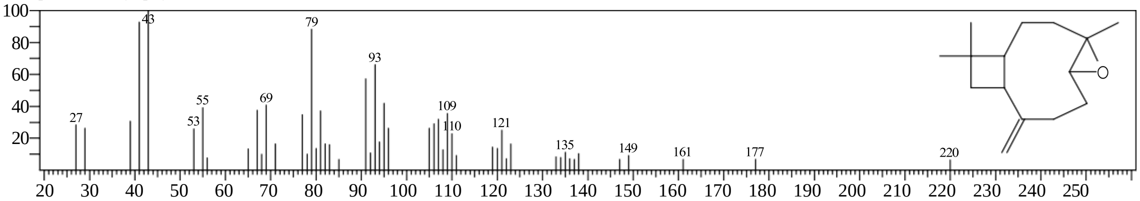

Hit#:2 Entry:43182 Library:NIST107.LIB
SI:84 Formula:C15H24O CAS:0-00-0 MolWeight:220 RetIndex:0
CompName:Aromadendrene oxide-(2) $$

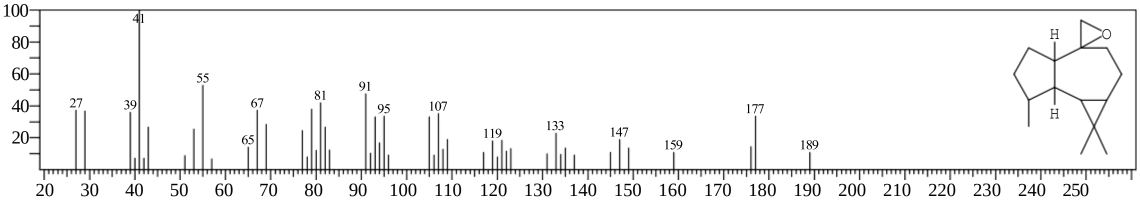

Hit#:3 Entry:43181 Library:NIST107.LIB
SI:83 Formula:C15H24O CAS:0-00-0 MolWeight:220 RetIndex:0
CompName:Aromadendrene oxide-(1) $$

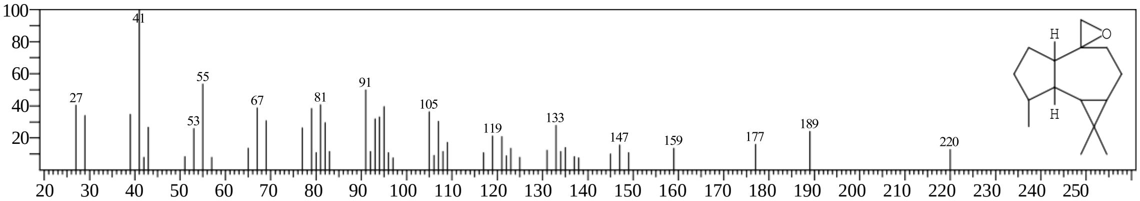

Hit#:4 Entry:37348 Library:NIST107.LIB
SI:82 Formula:C15H26 CAS:19078-35-4 MolWeight:206 RetIndex:0
CompName:1H-3a,7-Methanoazulene, octahydro-1,4,9,9-tetramethyl- $$ Patchulane $$ 1H-3a,7-Methanoazulene, 2,3,4,5,6,7.alpha.,8,8.alpha.-octahydro-1,

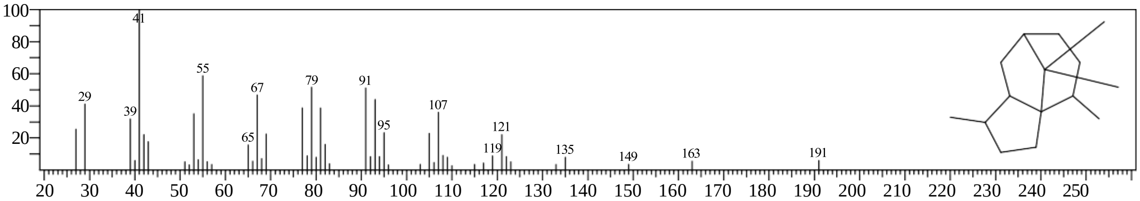

Hit#:5 Entry:76888 Library:NIST107.LIB
SI:82 Formula:C21H34O2 CAS:59149-01-8 MolWeight:318 RetIndex:0
CompName:Methyl (Z)-5,11,14,17-eicosatetraenoate $$

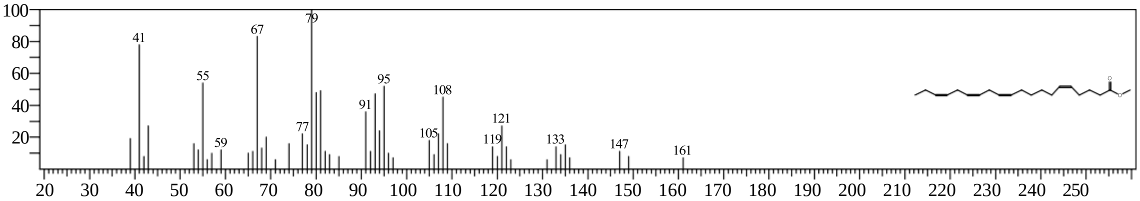

<< Target >>
Line#:83 R.Time:13.965(Scan#:2794) MassPeaks:100
RawMode:Averaged 13.960-13.970(2793-2795) BasePeak:43.10(908282)
BG Mode:Calc. from Peak

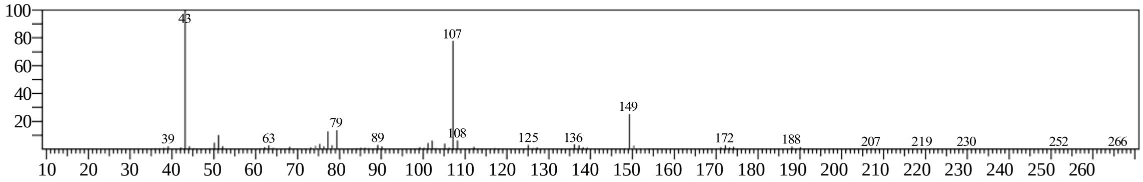

Hit#:1 Entry:60171 Library:NIST107.LIB
SI:93 Formula:C10H9Cl3O2 CAS:0-00-0 MolWeight:266 RetIndex:0
CompName:Rose acetate $$

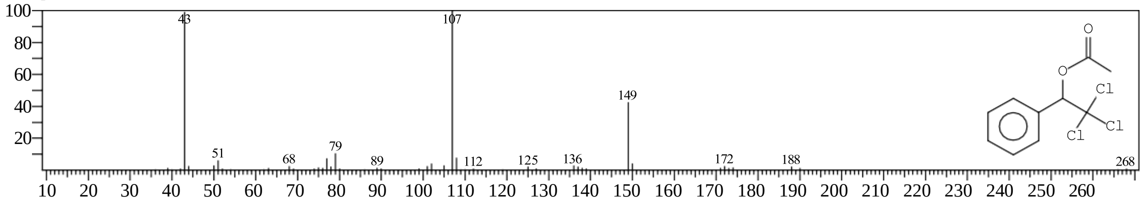

Hit#:2 Entry:60172 Library:NIST107.LIB
SI:93 Formula:C10H9Cl3O2 CAS:90-17-5 MolWeight:266 RetIndex:0
CompName:Benzenemethanol, .alpha.-(trichloromethyl)-, acetate $$ .alpha.-(Trichloromethyl)benzyl acetate $$ Acetic acid, .alpha.-(trichloromethyl)benzyl

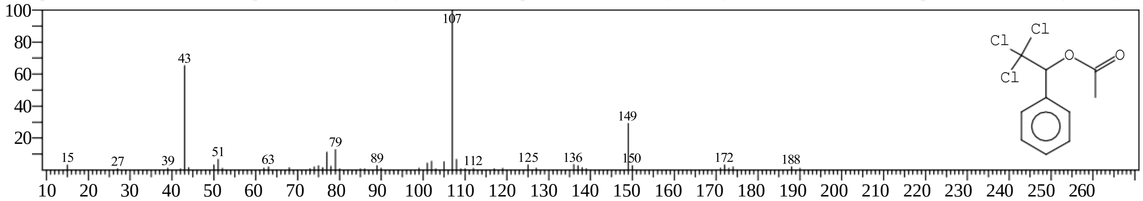

Hit#:3 Entry:65544 Library:NIST107.LIB
SI:81 Formula:C16H15N3O2 CAS:0-00-0 MolWeight:281 RetIndex:0
CompName:Acetic acid, 2-azido-1,2-diphenylethyl ester $$

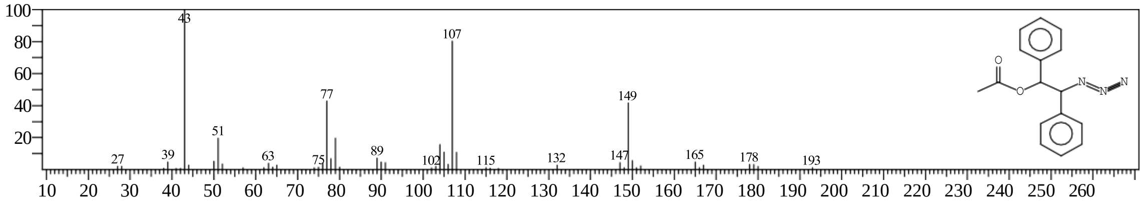

Hit#:4 Entry:37950 Library:NIST107.LIB
SI:78 Formula:C11H12O4 CAS:947-94-4 MolWeight:208 RetIndex:0
CompName:Benzeneacetic acid, .alpha.-(acetyloxy)-, methyl ester $$ Mandelic acid, methyl ester, acetate $$

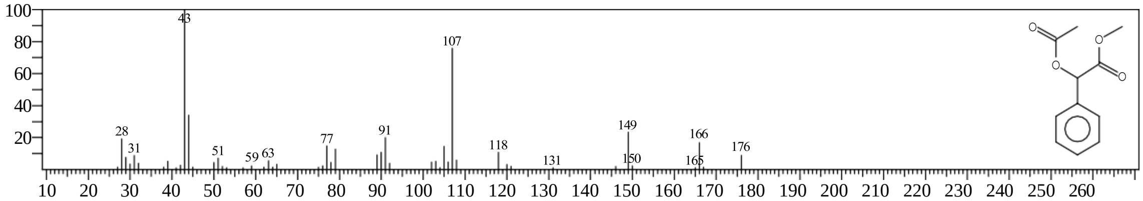

Hit#:5 Entry:37931 Library:NIST107.LIB
SI:76 Formula:C11H12O4 CAS:27548-25-0 MolWeight:208 RetIndex:0
CompName:Benzoic acid, 4-[(acetyloxy)methyl]-, methyl ester $$ p-Toluic acid, .alpha.-hydroxy-, methyl ester, acetate $$ Methyl 4-(acetoxymethyl)benzoat

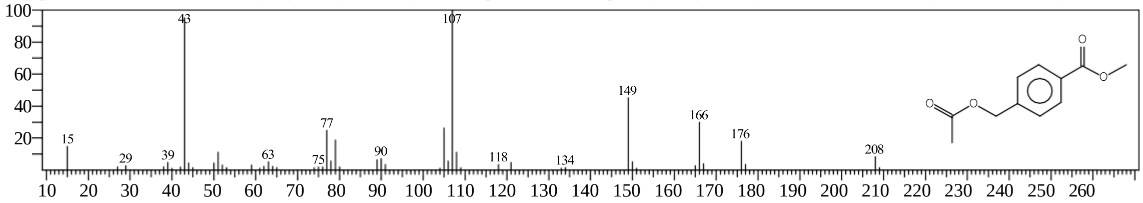

<< Target >>
Line#:84 R.Time:14.205(Scan#:2842) MassPeaks:117
RawMode:Averaged 14.200-14.210(2841-2843) BasePeak:111.25(58918)
BG Mode:Calc. from Peak

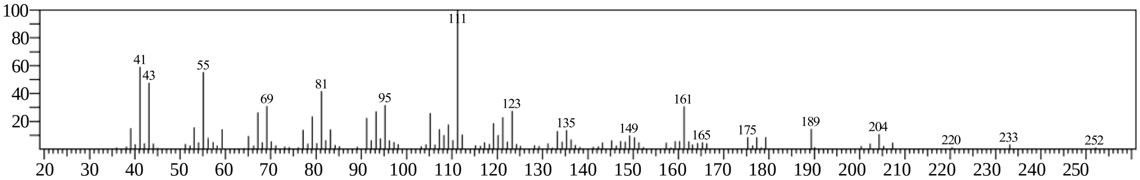

Hit#:1 Entry:44174 Library:NIST107.LIB
SI:82 Formula:C15H26O CAS:21414-53-9 MolWeight:222 RetIndex:0
CompName:1-Phenanthrenemethanol, 1,2,3,4,4a,5,6,9,10,10a-decahydro-1,4a-dimethyl-7-(1-methylethyl)-, [1R-(1.alpha.,4a.beta.,10a.alpha.)]- $$

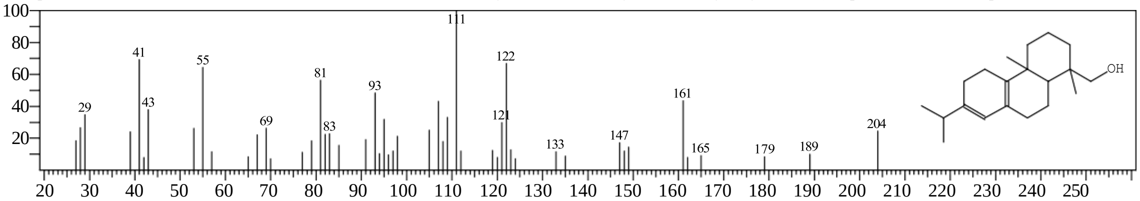

Hit#:2 Entry:44099 Library:NIST107.LIB
SI:80 Formula:C15H26O CAS:0-00-0 MolWeight:222 RetIndex:0
CompName:Caryophyllenyl alcohol $$

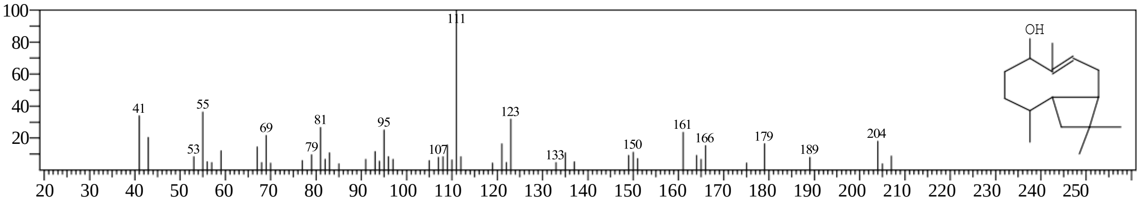

Hit#:3 Entry:44116 Library:NIST107.LIB
SI:80 Formula:C15H26O CAS:0-00-0 MolWeight:222 RetIndex:0
CompName:Viridiflorol $$

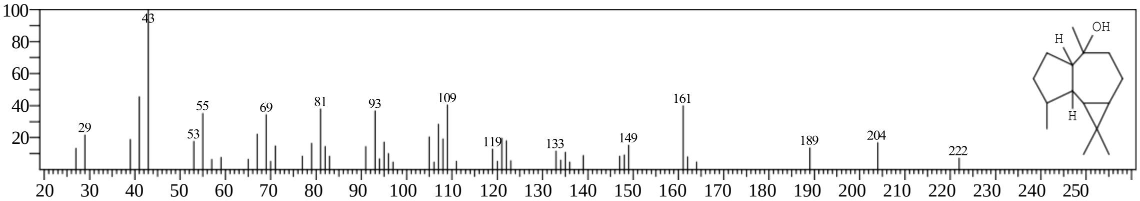

Hit#:4 Entry:44114 Library:NIST107.LIB
SI:80 Formula:C15H26O CAS:0-00-0 MolWeight:222 RetIndex:0
CompName:Globulol $$

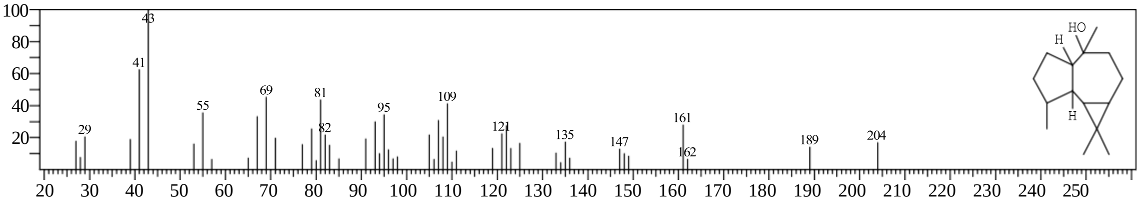

Hit#:5 Entry:44183 Library:NIST107.LIB
SI:79 Formula:C15H26O CAS:95975-84-1 MolWeight:222 RetIndex:0
CompName:4Ah-cycloprop[e]azulen-4a-ol, decahydro-1,1,4,7-tetramethyl-, [1ar-(1 $$

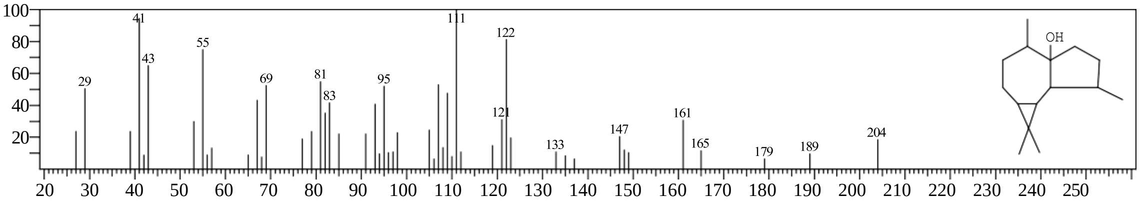

<< Target >>
Line#:85 R.Time:14.445(Scan#:2890) MassPeaks:150
RawMode:Averaged 14.440-14.450(2889-2891) BasePeak:41.15(239125)
BG Mode:Calc. from Peak

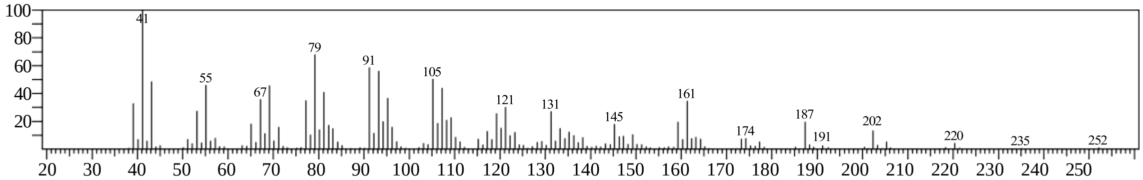

Hit#:1 Entry:43194 Library:NIST107.LIB
SI:87 Formula:C15H24O CAS:0-00-0 MolWeight:220 RetIndex:0
CompName:Caryophyllene oxide $$

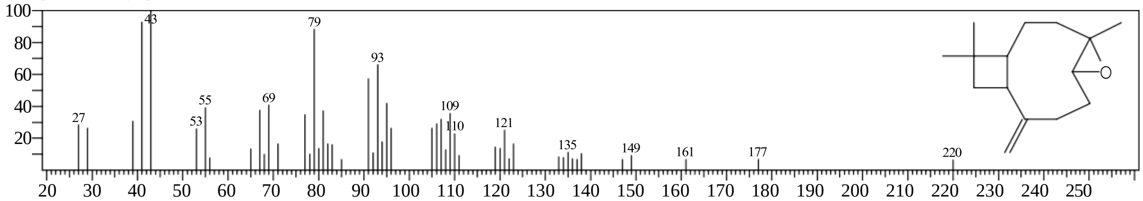

Hit#:2 Entry:43197 Library:NIST107.LIB
SI:85 Formula:C15H24O CAS:0-00-0 MolWeight:220 RetIndex:0
CompName:Isoaromadendrene epoxide $$

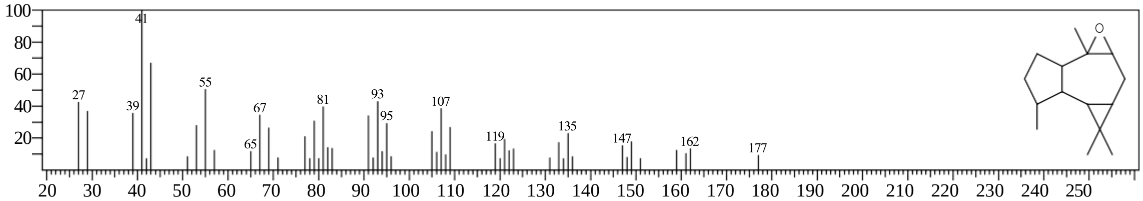

Hit#:3 Entry:43187 Library:NIST107.LIB
SI:84 Formula:C15H24O CAS:0-00-0 MolWeight:220 RetIndex:0
CompName:Alloaromadendrene oxide-(2) $$

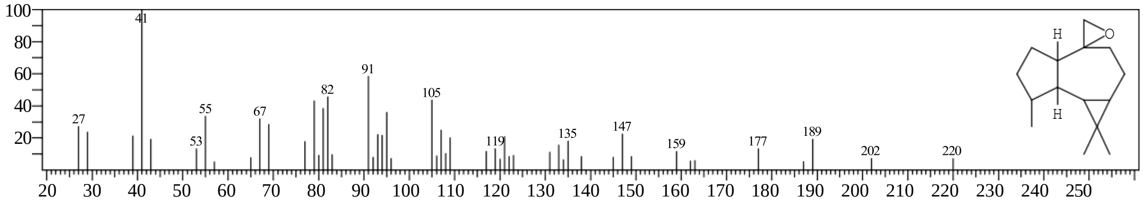

Hit#:4 Entry:43196 Library:NIST107.LIB
SI:84 Formula:C15H24O CAS:0-00-0 MolWeight:220 RetIndex:0
CompName:Longipinocarveol, trans- $$

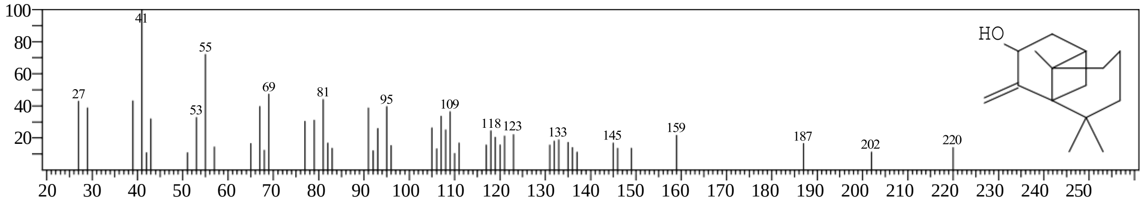

Hit#:5 Entry:43188 Library:NIST107.LIB
SI:83 Formula:C15H24O CAS:0-00-0 MolWeight:220 RetIndex:0
CompName:Alloaromadendrene oxide-(1) $$

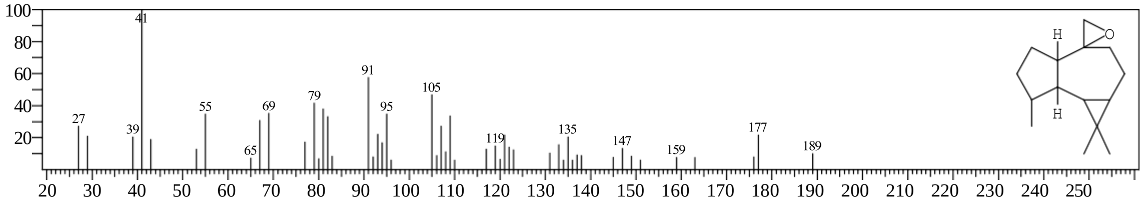

<< Target >>
Line#:86 R.Time:14.730(Scan#:2947) MassPeaks:92
RawMode:Averaged 14.725-14.735(2946-2948) BasePeak:149.20(51418)
BG Mode:Calc. from Peak

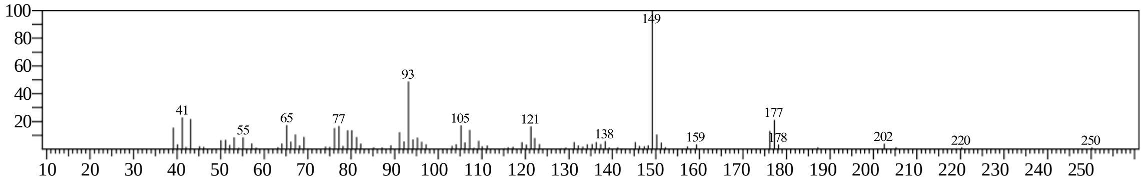

Hit#:1 Entry:46473 Library:NIST107.LIB
SI:77 Formula:C10H13BrO CAS:20098-20-8 MolWeight:228 RetIndex:0
CompName:5-Bromoadamantan-2-one $$

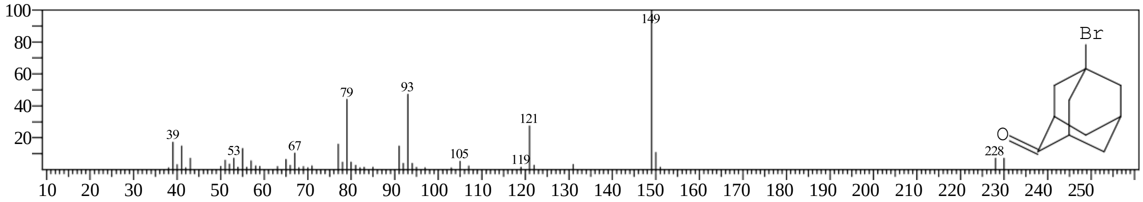

Hit#:2 Entry:19175 Library:NIST107.LIB
SI:76 Formula:C12H20 CAS:702-79-4 MolWeight:164 RetIndex:0
CompName:Adamantane, 1,3-dimethyl- $$ Tricyclo[3.3.1.13,7]decane, 1,3-dimethyl- $$ 1,3-Dimethyladamantane $$ Tricyclo[3.3.1.1<3,7>]decane, 1,3-di

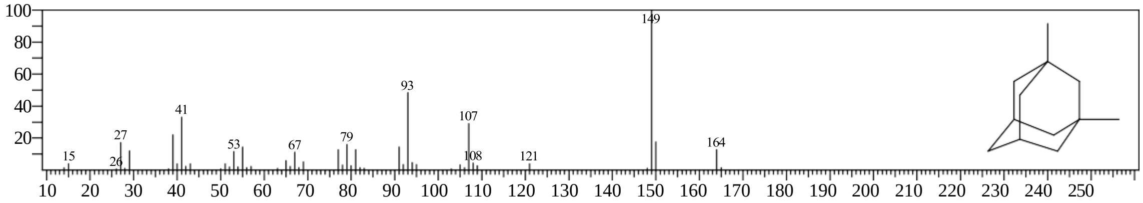

Hit#:3 Entry:46467 Library:NIST107.LIB
SI:76 Formula:C10H13BrO CAS:0-00-0 MolWeight:228 RetIndex:0
CompName:Myrtene acid bromide $$

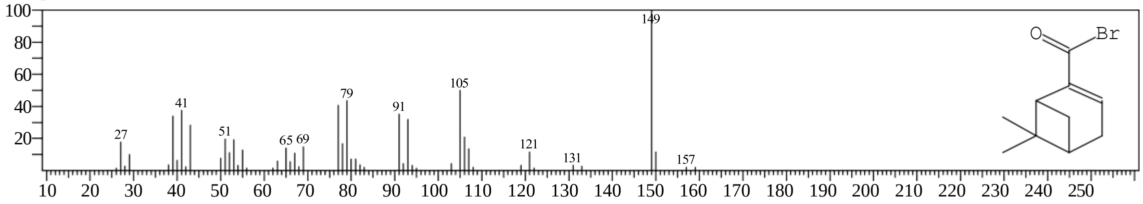

Hit#:4 Entry:19212 Library:NIST107.LIB
SI:75 Formula:C12H20 CAS:24145-89-9 MolWeight:164 RetIndex:0
CompName:Cis-1,4-dimethyladamantane $$

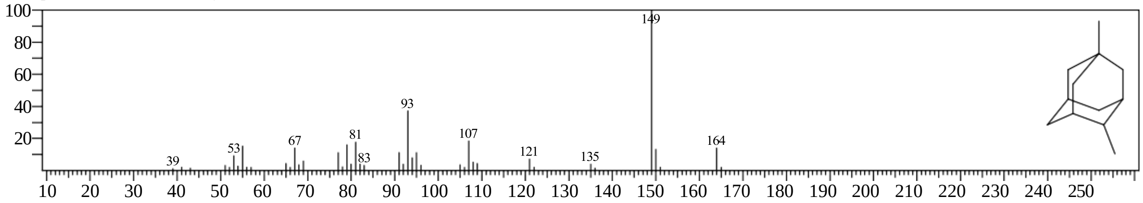

Hit#:5 Entry:19123 Library:NIST107.LIB
SI:75 Formula:C11H16O CAS:41437-92-7 MolWeight:164 RetIndex:0
CompName:4-(2-Methyl-cyclohex-1-enyl)-but-3-en-2-one $$

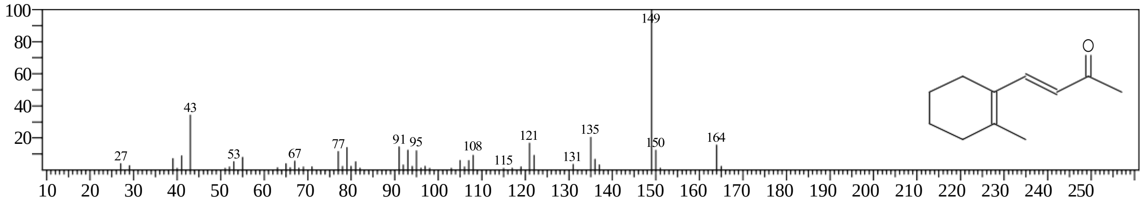

<< Target >>
Line#:87 R.Time:14.925(Scan#:2986) MassPeaks:139
RawMode:Averaged 14.920-14.930(2985-2987) BasePeak:41.15(203379)
BG Mode:Calc. from Peak

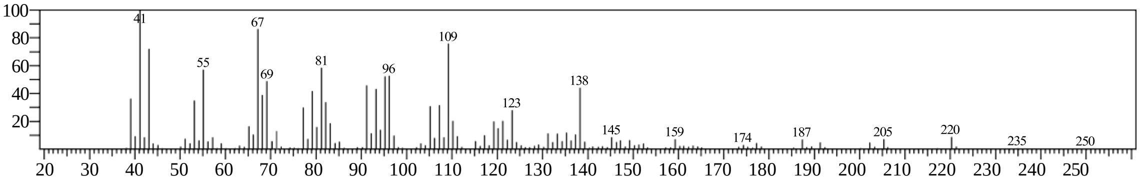

Hit#:1 Entry:43194 Library:NIST107.LIB
SI:85 Formula:C15H24O CAS:0-00-0 MolWeight:220 RetIndex:0
CompName:Caryophyllene oxide $$

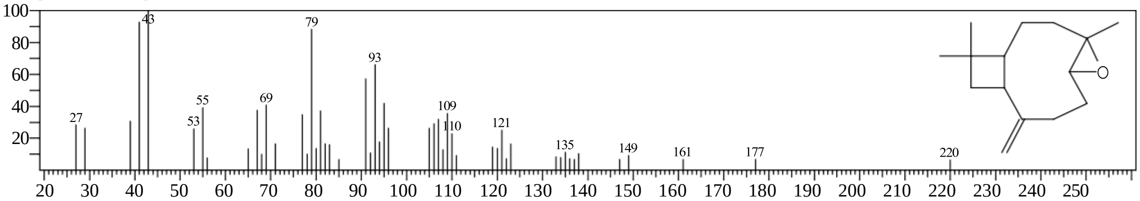

Hit#:2 Entry:19866 Library:NIST107.LIB
SI:83 Formula:C10H14O2 CAS:5951-57-5 MolWeight:166 RetIndex:0
CompName:Cyclopentaneacetaldehyde, 2-formyl-3-methyl-.alpha.-methylene- $$

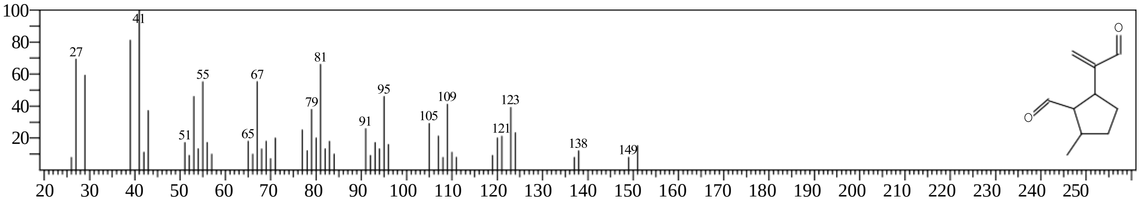

Hit#:3 Entry:15229 Library:NIST107.LIB
SI:83 Formula:C10H18O CAS:5944-20-7 MolWeight:154 RetIndex:0
CompName:3,6-Octadien-1-ol, 3,7-dimethyl-, (Z)- $$ Isogeraniol $$

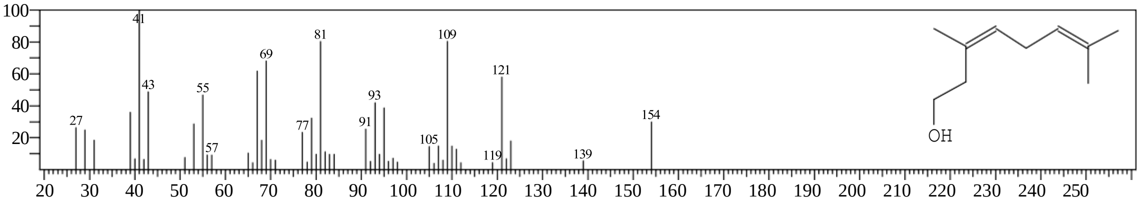

Hit#:4 Entry:43196 Library:NIST107.LIB
SI:82 Formula:C15H24O CAS:0-00-0 MolWeight:220 RetIndex:0
CompName:Longipinocarveol, trans- $$

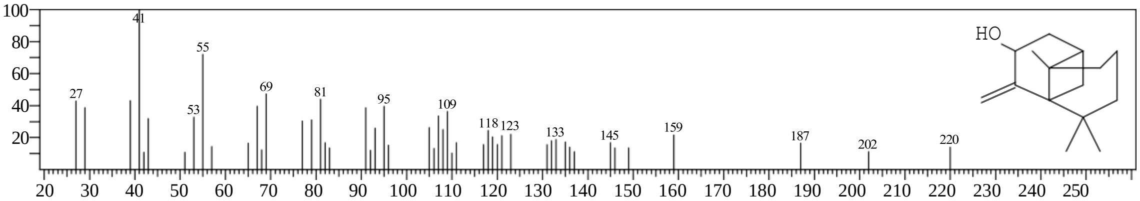

Hit#:5 Entry:43181 Library:NIST107.LIB
SI:82 Formula:C15H24O CAS:0-00-0 MolWeight:220 RetIndex:0
CompName:Aromadendrene oxide-(1) $$

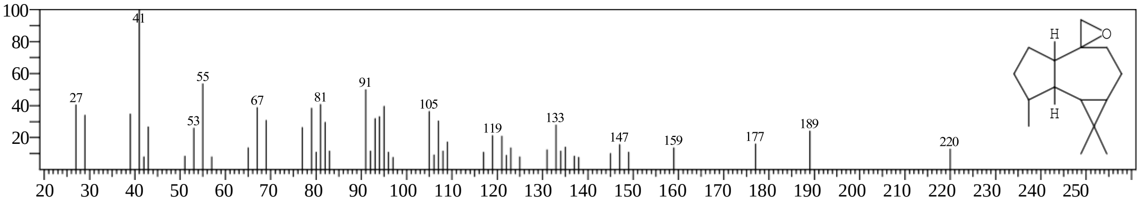

<< Target >>
Line#:88 R.Time:15.270(Scan#:3055) MassPeaks:73
RawMode:Averaged 15.265-15.275(3054-3056) BasePeak:81.15(10185)
BG Mode:Calc. from Peak

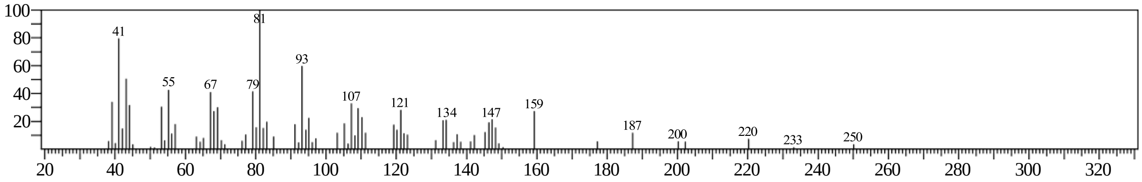

Hit#:1 Entry:43154 Library:NIST107.LIB
SI:78 Formula:C15H24O CAS:0-00-0 MolWeight:220 RetIndex:0
CompName:cis-Z-.alpha.-Bisabolene epoxide $$

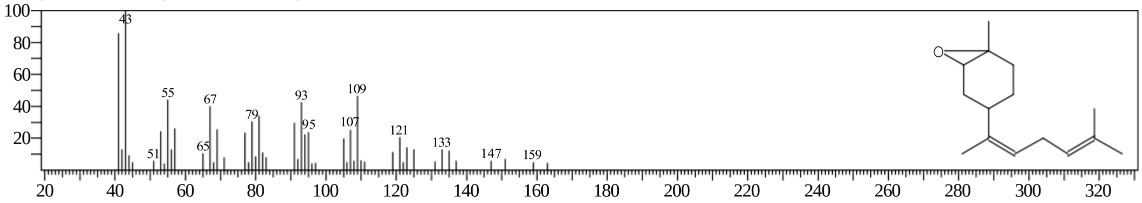

Hit#:2 Entry:79839 Library:NIST107.LIB
SI:77 Formula:C22H34O2 CAS:72150-74-4 MolWeight:330 RetIndex:0
CompName:Kauren-18-ol, acetate, (4.beta.)- $$

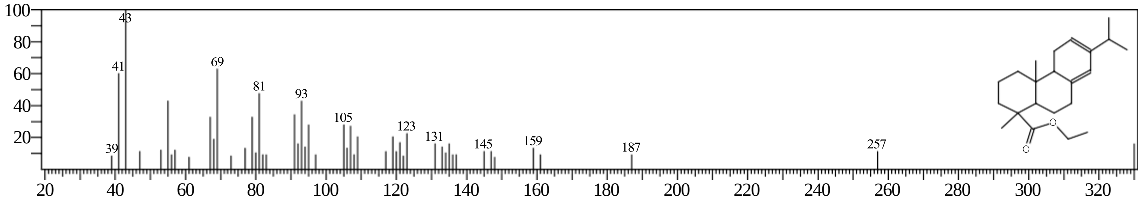

Hit#:3 Entry:43194 Library:NIST107.LIB
SI:77 Formula:C15H24O CAS:0-00-0 MolWeight:220 RetIndex:0
CompName:Caryophyllene oxide $$

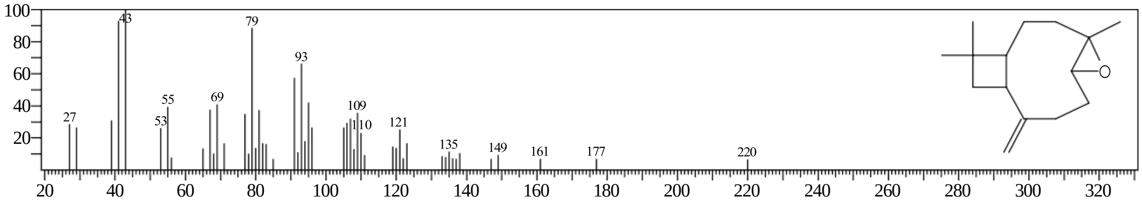

Hit#:4 Entry:43197 Library:NIST107.LIB
SI:77 Formula:C15H24O CAS:0-00-0 MolWeight:220 RetIndex:0
CompName:Isoaromadendrene epoxide $$

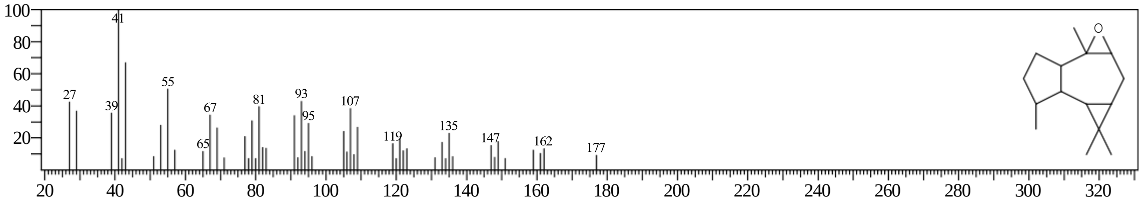

Hit#:5 Entry:36508 Library:NIST107.LIB
SI:77 Formula:C15H24 CAS:33880-83-0 MolWeight:204 RetIndex:0
CompName:Cyclohexane, 1-ethenyl-1-methyl-2,4-bis(1-methylethenyl)-, (1.alpha.,2.beta.,4.beta.)- $$

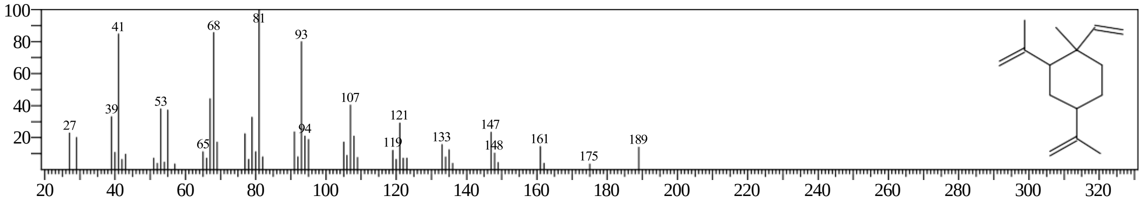

<< Target >>
Line#:89 R.Time:15.700(Scan#:3141) MassPeaks:84
RawMode:Averaged 15.695-15.705(3140-3142) BasePeak:83.15(88076)
BG Mode:Calc. from Peak

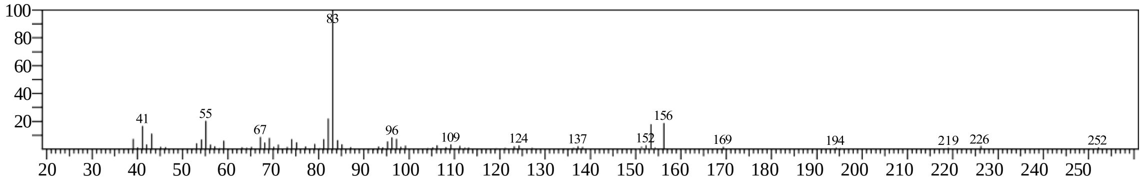

Hit#:1 Entry:45799 Library:NIST107.LIB
SI:89 Formula:C13H22O3 CAS:24851-98-7 MolWeight:226 RetIndex:0
CompName:Methyl dihydrojasmonate $$ Cyclopentaneacetic acid, 3-oxo-2-pentyl-, methyl ester $$

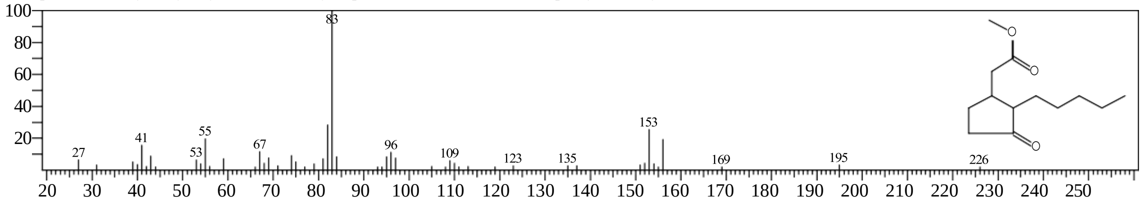

Hit#:2 Entry:16051 Library:NIST107.LIB
SI:81 Formula:C10H20O CAS:0-00-0 MolWeight:156 RetIndex:0
CompName:Isocitronellol $$

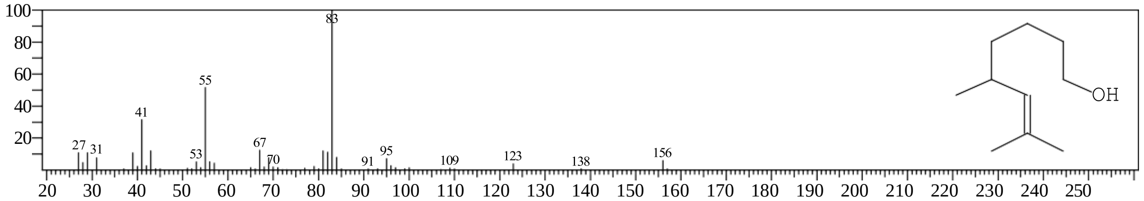

Hit#:3 Entry:49696 Library:NIST107.LIB
SI:79 Formula:C17H32 CAS:54833-31-7 MolWeight:236 RetIndex:0
CompName:Cyclohexane, 1,1'-(1,5-pentanediyl)bis- $$

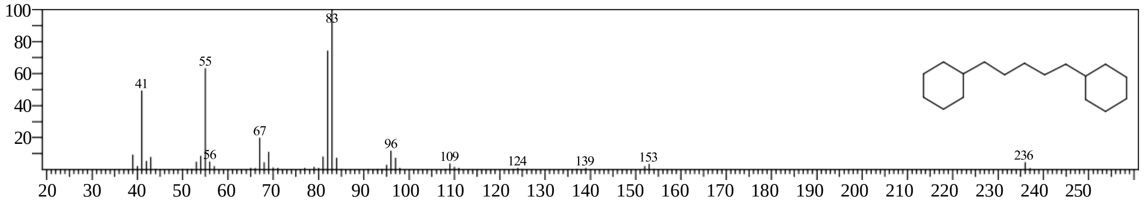

Hit#:4 Entry:33158 Library:NIST107.LIB
SI:79 Formula:C14H28 CAS:7090-88-2 MolWeight:196 RetIndex:0
CompName:3-Hexene, 2,5-dimethyl-3,4-bis(1-methylethyl)- $$

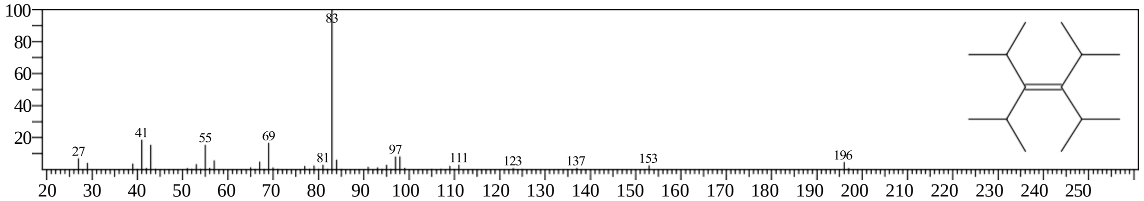

Hit#:5 Entry:21077 Library:NIST107.LIB
SI:78 Formula:C12H24 CAS:74421-03-7 MolWeight:168 RetIndex:0
CompName:2-Decene, 2,4-dimethyl- $$

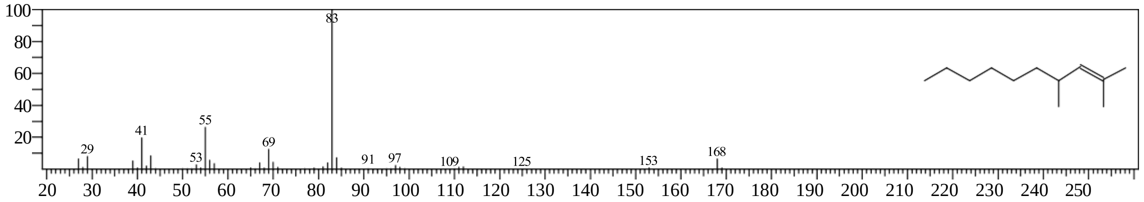

<< Target >>
Line#:90 R.Time:15.765(Scan#:3154) MassPeaks:70
RawMode:Averaged 15.760-15.770(3153-3155) BasePeak:157.25(49030)
BG Mode:Calc. from Peak

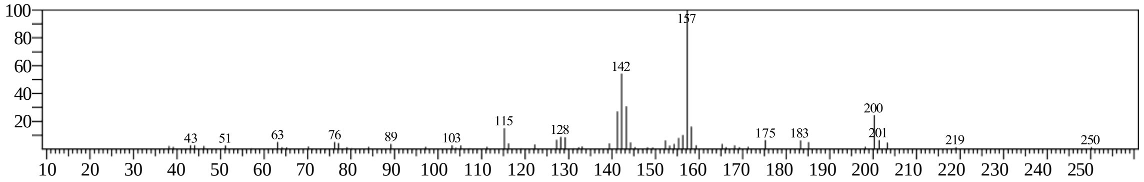

Hit#:1 Entry:35527 Library:NIST107.LIB
SI:78 Formula:C15H22 CAS:0-00-0 MolWeight:202 RetIndex:0
CompName:Cadala-1(10),3,8-triene $$

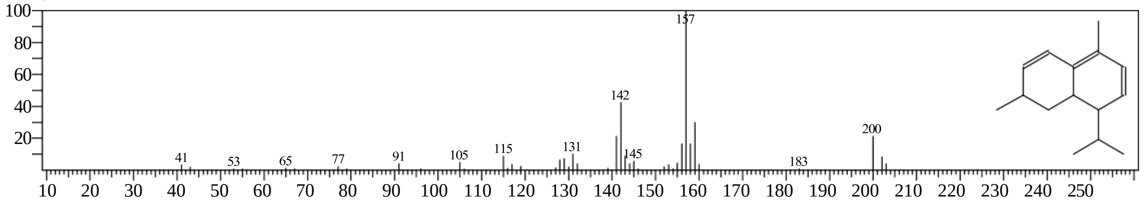

Hit#:2 Entry:49361 Library:NIST107.LIB
SI:76 Formula:C12H13Br CAS:0-00-0 MolWeight:236 RetIndex:0
CompName:1H-Indene, 3-(bromomethyl)-1,1-dimethyl- $$

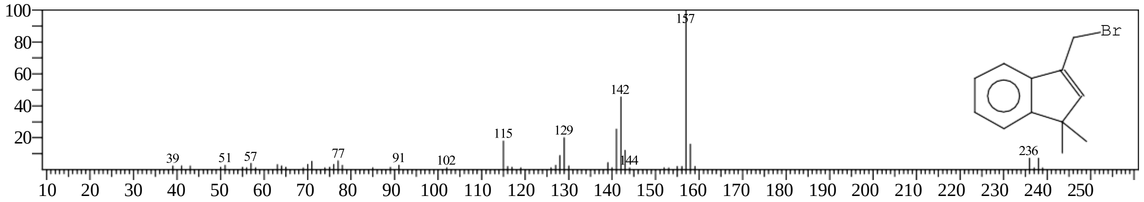

Hit#:3 Entry:22890 Library:NIST107.LIB
SI:75 Formula:C13H16 CAS:4506-36-9 MolWeight:172 RetIndex:0
CompName:Naphthalene, 1,2-dihydro-1,5,8-trimethyl- $$

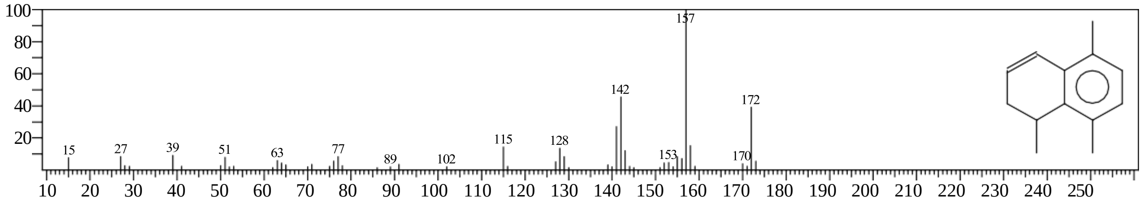

Hit#:4 Entry:22915 Library:NIST107.LIB
SI:74 Formula:C13H16 CAS:55682-80-9 MolWeight:172 RetIndex:0
CompName:Naphthalene, 1,2-dihydro-1,4,6-trimethyl- $$

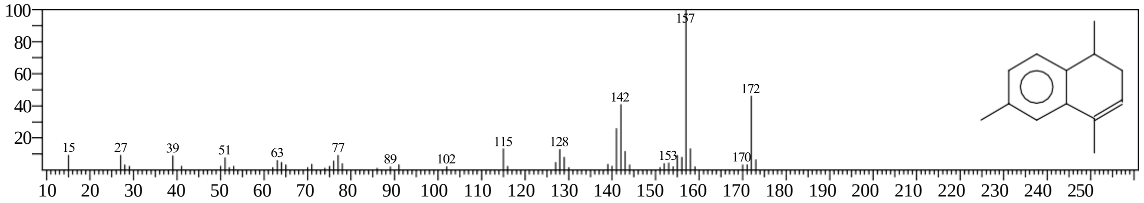

Hit#:5 Entry:22913 Library:NIST107.LIB
SI:73 Formula:C13H16 CAS:24578-28-7 MolWeight:172 RetIndex:0
CompName:Benzene, 1-methyl-4-[(1-methylethylidene)cyclopropyl]- $$ Toluene, p-(isopropylidenecyclopropyl)- $$ Benzene, 1-methyl-3-[(1-methylethylid

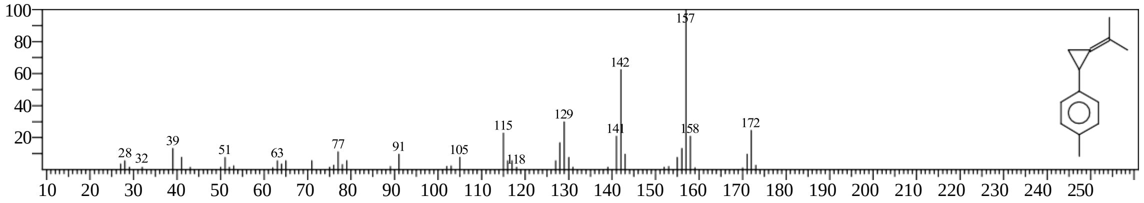

<< Target >>
Line#:91 R.Time:15.910(Scan#:3183) MassPeaks:72
RawMode:Averaged 15.905-15.915(3182-3184) BasePeak:157.25(54286)
BG Mode:Calc. from Peak

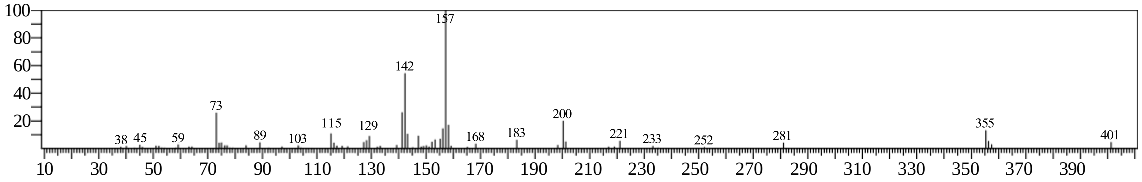

Hit#:1 Entry:35527 Library:NIST107.LIB
SI:72 Formula:C15H22 CAS:0-00-0 MolWeight:202 RetIndex:0
CompName:Cadala-1(10),3,8-triene $$

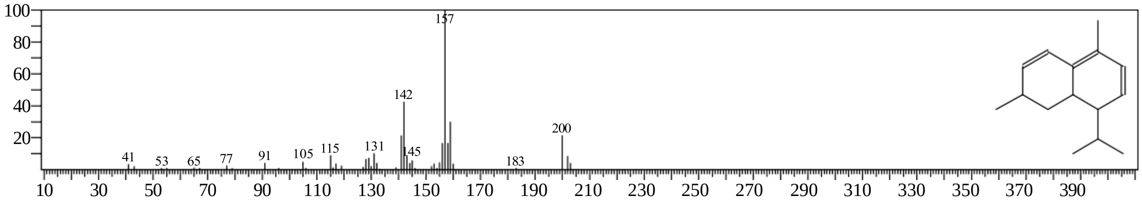

Hit#:2 Entry:49361 Library:NIST107.LIB
SI:71 Formula:C12H13Br CAS:0-00-0 MolWeight:236 RetIndex:0
CompName:1H-Indene, 3-(bromomethyl)-1,1-dimethyl- $$

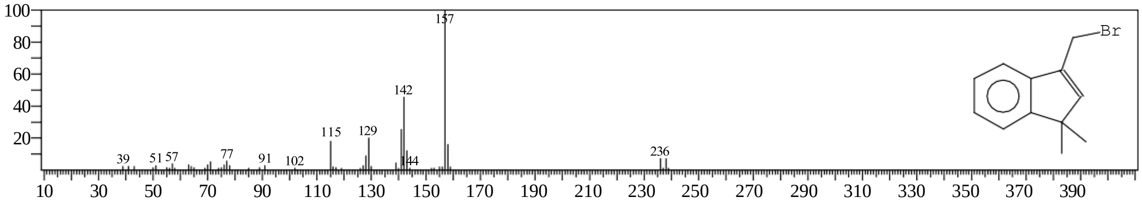

Hit#:3 Entry:22890 Library:NIST107.LIB
SI:69 Formula:C13H16 CAS:4506-36-9 MolWeight:172 RetIndex:0
CompName:Naphthalene, 1,2-dihydro-1,5,8-trimethyl- $$

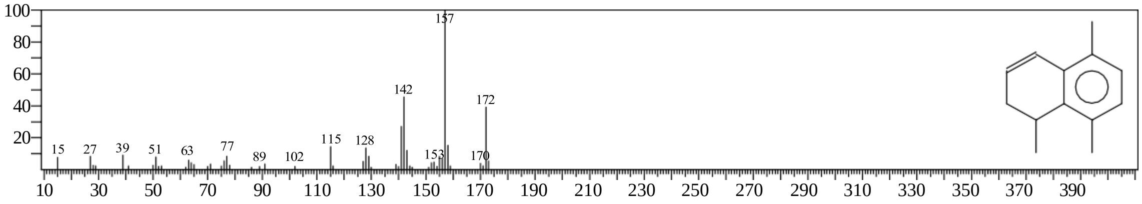

Hit#:4 Entry:22913 Library:NIST107.LIB
SI:68 Formula:C13H16 CAS:24578-28-7 MolWeight:172 RetIndex:0
CompName:Benzene, 1-methyl-4-[(1-methylethylidene)cyclopropyl]- $$ Toluene, p-(isopropylidenecyclopropyl)- $$ Benzene, 1-methyl-3-[(1-methylethylid

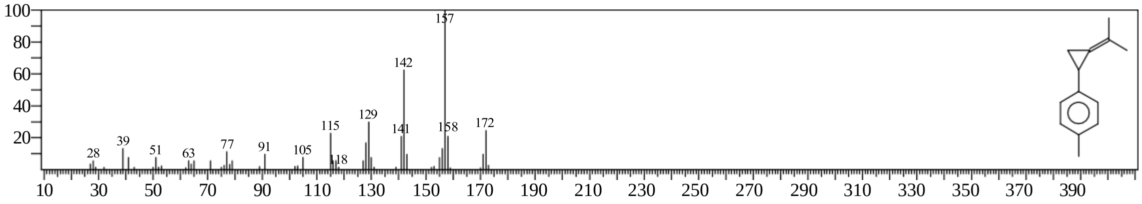

Hit#:5 Entry:22915 Library:NIST107.LIB
SI:68 Formula:C13H16 CAS:55682-80-9 MolWeight:172 RetIndex:0
CompName:Naphthalene, 1,2-dihydro-1,4,6-trimethyl- $$

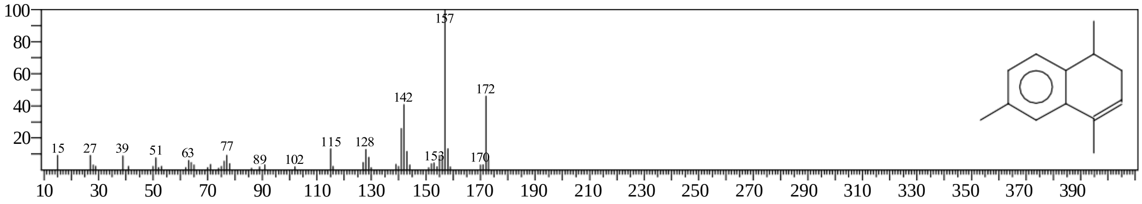

<< Target >>
Line#:92 R.Time:16.015(Scan#:3204) MassPeaks:83
RawMode:Averaged 16.010-16.020(3203-3205) BasePeak:183.25(15262)
BG Mode:Calc. from Peak

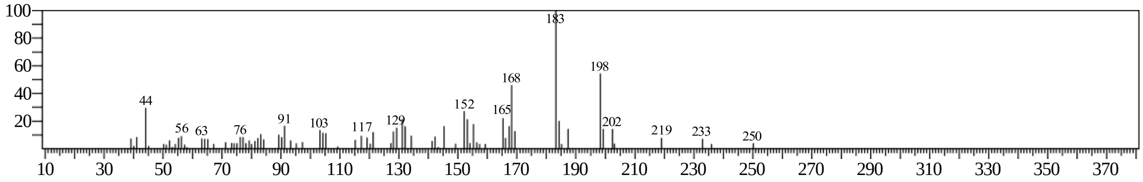

Hit#:1 Entry:34084 Library:NIST107.LIB
SI:66 Formula:C15H18 CAS:489-84-9 MolWeight:198 RetIndex:0
CompName:Azulene, 1,4-dimethyl-7-(1-methylethyl)- $$ Azulene, 7-isopropyl-1,4-dimethyl- $$ Azulen-Beris $$ Azulol $$ Azulon $$ Azunol $$ Cuteazul $

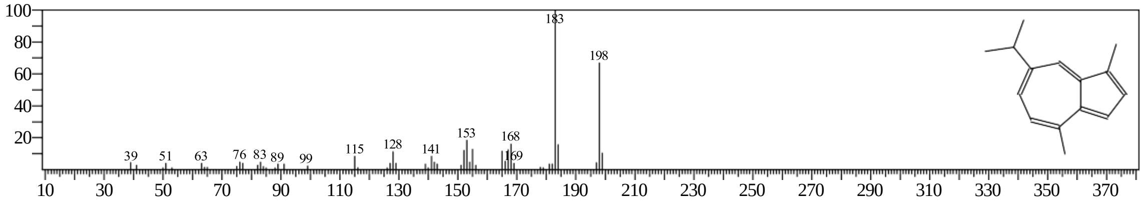

Hit#:2 Entry:34086 Library:NIST107.LIB
SI:66 Formula:C15H18 CAS:483-78-3 MolWeight:198 RetIndex:0
CompName:Naphthalene, 1,6-dimethyl-4-(1-methylethyl)- $$ Naphthalene, 4-isopropyl-1,6-dimethyl- $$ Cadalene $$ Cadalin $$ 4-Isopropyl-1,6-dimethyln

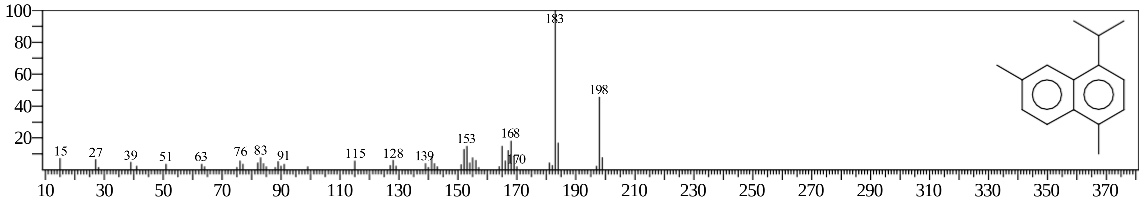

Hit#:3 Entry:34045 Library:NIST107.LIB
SI:63 Formula:C14H14O CAS:54852-74-3 MolWeight:198 RetIndex:0
CompName:Benzene, ethylphenoxy- $$

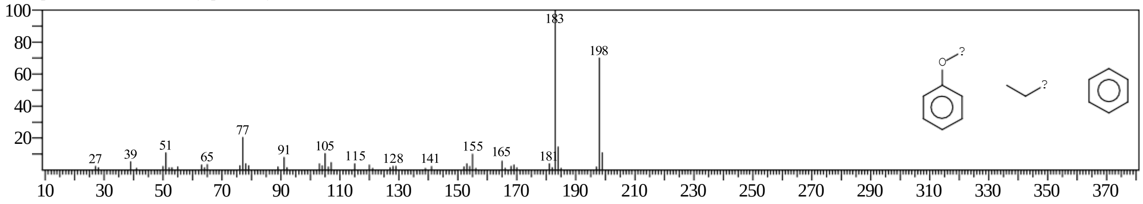

Hit#:4 Entry:34024 Library:NIST107.LIB
SI:62 Formula:C14H14O CAS:0-00-0 MolWeight:198 RetIndex:0
CompName:2-Methoxy-diphenylmethane $$

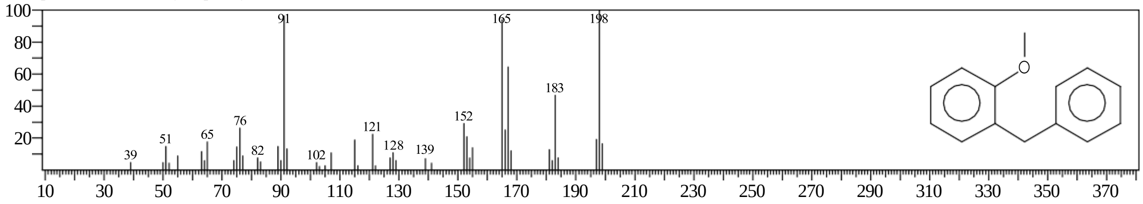

Hit#:5 Entry:88435 Library:NIST107.LIB
SI:62 Formula:C25H25NO2 CAS:0-00-0 MolWeight:371 RetIndex:0
CompName:15-Azaheptacyclo[10.5.1.0(1,13).0(2,11).0(5,10).0(7,9).0(4,8)]octadec-2(11)-ene-14,16-dione, 3,3-dimethyl-15-phenyl- $$

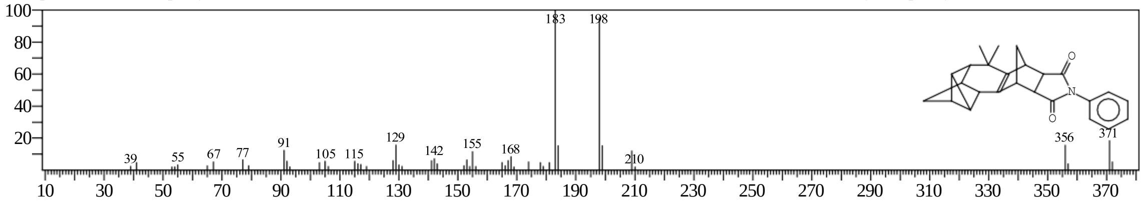

<< Target >>
Line#:93 R.Time:16.170(Scan#:3235) MassPeaks:78
RawMode:Averaged 16.165-16.175(3234-3236) BasePeak:55.10(10367)
BG Mode:Calc. from Peak

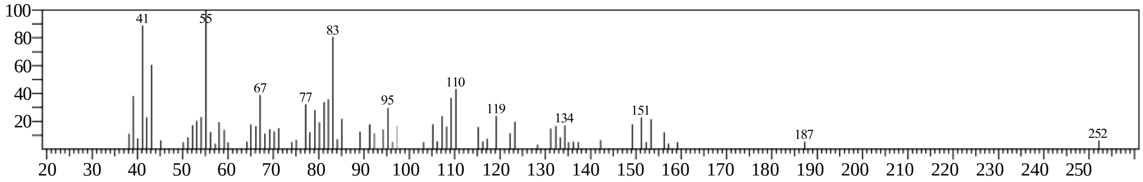

Hit#:1 Entry:19866 Library:NIST107.LIB
SI:75 Formula:C10H14O2 CAS:5951-57-5 MolWeight:166 RetIndex:0
CompName:Cyclopentaneacetaldehyde, 2-formyl-3-methyl-.alpha.-methylene- $$

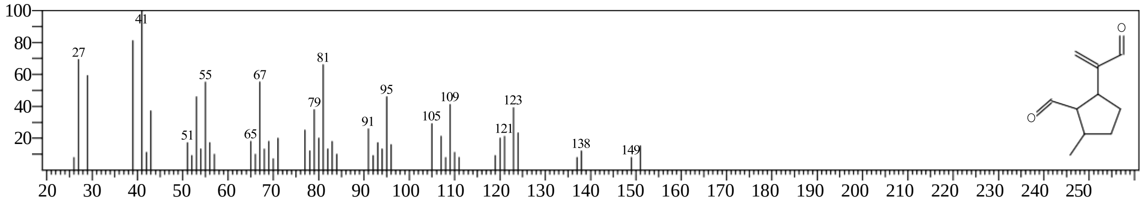

Hit#:2 Entry:14185 Library:NIST107.LIB
SI:75 Formula:C10H16O CAS:1753-35-1 MolWeight:152 RetIndex:0
CompName:(E)-3(10)-Caren-4-ol $$

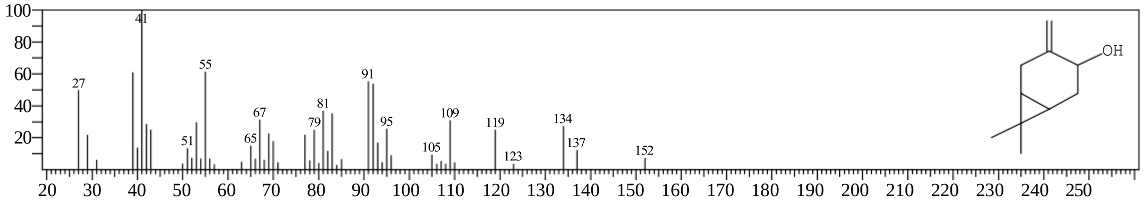

Hit#:3 Entry:14212 Library:NIST107.LIB
SI:74 Formula:C10H16O CAS:547-61-5 MolWeight:152 RetIndex:0
CompName:Bicyclo[3.1.1]heptan-3-ol, 6,6-dimethyl-2-methylene-, [1S-(1.alpha.,3.alpha.,5.alpha.)]- $$ 2(10)-Pinen-3-ol, (1S,3R,5S)-(-)- $$ L-trans-Pinocar

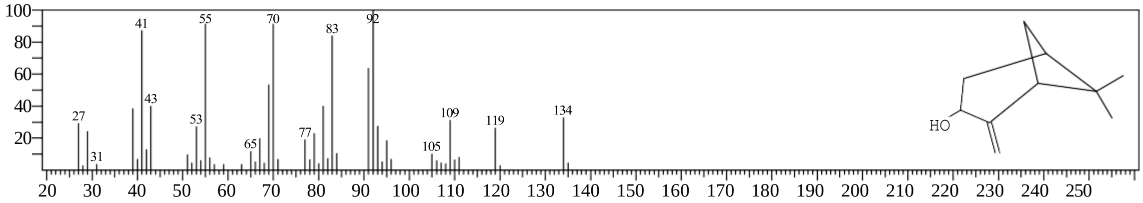

Hit#:4 Entry:21854 Library:NIST107.LIB
SI:74 Formula:C10H18O2 CAS:10359-41-8 MolWeight:170 RetIndex:0
CompName:Bicyclo[2.2.1]heptane-2,5-diol, 1,7,7-trimethyl-, (2-endo,5-exo)- $$

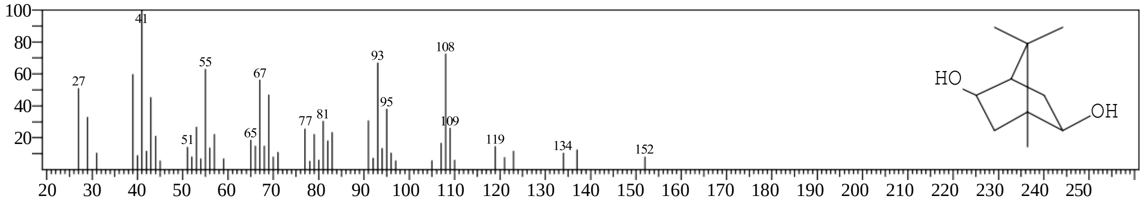

Hit#:5 Entry:14341 Library:NIST107.LIB
SI:74 Formula:C10H16O CAS:5947-36-4 MolWeight:152 RetIndex:0
CompName:Bicyclo[3.1.1]heptan-3-ol, 6,6-dimethyl-2-methylene- $$ Pinocarveol $$ 2(10)-Pinen-3-ol $$

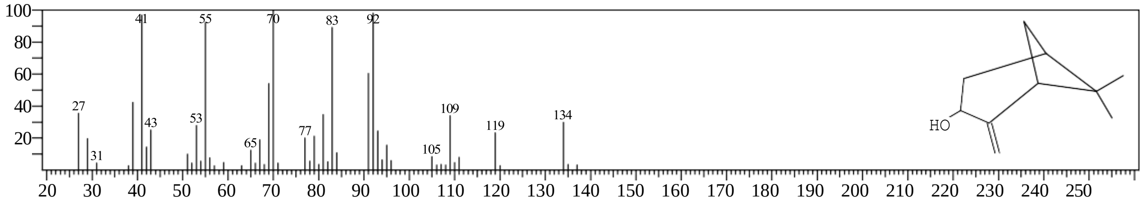

<< Target >>
Line#:94 R.Time:16.370(Scan#:3275) MassPeaks:70
RawMode:Averaged 16.365-16.375(3274-3276) BasePeak:55.15(9694)
BG Mode:Calc. from Peak

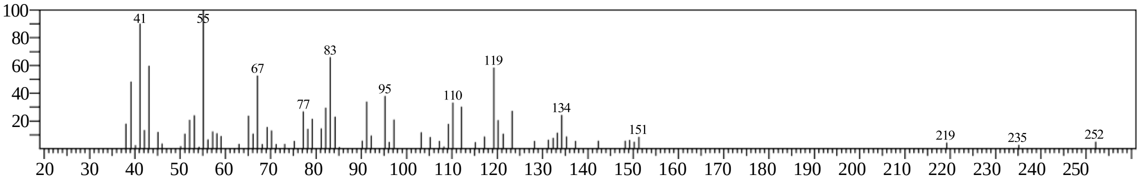

Hit#:1 Entry:14341 Library:NIST107.LIB
SI:74 Formula:C10H16O CAS:5947-36-4 MolWeight:152 RetIndex:0
CompName:Bicyclo[3.1.1]heptan-3-ol, 6,6-dimethyl-2-methylene- $$ Pinocarveol $$ 2(10)-Pinen-3-ol $$

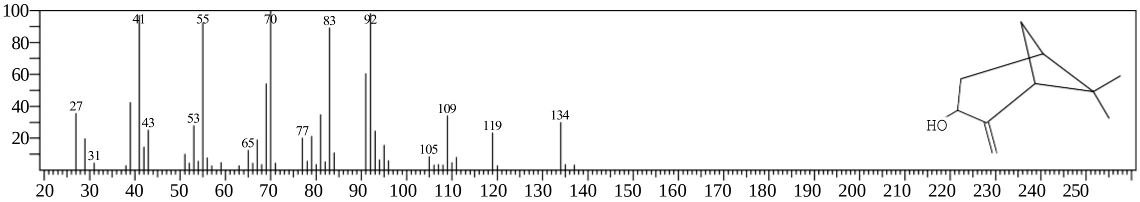

Hit#:2 Entry:14212 Library:NIST107.LIB
SI:74 Formula:C10H16O CAS:547-61-5 MolWeight:152 RetIndex:0
CompName:Bicyclo[3.1.1]heptan-3-ol, 6,6-dimethyl-2-methylene-, [1S-(1.alpha.,3.alpha.,5.alpha.)]- $$ 2(10)-Pinen-3-ol, (1S,3R,5S)-(-)- $$ L-trans-Pinocar

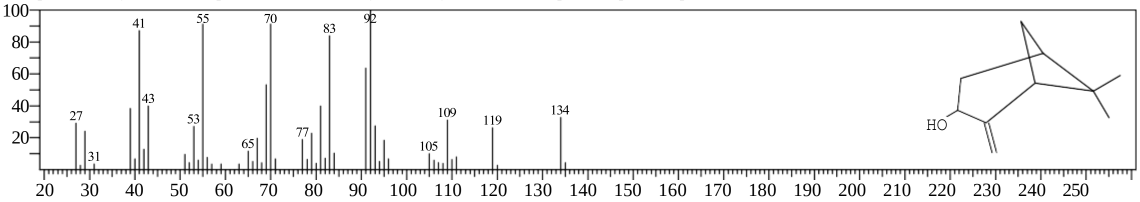

Hit#:3 Entry:14232 Library:NIST107.LIB
SI:73 Formula:C10H16O CAS:473-67-6 MolWeight:152 RetIndex:0
CompName:Bicyclo[3.1.1]hept-3-en-2-ol, 4,6,6-trimethyl- $$ d-Verbenol $$ Berbenol $$ 2-Pinen-4-ol $$ Verbenol $$ (+)-Verbenol $$

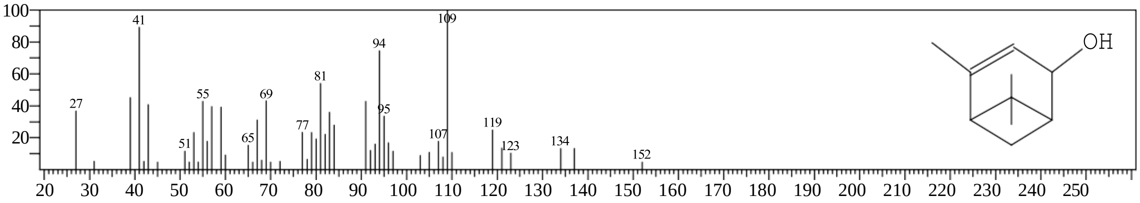

Hit#:4 Entry:14185 Library:NIST107.LIB
SI:73 Formula:C10H16O CAS:1753-35-1 MolWeight:152 RetIndex:0
CompName:(E)-3(10)-Caren-4-ol $$

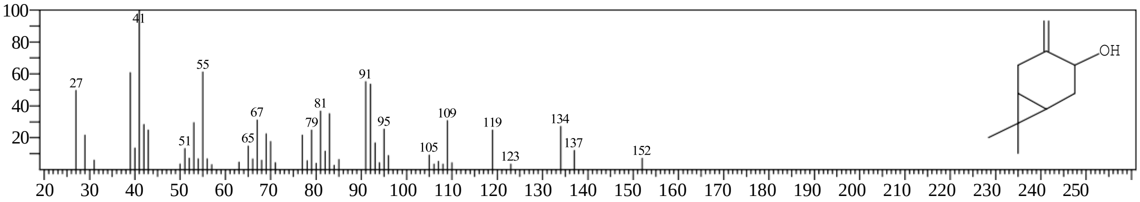

Hit#:5 Entry:42044 Library:NIST107.LIB
SI:73 Formula:C10H19Br CAS:61141-75-1 MolWeight:218 RetIndex:0
CompName:4-Decene, 5-bromo- $$

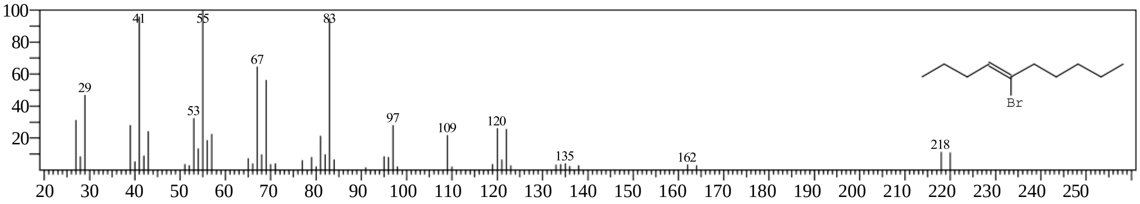

<< Target >>
Line#:95 R.Time:16.470(Scan#:3295) MassPeaks:78
RawMode:Averaged 16.465-16.475(3294-3296) BasePeak:41.15(6499)
BG Mode:Calc. from Peak

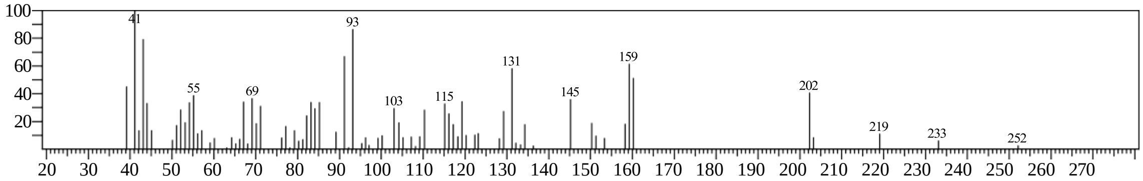

Hit#:1 Entry:36635 Library:NIST107.LIB
SI:63 Formula:C11H11NO3 CAS:15050-03-0 MolWeight:205 RetIndex:0
CompName:1H-Indole-2-carboxylic acid, 6-hydroxy-, ethyl ester $$ Indole-2-carboxylic acid, 6-hydroxy-, ethyl ester $$ Ethyl 6-hydroxyindole-2-carboxylat

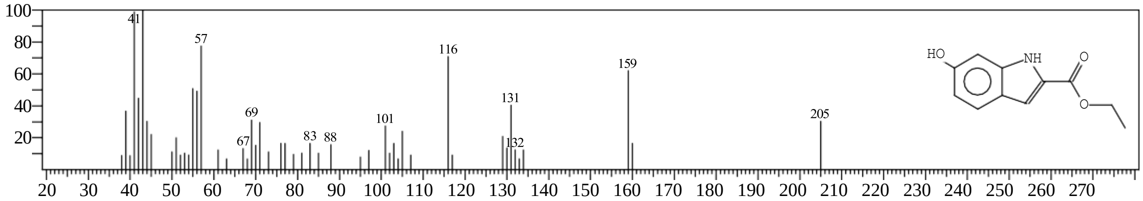

Hit#:2 Entry:52726 Library:NIST107.LIB
SI:63 Formula:C17H24O CAS:81203-57-8 MolWeight:244 RetIndex:0
CompName:Falcarinol (Z)-(-)-1,9-heptadecadiene-4,6-diyne-3-ol $$

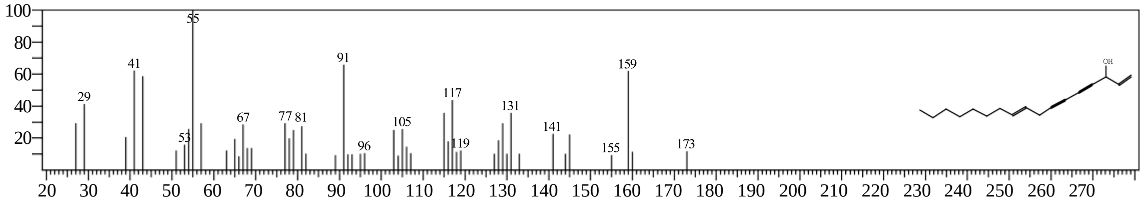

Hit#:3 Entry:79353 Library:NIST107.LIB
SI:63 Formula:C22H32O2 CAS:60534-16-9 MolWeight:328 RetIndex:0
CompName:Spiro[androst-5-ene-17,1'-cyclobutan]-2'-one, 3-hydroxy-, (3.beta.,17.beta.)- $$

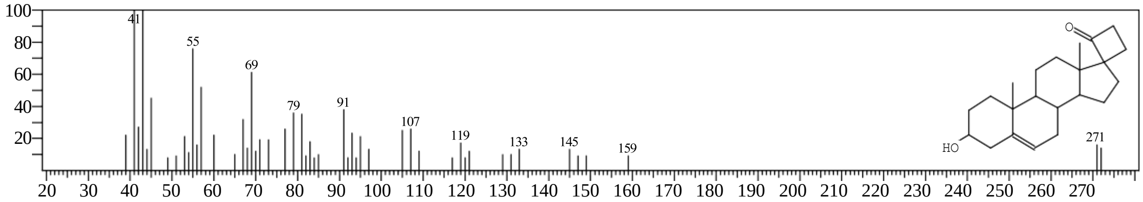

Hit#:4 Entry:52721 Library:NIST107.LIB
SI:62 Formula:C17H24O CAS:50816-77-8 MolWeight:244 RetIndex:0
CompName:2,9-Heptadecadiene-4,6-diyn-8-ol, (Z,E)- $$

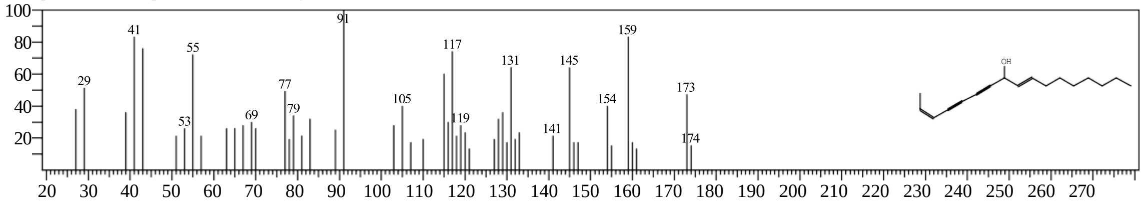

Hit#:5 Entry:43210 Library:NIST107.LIB
SI:62 Formula:C15H24O CAS:0-00-0 MolWeight:220 RetIndex:0
CompName:7-Tetracyclo[6.2.1.0(3.8)0(3.9)]undecanol, 4,4,11,11-tetramethyl- $$

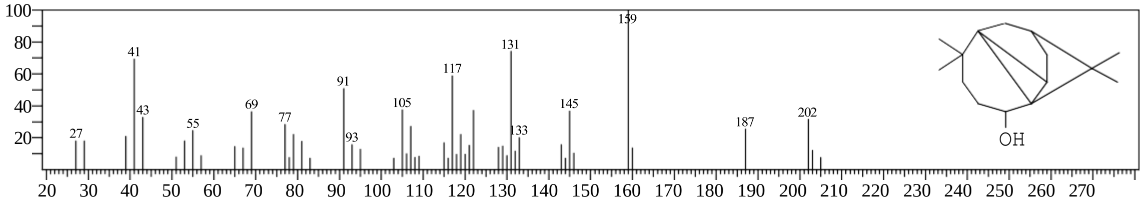

<< Target >>
Line#:96 R.Time:16.655(Scan#:3332) MassPeaks:60
RawMode:Averaged 16.650-16.660(3331-3333) BasePeak:43.10(2447)
BG Mode:Calc. from Peak

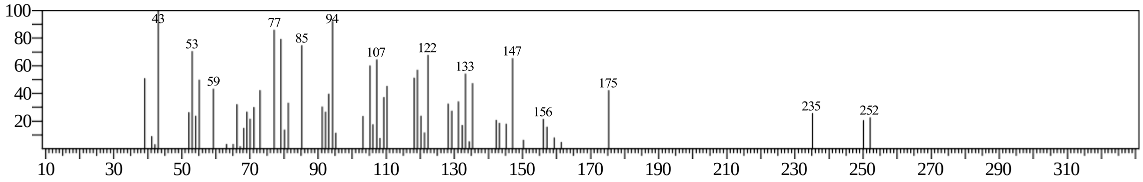

Hit#:1 Entry:102771 Library:NIST107.LIB
SI:62 Formula:C30H52O6 CAS:56053-00-0 MolWeight:508 RetIndex:0
CompName:Ergostane-3,5,6,12,25-pentol, 25-acetate, (3.beta.,5.alpha.,6.beta.,12.beta.)- $$

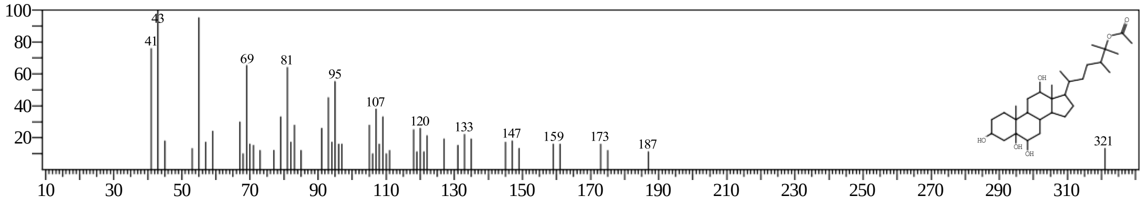

Hit#:2 Entry:42350 Library:NIST107.LIB
SI:61 Formula:C15H22O CAS:0-00-0 MolWeight:218 RetIndex:0
CompName:4,6,6-Trimethyl-2-(3-methylbuta-1,3-dienyl)-3-oxatricyclo[5.1.0.0(2,4)]octane $$

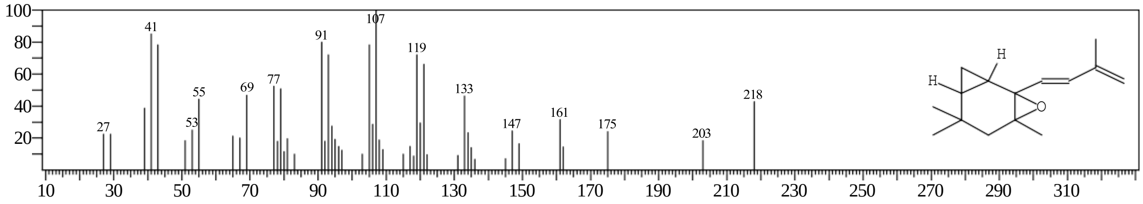

Hit#:3 Entry:76890 Library:NIST107.LIB
SI:60 Formula:C21H34O2 CAS:57344-99-7 MolWeight:318 RetIndex:0
CompName:Androstan-17-one, 3-ethyl-3-hydroxy-, (5.alpha.)- $$

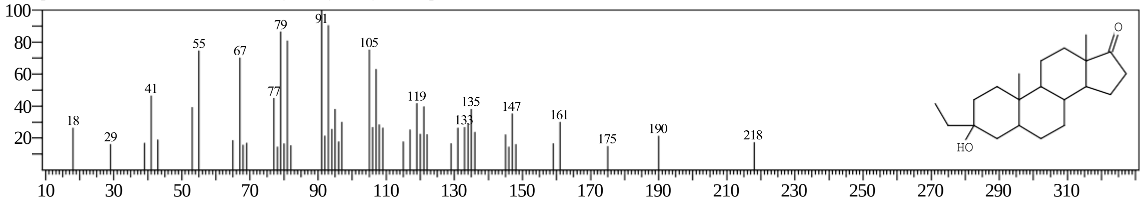

Hit#:4 Entry:64029 Library:NIST107.LIB
SI:60 Formula:C16H20O4 CAS:19892-19-4 MolWeight:276 RetIndex:0
CompName:5-Benzofuranacetic acid, 6-ethenyl-2,4,5,6,7,7a-hexahydro-3,6-dimethyl-.alpha.-methylene-2-oxo-, methyl ester $$ 5-Benzofuranacetic acid, 2,4

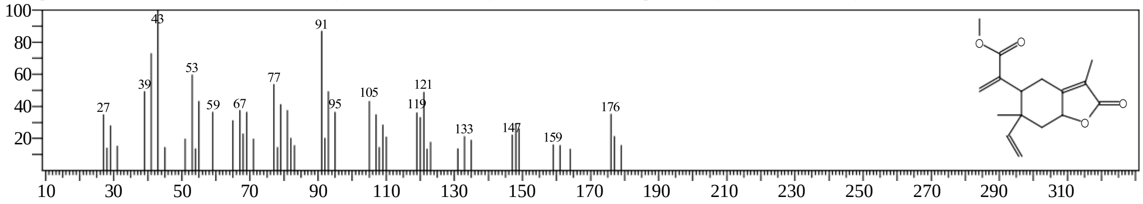

Hit#:5 Entry:24417 Library:NIST107.LIB
SI:60 Formula:C13H20 CAS:34528-95-5 MolWeight:176 RetIndex:0
CompName:Cyclohexane, 1,2-diethenyl-4-(1-methylethylidene)-, cis- $$

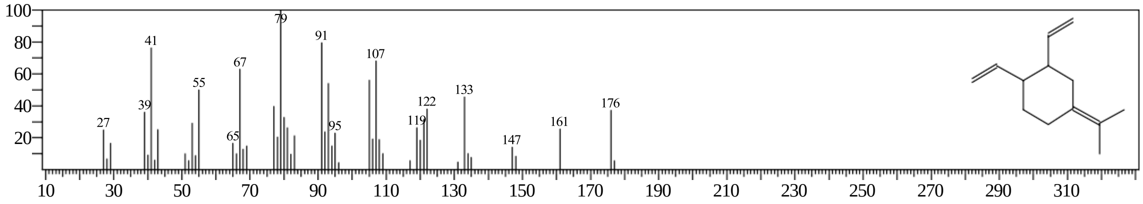

<< Target >>
Line#:97 R.Time:16.780(Scan#:3357) MassPeaks:71
RawMode:Averaged 16.775-16.785(3356-3358) BasePeak:43.10(12476)
BG Mode:Calc. from Peak

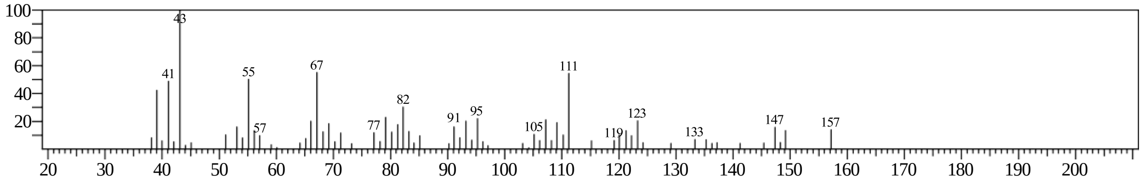

Hit#:1 Entry:44150 Library:NIST107.LIB
SI:81 Formula:C15H26O CAS:552-02-3 MolWeight:222 RetIndex:0
CompName:1H-Cycloprop[e]azulen-4-ol, decahydro-1,1,4,7-tetramethyl-, [1ar-(1a.alpha.,4.beta.,4a.beta.,7.alpha.,7a.beta.,7b.alpha.)]- $$

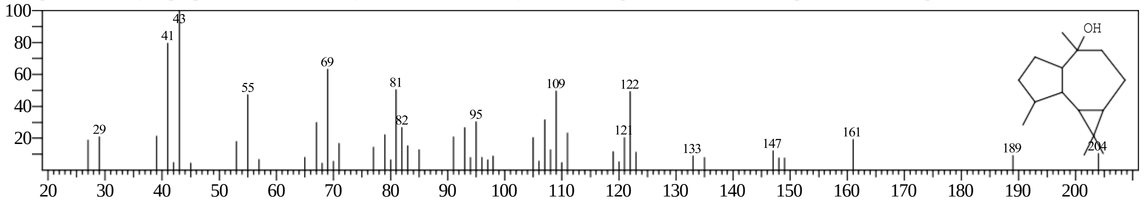

Hit#:2 Entry:43153 Library:NIST107.LIB
SI:81 Formula:C15H24O CAS:0-00-0 MolWeight:220 RetIndex:0
CompName:trans-Z-.alpha.-Bisabolene epoxide $$

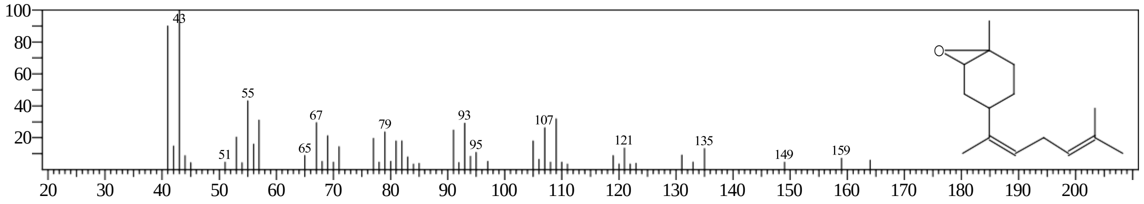

Hit#:3 Entry:32110 Library:NIST107.LIB
SI:80 Formula:C13H22O CAS:0-00-0 MolWeight:194 RetIndex:0
CompName:Cyclododeca-5,9-dien-1-ol, 2-methyl-, (Z,Z)- $$

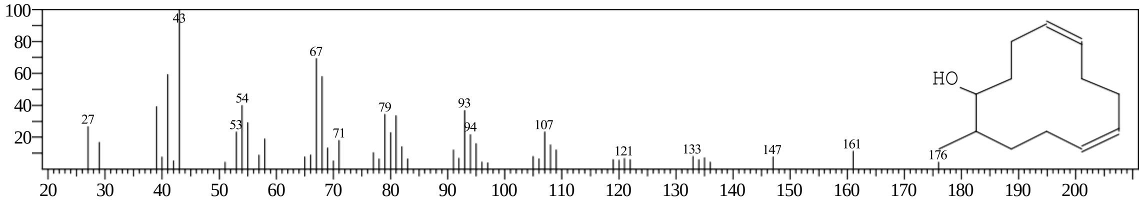

Hit#:4 Entry:45106 Library:NIST107.LIB
SI:79 Formula:C15H28O CAS:2834-00-6 MolWeight:224 RetIndex:0
CompName:2-Pentadecyn-1-ol $$

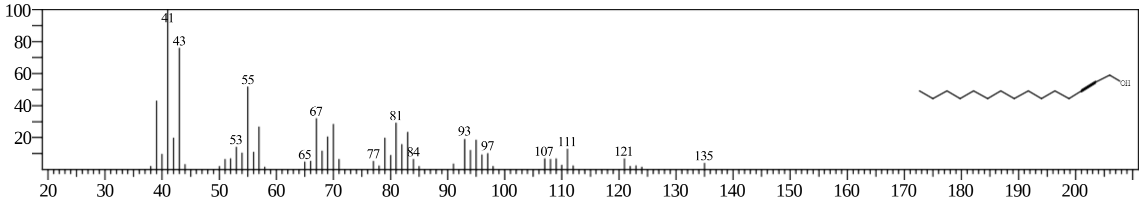

Hit#:5 Entry:20826 Library:NIST107.LIB
SI:79 Formula:C10H16O2 CAS:96-08-2 MolWeight:168 RetIndex:0
CompName:7-Oxabicyclo[4.1.0]heptane, 1-methyl-4-(2-methyloxiranyl)- $$ p-Menthane, 1,2:8,9-diepoxy- $$ .alpha.-Limonene diepoxide $$ Dipentene die

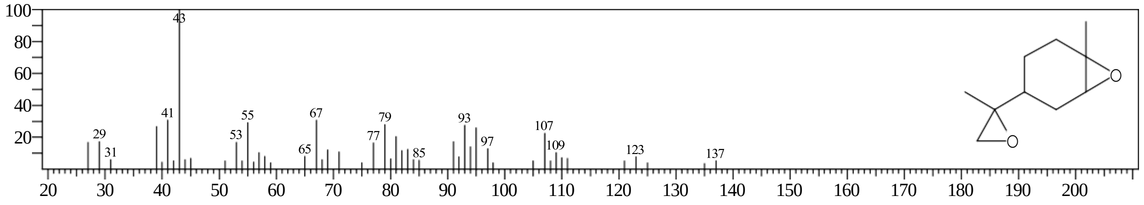

<< Target >>
Line#:98 R.Time:18.465(Scan#:3694) MassPeaks:83
RawMode:Averaged 18.460-18.470(3693-3695) BasePeak:243.30(30662)
BG Mode:Calc. from Peak

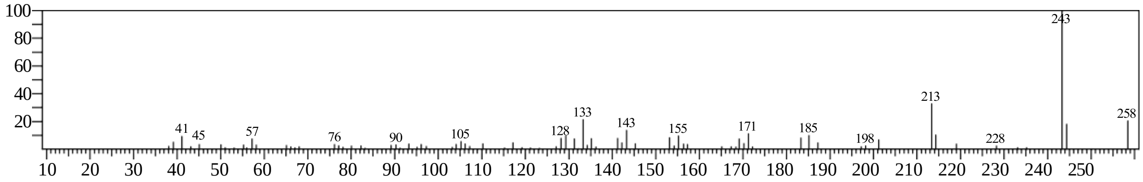

Hit#:1 Entry:57840 Library:NIST107.LIB
SI:72 Formula:C18H26O CAS:1222-05-5 MolWeight:258 RetIndex:0
CompName:Cyclopenta[g]-2-benzopyran, 1,3,4,6,7,8-hexahydro-4,6,6,7,8,8-hexamethyl- $$ Galoxolide $$ 1,3,4,6,7,8-Hexahydro-4,6,6,7,8,8-hexamethyl-c

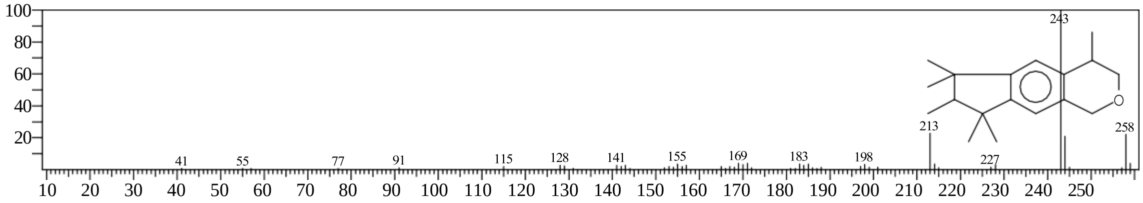

Hit#:2 Entry:57841 Library:NIST107.LIB
SI:65 Formula:C18H26O CAS:88-29-9 MolWeight:258 RetIndex:0
CompName:7-Acetyl-6-ethyl-1,1,4,4-tetramethyltetralin $$ Ethanone, 1-(3-ethyl-5,6,7,8-tetrahydro-5,5,8,8-tetramethyl-2-naphthalenyl)- $$ Musk 36A $$ Po

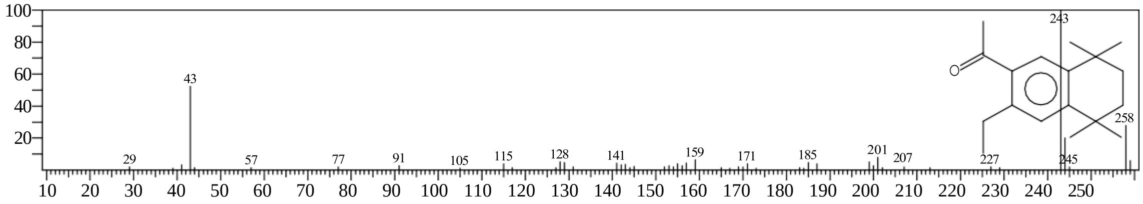

Hit#:3 Entry:99531 Library:NIST107.LIB
SI:58 Formula:C29H34N2O3 CAS:75941-90-1 MolWeight:458 RetIndex:0
CompName:Benzoic acid, 4-(4-butylcyclohexyl)-, 4-butoxy-2,3-dicyanophenyl ester $$

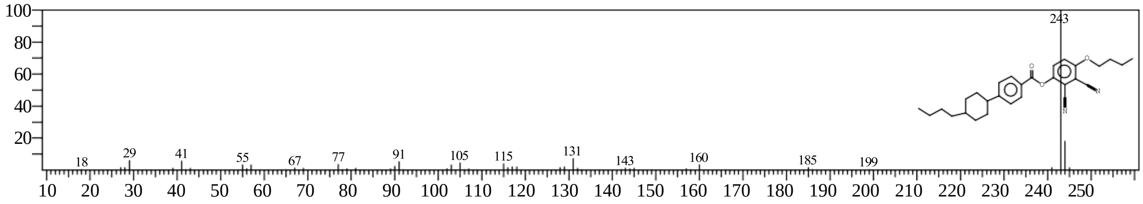

Hit#:4 Entry:100615 Library:NIST107.LIB
SI:57 Formula:C30H36N2O3 CAS:75941-91-2 MolWeight:472 RetIndex:0
CompName:Benzoic acid, 4-(4-butylcyclohexyl)-, 2,3-dicyano-4-(pentyloxy)phenyl ester $$

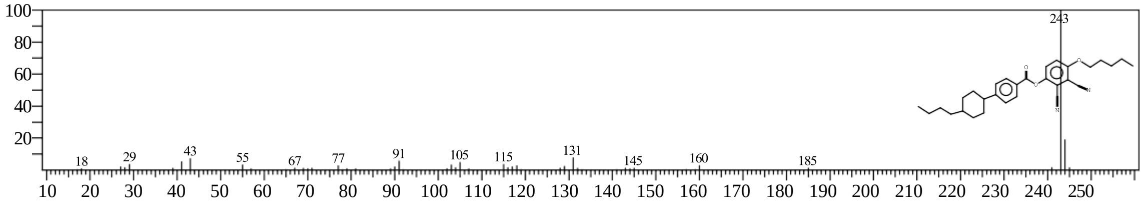

Hit#:5 Entry:57698 Library:NIST107.LIB
SI:55 Formula:C15H14O4 CAS:0-00-0 MolWeight:258 RetIndex:0
CompName:6-Acetyl-5-methoxy-2,7-dimethyl-1,4-naphthoguinone $$

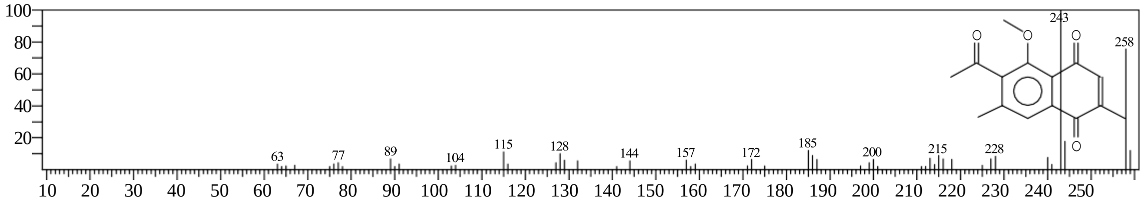

<< Target >>
Line#:99 R.Time:18.565(Scan#:3714) MassPeaks:80
RawMode:Averaged 18.560-18.570(3713-3715) BasePeak:133.25(62105)
BG Mode:Calc. from Peak

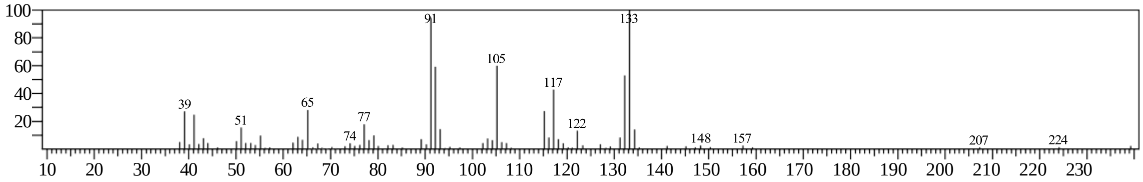

Hit#:1 Entry:12784 Library:NIST107.LIB
SI:82 Formula:C11H16 CAS:7338-49-0 MolWeight:148 RetIndex:0
CompName:1,3-Cyclopentadiene, 5-(1,3-dimethylbutylidene)- $$

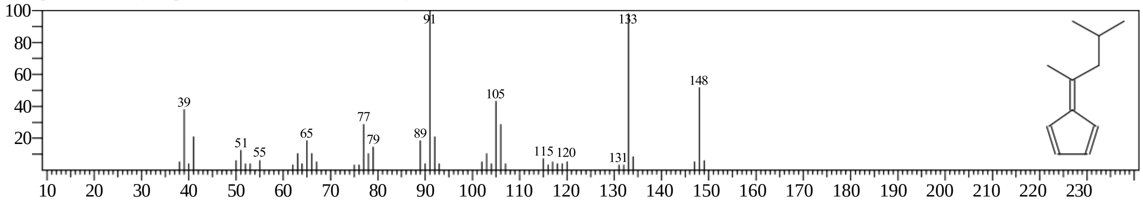

Hit#:2 Entry:7652 Library:NIST107.LIB
SI:81 Formula:C10H12 CAS:0-00-0 MolWeight:132 RetIndex:0
CompName:5,7-Dimethylenebicyclo[2.2.2]oct-2-ene $$

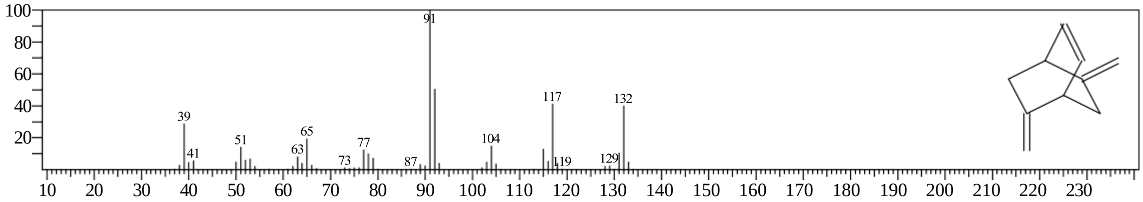

Hit#:3 Entry:18249 Library:NIST107.LIB
SI:81 Formula:C11H14O CAS:52417-50-2 MolWeight:162 RetIndex:0
CompName:Benzeneacetaldehyde, .alpha.,2,5-trimethyl- $$

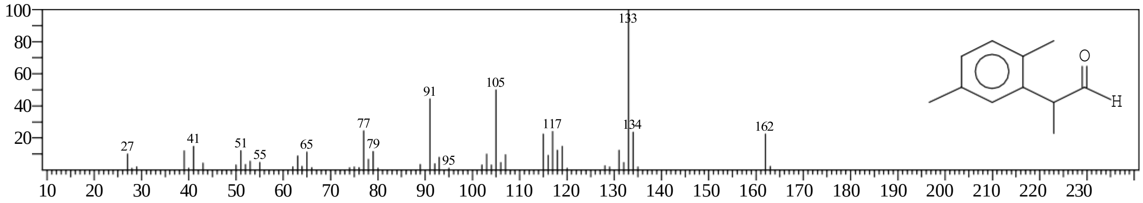

Hit#:4 Entry:7809 Library:NIST107.LIB
SI:80 Formula:C9H11N CAS:52562-19-3 MolWeight:133 RetIndex:0
CompName:Benzenamine, 2-(1-methylethenyl)- $$ o-Isopropenylaniline $$ 2-Isopropenylaniline $$

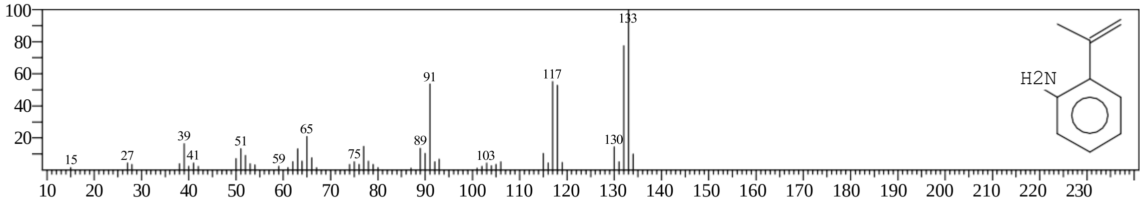

Hit#:5 Entry:7653 Library:NIST107.LIB
SI:80 Formula:C10H12 CAS:0-00-0 MolWeight:132 RetIndex:0
CompName:5,8-Dimethylenebicyclo[2.2.2]oct-2-ene $$

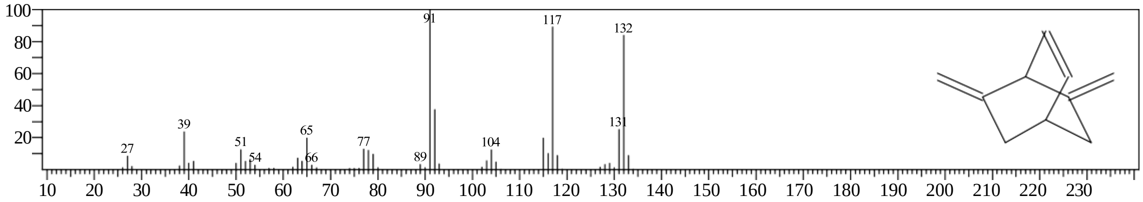

<< Target >>
Line#:100 R.Time:18.780(Scan#:3757) MassPeaks:79
RawMode:Averaged 18.775-18.785(3756-3758) BasePeak:105.20(105728)
BG Mode:Calc. from Peak

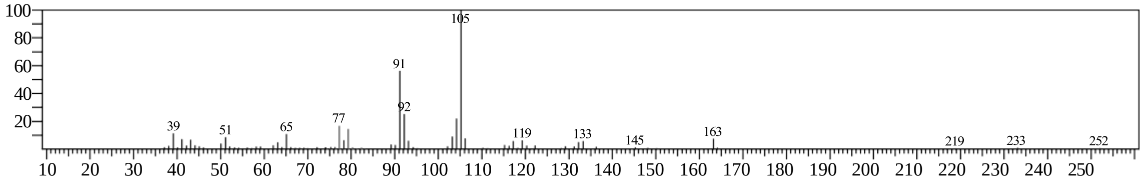

Hit#:1 Entry:62096 Library:NIST107.LIB
SI:85 Formula:C18H22O2 CAS:122-71-4 MolWeight:270 RetIndex:0
CompName:Phenylethylacetal $$ Benzene, 1,1'-[ethylidenebis(oxy-2,1-ethanediyl)]bis- $$ Acetaldehyde diphenylethylacetal $$

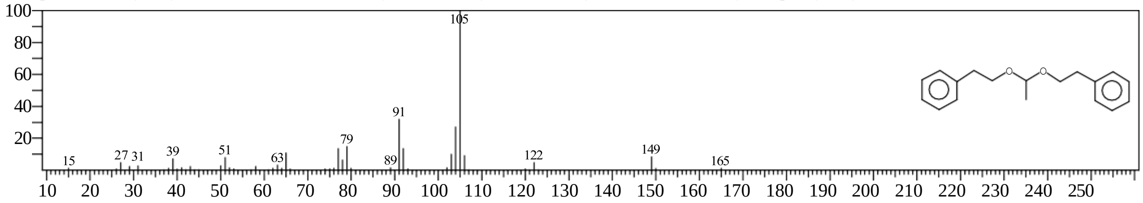

Hit#:2 Entry:39219 Library:NIST107.LIB
SI:82 Formula:C16H18 CAS:1520-44-1 MolWeight:210 RetIndex:0
CompName:Benzene, 1,1'-(1-methyl-1,3-propanediyl)bis- $$

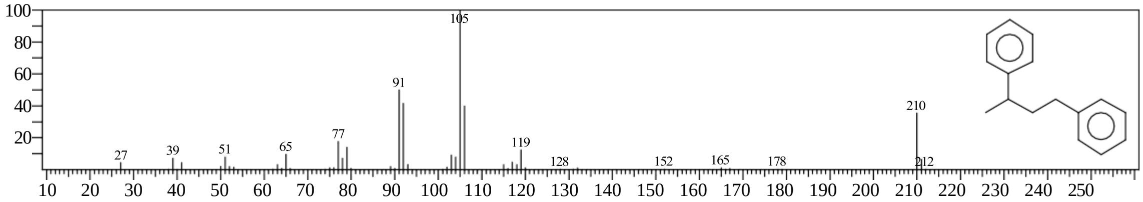

Hit#:3 Entry:12760 Library:NIST107.LIB
SI:82 Formula:C11H16 CAS:113003-13-7 MolWeight:148 RetIndex:0
CompName:Bicyclo[4.1.0]hept-3-ene, 7,7-dimethyl-3-vinyl- $$

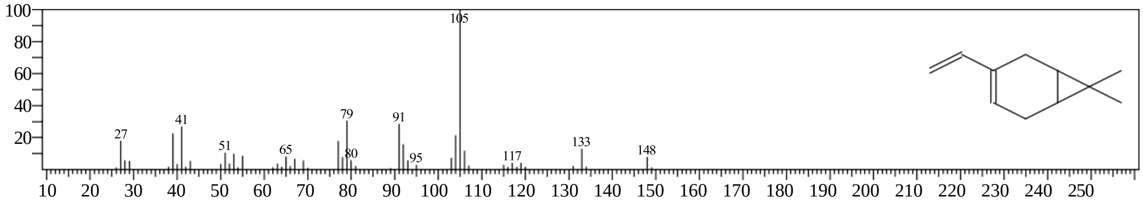

Hit#:4 Entry:45160 Library:NIST107.LIB
SI:82 Formula:C17H20 CAS:6443-80-7 MolWeight:224 RetIndex:0
CompName:Pentane, 1,4-diphenyl- $$

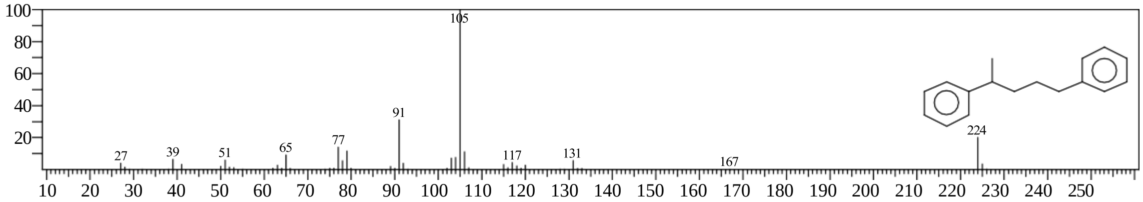

Hit#:5 Entry:4809 Library:NIST107.LIB
SI:82 Formula:C9H12 CAS:17634-51-4 MolWeight:120 RetIndex:0
CompName:1,3,5-Cycloheptatriene, 7-ethyl- $$ 7-Ethyl-1,3,5-cycloheptatriene $$ 7-Ethylcycloheptatriene $$

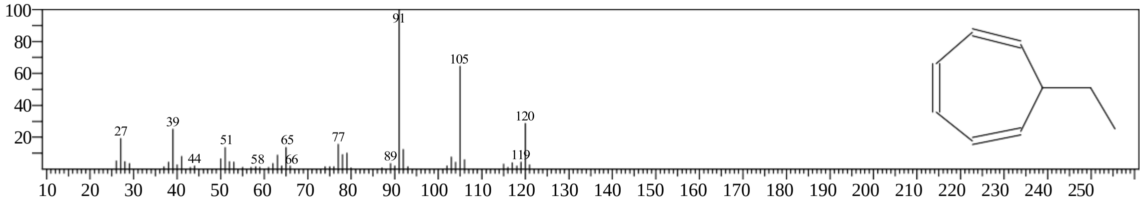

<< Target >>
Line#:101 R.Time:18.975(Scan#:3796) MassPeaks:77
RawMode:Averaged 18.970-18.980(3795-3797) BasePeak:135.25(34203)
BG Mode:Calc. from Peak

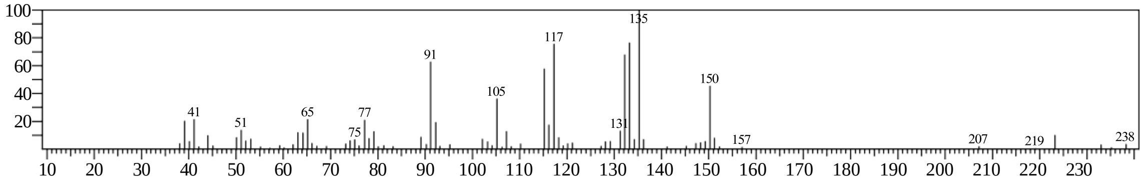

Hit#:1 Entry:13387 Library:NIST107.LIB
SI:81 Formula:C10H14O CAS:99500-87-5 MolWeight:150 RetIndex:0
CompName:1-(2,4-Dimethylphenyl)ethanol $$

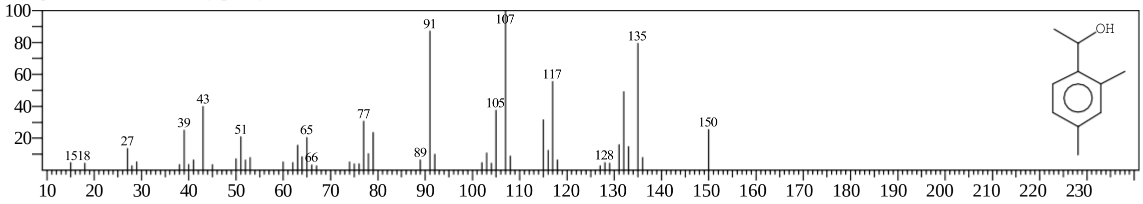

Hit#:2 Entry:13360 Library:NIST107.LIB
SI:80 Formula:C10H14O CAS:32917-52-5 MolWeight:150 RetIndex:0
CompName:2,5-Dimethylphenyl methyl carbinol $$ .alpha.,2,5-Trimethylbenzyl alcohol $$ Benzenemethanol, .alpha.,2,5-trimethyl- $$

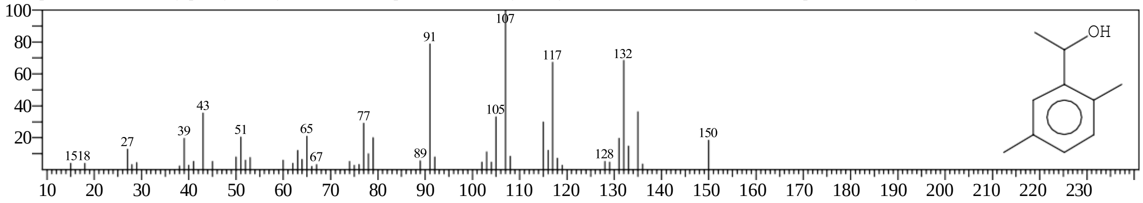

Hit#:3 Entry:13397 Library:NIST107.LIB
SI:79 Formula:C10H14O CAS:4393-05-9 MolWeight:150 RetIndex:0
CompName:Benzenemethanol, 2,4,5-trimethyl- $$ Benzyl alcohol, 2,4,5-trimethyl- $$ .alpha.-Durenol $$ Pseudocumene-5-methylol $$ 2,4,5-Trimethylbenz

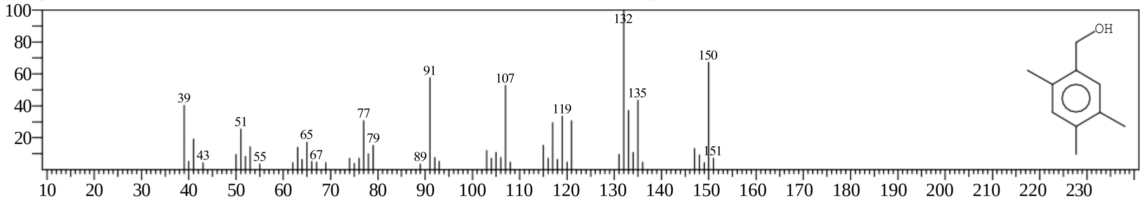

Hit#:4 Entry:7658 Library:NIST107.LIB
SI:75 Formula:C10H12 CAS:1195-32-0 MolWeight:132 RetIndex:0
CompName:Benzene, 1-methyl-4-(1-methylethenyl)- $$ Styrene, p,.alpha.-dimethyl- $$ .alpha.,p-Dimethylstyrene $$ .alpha.,4-Dimethylstyrene $$ p-Isoprop

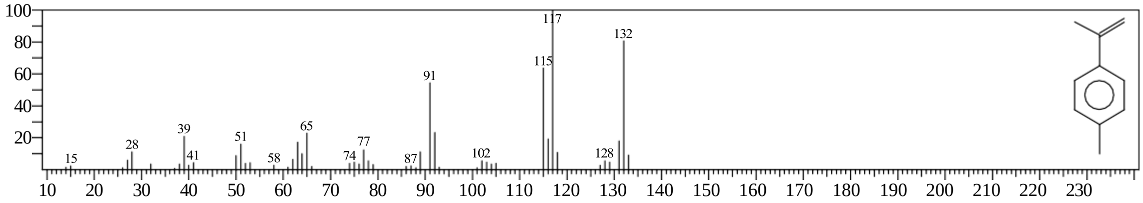

Hit#:5 Entry:7661 Library:NIST107.LIB
SI:73 Formula:C10H12 CAS:7399-49-7 MolWeight:132 RetIndex:0
CompName:o-Isopropenyltoluene $$ 1-Methyl-2-iso-propenylbenzene $$ Benzene, 1-methyl-2-(1-methylethenyl)- $$ o-Methyl-.alpha.-methylstyrene $$ o,.

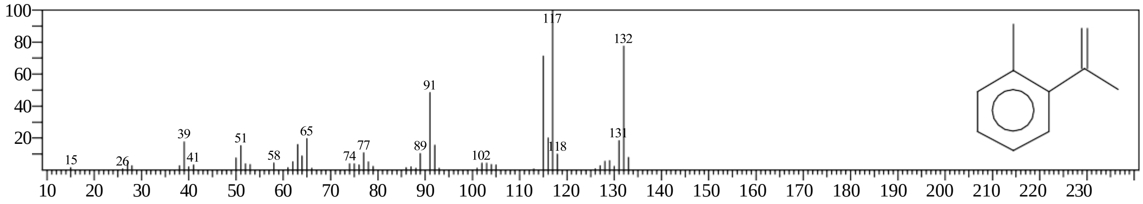

<< Target >>
Line#:102 R.Time:19.220(Scan#:3845) MassPeaks:63
RawMode:Averaged 19.215-19.225(3844-3846) BasePeak:135.25(48345)
BG Mode:Calc. from Peak

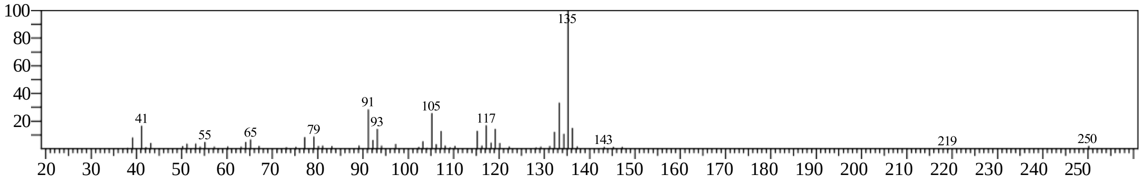

Hit#:1 Entry:13363 Library:NIST107.LIB
SI:78 Formula:C10H14O CAS:536-60-7 MolWeight:150 RetIndex:0
CompName:Benzenemethanol, 4-(1-methylethyl)- $$ p-Cymen-7-ol $$ p-Isopropylbenzyl alcohol $$ Cumic alcohol $$ Cuminic alcohol $$ Cuminol $$ Cum

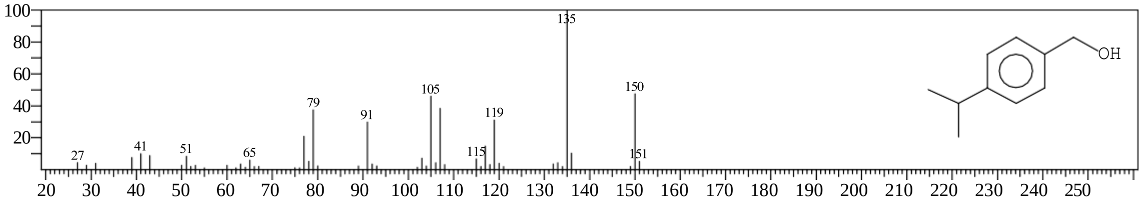

Hit#:2 Entry:52062 Library:NIST107.LIB
SI:78 Formula:C17H22O CAS:74685-43-1 MolWeight:242 RetIndex:0
CompName:Benzenemethanol, .alpha.-ethyl-.alpha.-2,5,7-octatrienyl- $$

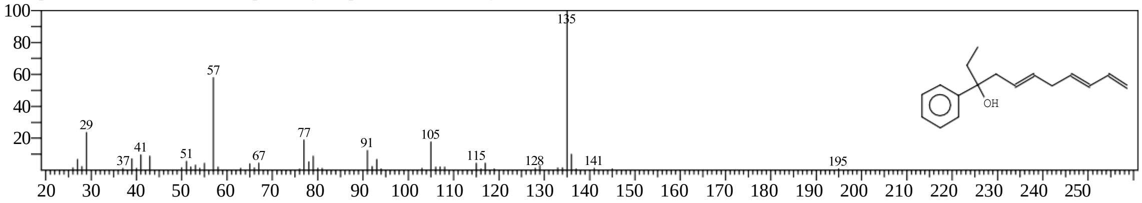

Hit#:3 Entry:26418 Library:NIST107.LIB
SI:77 Formula:C10H15NO2 CAS:7575-82-8 MolWeight:181 RetIndex:0
CompName:Tricyclo[3.3.1.13,7]decane, 1-nitro- $$ Adamantane, 1-nitro- $$ 1-Nitroadamantane $$

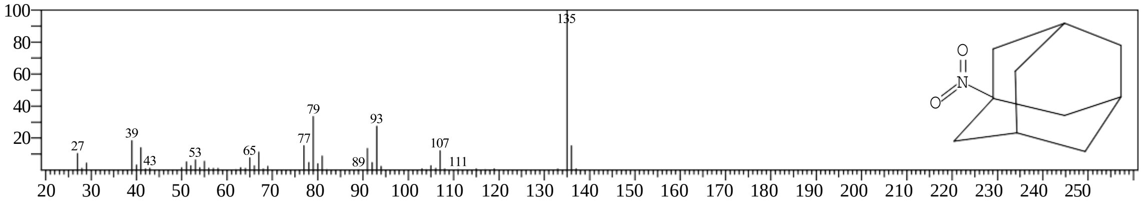

Hit#:4 Entry:32048 Library:NIST107.LIB
SI:77 Formula:C12H18O2 CAS:4942-47-6 MolWeight:194 RetIndex:0
CompName:1-Adamantaneacetic acid $$ Tricyclo[3.3.1.1(3,7)-]decane-1-acetic acid $$ 1-Adamantylacetic acid $$

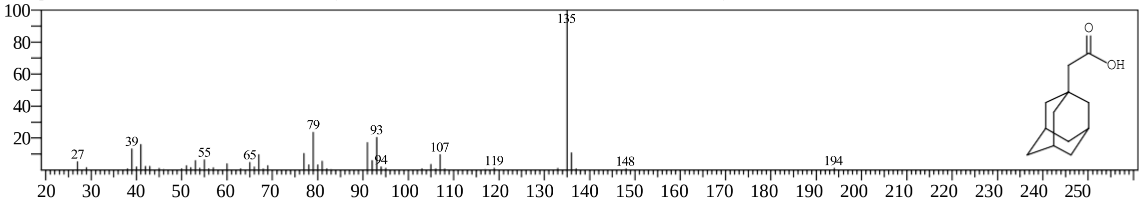

Hit#:5 Entry:13464 Library:NIST107.LIB
SI:76 Formula:C10H14O CAS:89-83-8 MolWeight:150 RetIndex:0
CompName:Thymol $$ Phenol, 5-methyl-2-(1-methylethyl)- $$ m-Thymol $$ p-Cymen-3-ol $$ Thyme camphor $$ 2-Isopropyl-5-methylphenol $$ 3-Hydro

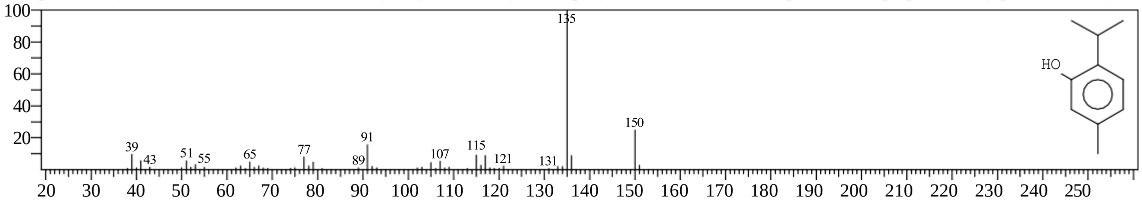

<< Target >>
Line#:103 R.Time:19.260(Scan#:3853) MassPeaks:66
RawMode:Averaged 19.255-19.265(3852-3854) BasePeak:133.25(8233)
BG Mode:Calc. from Peak

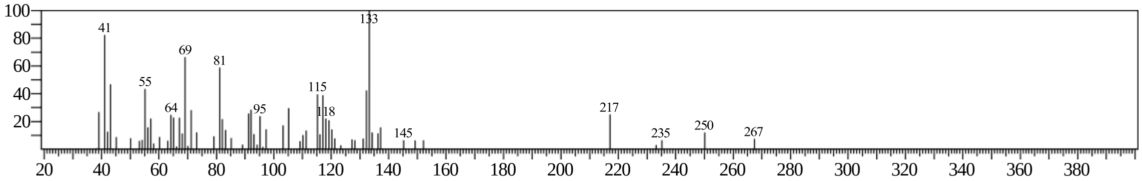

Hit#:1 Entry:79353 Library:NIST107.LIB
SI:68 Formula:C22H32O2 CAS:60534-16-9 MolWeight:328 RetIndex:0
CompName:Spiro[androst-5-ene-17,1'-cyclobutan]-2'-one, 3-hydroxy-, (3.beta.,17.beta.)- $$

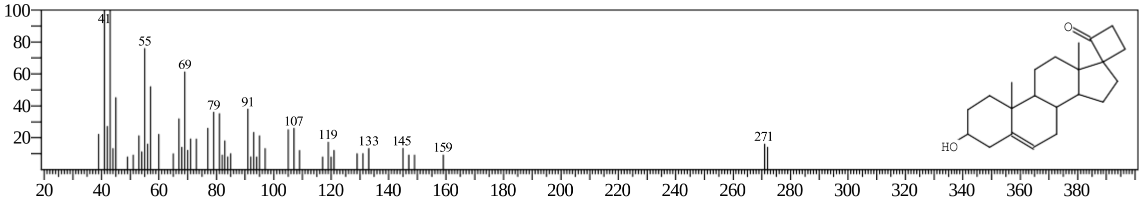

Hit#:2 Entry:92821 Library:NIST107.LIB
SI:67 Formula:C28H46O CAS:7199-92-0 MolWeight:398 RetIndex:0
CompName:Cholesta-8,24-dien-3-ol, 4-methyl-, (3.beta.,4.alpha.)- $$

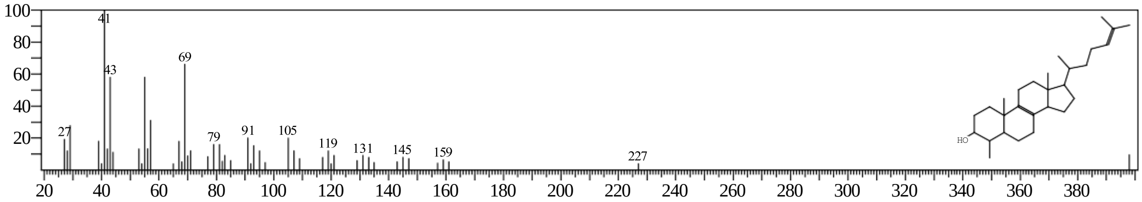

Hit#:3 Entry:43196 Library:NIST107.LIB
SI:66 Formula:C15H24O CAS:0-00-0 MolWeight:220 RetIndex:0
CompName:Longipinocarveol, trans- $$

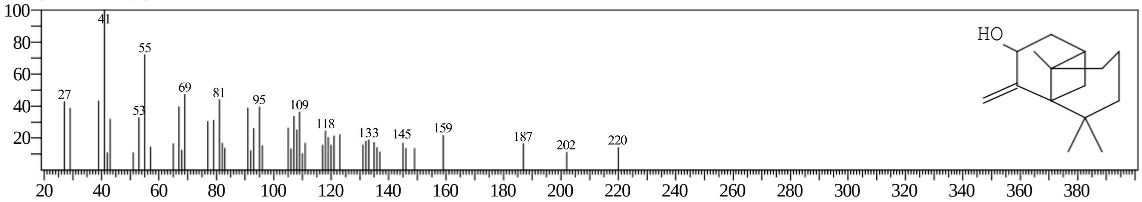

Hit#:4 Entry:68698 Library:NIST107.LIB
SI:66 Formula:C19H30O2 CAS:57156-91-9 MolWeight:290 RetIndex:0
CompName:2,5-Octadecadiynoic acid, methyl ester $$

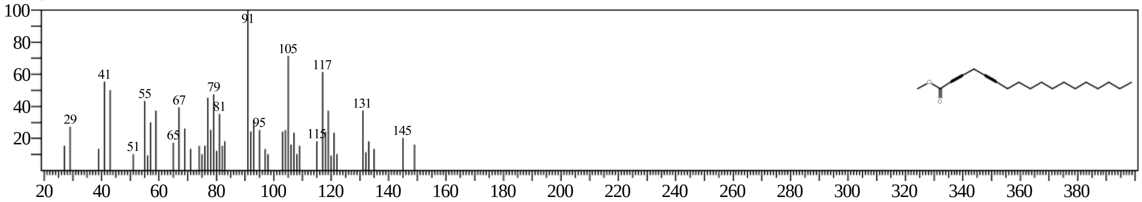

Hit#:5 Entry:96345 Library:NIST107.LIB
SI:65 Formula:C27H38O4 CAS:0-00-0 MolWeight:426 RetIndex:0
CompName:Spirost-4-ene-3,6-dione $$

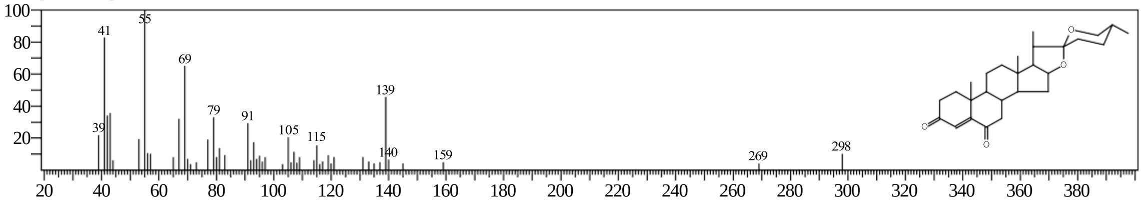

<< Target >>
Line#:104 R.Time:19.520(Scan#:3905) MassPeaks:86
RawMode:Averaged 19.515-19.525(3904-3906) BasePeak:41.15(10286)
BG Mode:Calc. from Peak

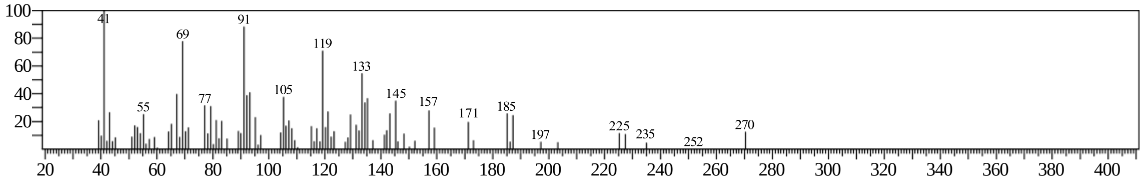

Hit#:1 Entry:62158 Library:NIST107.LIB
SI:77 Formula:C20H30 CAS:57988-82-6 MolWeight:270 RetIndex:0
CompName:Bicyclo[3.1.1]hept-2-ene, 2,2'-(1,2-ethanediyl)bis[6,6-dimethyl- $$

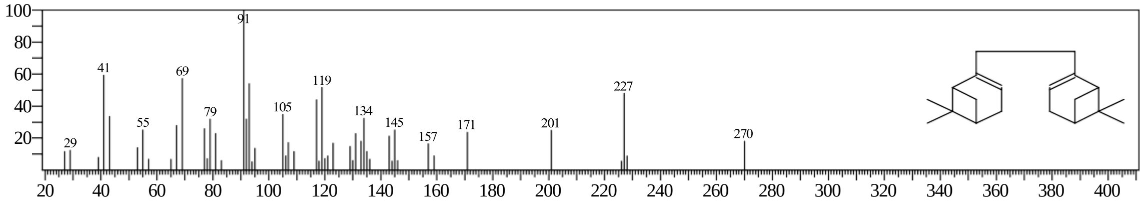

Hit#:2 Entry:49567 Library:NIST107.LIB
SI:75 Formula:C15H24O2 CAS:0-00-0 MolWeight:236 RetIndex:0
CompName:Bicyclo[4.4.0]dec-2-ene-4-ol, 2-methyl-9-(prop-1-en-3-ol-2-yl)- $$

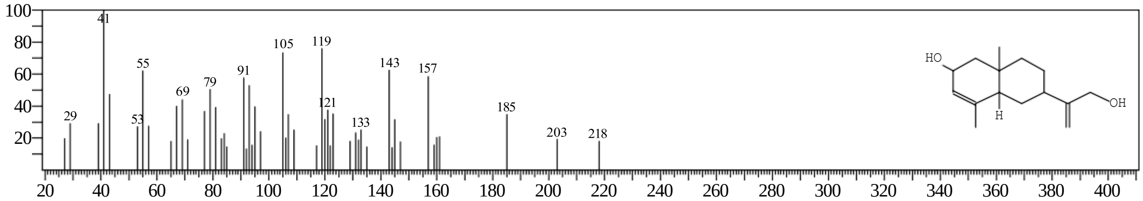

Hit#:3 Entry:79361 Library:NIST107.LIB
SI:73 Formula:C22H32O2 CAS:127-47-9 MolWeight:328 RetIndex:0
CompName:Retinol, acetate $$ Retinol, acetate, all-trans- $$ All-trans-retinol acetate $$ All-trans-retinyl acetate $$ All-trans-vitamin a acetate $$ trans-Retin

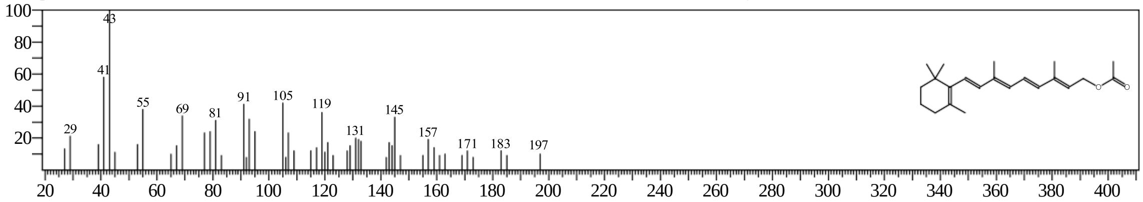

Hit#:4 Entry:86744 Library:NIST107.LIB
SI:72 Formula:C22H34O4 CAS:28393-02-4 MolWeight:362 RetIndex:0
CompName:10,12-Docasadiyndioic acid $$

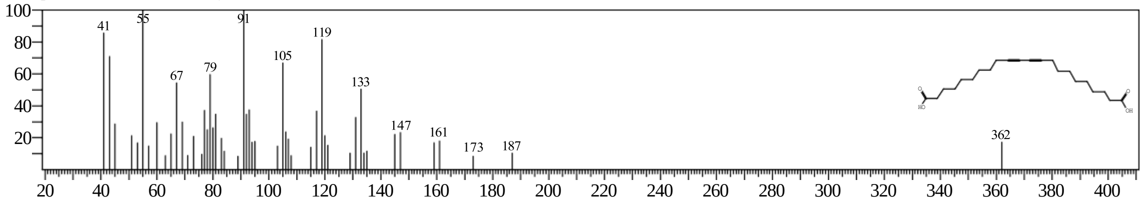

Hit#:5 Entry:93758 Library:NIST107.LIB
SI:72 Formula:C30H44 CAS:56613-34-4 MolWeight:404 RetIndex:0
CompName:Dibenzo[a,H]cyclotetradecene, 2,3,11,12-tetraethenyl-1,2,3,4,5,6,7,8,9,10,11,12,13,14,15,16,17,18-octadecahydro-, (2R@,3s@,4Z,9Z,11R@,1

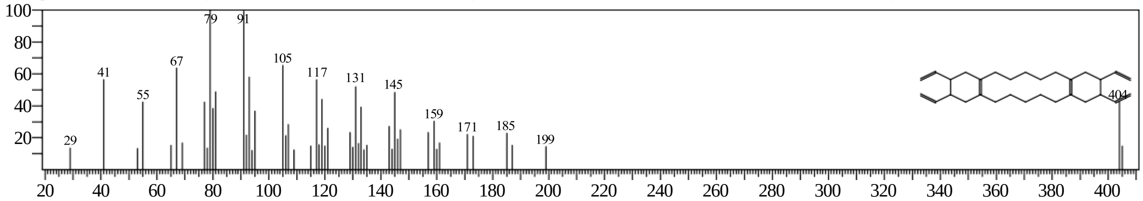

<< Target >>
Line#:105 R.Time:19.760(Scan#:3953) MassPeaks:77
RawMode:Averaged 19.755-19.765(3952-3954) BasePeak:105.20(37839)
BG Mode:Calc. from Peak

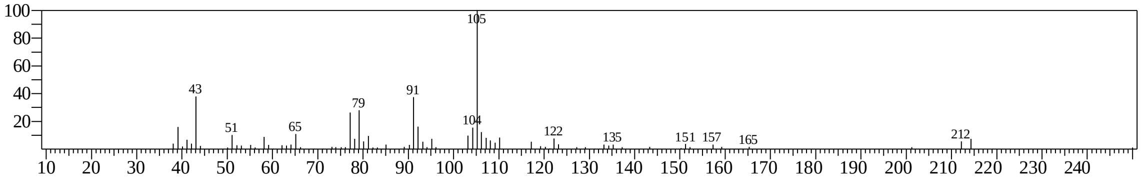

Hit#:1 Entry:12760 Library:NIST107.LIB
SI:79 Formula:C11H16 CAS:113003-13-7 MolWeight:148 RetIndex:0
CompName:Bicyclo[4.1.0]hept-3-ene, 7,7-dimethyl-3-vinyl- $$

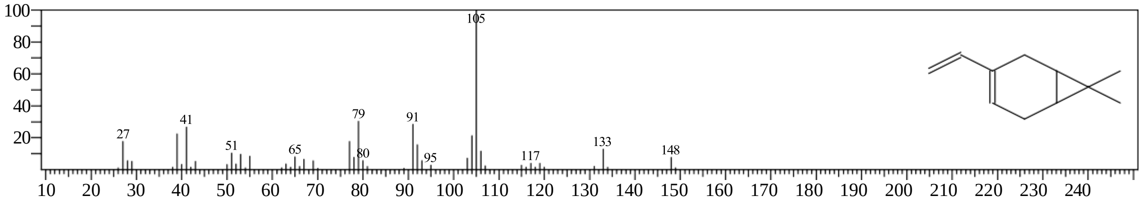

Hit#:2 Entry:62096 Library:NIST107.LIB
SI:77 Formula:C18H22O2 CAS:122-71-4 MolWeight:270 RetIndex:0
CompName:Phenylethylacetal $$ Benzene, 1,1'-[ethylidenebis(oxy-2,1-ethanediyl)]bis- $$ Acetaldehyde diphenylethylacetal $$

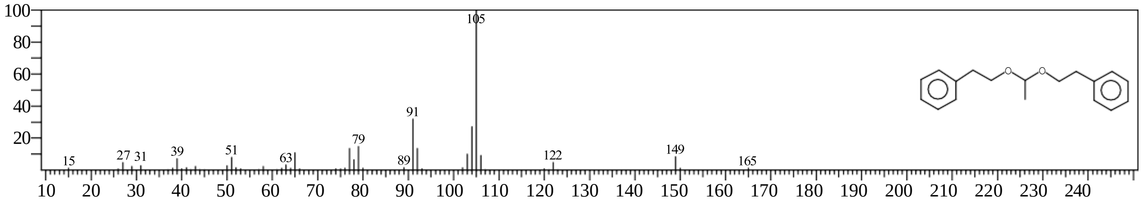

Hit#:3 Entry:20064 Library:NIST107.LIB
SI:77 Formula:C11H18O CAS:128-50-7 MolWeight:166 RetIndex:0
CompName:Bicyclo[3.1.1]hept-2-ene-2-ethanol, 6,6-dimethyl- $$ 2-Norpinene-2-ethanol, 6,6-dimethyl- $$ Homomyrtenol $$ Nopol $$ Nopol (terpene) $$

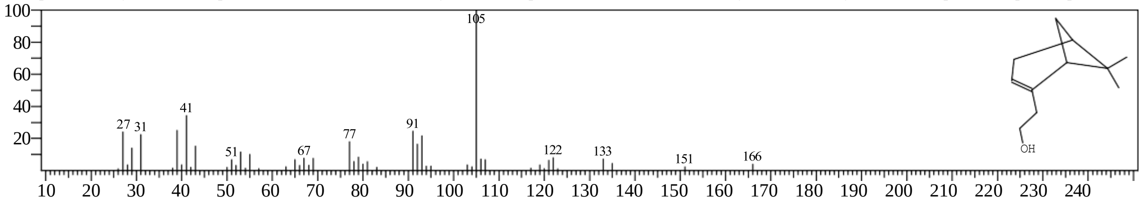

Hit#:4 Entry:38144 Library:NIST107.LIB
SI:75 Formula:C13H20O2 CAS:128-51-8 MolWeight:208 RetIndex:0
CompName:Nopyl acetate $$ Bicyclo[3.1.1]hept-2-ene-2-ethanol, 6,6-dimethyl-, acetate $$ Citroviol $$ Lignyl acetate $$ Nopol acetate $$ 2-Norpinene-2-

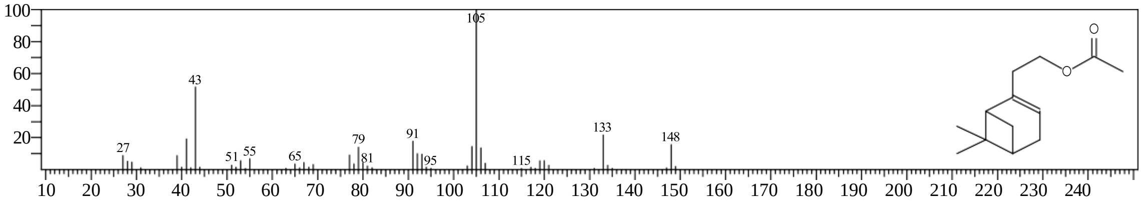

Hit#:5 Entry:8618 Library:NIST107.LIB
SI:74 Formula:C9H12O CAS:19819-98-8 MolWeight:136 RetIndex:0
CompName:Benzeneethanol, 2-methyl- $$ o-Methylphenethyl alcohol $$ 2-Methylphenethyl alcohol $$ Phenethyl alcohol, o-methyl- $$ 2-(2-Methylphenyl

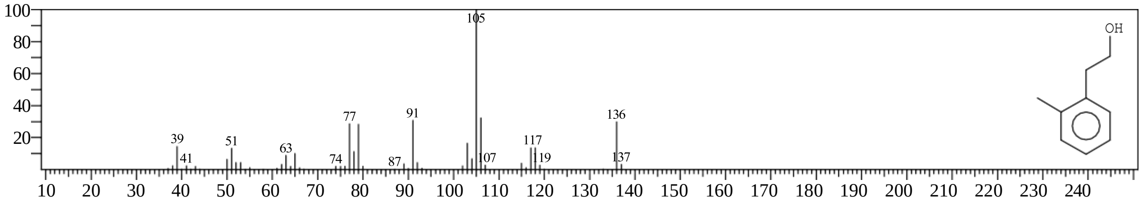

<< Target >>
Line#:106 R.Time:19.930(Scan#:3987) MassPeaks:71
RawMode:Averaged 19.925-19.935(3986-3988) BasePeak:91.15(21437)
BG Mode:Calc. from Peak

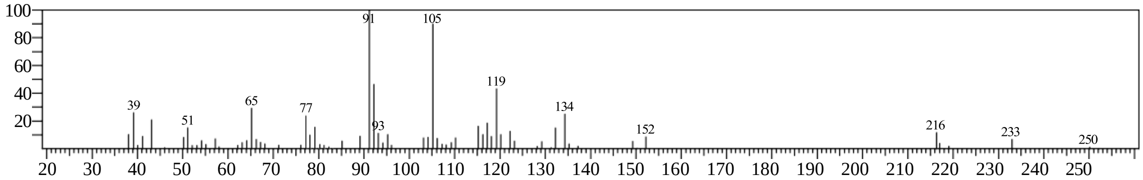

Hit#:1 Entry:14299 Library:NIST107.LIB
SI:77 Formula:C10H16O CAS:99-48-9 MolWeight:152 RetIndex:0
CompName:2-Cyclohexen-1-ol, 2-methyl-5-(1-methylethenyl)- $$ p-Mentha-1,8-dien-6-ol $$ p-Mentha-6,8-dien-2-ol $$ Carveol $$ L-p-Mentha-6,8-dien-2

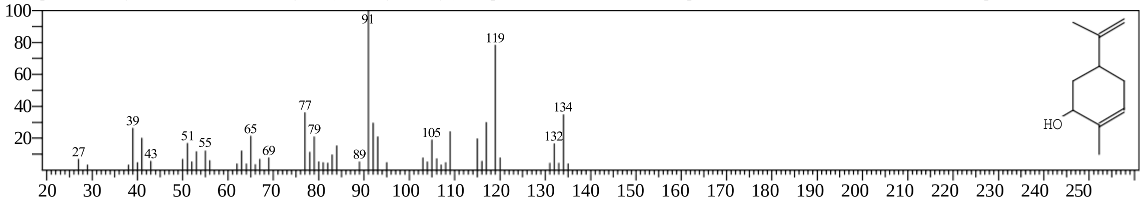

Hit#:2 Entry:8142 Library:NIST107.LIB
SI:76 Formula:C10H14 CAS:98640-10-9 MolWeight:134 RetIndex:0
CompName:3a,6-Methano-3ah-indene, 2,3,4,5,6,7-hexahydro- $$

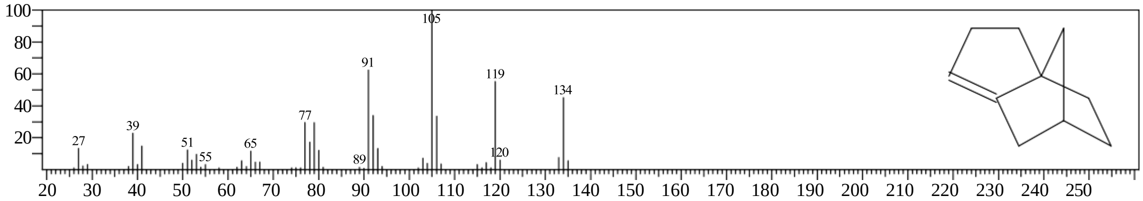

Hit#:3 Entry:14322 Library:NIST107.LIB
SI:76 Formula:C10H16O CAS:97631-68-0 MolWeight:152 RetIndex:0
CompName:Bicyclo[3.1.0]hex-3-en-2-ol, 2-methyl-5-(1-methylethyl)-, (1.alpha.,2.alpha.,5.alpha.)- $$

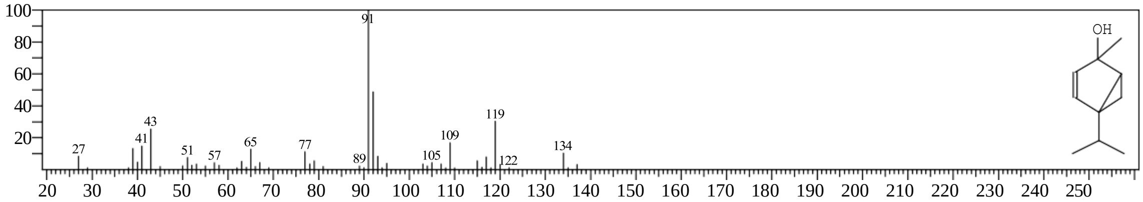

Hit#:4 Entry:8114 Library:NIST107.LIB
SI:76 Formula:C10H14 CAS:68284-24-2 MolWeight:134 RetIndex:0
CompName:Cycloheptane, 1,3,5-tris(methylene)- $$

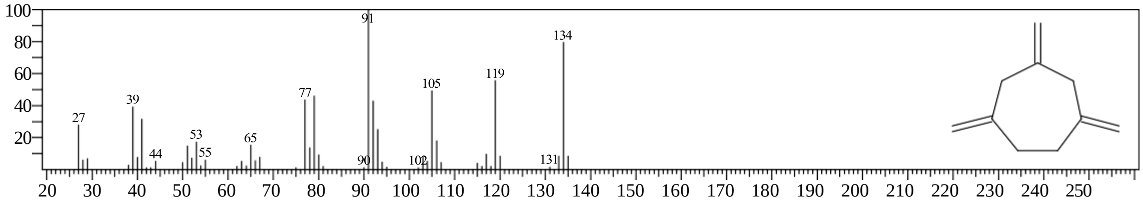

Hit#:5 Entry:14236 Library:NIST107.LIB
SI:76 Formula:C10H16O CAS:20053-58-1 MolWeight:152 RetIndex:0
CompName:2,3-Epoxycarane, (E)- $$

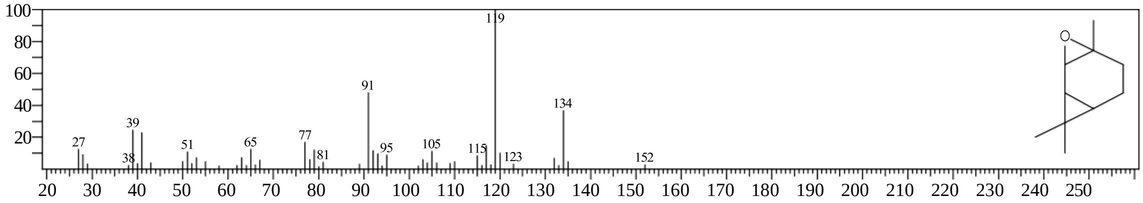

<< Target >>
Line#:107 R.Time:20.000(Scan#:4001) MassPeaks:77
RawMode:Averaged 19.995-20.005(4000-4002) BasePeak:105.15(12980)
BG Mode:Calc. from Peak

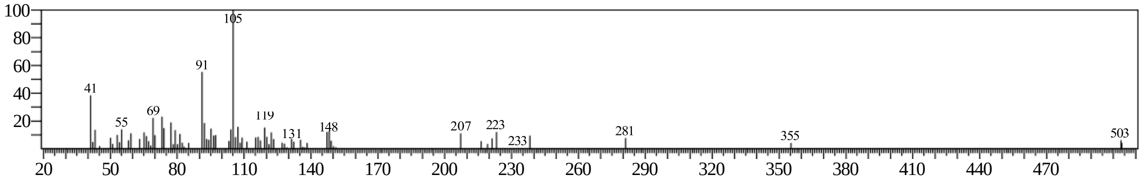

Hit#:1 Entry:12760 Library:NIST107.LIB
SI:69 Formula:C11H16 CAS:113003-13-7 MolWeight:148 RetIndex:0
CompName:Bicyclo[4.1.0]hept-3-ene, 7,7-dimethyl-3-vinyl- $$

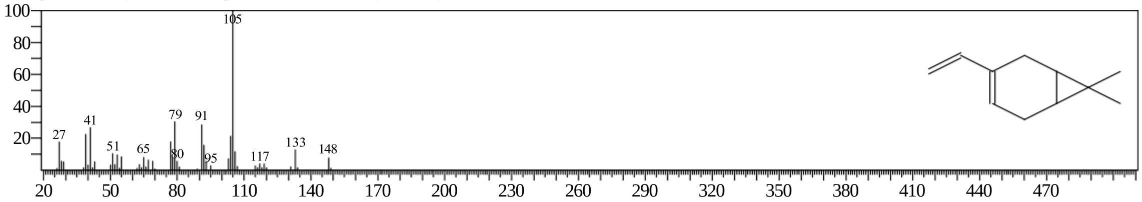

Hit#:2 Entry:24376 Library:NIST107.LIB
SI:69 Formula:C13H20 CAS:0-00-0 MolWeight:176 RetIndex:0
CompName:Megastigma-3,7(E),9-triene $$

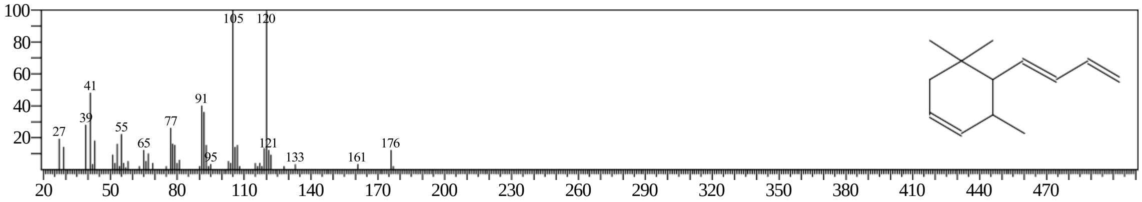

Hit#:3 Entry:20064 Library:NIST107.LIB
SI:68 Formula:C11H18O CAS:128-50-7 MolWeight:166 RetIndex:0
CompName:Bicyclo[3.1.1]hept-2-ene-2-ethanol, 6,6-dimethyl- $$ 2-Norpinene-2-ethanol, 6,6-dimethyl- $$ Homomyrtenol $$ Nopol $$ Nopol (terpene) $$

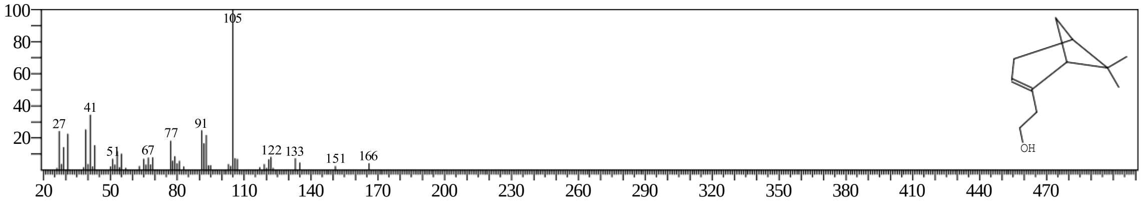

Hit#:4 Entry:30390 Library:NIST107.LIB
SI:68 Formula:C14H22 CAS:51255-61-9 MolWeight:190 RetIndex:0
CompName:5,9-Tetradecadiyne $$

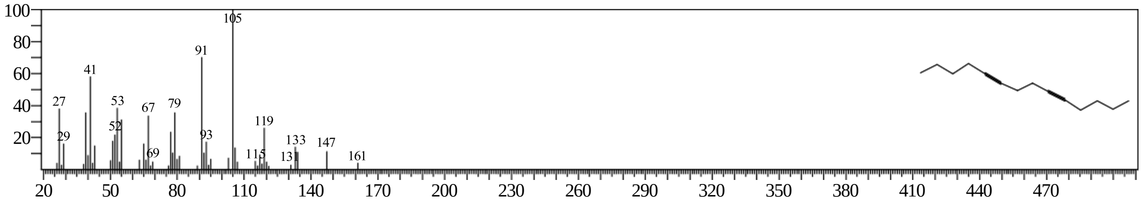

Hit#:5 Entry:12756 Library:NIST107.LIB
SI:67 Formula:C11H16 CAS:107914-86-3 MolWeight:148 RetIndex:0
CompName:6-Methyl-1,2,3,5,8,8a-hexahydronaphthalene $$

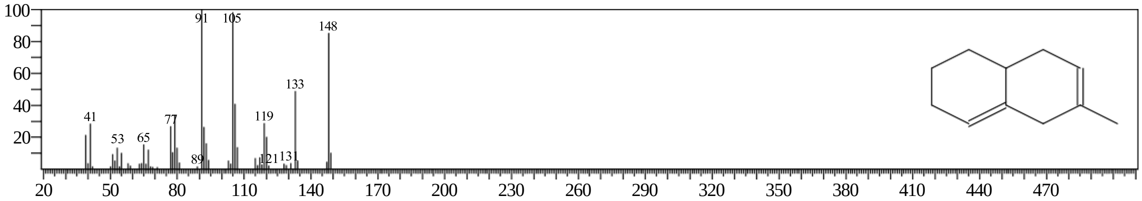

<< Target >>
Line#:108 R.Time:20.075(Scan#:4016) MassPeaks:124
RawMode:Averaged 20.070-20.080(4015-4017) BasePeak:241.35(16455)
BG Mode:Calc. from Peak

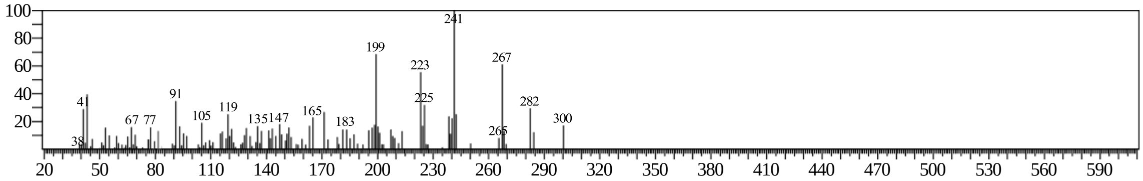

Hit#:1 Entry:65844 Library:NIST107.LIB
SI:50 Formula:C15H23Br CAS:0-00-0 MolWeight:282 RetIndex:0
CompName:.alpha.-Cedrene, 2-bromo- $$

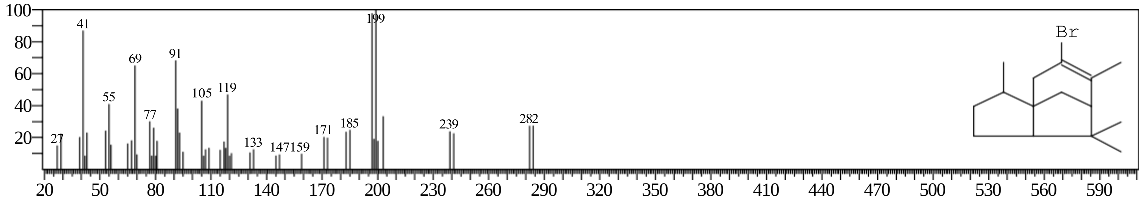

Hit#:2 Entry:78210 Library:NIST107.LIB
SI:48 Formula:C20H20O4 CAS:68873-18-7 MolWeight:324 RetIndex:0
CompName:2,7-Naphthalenediol, 1,8-di-2-propenyl-, diacetate $$

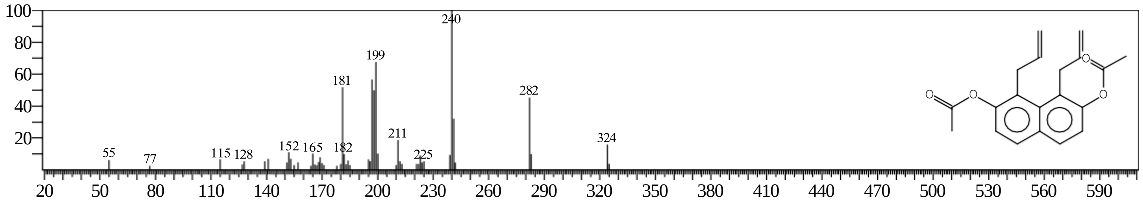

Hit#:3 Entry:106668 Library:NIST107.LIB
SI:48 Formula:C29H33Br2NO5Si CAS:133635-26-4 MolWeight:661 RetIndex:0
CompName:.alpha.-d-Ribofuranose, 1-c-(2,6-dibromo-3-pyridinyl)-5-O-[(1,1-dimethylethyl)diphenylsilyl]-2,3-O-(1-methylethylidene)- $$

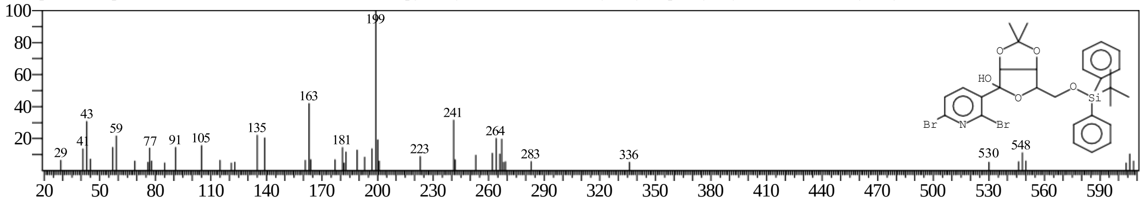

Hit#:4 Entry:105071 Library:NIST107.LIB
SI:47 Formula:C29H33Cl2NO5Si CAS:0-00-0 MolWeight:573 RetIndex:0
CompName:.alpha.D-Ribofuranose, 1(C)-(2,6-dichloro-4-pyridyl)-2,3(O)-isopropylidene-5(O)-(t-butyldiphenylsilyl)- $$

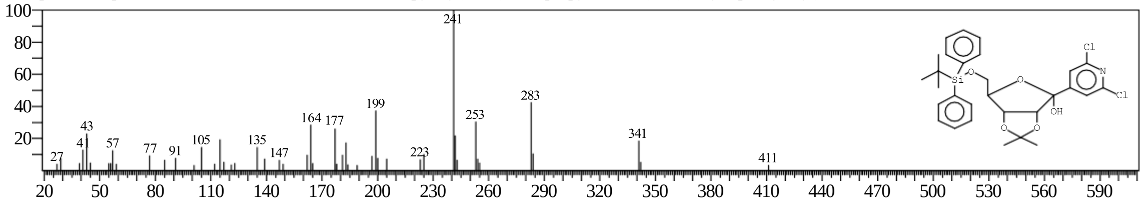

Hit#:5 Entry:71752 Library:NIST107.LIB
SI:47 Formula:C19H24O3 CAS:29461-94-7 MolWeight:300 RetIndex:0
CompName:Estra-1,3,5(10)-trien-17-one, 12-hydroxy-3-methoxy-, (12.alpha.)- $$ Estra-1,3,5(10)-trien-17-one, 12.alpha.-hydroxy-3-methoxy- $$

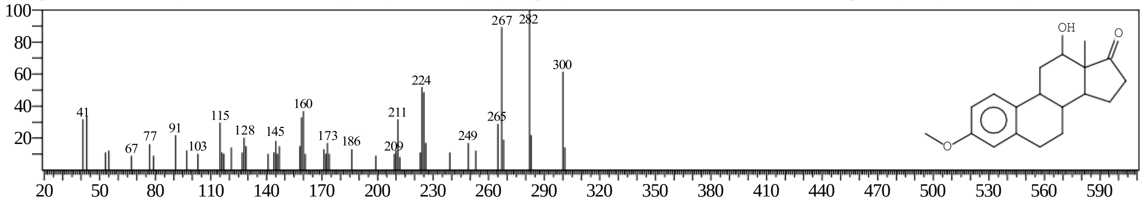

<< Target >>
Line#:109 R.Time:20.155(Scan#:4032) MassPeaks:110
RawMode:Averaged 20.150-20.160(4031-4033) BasePeak:135.20(29252)
BG Mode:Calc. from Peak

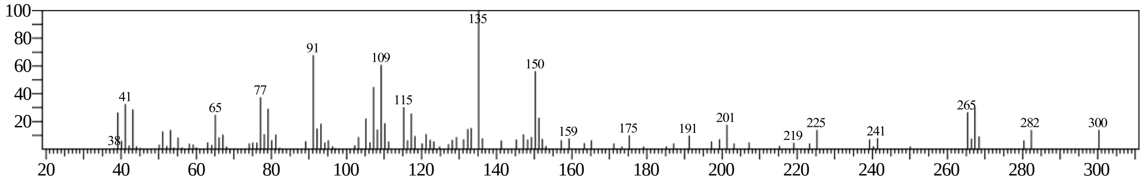

Hit#:1 Entry:31002 Library:NIST107.LIB
SI:71 Formula:C12H16O2 CAS:0-00-0 MolWeight:192 RetIndex:0
CompName:Acetic acid, 3-cyclohex-1-enyl-1-methylprop-2-ynyl ester $$

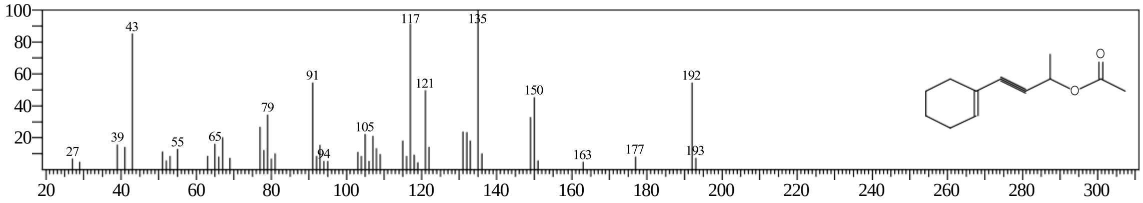

Hit#:2 Entry:13333 Library:NIST107.LIB
SI:70 Formula:C10H14O CAS:0-00-0 MolWeight:150 RetIndex:0
CompName:5-Isopropenyl-2-methylcyclopent-1-enecarboxaldehyde $$

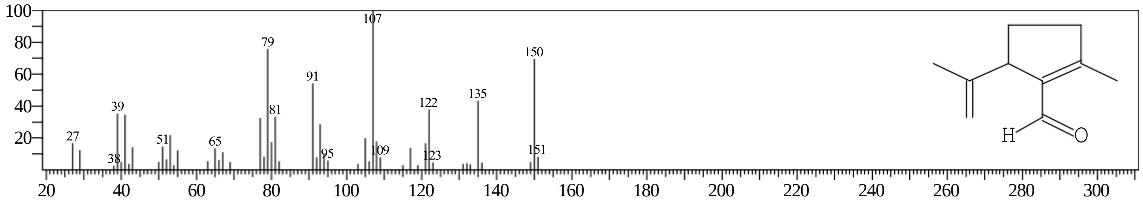

Hit#:3 Entry:13427 Library:NIST107.LIB
SI:69 Formula:C10H14O CAS:24555-40-6 MolWeight:150 RetIndex:0
CompName:Ethanone, 1-(6,6-dimethylbicyclo[3.1.0]hex-2-en-2-yl)- $$

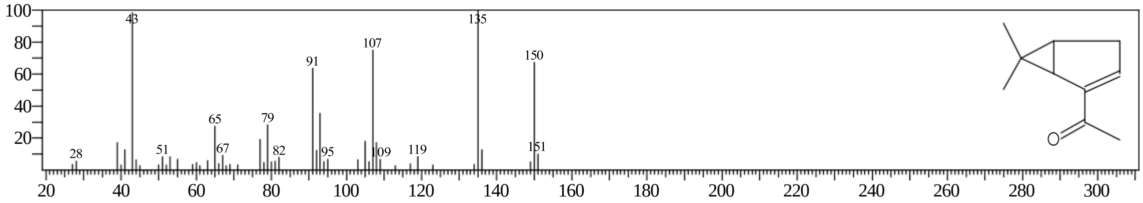

Hit#:4 Entry:13369 Library:NIST107.LIB
SI:69 Formula:C10H14O CAS:54725-16-5 MolWeight:150 RetIndex:0
CompName:2H-Inden-2-one, 1,4,5,6,7,7a-hexahydro-7a-methyl-, (S)- $$

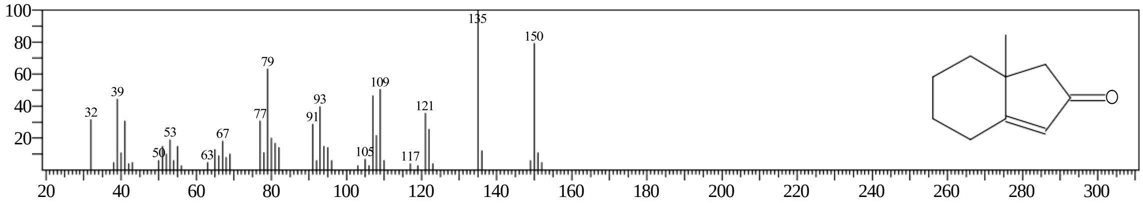

Hit#:5 Entry:13342 Library:NIST107.LIB
SI:68 Formula:C10H14O CAS:0-00-0 MolWeight:150 RetIndex:0
CompName:2-Isopropylidene-3-methylhexa-3,5-dienal $$

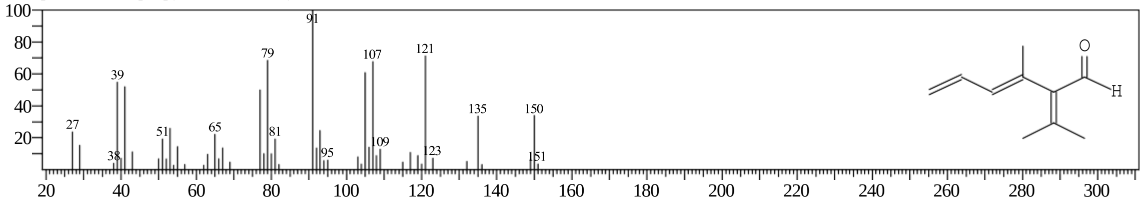

<< Target >>
Line#:110 R.Time:20.275(Scan#:4056) MassPeaks:79
RawMode:Averaged 20.270-20.280(4055-4057) BasePeak:283.35(14989)
BG Mode:Calc. from Peak

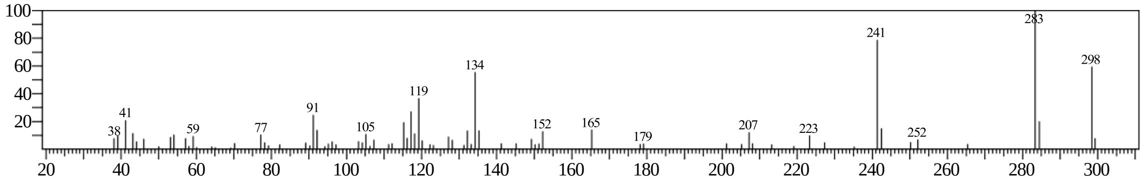

Hit#:1 Entry:71202 Library:NIST107.LIB
SI:52 Formula:C20H26O2 CAS:18326-19-7 MolWeight:298 RetIndex:0
CompName:2(1H)-Phenanthrenone, 4a,9,10,10a-tetrahydro-6-hydroxy-1,1,4a-trimethyl-7-(1-methylethyl)-, (4aS-trans)- $$ Podocarpa-1,8,11,13-tetraen-3-o

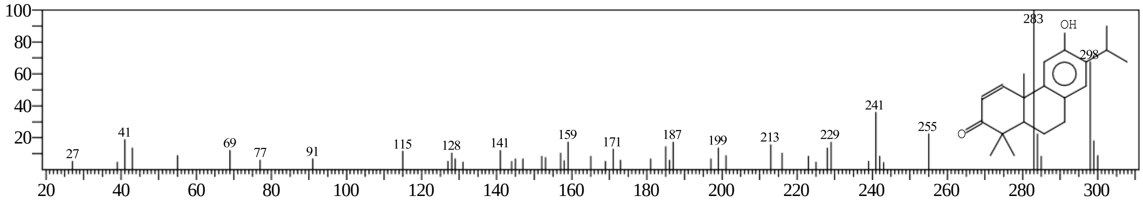

Hit#:2 Entry:66356 Library:NIST107.LIB
SI:51 Formula:C18H21NO2 CAS:106827-54-7 MolWeight:283 RetIndex:0
CompName:p-Tolylethanamine, N-[4-hydroxyhydrocinnamoyl]- $$

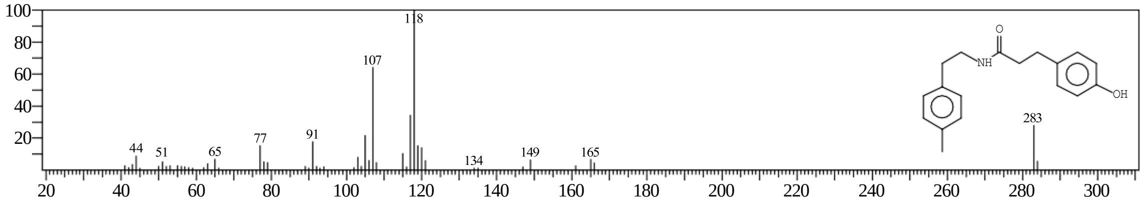

Hit#:3 Entry:71103 Library:NIST107.LIB
SI:51 Formula:C18H26Si2 CAS:1625-89-4 MolWeight:298 RetIndex:0
CompName:Biphenyl, 4,4'-bis(trimethylsilyl)- $$

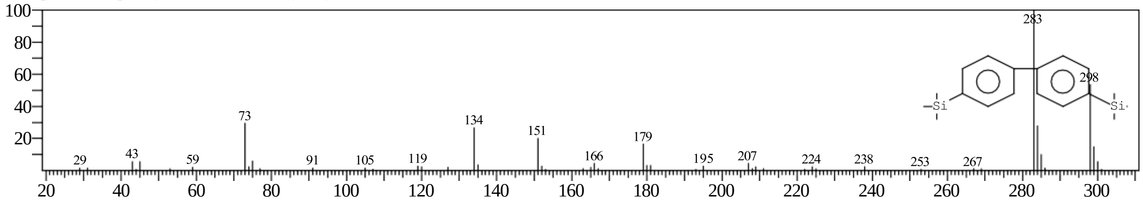

Hit#:4 Entry:66346 Library:NIST107.LIB
SI:51 Formula:C18H21NO2 CAS:0-00-0 MolWeight:283 RetIndex:0
CompName:Benzenepropylamine, N-[[(4-hydroxyphenyl)ethyl]carbonyl]- $$

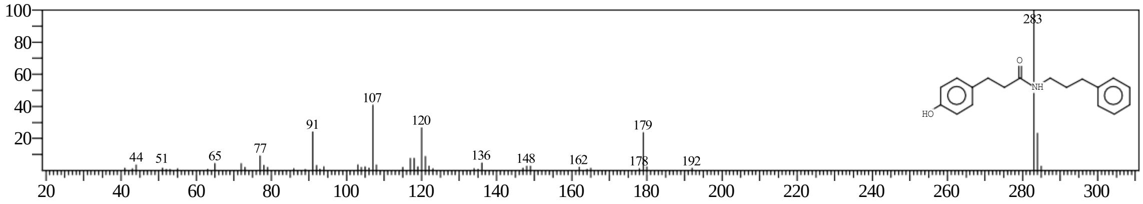

Hit#:5 Entry:70258 Library:NIST107.LIB
SI:51 Formula:C14H22Pd CAS:0-00-0 MolWeight:296 RetIndex:0
CompName:Palladium, (.eta.-3-crotyl)-pentamethylcyclopentadienyl)- $$

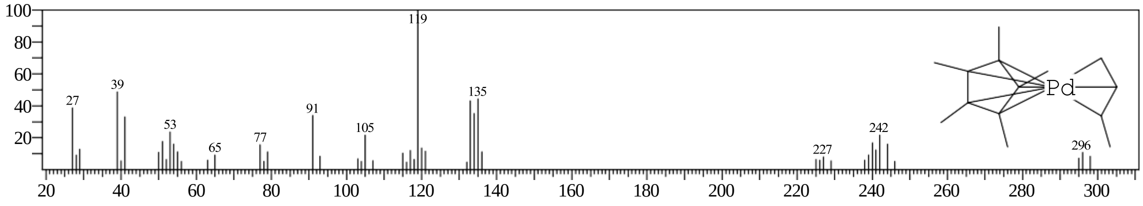

<< Target >>
Line#:111 R.Time:20.380(Scan#:4077) MassPeaks:95
RawMode:Averaged 20.375-20.385(4076-4078) BasePeak:135.25(45053)
BG Mode:Calc. from Peak

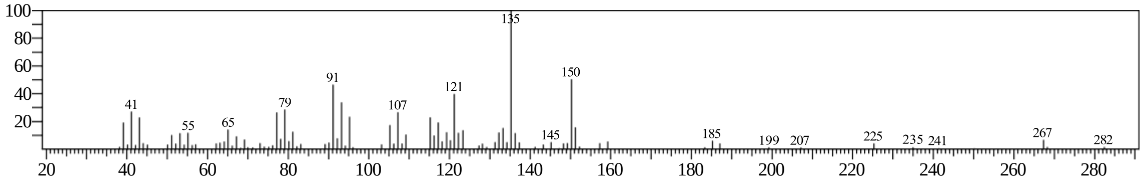

Hit#:1 Entry:31002 Library:NIST107.LIB
SI:79 Formula:C12H16O2 CAS:0-00-0 MolWeight:192 RetIndex:0
CompName:Acetic acid, 3-cyclohex-1-enyl-1-methylprop-2-ynyl ester $$

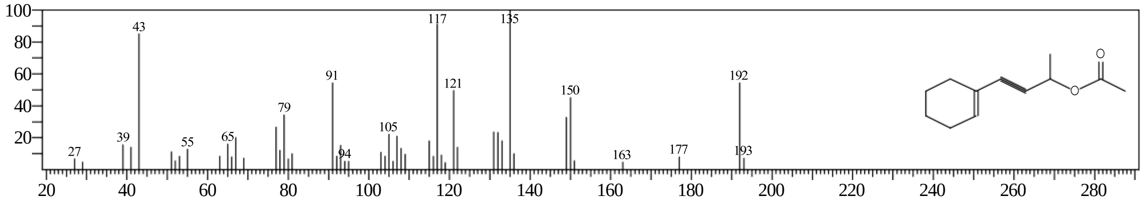

Hit#:2 Entry:13535 Library:NIST107.LIB
SI:78 Formula:C11H18 CAS:13943-77-6 MolWeight:150 RetIndex:0
CompName:Naphthalene, 1,2,3,4,4a,5,6,7-octahydro-4a-methyl- $$

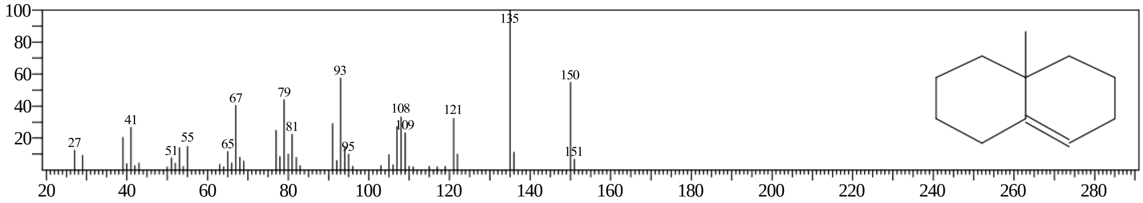

Hit#:3 Entry:13333 Library:NIST107.LIB
SI:77 Formula:C10H14O CAS:0-00-0 MolWeight:150 RetIndex:0
CompName:5-Isopropenyl-2-methylcyclopent-1-enecarboxaldehyde $$

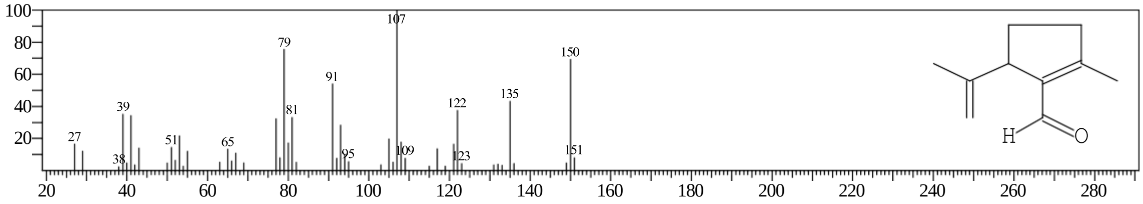

Hit#:4 Entry:13512 Library:NIST107.LIB
SI:76 Formula:C11H18 CAS:5293-90-3 MolWeight:150 RetIndex:0
CompName:Cyclohexene, 2-ethenyl-1,3,3-trimethyl- $$

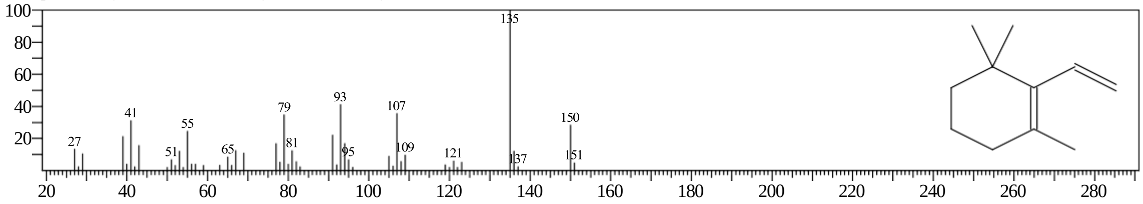

Hit#:5 Entry:37264 Library:NIST107.LIB
SI:76 Formula:C14H22O CAS:127-51-5 MolWeight:206 RetIndex:0
CompName:.alpha. Isomethyl ionone $$ 4-(2,6,6-Trimethyl 2-cyclohexen-1-yl)-3-methyl-3-buten-2-one $$ 3-Buten-2-one, 3-methyl-4-(2,6,6-trimethyl-2-cyc

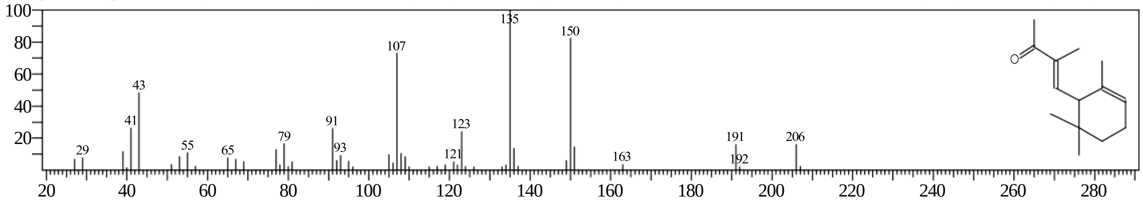

<< Target >>
Line#:112 R.Time:20.550(Scan#:4111) MassPeaks:126
RawMode:Averaged 20.545-20.555(4110-4112) BasePeak:135.25(102093)
BG Mode:Calc. from Peak

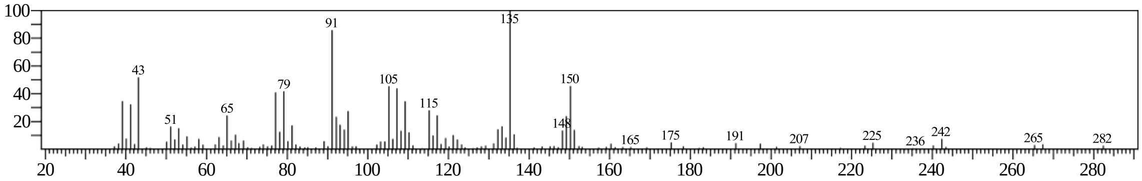

Hit#:1 Entry:13342 Library:NIST107.LIB
SI:82 Formula:C10H14O CAS:0-00-0 MolWeight:150 RetIndex:0
CompName:2-Isopropylidene-3-methylhexa-3,5-dienal $$

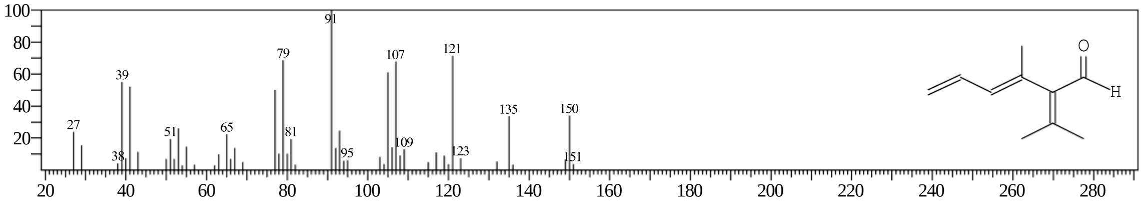

Hit#:2 Entry:31002 Library:NIST107.LIB
SI:81 Formula:C12H16O2 CAS:0-00-0 MolWeight:192 RetIndex:0
CompName:Acetic acid, 3-cyclohex-1-enyl-1-methylprop-2-ynyl ester $$

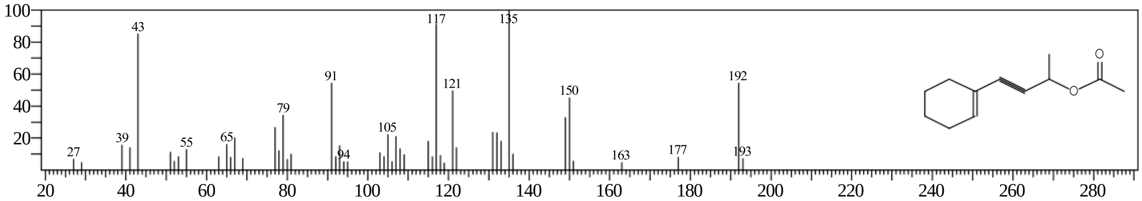

Hit#:3 Entry:13333 Library:NIST107.LIB
SI:81 Formula:C10H14O CAS:0-00-0 MolWeight:150 RetIndex:0
CompName:5-Isopropenyl-2-methylcyclopent-1-enecarboxaldehyde $$

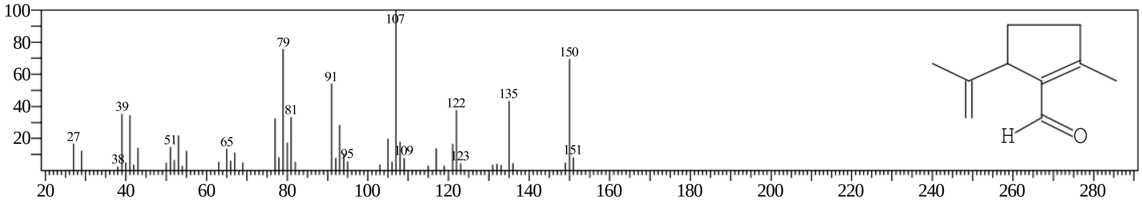

Hit#:4 Entry:13393 Library:NIST107.LIB
SI:80 Formula:C10H14O CAS:99172-18-6 MolWeight:150 RetIndex:0
CompName:3,5-Heptadienal, 2-ethylidene-6-methyl- $$

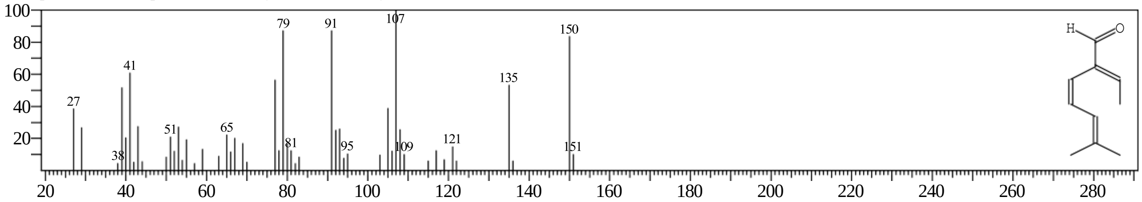

Hit#:5 Entry:13451 Library:NIST107.LIB
SI:80 Formula:C10H14O CAS:18309-32-5 MolWeight:150 RetIndex:0
CompName:D-Verbenone $$ Bicyclo[3.1.1]hept-3-en-2-one, 4,6,6-trimethyl-, (1R)- $$ 2-Pinen-4-one, (1R,5R)-(+)- $$ (+)-Verbenone $$ Bicyclo[3.1.1]hep

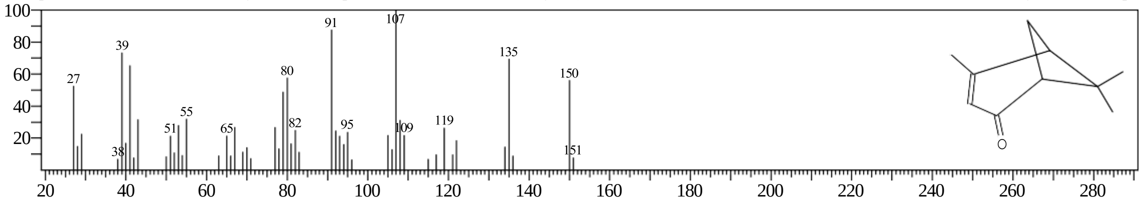

<< Target >>
Line#:113 R.Time:21.220(Scan#:4245) MassPeaks:121
RawMode:Averaged 21.215-21.225(4244-4246) BasePeak:141.20(40827)
BG Mode:Calc. from Peak

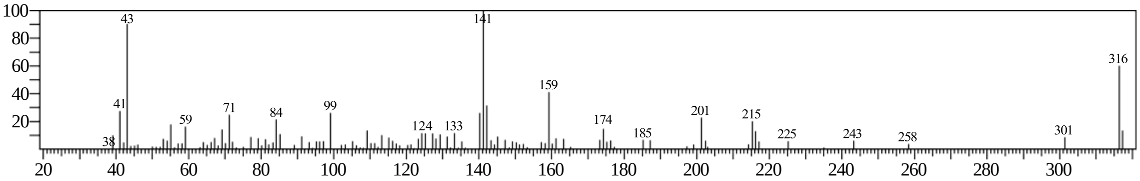

Hit#:1 Entry:27830 Library:NIST107.LIB
SI:58 Formula:C11H20O2 CAS:63922-38-3 MolWeight:184 RetIndex:0
CompName:3-Heptyne-2,5-diol, 6-methyl-5-(1-methylethyl)- $$

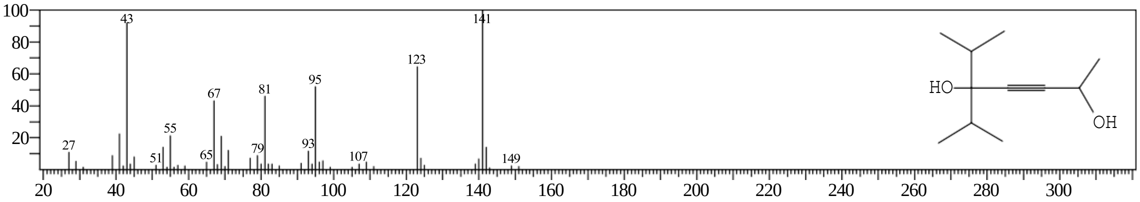

Hit#:2 Entry:88510 Library:NIST107.LIB
SI:55 Formula:C18H28O8 CAS:84781-84-0 MolWeight:372 RetIndex:0
CompName:d-Allo-dec-2-enonic acid, 5,81-anhydro-2,3,4,9-tetradeoxy-8-c-(hydroxymethyl)-3-methyl-7,8-O-(1-methylethylidene)-, methyl ester, 10-acetate

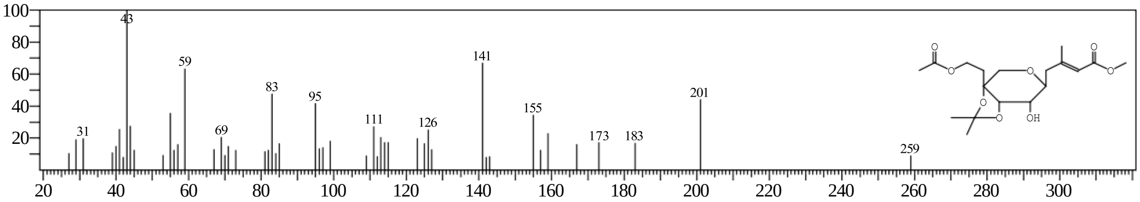

Hit#:3 Entry:34498 Library:NIST107.LIB
SI:55 Formula:C10H16O4 CAS:0-00-0 MolWeight:200 RetIndex:0
CompName:Fumaric acid, diisopropyl ester $$

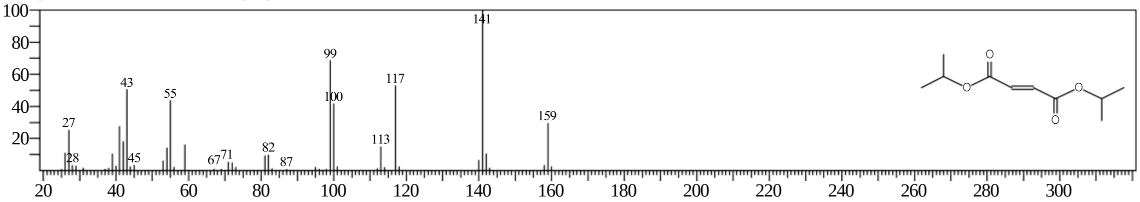

Hit#:4 Entry:53080 Library:NIST107.LIB
SI:55 Formula:C11H18O6 CAS:111572-04-4 MolWeight:246 RetIndex:0
CompName:3-Methoxycarbonylmethylhexanedioic acid, dimethyl ester $$

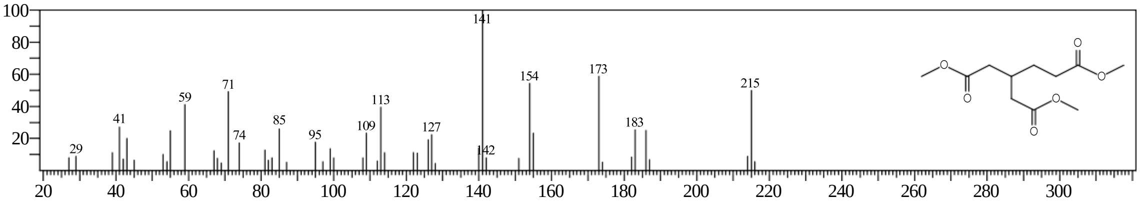

Hit#:5 Entry:62085 Library:NIST107.LIB
SI:54 Formula:C18H22O2 CAS:0-00-0 MolWeight:270 RetIndex:0
CompName:Hexyl 2-(1-naphthyl)acetate $$

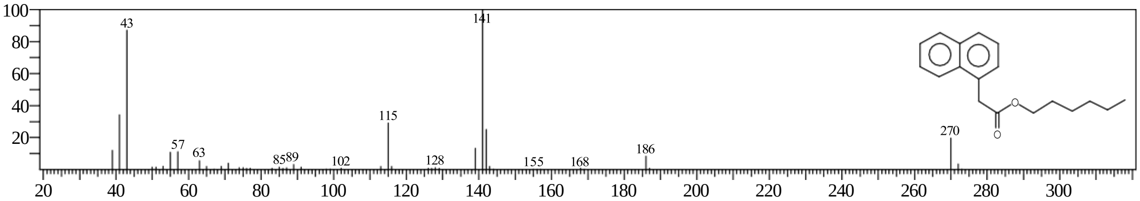

<< Target >>
Line#:114 R.Time:21.670(Scan#:4335) MassPeaks:83
RawMode:Averaged 21.665-21.675(4334-4336) BasePeak:105.15(18436)
BG Mode:Calc. from Peak

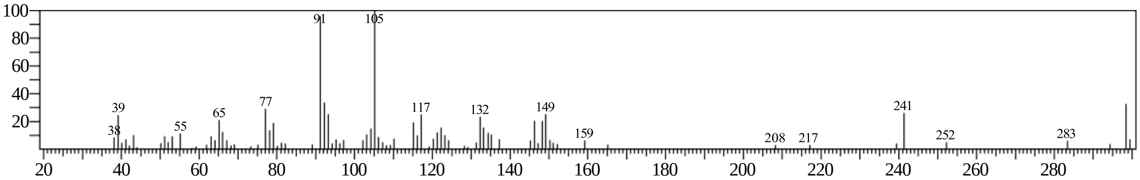

Hit#:1 Entry:12761 Library:NIST107.LIB
SI:70 Formula:C11H16 CAS:107914-88-5 MolWeight:148 RetIndex:0
CompName:7-Methyl-1,2,3,5,8,8a-hexahydronaphthalene $$

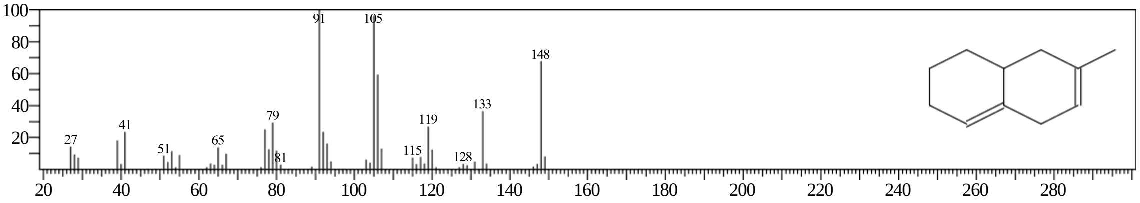

Hit#:2 Entry:12764 Library:NIST107.LIB
SI:69 Formula:C11H16 CAS:107914-89-6 MolWeight:148 RetIndex:0
CompName:2-Methyl-7-exo-vinylbicyclo[4.2.0]oct-1(2)-ene $$

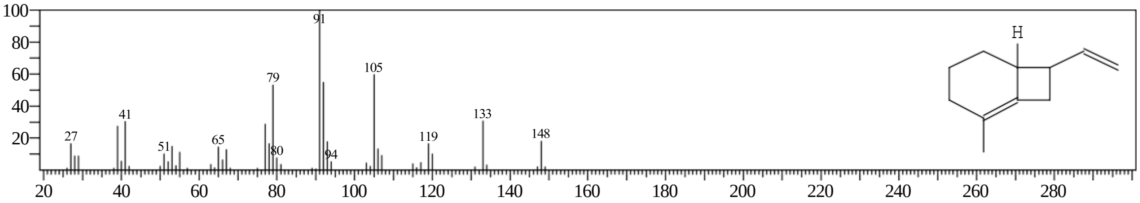

Hit#:3 Entry:12784 Library:NIST107.LIB
SI:69 Formula:C11H16 CAS:7338-49-0 MolWeight:148 RetIndex:0
CompName:1,3-Cyclopentadiene, 5-(1,3-dimethylbutylidene)- $$

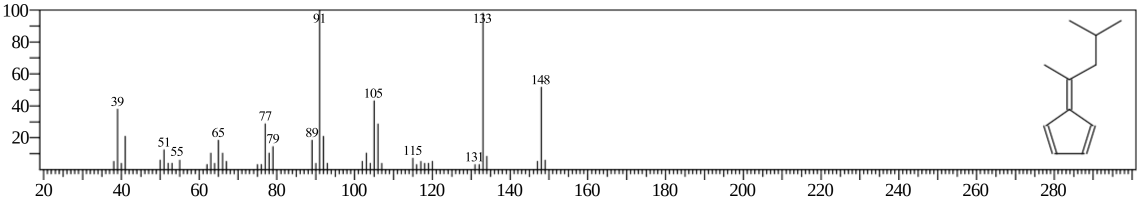

Hit#:4 Entry:12726 Library:NIST107.LIB
SI:69 Formula:C11H16 CAS:0-00-0 MolWeight:148 RetIndex:0
CompName:2-Methyl-7-endo-vinylbicyclo[4.2.0]oct-1(2)-ene $$

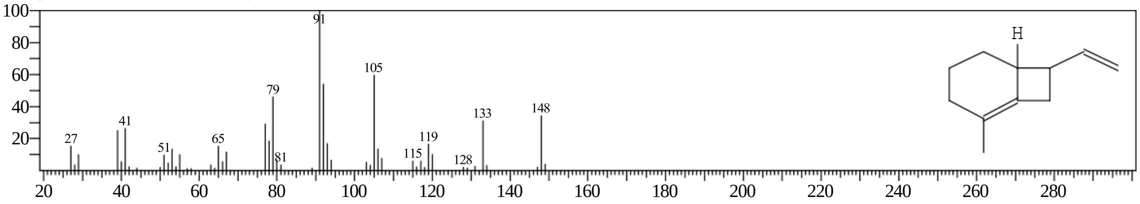

Hit#:5 Entry:12728 Library:NIST107.LIB
SI:69 Formula:C11H16 CAS:0-00-0 MolWeight:148 RetIndex:0
CompName:1-(3,3-Dimethylbuthyn-1-yl)-2,2-dimethylcyclopropene $$

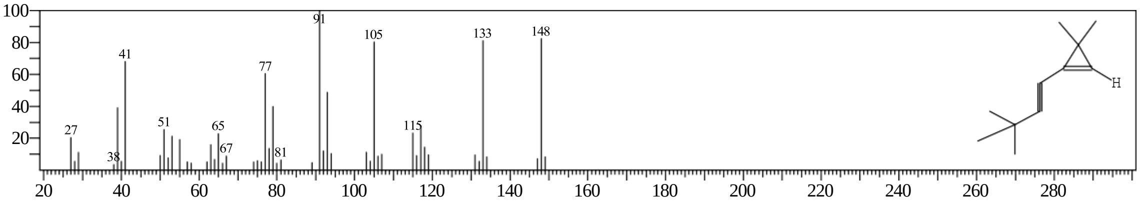

<< Target >>
Line#:115 R.Time:21.830(Scan#:4367) MassPeaks:109
RawMode:Averaged 21.825-21.835(4366-4368) BasePeak:267.35(62230)
BG Mode:Calc. from Peak

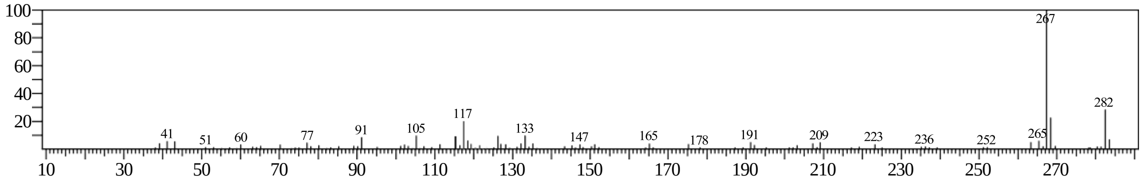

Hit#:1 Entry:66108 Library:NIST107.LIB
SI:68 Formula:C20H26O CAS:24085-65-2 MolWeight:282 RetIndex:0
CompName:Ether, bis(p-tert-butylphenyl) $$

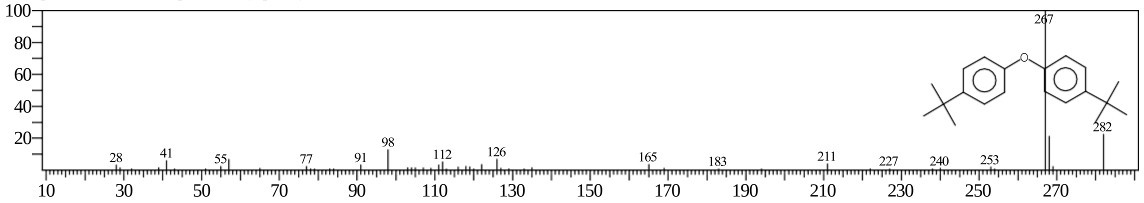

Hit#:2 Entry:66067 Library:NIST107.LIB
SI:64 Formula:C19H22O2 CAS:33745-21-0 MolWeight:282 RetIndex:0
CompName:6H-Dibenzo[b,d]pyran-1-ol, 6,6,9-trimethyl-3-propyl- $$ Cannabivarin $$

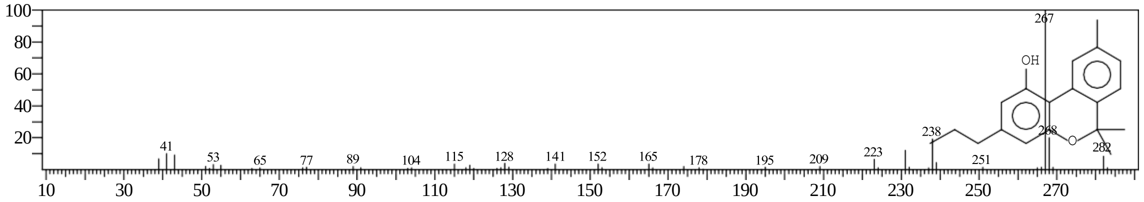

Hit#:3 Entry:65762 Library:NIST107.LIB
SI:64 Formula:C13H22O3Si2 CAS:33617-38-8 MolWeight:282 RetIndex:0
CompName:Benzaldehyde, 2,4-bis(trimethylsiloxy)- $$

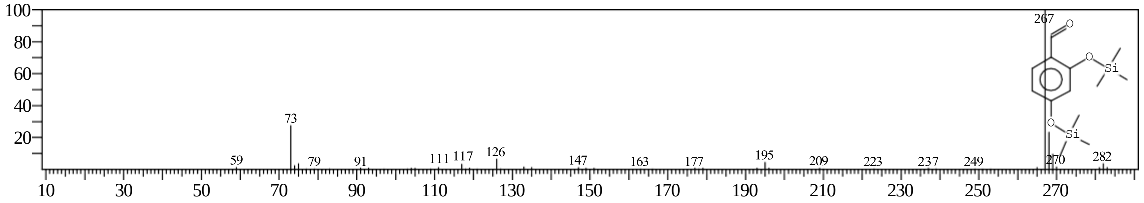

Hit#:4 Entry:66129 Library:NIST107.LIB
SI:62 Formula:C21H30 CAS:40650-56-4 MolWeight:282 RetIndex:0
CompName:1H-Trindene, 2,3,4,5,6,7,8,9-octahydro-1,1,4,4,7,7-hexamethyl- $$ 1,1,4,4,7,7-Hexamethyltrindan $$

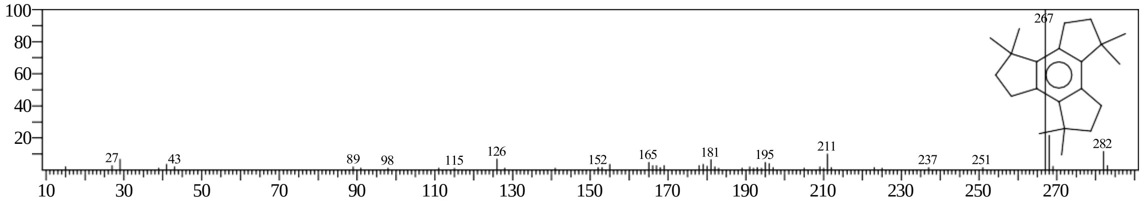

Hit#:5 Entry:65758 Library:NIST107.LIB
SI:61 Formula:C13H22O3Si2 CAS:56114-69-3 MolWeight:282 RetIndex:0
CompName:Benzaldehyde, 2,5-bis[(trimethylsilyl)oxy]- $$

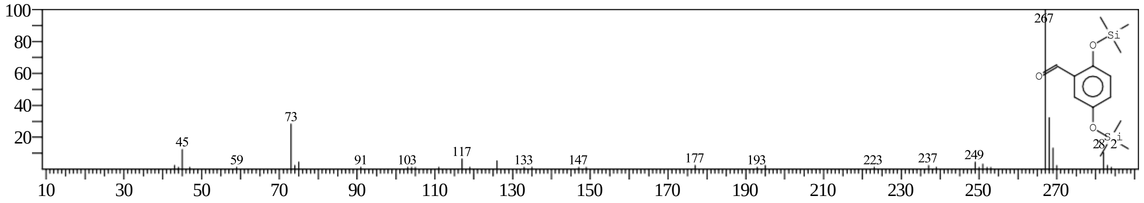

<< Target >>
Line#:116 R.Time:22.000(Scan#:4401) MassPeaks:101
RawMode:Averaged 21.995-22.005(4400-4402) BasePeak:43.10(15713)
BG Mode:Calc. from Peak

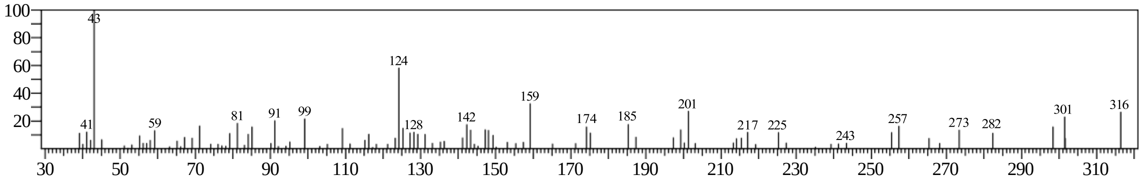

Hit#:1 Entry:80882 Library:NIST107.LIB
SI:54 Formula:C21H34O3 CAS:52340-98-4 MolWeight:334 RetIndex:0
CompName:Pregnan-20-one, 3,11-dihydroxy-, (3.beta.,5.beta.,11.alpha.)- $$

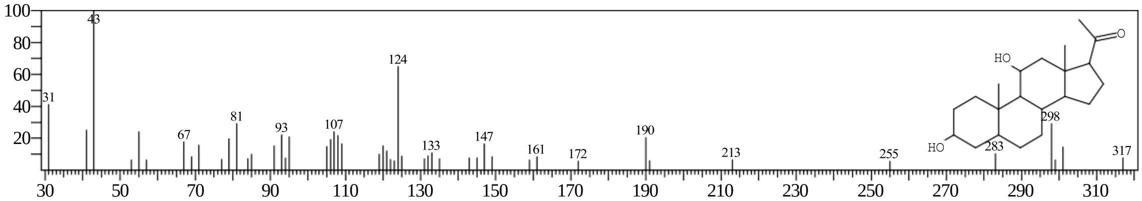

Hit#:2 Entry:80875 Library:NIST107.LIB
SI:53 Formula:C21H34O3 CAS:600-52-2 MolWeight:334 RetIndex:0
CompName:Pregnan-20-one, 3,11-dihydroxy-, (3.alpha.,5.beta.,11.alpha.)- $$ 5.beta.-Pregnan-20-one, 3.alpha.,11.alpha.-dihydroxy- $$ 3.alpha.,11.alpha.-D

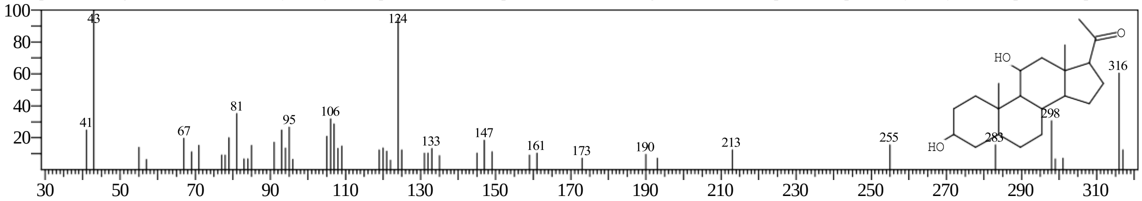

Hit#:3 Entry:80881 Library:NIST107.LIB
SI:53 Formula:C21H34O3 CAS:38398-44-6 MolWeight:334 RetIndex:0
CompName:Pregnan-20-one, 3,11-dihydroxy-, (3.alpha.,5.alpha.,11.alpha.)- $$ 5.alpha.-Pregnan-20-one, 3.alpha.,11.alpha.-dihydroxy- $$

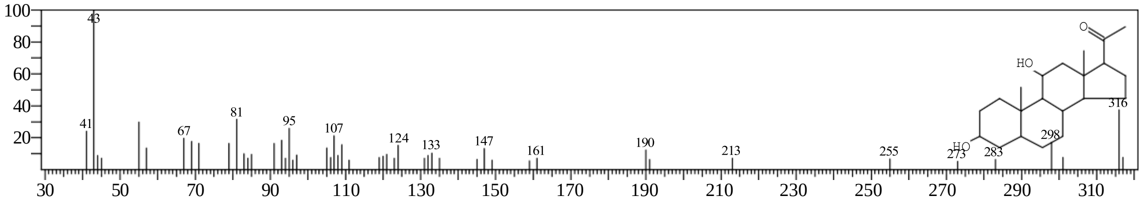

Hit#:4 Entry:80853 Library:NIST107.LIB
SI:52 Formula:C21H34O3 CAS:48200-75-5 MolWeight:334 RetIndex:0
CompName:Pregnan-20-one, 3,11-dihydroxy-, (3.beta.,5.beta.,11.beta.)- $$

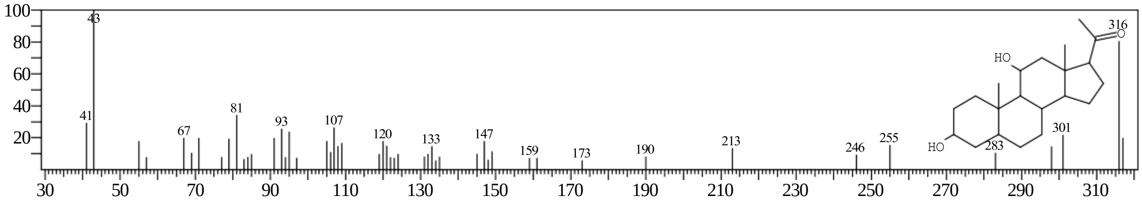

Hit#:5 Entry:80863 Library:NIST107.LIB
SI:52 Formula:C21H34O3 CAS:565-91-3 MolWeight:334 RetIndex:0
CompName:Pregnan-20-one, 3,11-dihydroxy-, (3.beta.,5.alpha.,11.alpha.)- $$ 5.alpha.-Pregnan-20-one, 3.beta.,11.alpha.-dihydroxy- $$

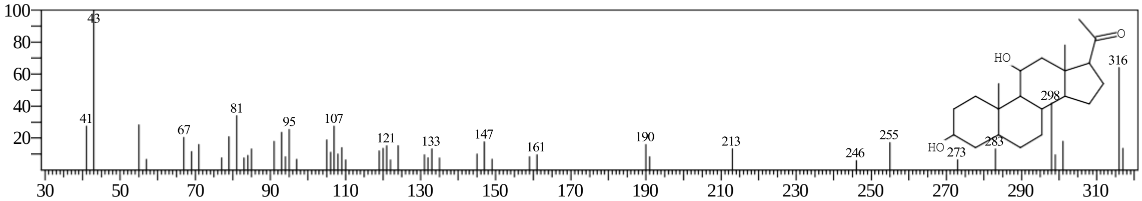

<< Target >>
Line#:117 R.Time:22.215(Scan#:4444) MassPeaks:76
RawMode:Averaged 22.210-22.220(4443-4445) BasePeak:223.30(16036)
BG Mode:Calc. from Peak

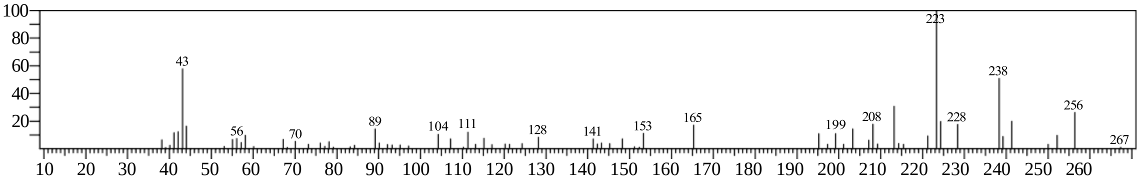

Hit#:1 Entry:50562 Library:NIST107.LIB
SI:57 Formula:C18H22 CAS:18970-30-4 MolWeight:238 RetIndex:0
CompName:4,4'-Diisopropylbiphenyl $$

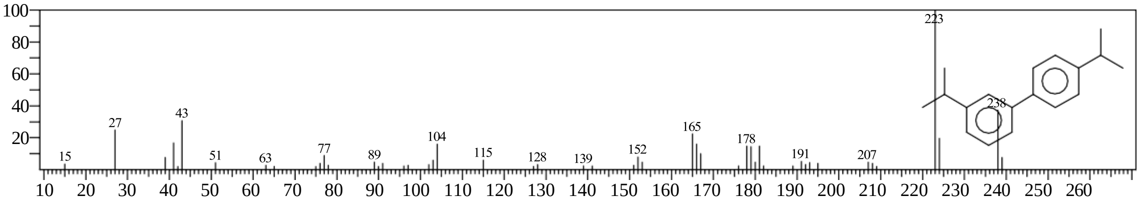

Hit#:2 Entry:50369 Library:NIST107.LIB
SI:55 Formula:C15H23Cl CAS:55538-62-0 MolWeight:238 RetIndex:0
CompName:Benzene, 2-chloro-1,3,5-tris(1-methylethyl)- $$

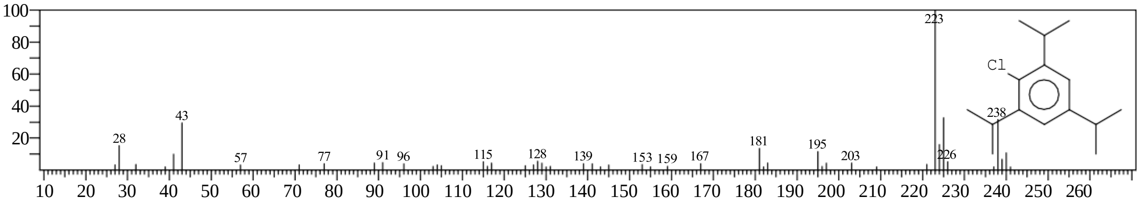

Hit#:3 Entry:50557 Library:NIST107.LIB
SI:55 Formula:C18H22 CAS:61434-46-6 MolWeight:238 RetIndex:0
CompName:3,4'-Diisopropylbiphenyl $$

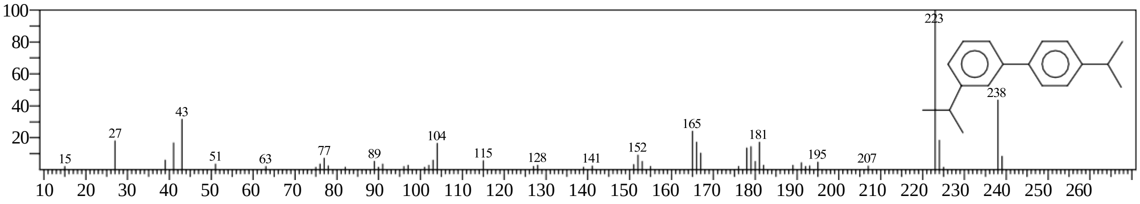

Hit#:4 Entry:50472 Library:NIST107.LIB
SI:55 Formula:C16H14O2 CAS:787-69-9 MolWeight:238 RetIndex:0
CompName:4,4'-Diacetyl biphenyl $$

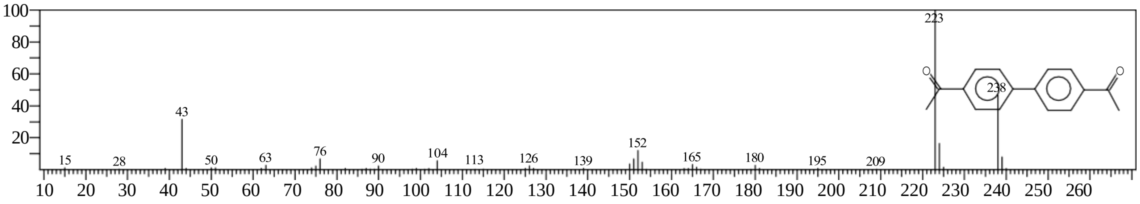

Hit#:5 Entry:50565 Library:NIST107.LIB
SI:52 Formula:C18H22 CAS:69009-90-1 MolWeight:238 RetIndex:0
CompName:1,1'-Biphenyl, bis(1-methylethyl)- $$

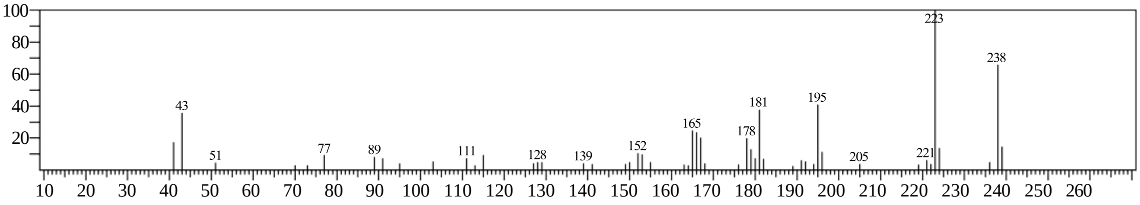

<< Target >>
Line#:118 R.Time:22.280(Scan#:4457) MassPeaks:105
RawMode:Averaged 22.275-22.285(4456-4458) BasePeak:43.10(16066)
BG Mode:Calc. from Peak

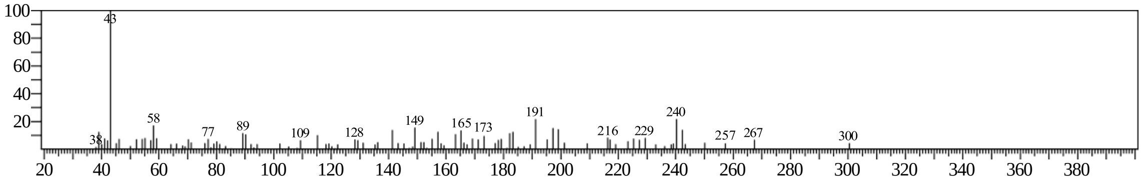

Hit#:1 Entry:92220 Library:NIST107.LIB
SI:52 Formula:C28H42O CAS:0-00-0 MolWeight:394 RetIndex:0
CompName:Anthiaergosta-5,7,9-trien-3-one $$

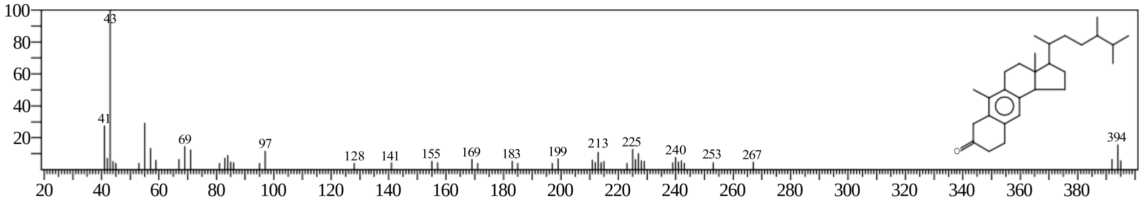

Hit#:2 Entry:64015 Library:NIST107.LIB
SI:51 Formula:C16H20O4 CAS:0-00-0 MolWeight:276 RetIndex:0
CompName:5,8-Dimethyl-1,4,6,7-tetrahydronaphtalindicarbonic acid,1,4-dimethyl ester $$

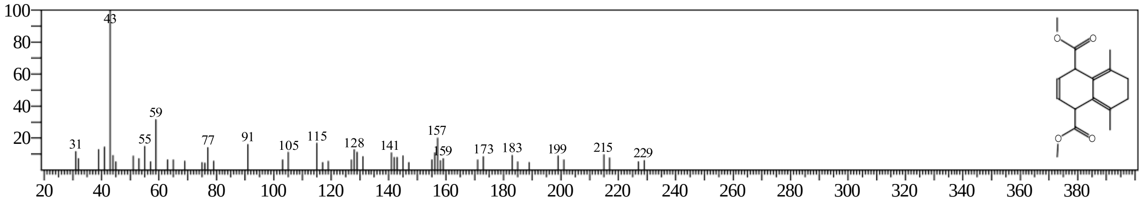

Hit#:3 Entry:66169 Library:NIST107.LIB
SI:51 Formula:C11H10BrNO3 CAS:0-00-0 MolWeight:283 RetIndex:0
CompName:3-Acetonyl-5-bromo-3-hydroxy-2-indolinone $$

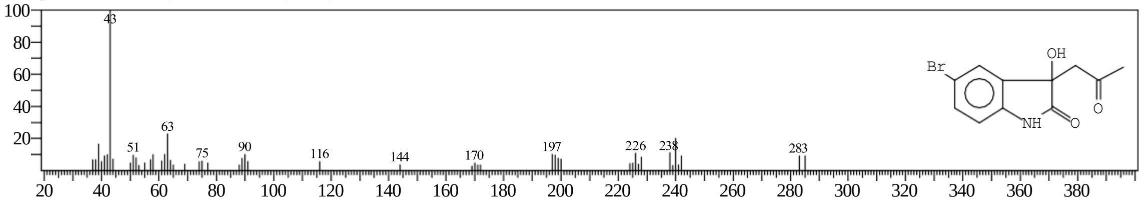

Hit#:4 Entry:50838 Library:NIST107.LIB
SI:50 Formula:C6H14BBrS2 CAS:89449-88-7 MolWeight:240 RetIndex:0
CompName:Borane, bromobis[(1-methylethyl)thio]- $$

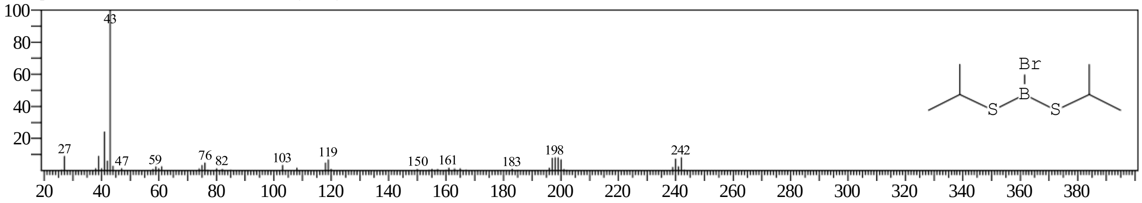

Hit#:5 Entry:75210 Library:NIST107.LIB
SI:50 Formula:C21H28O2 CAS:0-00-0 MolWeight:312 RetIndex:0
CompName:17-Noranthiaergostan-5,7,9-trien-3-ol, 17-acetyl $$

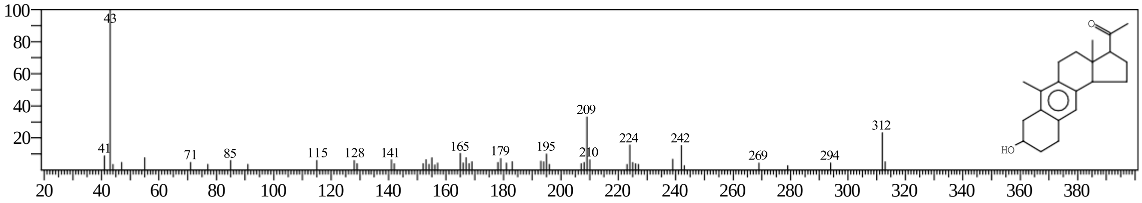

<< Target >>
Line#:119 R.Time:22.320(Scan#:4465) MassPeaks:161
RawMode:Averaged 22.315-22.325(4464-4466) BasePeak:283.35(42606)
BG Mode:Calc. from Peak

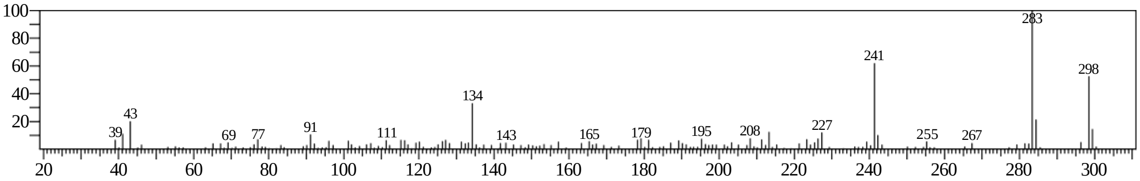

Hit#:1 Entry:71202 Library:NIST107.LIB
SI:63 Formula:C20H26O2 CAS:18326-19-7 MolWeight:298 RetIndex:0
CompName:2(1H)-Phenanthrenone, 4a,9,10,10a-tetrahydro-6-hydroxy-1,1,4a-trimethyl-7-(1-methylethyl)-, (4aS-trans)- $$ Podocarpa-1,8,11,13-tetraen-3-o

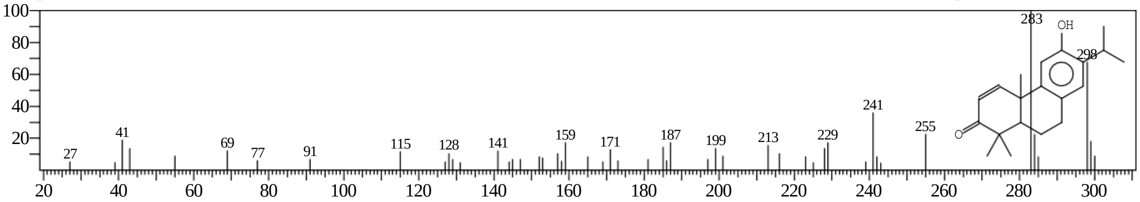

Hit#:2 Entry:71224 Library:NIST107.LIB
SI:60 Formula:C21H18Si CAS:0-00-0 MolWeight:298 RetIndex:0
CompName:Benzene, 1-(4-phenyl-1,3-butadiynyl)-3-[2-(trimethylsilyl)ethynyl]- $$

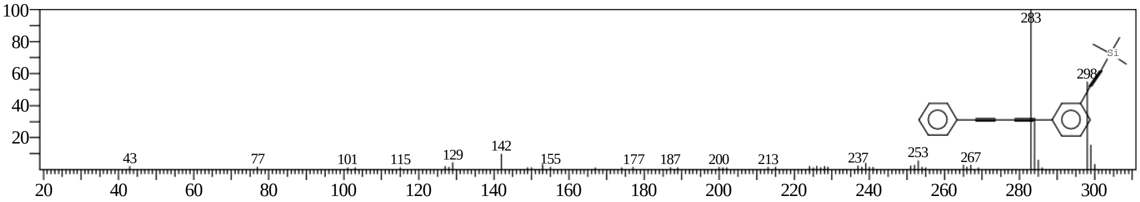

Hit#:3 Entry:71103 Library:NIST107.LIB
SI:58 Formula:C18H26Si2 CAS:1625-89-4 MolWeight:298 RetIndex:0
CompName:Biphenyl, 4,4'-bis(trimethylsilyl)- $$

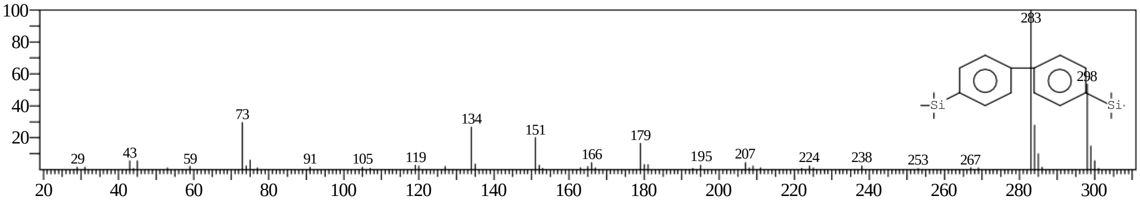

Hit#:4 Entry:71231 Library:NIST107.LIB
SI:57 Formula:C22H34 CAS:22306-30-5 MolWeight:298 RetIndex:0
CompName:Anthracene, 1,2,3,4,5,6,7,8-octahydro-1,1,4,4,5,5,8,8-octamethyl- $$

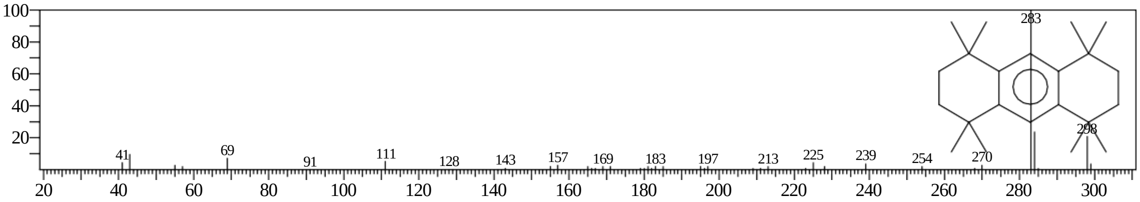

Hit#:5 Entry:71236 Library:NIST107.LIB
SI:56 Formula:C23H22 CAS:0-00-0 MolWeight:298 RetIndex:0
CompName:10,11-(3',6'-Dimethylbenzo)[3.2]paracyclophane $$

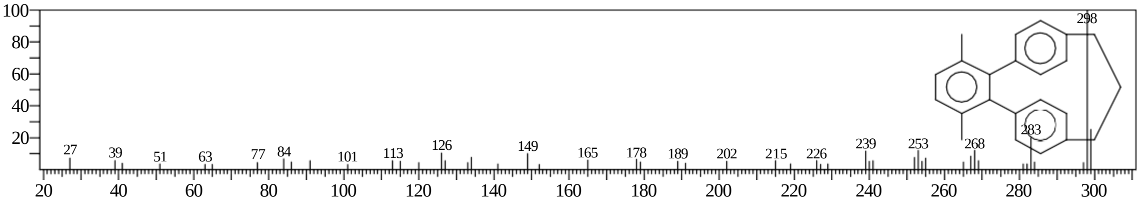

Supplement: Supplementary file 1 [file gels-11-00400-s001.zip › gels-3585126-supplementary.pdf]
